# Education as a protective factor against lung cancer: A comprehensive Mendelian randomization analysis

**DOI:** 10.1097/MD.0000000000045651

**Published:** 2025-11-07

**Authors:** Yirong Wang, Xiaoqin Wang, Guodong Sun, Pengcheng Feng

**Affiliations:** a Department of Basic Medicine, Changzhi Medical College, Changzhi, China; b Department of Anesthesiology, Changzhi Medical College, Changzhi, China.

**Keywords:** co-localization analysis, education, hyprcoloc, lung cancer, Mendelian randomization, METAL

## Abstract

Lung cancer remains a leading cause of global morbidity and mortality, imposing a significant healthcare burden worldwide. To comprehensively investigate potential risk factors for lung cancer and provide a theoretical basis for its prevention and treatment, we utilized publicly available genome-wide association study data of European ancestry. We treated various traits as exposures and lung cancer as the outcome. Causal relationships were assessed using 5 Mendelian randomization (MR) methods: inverse-variance weighted, constrained maximum likelihood and model averaging, debiased inverse-variance weighted, contamination mixture, and Mendelian randomization-robust adjusted profile score. We subsequently performed meta-analysis and median-based MR to synthesize the roles of different risk factors in lung cancer development. Following outlier removal via radial MR and MR analyses, we identified 88, 113, 248, and 232 exposure datasets showing causal associations with lung adenocarcinoma, small cell lung cancer, lung cancer overall, and squamous cell lung cancer, respectively. Further integration via meta-analysis and median MR revealed that higher educational attainment may reduce lung cancer risk by delaying age at first sexual intercourse, increasing age at first childbirth, lowering body mass index and adiposity, and reducing smoking frequency. Our comprehensive analysis suggests that education may play a protective role against lung carcinogenesis, potentially mediated through behavioral changes such as reduced smoking and modifications in physical health indicators including body mass index and adiposity.

## 1. Introduction

Lung cancer remains one of the most lethal and prevalent diseases in modern medicine, securing its position as the primary driver of cancer-related fatalities. Pathologically, lung cancer is bifurcated into small cell lung cancer (SCLC) and non-small cell lung cancer.^[1–3]^ Non-small cell lung cancer accounts for approximately 85% of all lung cancer cases and is further subdivided into lung adenocarcinoma (LUAD) and squamous cell carcinoma.^[4–6]^ Patients are frequently diagnosed at an advanced stage or after metastasis has occurred, a consequence of the disease’s aggressive progression. This late diagnosis contributes significantly to the high mortality and generally poor prognosis associated with lung cancer. Moreover, the development of resistance to targeted therapies further complicates treatment, adversely affecting both patient quality of life and clinical outcomes. Therefore, early detection and research focused on overcoming drug resistance are critically important, as they hold the key to enhancing therapeutic efficacy and improving survival.

Mendelian randomization (MR) is a genetic epidemiological method used to infer causal relationships between exposures and outcomes by employing genetic variants – typically single nucleotide polymorphisms (SNPs) – as instrumental variables (IVs). This approach leverages the random allocation of alleles during gamete formation, which largely avoids confounding biases from environmental and behavioral factors. Building on this framework, phenotype-wide MR utilizes a systematically curated and rigorously classified array of phenotypes to conduct large-scale causal inference across diverse traits. It has shown great utility in identifying potential risk factors contributing to lung cancer development and progression.

Lung cancer remains one of the most lethal malignancies worldwide, underscoring the urgent need for etiological studies to inform prevention and early detection strategies. The primary objective of this study was to systematically identify and prioritize modifiable risk factors influencing lung carcinogenesis, thereby providing evidence-based guidance for preventive interventions. Furthermore, our analysis serves to validate and complement previously reported associations, strengthening the epidemiological foundation underlying lung cancer development. To this end, we assessed putative causal relationships between 23,000 publicly available traits derived from large-scale genome-wide association study (GWAS) and major subtypes of lung cancer.^[7–10]^ We implemented meta-analysis techniques together with robust median-based MR methods to enhance the reliability of causal estimates. Additionally, co-localization analysis was conducted to examine whether the observed associations were likely driven by shared causal genetic variants rather than linkage disequilibrium (LD).

## 2. Materials and methods

**Figure 1.**
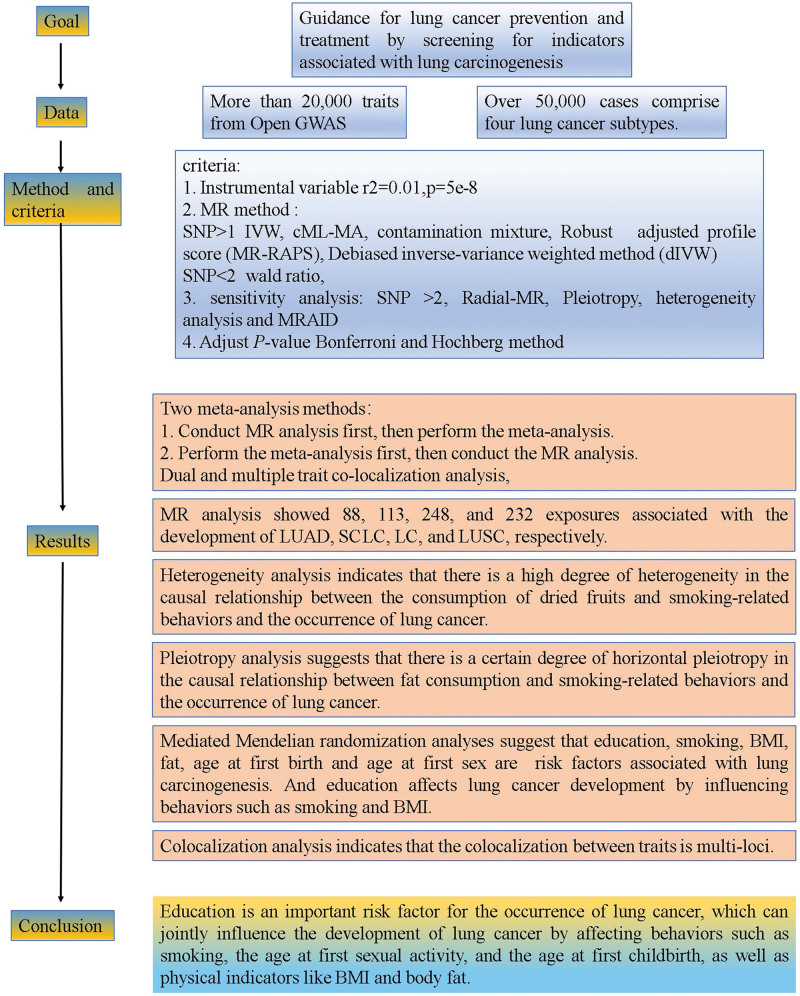
The research roadmap of this study. BMI = body mass index, cML-MA = constrained maximum likelihood and model averaging, dIVW = debiased inverse-variance weighted, GWAS = genome-wide association study, IVW = inverse-variance weighted, LC = lung cancer, LUAD = lung adenocarcinoma, LUSC = lung squamous cell carcinoma, MRAID = Mendelian randomization with automated instrument determination, MR = Mendelian randomization, MR-RAPS = Mendelian randomization-robust adjusted profile score, SCLC = small cell lung cancer, SNP = single nucleotide polymorphism.

Ethical approval for the original data collection was obtained from the relevant committee prior to commencement; therefore, further ethical review for this secondary analysis was not necessary. A graphical abstract of the overall research trajectory is provided in Figure 1. Our analysis was specifically restricted to datasets from European populations, and any pertaining to lung cancer were rigorously excluded to minimize confounding.

### 2.1. Genetic instruments for phenotypes

The selection of IVs is critical to the reliability of the analysis results. We applied the following criteria, selecting SNPs as IVs only when they satisfied all conditions below:

Genome-wide significant association with the phenotype (*P* < 5 × 10⁻⁸);

Linkage disequilibrium (LD) parameters: *r*² ≤ 0.01 and window distance ≥ 1000 kb;

*F*-statistic ≥ 10 for each SNP to minimize the influence of weak IVs.

### 2.2. MR-pheWAS based on OpenGWAS database

The main source of exposure data for this MR analysis was based on the OpenGWAS database, which contains 18 dataset sources including gene expression databases (eqtl-a), multiple phenotype databases (bbj-a, finn-b, ieu-a, ieu-b, ukb-a, ukb-b, ukb-d, and ukb-e), multiple metabolic databases (met-a, met-c, and met-d), multiple protein expression databases (prot-a, prot-b, and prot-c), and immune system databases (met-b),^[7–10]^ When the number of participants exceeds 1000 and European population, it is considered a valid exposure dataset.

### 2.3. Lung cancer GWAS data

The outcome data were primarily derived from a large-scale GWAS meta-analysis conducted by McKay et al, which integrated multiple datasets comprising 29,266 lung cancer cases and 56,450 controls. All samples included in these datasets were of European ancestry.

### 2.4. Mendelian randomization analysis

The inverse-variance weighted (IVW) method served as the primary approach for causal inference, supplemented by several robust MR techniques including constrained maximum likelihood and model averaging (cML-MA), contamination mixture (ConMix), Mendelian randomization-robust adjusted profile score (MR-RAPS), and debiased inverse-variance weighted (dIVW). The cML-MA method, which demonstrates superior performance over MR-Egger in certain settings, was employed to assess horizontal pleiotropy. ConMix, MR-RAPS, and dIVW were applied to mitigate biases due to weak IVs and horizontal pleiotropy.^[11–14]^ Notably, ConMix yields higher accuracy when the number of IVs exceeds 100.

A significance threshold of *P* < .05 after Bonferroni–Hochberg correction was applied to IVW results. For other methods, an uncorrected threshold of *P* < .05 was used.

### 2.5. Sensitivity analysis

Sensitivity analyses were conducted to validate the robustness of the MR findings. Outliers among IVs were identified and removed using the radial MR package,^[15]^ provided the number of SNP instruments exceeded 2. A result was considered statistically significant at *P* < .05, with significance indicating possible horizontal pleiotropy.

Heterogeneity was quantified using the *Q*-statistic and *I*^2^ values. Moderate heterogeneity was defined as a *Q*-value below 0.05 accompanied by an *I*^2^ between 0.25 and 0.5, while high heterogeneity was indicated by an *I*^2^ exceeding 0.5. The MRAID framework was further utilized to evaluate the influence of horizontal pleiotropy and to assess the robustness of the causal estimates.^[16]^

### 2.6. Meta and mediated Mendelian randomization analysis

Previous studies have indicated that education is one of the risk factors for lung cancer development. To elucidate the intrinsic mechanisms through which education influences lung carcinogenesis, we conducted a 2-step MR mediation analysis. This mediation analysis employed educational attainment as the exposure, with smoking, body mass index (BMI), age at first sexual intercourse, and age at first birth as mediators, and 4 lung cancer subtypes as outcomes. The METAL software was used to aggregate multiple datasets for the same trait. The analytical process resembled the earlier 2-sample MR analysis, wherein the radial MR package was utilized to remove outliers from the IVs. The 2-step method was applied to calculate the effect of the exposure on the mediators (α) and the effect of the mediators on the outcomes (β), with the point estimate of the mediation effect subsequently derived using the product method (αβ). The total effect (f_b) was obtained from a separate MR analysis with smoking as the exposure and lung cancer as the outcome. Finally, the standard error (SE) and confidence interval were estimated using the Delta method, and the proportion mediated was calculated as follows: Proportion mediated = (αβ)/f_b

The variance of the indirect effect was approximated as:

Var(α) = SE(α)², Var(β) = SE(β)²

SE(αβ) = √[Var(αβ)] ≈ √[α² × Var(β) + β² × Var(α)]

The 95% confidence interval was calculated as:

95% CI = (αβ) ± 1.96 × SE(αβ)

### 2.7. Co-localization analysis

The validity of MR analysis is highly dependent on the strength and number of IVs (IVs) used. To minimize potential bias introduced by IV selection, we extracted SNPs located within ± 1000 kb of the top 10 IVs. We subsequently performed co-localization analysis using the coloc R package to assess whether each risk factor and lung cancer shared a common causal variant. Evidence of co-localization was considered strong if the posterior probability for a shared causal variant (PPH4) exceeded 0.70, or if PPH4 was > 0.50 and the ratio PPH4/(PPH3 + PPH4) was above 0.70.

### 2.8. Multi-trait co-localization analysis

To explore pleiotropic genetic effects across multiple traits, we applied HyPrColoc, a Bayesian-based R package designed for efficient detection of co-localization across numerous traits.^[17]^ This analysis included education, smoking, and other phenotypes to identify genomic regions and candidate causal genes jointly associated with these traits and lung cancer. For the multi-trait co-localization, each chromosome was divided into 20 segments of equal size. We then tested for regional co-localization among 8 traits – education, BMI, age at first sexual intercourse, age at first birth, smoking status, body fat percentage, adipose mass, and lung cancer subtypes – across these 20 regions per chromosome.

### 2.9. Statistical analysis

IVs were selected as SNPs satisfying the 3 key assumptions of MR: association with the exposure, independence from confounders, and influence on the outcome only through the exposure. SNPs were prioritized based on *F*-statistics and genome-wide significance. When rsID was unavailable, the MungeSumstats package was used to convert genomic coordinates to rsIDs. Missing effect allele frequency was tolerated without imputation. If one of beta, SE, or *P*-value was missing, it was derived from the remaining 2 using standard conversion formulas. Loci with 2 or more missing values among these statistics were excluded.

All analyses were performed in R version 4.3.3, ensuring computational reproducibility and reliability. This rigorous statistical framework strengthens the causal inferences drawn between exposures and lung cancer risk.

The R packages used in this analysis are detailed as follows:

Transform SNP position to rsid id (SNPlocs.Hsapiens.dbSNP144.GRCh38_0.99.20; vroom_1.6.5; SNPlocs.Hsapiens.dbSNP155.GRCh37_0.99.24)

Mendelian randomization analysis (ieugwasr_1.0.0; TwoSampleMR_0.6.7; dplyr_1.1.3; MendelianRandomization_0.9.0; mr.raps_0.4.1; MRAID_1.0)

extract outliers (RadialMR_1.0; reactable_0.4.4)

plots (ggrepel_0.9.3; rio_1.2.0; reshape_0.8.9; CMplot_4.5.1; webshot_0.5.5; tibble_3.2.1; networkD3_0.4)

co-localization (coloc_5.2.3; hyprcoloc_1.0; circlize_0.4.15)

## 3. Result

### 3.1. Mendelian randomization analysis

Following rigorous significance-level filtering and data harmonization, we evaluated over 23,000 datasets as potential exposure factors in our MR analysis of lung cancer causality. After applying the Benjamini-Hochberg correction for multiple testing, 90 datasets comprising 76 distinct traits were identified as causally associated with LUAD.

Notable protective factors included educational attainment and school-related attitudes, which were inversely associated with lung cancer risk. Similarly, longer paternal lifespan and later initiation of sexual activity were linked to a reduction in risk. Smoking behavior – including smoking status and frequency – was reaffirmed as a significant risk factor, consistent with established evidence. An unexpected inverse association was observed with light smoking; however, this result should be interpreted with caution due to reliance on only 1 IV.

**Figure 2.**
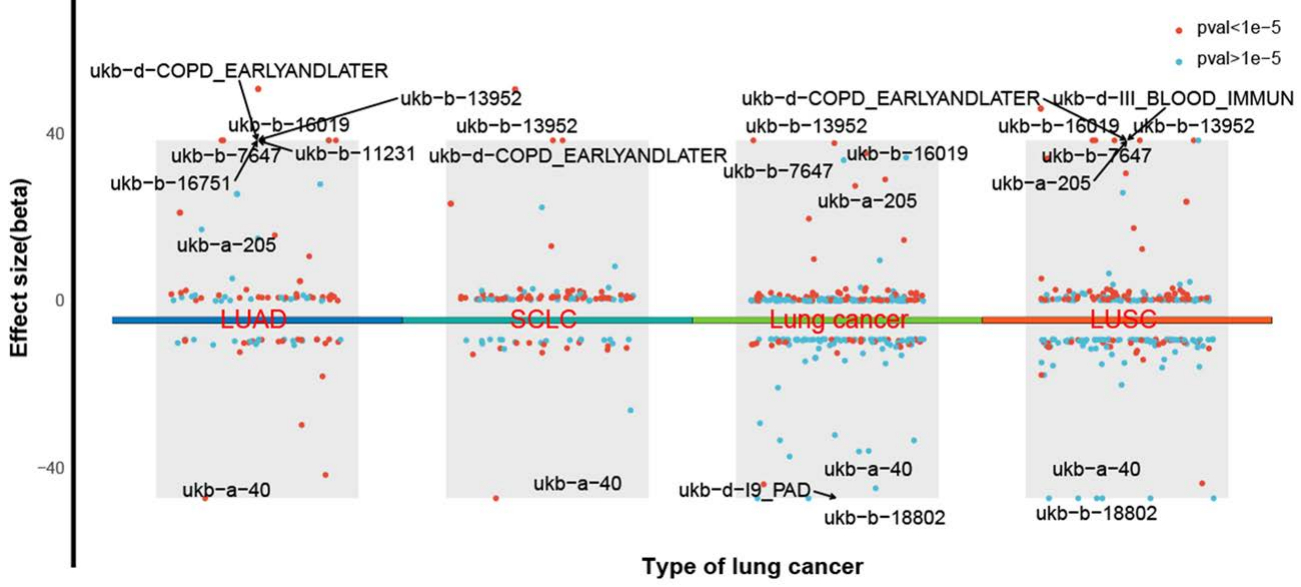
Volcano plot displaying effect sizes of exposure factors associated with lung cancer incidence.

Positive associations were identified between lung cancer risk and telomere length, as well as several respiratory conditions such as chronic obstructive pulmonary disease, pneumothorax, and other chronic obstructive lung diseases, implicating impaired lung health and accelerated aging in lung carcinogenesis. A comprehensive overview of these associations is provided in Figure 2.

In the analysis of SCLC, 116 datasets yielded 75 traits with causal implications. Protective associations were again observed for educational attainment, paternal longevity, and delayed sexual debut. Smoking and preexisting lung diseases were confirmed as risk factors. Additionally, elevated BMI and other adiposity-related traits were positively associated with SCLC risk. Parental history of lung cancer also appeared to contribute to inherited risk.

Further MR analyses incorporating 271 and 246 datasets identified 212 and 196 traits associated with overall lung cancer risk, respectively. Consistent with earlier findings, education, age at first birth, age at first sexual intercourse, paternal survival, and alcohol intake were inversely correlated with risk. Smoking, telomere length, adiposity, and pulmonary diseases were reaffirmed as risk factors. Of note, gastroesophageal reflux disease showed inconsistent effects across analyses, meriting further investigation, particularly in squamous cell carcinoma subtypes.

MR analysis showed 246 data sets with 196 traits causally associated with the development of lung squamous cell carcinoma (LUSC), and education, time to first sexual intercourse, drink, time to first birth, father’s survival status, and cardiovascular disease were considered to be inversely associated with the development of lung cancer. Smoking, adiposity index, some diseases occurring in the lungs and certain infectious and parasitic diseases can lead to the development of lung cancer. And the role of gastroesophageal reflux disease on squamous lung carcinogenesis still needs to be further explored.

### 3.2. Reliability testing of analytical results

We employed radial MR to identify and remove outliers among exposures that initially showed significant associations in MR analyses (Table S1, Supplemental Digital Content, https://links.lww.com/MD/Q526). Following outlier removal, all MR analyses were repeated.

Using the cML-MA method, the following previously suggested causal relationships were no longer supported: ENSG00000135698 with LUAD, Pack-years of smoking and SCLC, age at sexual debut, telomere length, and waist circumference with lung cancer, HbA1c, forced expiratory volume (FEV1), myocardial infarction, and uterine leiomyoma with LUSC.

Similarly, ConMix analysis did not support causal associations between: Breast cancer and LUAD, weight, hip circumference, overall health rating, or never smoking and lung cancer, telomere length or educational attainment and SCLC.

**Figure 3.**
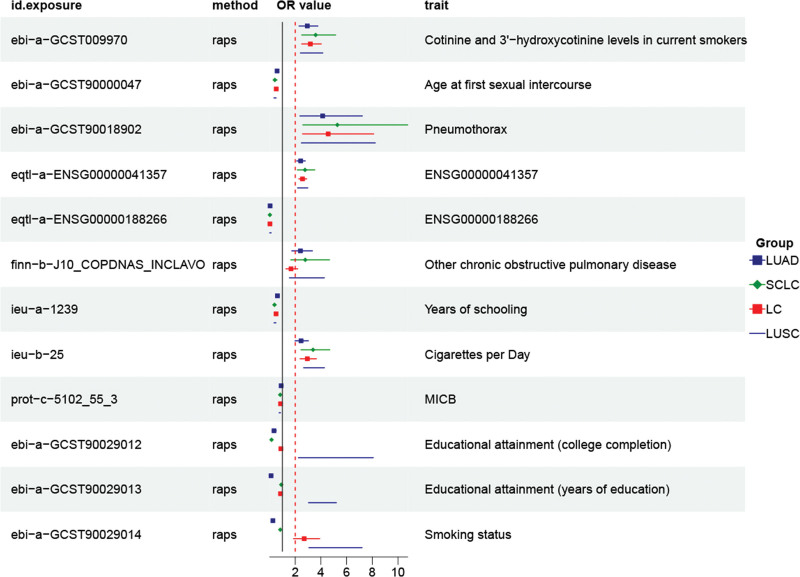
Forest plot illustrating the effects of selected exposures on lung cancer occurrence. LUAD = lung adenocarcinoma, LUSC = lung squamous cell carcinoma, SCLC = small cell lung cancer.

Familial combined hyperlipidemia (defined by consensus criteria), HbA1c, FEV1, or hip circumference and lung cancer, any exposure and squamous lung cancer. It should be noted that, consistent with its methodological design, ConMix results were considered unreliable and thus disregarded when the number of IVs was fewer than 50. All MR analyses were subsequently rerun using all 5 methods (IVW, cML-MA, ConMix, MR-RAPS, and dIVW). These comprehensive results are presented in Tables S2–S5, Supplemental Digital Content, https://links.lww.com/MD/Q526 and summarized visually in Figure 3.

### 3.3. Excluding the effect of horizontal pleiotropy on the results

To evaluate the robustness of our causal inferences, we assessed potential horizontal pleiotropy and heterogeneity across the IVs. Heterogeneity analysis revealed substantial heterogeneity (*I*^2^ > 0.5) for multiple traits, including smoking-related phenotypes (e.g., smoking frequency, nicotine metabolism rate, pack-years of smoking), dietary behaviors (such as consumption of dried fruits and avoidance of eggs or milk), and gastroesophageal reflux disease. Evidence of horizontal pleiotropy was also detected for several behavioral traits, including smoking frequency, as well as for education-related factors (such as educational attainment, qualification level, and certificate possession), adiposity-related traits, and telomere length. Complete results of these analyses are available in Tables S6 and S7, Supplemental Digital Content, https://links.lww.com/MD/Q526.

We further applied the MRAID method to evaluate the robustness of causal associations for the 90, 116, 271, and 246 traits linked to LUAD, SCLC, overall lung cancer, and LUSC, respectively. The results indicate that horizontal pleiotropy may underlie putative causal relationships for 35 traits associated with LUAD (including ENSG00000135698), 49 with SCLC (including adiposity), 90 with overall lung cancer (including adiposity and general health status), and 100 with LUSC (including body fat-related measures). Detailed MRAID outcomes are provided in Tables S8–S11, Supplemental Digital Content, https://links.lww.com/MD/Q526.

### 3.4. Meta-analysis of Mendelian randomization results

Using the meta-analyzed educational factor as exposure and factors such as smoking, BMI, age at first sexual intercourse, age at first birth, and fat-related metrics as outcomes, MR analysis demonstrated that educational attainment exhibits varying degrees of causal effects on these factors. The detailed id of dataset used for summarizing each trait is compiled in Table S12, Supplemental Digital Content, https://links.lww.com/MD/Q526. Specifically, higher education levels showed positive causal relationships with age at first birth, fat mass, and age at first sexual intercourse, while demonstrating negative causal relationships with BMI, fat percentage, and smoking behavior. However, these findings require further validation as the results failed to pass the intercept test of MR-Egger regression even after removing outliers through radial MR.

This 2-step MR analysis investigated the mediating role of several factors in the relationship between educational attainment and multiple lung cancer subtypes (LUAD, LUSC, SCLC, lung cancer). Significant indirect effects were observed for multiple mediators. For instance, age at first birth mediated a substantial proportion of the effect (Proportion Mediated = 0.538 for LUAD; β_indirect_ = 0.376, *P* < .0001), with a significant direct effect remaining (β_direct_ = −0.364, *P* < .0001). Similar significant mediation was found for BMI, fat percentage, fat mass, age at first sex, and smoking initiation across different outcome cohorts, with varying proportions mediated. The results consistently demonstrated that while these lifestyle and anthropometric factors significantly mediate the genetic effect of education on cognitive function, a significant direct effect of education persists, suggesting both mediated and direct pathways exist. Results from the 2-sample MR and MR mediation analyses are summarized in Tables S12 and S13, Supplemental Digital Content, https://links.lww.com/MD/Q526.

### 3.5. Co-localization analysis

For the co-localization analysis, we carefully selected the top 10 SNPs after harmonization. Our results demonstrate that lung carcinogenesis shares causal genetic variants with 179 datasets, corresponding to 118 distinct traits. These traits are primarily associated with smoking behavior, adiposity, educational attainment, pulmonary conditions, telomere length, alcohol use, and key life events – such as age at first sexual intercourse, age at first birth, and paternal survival. Notably, several traits exhibited co-localization with lung cancer across multiple genomic regions. For example, BMI co-localized with lung cancer on numerous chromosomes, including 1, 2, 3, 4, 5, 7, 8, 9, 10, 12, 14, 15, 17, 19, and 21. Comparable multi-locus co-localization patterns were detected for smoking- and education-related traits, underscoring the complex genetic architecture underlying lung cancer development.

To further investigate the interrelated roles of education, smoking, and other factors in lung cancer etiology, we conducted a multi-trait co-localization analysis. Adiposity-related measures were categorized into mass-based and percentage-based groups, which separated into 2 distinct clusters upon meta-analysis. Eight core traits were included: education, age at first birth, age at first sexual intercourse, BMI, smoking behavior, fat mass, fat percentage, and lung cancer itself.

**Figure 4.**
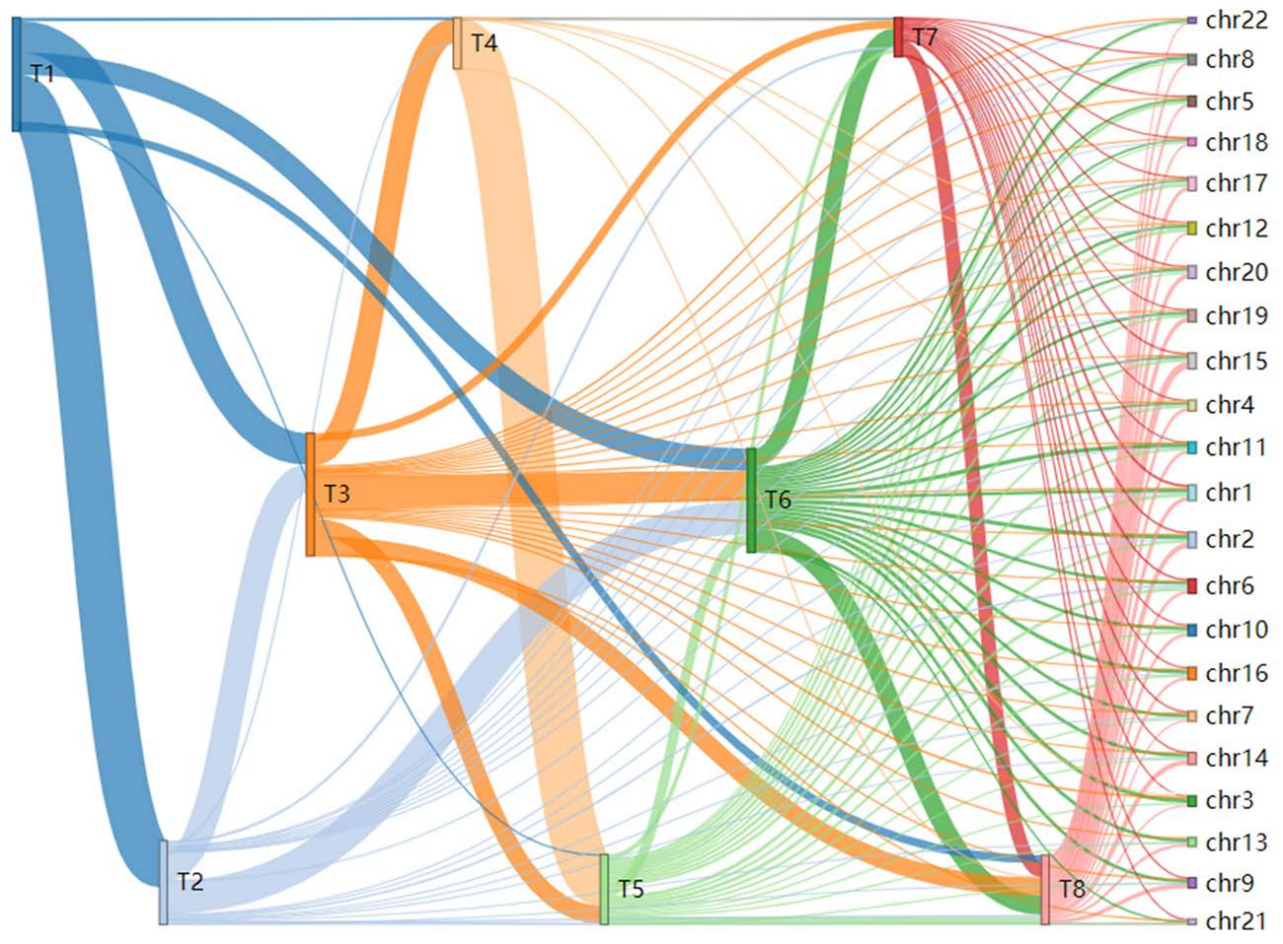
Results of co-localization analysis across multiple traits. T1: age at first birth, T2: BMI, T3: education, T4: fat mass, T5: fat percentage, T6: age at first sexual intercourse, T7: smoking, T8: lung cancer. Chromosomal regions (chr1–22) correspond to the 22 autosomal chromosomes. Connecting lines indicate evidence of co-localization between respective trait pairs. BMI = body mass index.

**Figure 5.**
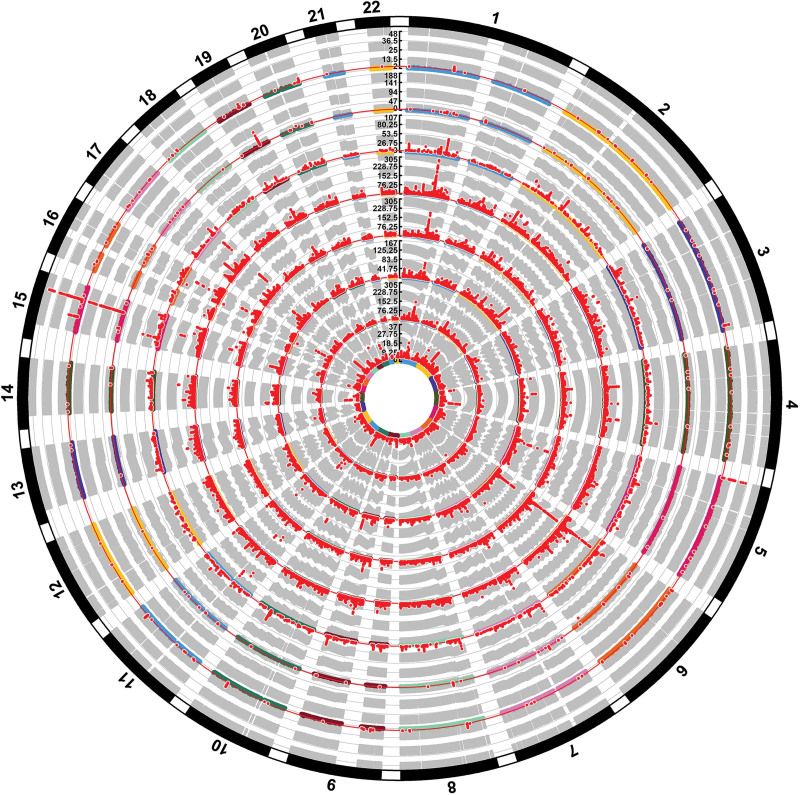
Circular Manhattan plot showing trait-associated genetic loci across the genome. Concentric circles represent the following traits from inner to outer rings: age at first birth, BMI, education, fat mass, fat percentage, age at first sexual intercourse, smoking, and lung adenocarcinoma. Chromosomes are arranged radially from 1 to 22. Each dot represents a single nucleotide polymorphism (SNP), with red dots denoting SNPs significantly associated with the corresponding trait. BMI = body mass index, SNP = single nucleotide polymorphism.

This multi-trait analysis revealed co-localization across 109 different combinations of these traits, illustrating widespread genetic pleiotropy, as depicted in Sankey and Manhattan plots. For instance, education and smoking traits showed shared genetic signals on chromosomes 1, 2, 3, 4, and 5. These results reflect the intricate regulatory networks and multi-pathway processes involved in lung cancer development. Complete co-localization results are available in Tables S14–S17, Supplemental Digital Content, https://links.lww.com/MD/Q526 and Figures 4 and 5.

## 4. Discussion

Lung cancer remains a major global health challenge, underscoring the urgent need to identify modifiable risk factors and develop effective prevention strategies. In this study, we utilized OpenGWAS databases to systematically screen and evaluate potential causal factors influencing lung cancer development.^[7–9]^ The primary goal was to identify key determinants that could serve as targets for primary prevention and early intervention. Our results underscore the multifactorial nature of lung cancer etiology and highlight the value of integrating diverse risk profiles into comprehensive cancer prevention frameworks.

In this work, through methods such as genome-wide significance screening, LD clumping, Cochran’s *Q* test, radial MR-based outlier removal, and restriction to individuals of European ancestry, we sought to mitigate the impact of type I error as much as possible. Meanwhile, the use of *F*-statistics, sample size control, and multiple causal analysis methods was employed to minimize the influence of type II error. The validity of IVs and the potential for horizontal pleiotropy are critical considerations in MR analyses. In addition to the standard IVW method, we employed several robust approaches – including cML-MA, ConMix, MR-RAPS, dIVW, and MRAID – to evaluate and adjust for pleiotropic effects.

Smoking was robustly identified as a major risk factor for lung cancer across multiple datasets and dimensions, including smoking frequency and nicotine metabolism. Co-localization analyses further indicated that smoking and lung cancer share several driver SNPs, reinforcing the well-established role of smoking in lung carcinogenesis.^[18–23]^ These findings suggest that smoking promotes lung cancer through diverse molecular mechanisms, such as impairing the production of oncogenic proteins (e.g., FANCD2, APOBEC),^[24,25]^ inhibiting CD8 + T-cell activity via IDO1 activation,^[26]^ promoting M2-type tumor-associated macrophage polarization,^[27]^ and inducing metabolic reprogramming through nicotine metabolites.^[28]^ These insights reflect the complexity of smoking-related lung oncogenesis.

We also identified a significant causal relationship between educational attainment – including attitudes toward education, age at completion, and number of qualifications – and lung cancer risk. These results align with previous observational studies indicating that higher education is associated with reduced lung cancer incidence and better prognosis.^[29,30]^ Potential mechanisms include reduced exposure to occupational carcinogens,^[31]^ adoption of healthier lifestyles,^[32]^ and better access to smoking cessation resources.^[33]^ Notably, lower educational attainment was correlated with greater difficulty in quitting smoking and higher weight gain, further implicating socioeconomic and behavioral pathways.

The role of adiposity in lung cancer development appears dualistic, reminiscent of the obesity paradox.^[34]^ On 1 hand, obesity promotes carcinogenesis through mechanisms such as elevated survivin expression – which inhibits apoptosis^[35]^ – and adiponectin-enhanced cancer stemness.^[36]^ High-fat diets may also alter tumor metabolism and immune surveillance.^[37]^ On the other hand, some studies report a protective effect of obesity, such as reduced mortality in LUAD,^[38]^ potentially mediated by leptin signaling inhibition via lipocalin and PTP1B activation.^[39]^ These conflicting findings suggest that the influence of adiposity may depend on context, including cancer subtype and metabolic microenvironment.^[40]^

Clinical measurements of inflammatory markers may not adequately reflect lung cancer risk. Contrary to some previous reports, our MR analyses did not support a broad causal role of systemic inflammation in lung cancer development. Although an association was observed between C-reactive protein and small cell lung cancer, this finding was not corroborated by sensitivity analyses using MRAID or co-localization testing. Furthermore, multiple causal inference methods failed to identify significant causal relationships between lung cancer and other inflammatory markers – including procalcitonin, serum amyloid A, IL-6, and TNF – after multiple testing correction. Previous studies focusing solely on C-reactive protein without comprehensive inclusion of inflammatory markers or rigorous *P*-value adjustment may have overestimated this association.^[41–43]^

This study utilized the OpenGWAS database to systematically evaluate traits with potential causal links to lung cancer, providing valuable insights for prevention and treatment. Nevertheless, several limitations should be acknowledged. The analysis included over 20,000 traits, preventing in-depth mechanistic exploration of each association. The pathways through which education influences lung cancer risk require further elucidation. Similarly, the dual role of adiposity and the molecular mechanisms underlying its protective effects – particularly related to body fat percentage – merit additional investigation. Finally, as our data were primarily drawn from European populations, the generalizability of findings to other ethnic groups remains uncertain.

## 5. Conclusion

MR and co-localization analyses indicate that educational attainment, BMI, age at first sexual intercourse, age at first childbirth, smoking, and adiposity are significant risk factors associated with lung cancer. Mediation analyses suggest that education may indirectly affect lung cancer risk by shaping health-related behaviors, such as sexual debut, reproductive timing, smoking, and weight management. These findings underscore the importance of integrating social, behavioral, and biological factors in public health strategies aimed at reducing the burden of lung cancer.

## Acknowledgments

We are grateful for the support and help from Departments of Anesthesiology and Basic Medicine at Changzhi Medical College. Thanks to the MCK team and the Finnish database, it is their selfless dedication of their data that gives us the privilege to make some contribution to lung cancer research. Thanks to the MRCIEU team for developing a convenient toolkit and database. It is the hard work of these people behind the scenes that made this research possible.

## Author contributions

**Conceptualization:** Pengcheng Feng.

**Data curation:** Yirong Wang, Pengcheng Feng.

**Formal analysis:** Pengcheng Feng.

**Funding acquisition:** Pengcheng Feng.

**Investigation:** Pengcheng Feng.

**Methodology:** Yirong Wang, Pengcheng Feng.

**Project administration:** Pengcheng Feng.

**Resources:** Pengcheng Feng.

**Software:** Xiaoqin Wang, Guodong Sun, Pengcheng Feng.

**Supervision:** Pengcheng Feng.

**Validation:** Pengcheng Feng.

**Visualization:** Xiaoqin Wang, Guodong Sun, Pengcheng Feng.

**Writing – original draft:** Yirong Wang, Xiaoqin Wang, Guodong Sun, Pengcheng Feng.

**Writing – review & editing:** Yirong Wang, Xiaoqin Wang, Guodong Sun, Pengcheng Feng.

## Supplementary Material


