# Supplementary material for: Education as a protective factor against lung cancer: A comprehensive Mendelian randomization analysis
Source: Medicine (Baltimore). 2025 Nov 7;104(45):e45651. doi: 10.1097/MD.0000000000045651 (PMC12599716; doi:10.1097/MD.0000000000045651)
Supplement: Supplementary file 1 [file medi-104-e45651-s001.pdf]

Supplementary Table 1 The detailed outliers SNP infromation generated by radial_MR

id	type	Estimate	Std.Error	SNP	Q_statistic	p.value
ebi-a-GCST004604	LUSC	-0.25	0.07	rs9273018	24.31	8.19E-07
ebi-a-GCST004604	Lung cancer	-0.17	0.04	rs9273018	23.23	1.44E-06
ebi-a-GCST004622	Lung cancer	-0.16	0.06	rs9270493	33.37	7.62E-09
ebi-a-GCST006250	Lung cancer	-0.27	0.06	rs11634187	11.97	5.40E-04
ebi-a-GCST007432	LUSC	-0.42	0.10	rs931794	155.00	1.40E-35
ebi-a-GCST007432	LUSC	-0.80	0.38	rs75499503	22.60	2.00E-06
ebi-a-GCST009971	LUSC	0.22	0.07	rs62117431	12.88	3.31E-04
ebi-a-GCST011365	LUSC	-0.18	0.05	rs7173743	14.07	1.77E-04
ebi-a-GCST90000045	LUSC	-0.85	0.13	rs12203592	12.33	4.47E-04
ebi-a-GCST90000047	LUAD	-0.55	0.11	rs34517439	25.96	3.48E-07
ebi-a-GCST90000047	LUAD	-0.55	0.11	rs12203592	16.98	3.78E-05
ebi-a-GCST90000047	LUSC	-0.84	0.11	rs838042	12.11	5.02E-04
ebi-a-GCST90000047	Lung cancer	-0.36	0.43	rs34517439	31.79	1.72E-08
ebi-a-GCST90000048	LUSC	-0.32	0.26	rs329122	12.49	4.10E-04
ebi-a-GCST90000048	Lung cancer	-0.16	0.03	rs329122	12.21	4.76E-04
ebi-a-GCST90000050	Lung cancer	-0.25	0.22	rs17391694	24.03	9.47E-07
ebi-a-GCST90000514	Lung cancer	0.65	0.52	rs7032155	14.06	1.77E-04
ebi-a-GCST90000514	LUSC	0.48	0.08	rs329122	12.06	5.16E-04
ebi-a-GCST90000514	Lung cancer	0.33	0.06	rs329122	11.88	5.66E-04
ebi-a-GCST90013870	Lung cancer	0.25	0.04	rs2066295	19.29	1.12E-05
ebi-a-GCST90013870	LUSC	0.37	0.06	rs329118	14.87	1.15E-04
ebi-a-GCST90013870	Lung cancer	0.14	0.14	rs329118	15.27	9.30E-05
ebi-a-GCST90013870	Lung cancer	0.14	0.14	rs6707445	12.07	5.12E-04
ebi-a-GCST90013870	Lung cancer	0.25	0.04	rs34517439	34.34	4.63E-09
ebi-a-GCST90013870	LUSC	0.54	0.23	rs2066295	25.91	3.57E-07
ebi-a-GCST90013870	LUSC	0.54	0.23	rs7132908	21.53	3.49E-06
ebi-a-GCST90013922	LUSC	2.02	0.43	rs2853677	17.88	2.35E-05
ebi-a-GCST90013922	LUSC	2.06	0.46	rs34684276	14.44	1.45E-04
ebi-a-GCST90013922	Lung cancer	1.94	0.37	rs28418426	21.96	2.79E-06

ebi-a-GCST90013972	LUSC	2.02	0.43	rs2853677	17.90	2.33E-05
ebi-a-GCST90013972	LUSC	2.07	0.46	rs34684276	13.98	1.85E-04
ebi-a-GCST90013974	LUSC	0.51	0.23	rs329118	14.85	1.16E-04
ebi-a-GCST90013974	LUSC	0.36	0.06	rs7132908	20.35	6.45E-06
ebi-a-GCST90013974	LUSC	0.36	0.06	rs2066295	25.92	3.55E-07
ebi-a-GCST90013974	Lung cancer	0.25	0.04	rs329118	15.20	9.66E-05
ebi-a-GCST90013974	Lung cancer	0.25	0.04	rs6707445	12.04	5.20E-04
ebi-a-GCST90013974	Lung cancer	0.12	0.14	rs34517439	35.17	3.02E-09
ebi-a-GCST90013974	Lung cancer	0.12	0.14	rs2066295	19.13	1.22E-05
ebi-a-GCST90013975	LUSC	0.10	0.38	rs10946808	25.28	4.95E-07
ebi-a-GCST90013975	Lung cancer	0.30	0.06	rs6707445	12.52	4.03E-04
ebi-a-GCST90013975	LUSC	0.44	0.09	rs112747606	13.97	1.86E-04
ebi-a-GCST90013975	Lung cancer	-0.15	0.23	rs10946808	33.49	7.15E-09
ebi-a-GCST90013975	LUSC	0.44	0.09	rs4884331	13.39	2.53E-04
ebi-a-GCST90013975	LUSC	0.10	0.38	rs12367809	20.84	4.99E-06
ebi-a-GCST90014020	Lung cancer	0.26	0.06	rs6711584	12.30	4.52E-04
ebi-a-GCST90014020	Lung cancer	0.26	0.06	rs2066295	18.21	1.98E-05
ebi-a-GCST90014020	LUSC	0.40	0.09	rs2066295	24.52	7.34E-07
ebi-a-GCST90014020	Lung cancer	0.11	0.20	rs1250600	15.39	8.73E-05
ebi-a-GCST90014020	LUSC	0.56	0.33	rs78378222	14.27	1.58E-04
ebi-a-GCST90014020	Lung cancer	0.11	0.20	rs34517439	35.93	2.05E-09
ebi-a-GCST90014020	LUSC	0.56	0.33	rs12367809	22.76	1.84E-06
ebi-a-GCST90014021	Lung cancer	0.00	0.15	rs927985	13.01	3.09E-04
ebi-a-GCST90014021	Lung cancer	0.00	0.15	rs28667353	13.98	1.85E-04
ebi-a-GCST90014021	Lung cancer	0.17	0.04	rs34517439	35.57	2.47E-09
ebi-a-GCST90018947	Lung cancer	0.28	0.04	rs34517439	33.92	5.76E-09
ebi-a-GCST90018947	Lung cancer	0.07	0.14	rs1624064	11.94	5.49E-04
ebi-a-GCST90018947	SCLC	0.61	0.10	rs73286281	13.57	2.30E-04
ebi-a-GCST90018947	Lung cancer	0.28	0.04	rs2066295	18.53	1.67E-05
ebi-a-GCST90018947	Lung cancer	0.07	0.14	rs329124	14.93	1.11E-04
ebi-a-GCST90018947	LUSC	0.39	0.07	rs13193685	13.14	2.89E-04

ebi-a-GCST90018947	LUSC	0.39	0.07	rs329124	15.13	1.00E-04
ebi-a-GCST90018947	Lung cancer	0.28	0.04	rs13193685	12.68	3.70E-04
ebi-a-GCST90018947	LUSC	0.41	0.24	rs2066295	25.30	4.91E-07
ebi-a-GCST90018947	LUSC	0.41	0.24	rs7132908	20.10	7.36E-06
ebi-a-GCST90018949	Lung cancer	0.17	0.13	rs10109521	12.86	3.36E-04
ebi-a-GCST90018949	LUSC	0.27	0.07	rs329118	15.88	6.75E-05
ebi-a-GCST90018949	LUSC	0.30	0.21	rs9379832	19.65	9.30E-06
ebi-a-GCST90018949	SCLC	0.55	0.29	rs7216064	14.45	1.44E-04
ebi-a-GCST90018949	LUSC	0.30	0.21	rs7132908	18.62	1.60E-05
ebi-a-GCST90018949	Lung cancer	0.20	0.04	rs9379832	21.53	3.48E-06
ebi-a-GCST90018949	Lung cancer	0.20	0.04	rs2277113	16.28	5.46E-05
ebi-a-GCST90018949	Lung cancer	0.17	0.13	rs2303262	12.99	3.13E-04
ebi-a-GCST90018949	LUSC	0.27	0.07	rs78378222	14.63	1.31E-04
ebi-a-GCST90018949	Lung cancer	0.17	0.13	rs34517439	34.78	3.69E-09
ebi-a-GCST90025972	Lung cancer	-0.16	0.05	rs61318425	18.07	2.12E-05
ebi-a-GCST90025994	LUSC	0.26	0.22	rs329118	15.32	9.07E-05
ebi-a-GCST90025994	LUSC	0.26	0.22	rs7132908	19.25	1.15E-05
ebi-a-GCST90025994	LUSC	0.31	0.07	rs4486004	24.80	6.36E-07
ebi-a-GCST90025994	Lung cancer	0.12	0.13	rs4486004	17.19	3.38E-05
ebi-a-GCST90025994	Lung cancer	0.22	0.04	rs329118	15.66	7.57E-05
ebi-a-GCST90025994	SCLC	0.50	0.09	rs8079754	13.50	2.39E-04
ebi-a-GCST90025994	Lung cancer	0.22	0.04	rs17391694	26.97	2.06E-07
ebi-a-GCST90025994	LUSC	0.31	0.07	rs112095287	13.21	2.79E-04
ebi-a-GCST90025994	Lung cancer	0.12	0.13	rs27640	12.91	3.27E-04
ebi-a-GCST90029007	LUSC	0.43	0.06	rs7132908	21.11	4.34E-06
ebi-a-GCST90029007	Lung cancer	0.28	0.04	rs2183947	17.99	2.23E-05
ebi-a-GCST90029007	Lung cancer	0.07	0.12	rs3822683	12.00	5.33E-04
ebi-a-GCST90029007	Lung cancer	0.07	0.12	rs329124	15.11	1.01E-04
ebi-a-GCST90029007	LUSC	0.43	0.06	rs2183947	22.70	1.89E-06
ebi-a-GCST90029007	LUSC	0.25	0.20	rs329124	15.31	9.13E-05
ebi-a-GCST90029007	Lung cancer	0.28	0.04	rs6707445	12.04	5.20E-04

ebi-a-GCST90029007	Lung cancer	0.28	0.04	rs34517439	33.47	7.23E-09
ebi-a-GCST90029012	Lung cancer	-1.38	0.16	rs4235642	14.52	1.39E-04
ebi-a-GCST90029012	Lung cancer	-1.35	0.85	rs2860049	19.79	8.63E-06
ebi-a-GCST90029012	Lung cancer	-1.35	0.85	rs806789	22.18	2.48E-06
ebi-a-GCST90029013	LUAD	-0.09	0.02	rs2706762	14.84	1.17E-04
ebi-a-GCST90029014	LUSC	0.79	1.17	rs329120	12.18	4.82E-04
ebi-a-GCST90029025	Lung cancer	0.14	0.13	rs1967315	12.90	3.28E-04
ebi-a-GCST90029025	Lung cancer	0.17	0.05	rs17608150	12.89	3.30E-04
ebi-a-GCST90029025	Lung cancer	0.14	0.13	rs34517439	36.58	1.47E-09
ebi-a-GCST90029025	Lung cancer	0.17	0.05	rs7186761	12.96	3.18E-04
ebi-a-GCST90029025	Lung cancer	0.17	0.05	rs329124	17.05	3.65E-05
ebi-a-GCST90104006	LUSC	-0.12	0.03	rs9378248	14.12	1.71E-04
eqtl-a-ENSG00000158406	Lung cancer	0.10	0.02	rs34661691	12.58	3.90E-04
eqtl-a-ENSG00000158406	Lung cancer	0.12	0.04	rs113282904	13.57	2.30E-04
finn-b-I9_REVASC	Lung cancer	-0.08	0.06	rs72749233	13.33	2.61E-04
ieu-a-1001	Lung cancer	-0.75	0.13	rs1378214	18.41	1.78E-05
ieu-a-1001	LUSC	0.06	1.15	rs12410444	12.92	3.25E-04
ieu-a-1239	LUAD	-0.43	0.40	rs2706762	14.67	1.28E-04
ieu-a-1239	Lung cancer	-0.87	0.31	rs6493265	18.82	1.44E-05
ieu-b-104	LUSC	-0.58	0.16	rs72738786	161.40	5.61E-37
ieu-b-104	LUSC	-0.79	0.70	rs6904596	34.04	5.40E-09
ieu-b-104	LUSC	-0.58	0.16	rs17843614	12.00	5.32E-04
ieu-b-142	Lung cancer	1.53	0.18	rs73229090	28.80	8.04E-08
ieu-b-142	LUAD	1.46	0.17	rs73229090	15.91	6.63E-05
ieu-b-142	LUSC	-0.96	0.55	rs806798	13.78	2.06E-04
ieu-b-142	LUSC	1.48	0.21	rs73229090	18.58	1.63E-05
ieu-b-142	Lung cancer	1.08	0.19	rs8034191	16.41	5.10E-05
ieu-b-142	Lung cancer	1.06	0.12	rs11725618	12.10	5.04E-04
ieu-b-142	Lung cancer	1.06	0.12	rs790564	15.78	7.13E-05
ieu-b-25	LUSC	-0.96	0.55	rs806798	13.78	2.06E-04
ieu-b-25	LUAD	0.94	0.12	rs73229090	27.04	1.99E-07

ieu-b-25	Lung cancer	1.08	0.19	rs8034191	16.41	5.10E-05
ieu-b-25	Lung cancer	1.06	0.12	rs790564	15.78	7.13E-05
ieu-b-25	LUSC	1.48	0.21	rs73229090	18.58	1.63E-05
ieu-b-25	Lung cancer	1.53	0.18	rs73229090	28.80	8.04E-08
ieu-b-25	Lung cancer	1.06	0.12	rs11725618	12.10	5.04E-04
ieu-b-40	Lung cancer	0.09	0.12	rs6707445	12.97	3.17E-04
ieu-b-40	LUSC	0.15	0.17	rs329122	16.00	6.35E-05
ieu-b-40	SCLC	0.44	0.28	rs12602912	13.55	2.32E-04
ieu-b-40	LUSC	0.33	0.06	rs7138803	20.02	7.68E-06
ieu-b-40	Lung cancer	0.09	0.12	rs17608150	12.43	4.23E-04
ieu-b-40	Lung cancer	0.18	0.04	rs17391694	27.78	1.36E-07
ieu-b-40	LUSC	0.33	0.06	rs17200912	13.65	2.20E-04
ieu-b-40	Lung cancer	0.18	0.04	rs329122	16.92	3.91E-05
ieu-b-40	LUSC	0.15	0.17	rs1150659	13.15	2.87E-04
ieu-b-4877	LUSC	0.95	0.56	rs134529	17.06	3.62E-05
ieu-b-4879	Lung cancer	0.51	0.09	rs76666449	12.27	4.60E-04
ieu-b-4879	LUAD	0.94	0.09	rs148297846	21.90	2.87E-06
ieu-b-4879	Lung cancer	0.85	0.13	rs79744444	12.96	3.18E-04
ieu-b-4879	LUAD	0.95	0.17	rs2056726	14.30	1.56E-04
ieu-b-4879	Lung cancer	0.50	0.07	rs112401627	32.07	1.49E-08
ieu-b-4879	LUAD	1.65	0.18	rs7705526	37.66	8.42E-10
ieu-b-4879	Lung cancer	0.50	0.07	rs7705526	34.37	4.57E-09
ieu-b-5113	LUSC	-0.58	0.16	rs72738786	161.40	5.61E-37
ieu-b-5113	LUSC	-0.79	0.70	rs6904596	34.04	5.40E-09
ieu-b-5113	LUSC	-0.58	0.16	rs17843614	12.00	5.32E-04
ieu-b-5117	LUSC	0.31	0.51	rs7132908	19.54	9.84E-06
ieu-b-5117	Lung cancer	0.37	0.09	rs34517439	34.41	4.47E-09
ieu-b-5118	LUSC	0.61	0.10	rs329118	14.92	1.12E-04
ieu-b-5118	Lung cancer	0.22	0.23	rs329118	15.69	7.46E-05
ieu-b-5118	Lung cancer	0.22	0.23	rs75499503	17.94	2.27E-05
ieu-b-5118	LUSC	0.61	0.39	rs7132908	20.56	5.78E-06

ieu-b-5118	SCLC	0.98	0.15	rs11000993	13.73	2.11E-04
ieu-b-5118	Lung cancer	0.34	0.07	rs34517439	34.98	3.33E-09
ieu-b-5118	LUSC	0.61	0.10	rs75499503	22.60	1.99E-06
ukb-a-176	LUSC	-4.76	7.73	rs10738607	12.97	3.16E-04
ukb-a-237	LUAD	1.89	0.39	rs8034191	18.81	1.44E-05
ukb-a-237	Lung cancer	2.07	0.40	rs8034191	31.32	2.18E-08
ukb-a-238	LUSC	2.10	0.34	rs4887067	12.13	4.97E-04
ukb-a-238	LUAD	1.72	0.36	rs4887067	26.00	3.41E-07
ukb-a-238	Lung cancer	1.97	0.60	rs4887067	37.46	9.33E-10
ukb-a-248	Lung cancer	0.23	0.05	rs329118	15.24	9.48E-05
ukb-a-248	Lung cancer	0.12	0.15	rs34517439	35.62	2.40E-09
ukb-a-248	LUSC	0.33	0.26	rs329118	14.77	1.22E-04
ukb-a-248	LUSC	0.35	0.07	rs7132908	19.92	8.09E-06
ukb-a-249	SCLC	0.42	0.10	rs68106312	15.35	8.92E-05
ukb-a-249	SCLC	0.01	0.34	rs58551145	13.82	2.01E-04
ukb-a-249	LUSC	0.10	0.23	rs329118	15.66	7.58E-05
ukb-a-249	Lung cancer	0.17	0.04	rs34517439	35.10	3.14E-09
ukb-a-249	Lung cancer	0.08	0.14	rs329118	16.24	5.59E-05
ukb-a-249	LUSC	0.27	0.06	rs78378222	14.64	1.30E-04
ukb-a-249	LUSC	0.10	0.23	rs7132908	17.99	2.22E-05
ukb-a-249	LUSC	0.14	0.19	rs34517439	12.35	4.40E-04
ukb-a-249	Lung cancer	0.17	0.04	rs2236084	15.28	9.26E-05
ukb-a-264	LUSC	0.49	0.11	rs1324088	22.82	1.78E-06
ukb-a-264	Lung cancer	0.30	0.07	rs1535274	17.18	3.39E-05
ukb-a-264	LUSC	-0.35	0.50	rs12367809	20.22	6.91E-06
ukb-a-264	Lung cancer	-0.26	0.30	rs9358912	31.35	2.16E-08
ukb-a-264	Lung cancer	-0.26	0.30	rs1324088	18.55	1.65E-05
ukb-a-264	LUSC	-0.35	0.50	rs9358912	23.87	1.03E-06
ukb-a-265	LUSC	0.03	0.31	rs329118	15.22	9.54E-05
ukb-a-265	LUSC	0.30	0.07	rs12367809	21.99	2.74E-06
ukb-a-265	Lung cancer	0.23	0.05	rs329118	15.73	7.31E-05

ukb-a-265	LUSC	0.25	0.27	rs34517439	12.40	4.29E-04
ukb-a-265	Lung cancer	-0.02	0.19	rs34517439	36.03	1.95E-09
ukb-a-272	Lung cancer	-0.25	0.06	rs34517439	37.60	8.70E-10
ukb-a-273	Lung cancer	-0.19	0.21	rs34517439	37.62	8.59E-10
ukb-a-274	Lung cancer	-0.79	0.41	rs1324087	17.07	3.60E-05
ukb-a-274	Lung cancer	0.46	0.09	rs12049202	15.02	1.06E-04
ukb-a-274	LUSC	0.57	0.13	rs1324087	22.76	1.84E-06
ukb-a-274	LUSC	-0.14	0.72	rs12367809	20.81	5.07E-06
ukb-a-275	Lung cancer	0.49	0.27	rs56913458	11.89	5.65E-04
ukb-a-275	Lung cancer	-0.08	0.23	rs34517439	35.71	2.28E-09
ukb-a-275	LUSC	0.44	0.28	rs34517439	12.35	4.40E-04
ukb-a-275	LUSC	-0.16	0.39	rs7132908	17.78	2.48E-05
ukb-a-275	LUSC	-0.16	0.39	rs329118	14.60	1.33E-04
ukb-a-275	Lung cancer	0.32	0.06	rs329118	15.54	8.10E-05
ukb-a-277	Lung cancer	0.06	0.18	rs34517439	36.87	1.26E-09
ukb-a-277	Lung cancer	0.20	0.06	rs9272550	22.87	1.73E-06
ukb-a-277	Lung cancer	0.06	0.18	rs1967315	12.31	4.52E-04
ukb-a-277	Lung cancer	0.20	0.06	rs2236084	15.57	7.93E-05
ukb-a-277	Lung cancer	0.20	0.06	rs7220	12.26	4.62E-04
ukb-a-277	Lung cancer	0.06	0.18	rs8126213	16.26	5.52E-05
ukb-a-278	LUSC	0.25	0.71	rs75499503	21.94	2.81E-06
ukb-a-278	Lung cancer	-0.60	0.39	rs75499503	18.04	2.16E-05
ukb-a-278	LUSC	0.60	0.13	rs12367809	21.91	2.86E-06
ukb-a-279	Lung cancer	0.33	0.06	rs329118	15.36	8.88E-05
ukb-a-279	LUSC	-0.03	0.38	rs34517439	12.20	4.79E-04
ukb-a-279	LUSC	0.44	0.09	rs7132908	19.88	8.26E-06
ukb-a-279	Lung cancer	-0.08	0.23	rs34517439	35.65	2.36E-09
ukb-a-279	LUSC	0.44	0.09	rs329118	15.19	9.70E-05
ukb-a-281	Lung cancer	0.20	0.06	rs116165844	16.73	4.31E-05
ukb-a-281	Lung cancer	0.09	0.18	rs1967315	12.36	4.38E-04
ukb-a-281	Lung cancer	0.20	0.06	rs7220	12.24	4.67E-04

ukb-a-281	Lung cancer	0.09	0.18	rs34517439	36.71	1.37E-09
ukb-a-281	Lung cancer	0.20	0.06	rs2236084	15.48	8.36E-05
ukb-a-282	LUSC	-0.36	0.48	rs75499503	23.08	1.55E-06
ukb-a-282	Lung cancer	0.34	0.07	rs75499503	16.66	4.47E-05
ukb-a-282	Lung cancer	-0.41	0.27	rs329118	14.05	1.78E-04
ukb-a-282	LUSC	0.42	0.11	rs329118	15.47	8.37E-05
ukb-a-282	LUSC	-0.36	0.48	rs12367809	19.62	9.46E-06
ukb-a-283	LUSC	0.27	0.07	rs34517439	11.99	5.35E-04
ukb-a-283	Lung cancer	-0.01	0.17	rs329118	15.25	9.42E-05
ukb-a-283	Lung cancer	0.22	0.05	rs34517439	34.55	4.15E-09
ukb-a-283	LUSC	-0.01	0.29	rs329118	15.35	8.92E-05
ukb-a-283	LUSC	0.27	0.07	rs12367809	21.71	3.18E-06
ukb-a-284	Lung cancer	0.22	0.19	rs116165844	16.67	4.44E-05
ukb-a-284	Lung cancer	0.22	0.19	rs34517439	35.94	2.04E-09
ukb-a-284	Lung cancer	0.21	0.06	rs7194734	13.35	2.59E-04
ukb-a-284	Lung cancer	0.21	0.06	rs1967315	12.45	4.19E-04
ukb-a-284	Lung cancer	0.21	0.06	rs35710322	12.22	4.72E-04
ukb-a-284	Lung cancer	0.22	0.19	rs521977	24.33	8.13E-07
ukb-a-286	LUSC	-0.33	0.48	rs329118	14.90	1.13E-04
ukb-a-286	Lung cancer	0.27	0.07	rs75499503	17.35	3.10E-05
ukb-a-286	Lung cancer	-0.22	0.27	rs329118	15.15	9.95E-05
ukb-a-286	LUSC	0.38	0.11	rs75499503	22.51	2.09E-06
ukb-a-287	LUSC	0.28	0.07	rs329118	15.50	8.26E-05
ukb-a-287	LUSC	0.29	0.08	rs7723426	12.01	5.30E-04
ukb-a-287	Lung cancer	-0.02	0.17	rs329118	15.50	8.25E-05
ukb-a-287	Lung cancer	0.20	0.05	rs34517439	34.98	3.34E-09
ukb-a-287	LUSC	-0.08	0.29	rs34517439	12.80	3.46E-04
ukb-a-287	LUSC	-0.08	0.29	rs12367809	19.96	7.91E-06
ukb-a-287	LUSC	0.28	0.07	rs56913458	13.63	2.23E-04
ukb-a-290	SCLC	-0.22	0.56	rs80135947	13.45	2.45E-04
ukb-a-291	SCLC	0.45	0.11	rs68106312	15.33	9.04E-05

ukb-a-291	LUSC	0.25	0.07	rs12367809	21.35	3.84E-06
ukb-a-291	LUSC	-0.25	0.30	rs34517439	13.44	2.47E-04
ukb-a-382	LUSC	0.62	0.38	rs2183947	21.33	3.87E-06
ukb-a-382	Lung cancer	0.14	0.21	rs2183947	17.59	2.73E-05
ukb-a-382	LUSC	0.32	0.09	rs12367809	21.30	3.94E-06
ukb-a-397	Lung cancer	-1.09	0.14	rs2860049	19.54	9.83E-06
ukb-a-397	LUSC	-1.42	0.19	rs9357004	24.27	8.39E-07
ukb-a-397	Lung cancer	-1.57	0.74	rs9357004	26.70	2.38E-07
ukb-a-5	Lung cancer	0.72	0.18	rs75499503	12.85	3.37E-04
ukb-b-10831	Lung cancer	2.69	0.68	rs2026174	13.34	2.59E-04
ukb-b-10831	LUAD	2.42	0.80	rs8042849	14.37	1.50E-04
ukb-b-10831	LUAD	2.42	0.80	rs2026174	15.71	7.38E-05
ukb-b-10831	Lung cancer	2.65	0.47	rs8042849	17.77	2.50E-05
ukb-b-11842	LUSC	0.14	0.14	rs34517439	13.07	3.01E-04
ukb-b-11842	LUSC	0.23	0.06	rs78378222	14.32	1.54E-04
ukb-b-11842	LUSC	0.03	0.20	rs329118	16.05	6.17E-05
ukb-b-11842	LUSC	0.03	0.20	rs7132908	17.16	3.43E-05
ukb-b-11842	SCLC	0.21	0.28	rs72833620	15.38	8.80E-05
ukb-b-12039	LUSC	0.11	0.20	rs7132908	17.69	2.59E-05
ukb-b-12039	LUSC	0.24	0.06	rs78378222	14.37	1.50E-04
ukb-b-12039	Lung cancer	0.17	0.04	rs6711584	13.18	2.83E-04
ukb-b-12039	Lung cancer	0.18	0.12	rs1624064	12.75	3.55E-04
ukb-b-12039	Lung cancer	0.18	0.12	rs4889464	11.97	5.40E-04
ukb-b-12039	Lung cancer	0.17	0.04	rs36035949	13.73	2.11E-04
ukb-b-12039	LUSC	0.09	0.14	rs34517439	12.54	3.98E-04
ukb-b-12039	LUSC	0.11	0.20	rs329118	16.00	6.35E-05
ukb-b-12039	Lung cancer	0.18	0.12	rs34517439	34.82	3.62E-09
ukb-b-12039	Lung cancer	0.17	0.04	rs329118	16.47	4.94E-05
ukb-b-12039	SCLC	0.37	0.09	rs72833620	15.32	9.05E-05
ukb-b-12854	LUSC	0.50	0.09	rs329118	15.26	9.35E-05
ukb-b-12854	Lung cancer	-0.22	0.22	rs75499503	17.92	2.30E-05

ukb-b-12854	Lung cancer	-0.22	0.22	rs329118	14.93	1.12E-04
ukb-b-12854	Lung cancer	0.56	0.20	rs3822683	12.39	4.32E-04
ukb-b-12854	LUSC	-0.16	0.36	rs12367809	20.07	7.48E-06
ukb-b-12854	LUSC	-0.16	0.36	rs75499503	23.00	1.62E-06
ukb-b-14540	Lung cancer	0.17	0.05	rs17608150	12.95	3.21E-04
ukb-b-14540	Lung cancer	0.20	0.14	rs6920372	16.05	6.18E-05
ukb-b-14540	Lung cancer	0.17	0.05	rs329124	17.06	3.63E-05
ukb-b-14540	Lung cancer	0.20	0.14	rs1624064	13.41	2.51E-04
ukb-b-14540	Lung cancer	0.17	0.05	rs521977	24.96	5.86E-07
ukb-b-14540	Lung cancer	0.20	0.14	rs34517439	35.90	2.08E-09
ukb-b-14540	Lung cancer	0.17	0.05	rs7202258	13.32	2.63E-04
ukb-b-14540	Lung cancer	0.20	0.14	rs1967315	12.82	3.42E-04
ukb-b-14540	Lung cancer	0.17	0.05	rs116165844	17.16	3.44E-05
ukb-b-15590	Lung cancer	0.14	0.14	rs329118	16.12	5.95E-05
ukb-b-15590	Lung cancer	0.17	0.04	rs34517439	35.47	2.59E-09
ukb-b-15590	LUSC	0.26	0.06	rs7132908	18.44	1.75E-05
ukb-b-15590	Lung cancer	0.14	0.14	rs12209691	13.76	2.08E-04
ukb-b-15590	Lung cancer	0.17	0.04	rs1624064	12.59	3.88E-04
ukb-b-15590	LUSC	0.26	0.06	rs329118	15.58	7.90E-05
ukb-b-15590	LUSC	0.11	0.22	rs34517439	12.48	4.11E-04
ukb-b-16407	Lung cancer	0.06	0.19	rs4936099	14.06	1.77E-04
ukb-b-16407	LUSC	0.15	0.29	rs9379833	25.22	5.12E-07
ukb-b-16407	Lung cancer	0.18	0.05	rs9379833	29.76	4.88E-08
ukb-b-16446	Lung cancer	0.17	0.05	rs1967315	12.91	3.27E-04
ukb-b-16446	Lung cancer	0.18	0.14	rs7202258	13.34	2.60E-04
ukb-b-16446	Lung cancer	0.17	0.05	rs1624064	13.30	2.66E-04
ukb-b-16446	Lung cancer	0.18	0.14	rs116165844	17.18	3.39E-05
ukb-b-16446	Lung cancer	0.18	0.14	rs17608150	12.93	3.24E-04
ukb-b-16446	Lung cancer	0.17	0.05	rs329124	16.97	3.80E-05
ukb-b-16446	Lung cancer	0.18	0.14	rs34517439	35.90	2.08E-09
ukb-b-16446	Lung cancer	0.17	0.05	rs521977	25.05	5.58E-07

ukb-b-16446	Lung cancer	0.18	0.14	rs2236084	15.06	1.04E-04
ukb-b-16489	Lung cancer	-1.30	0.13	rs10946808	23.60	1.18E-06
ukb-b-16489	Lung cancer	-1.53	0.71	rs329122	13.45	2.45E-04
ukb-b-16489	LUSC	-2.23	0.92	rs329122	13.91	1.92E-04
ukb-b-16489	Lung cancer	-1.30	0.13	rs4235642	14.21	1.64E-04
ukb-b-16489	Lung cancer	-1.53	0.71	rs6493265	19.75	8.82E-06
ukb-b-16489	LUSC	-1.69	0.18	rs10946808	19.70	9.06E-06
ukb-b-16698	Lung cancer	0.21	0.05	rs1967315	12.72	3.61E-04
ukb-b-16698	Lung cancer	0.21	0.05	rs116165844	16.97	3.81E-05
ukb-b-16698	Lung cancer	0.15	0.16	rs6920372	16.11	5.96E-05
ukb-b-16698	Lung cancer	0.21	0.05	rs34517439	35.79	2.20E-09
ukb-b-16698	Lung cancer	0.15	0.16	rs17608150	12.76	3.54E-04
ukb-b-16698	Lung cancer	0.15	0.16	rs329124	16.79	4.17E-05
ukb-b-16698	Lung cancer	0.21	0.05	rs7194734	13.46	2.44E-04
ukb-b-16878	Lung cancer	-0.17	3.56	rs806789	21.44	3.65E-06
ukb-b-17685	LUSC	7.62	2.08	rs576982	54.68	1.42E-13
ukb-b-18096	LUSC	0.27	0.32	rs329118	15.30	9.15E-05
ukb-b-18096	LUSC	0.43	0.08	rs56913458	13.90	1.93E-04
ukb-b-18096	Lung cancer	0.13	0.18	rs34517439	34.75	3.75E-09
ukb-b-18096	Lung cancer	0.23	0.18	rs3822683	12.06	5.15E-04
ukb-b-18096	Lung cancer	0.13	0.18	rs6707445	11.97	5.41E-04
ukb-b-18096	Lung cancer	0.35	0.05	rs329118	15.34	8.96E-05
ukb-b-18096	LUSC	0.27	0.32	rs7132908	19.17	1.20E-05
ukb-b-18377	Lung cancer	0.49	0.08	rs329118	15.57	7.97E-05
ukb-b-18377	Lung cancer	0.49	0.08	rs1749850	12.71	3.64E-04
ukb-b-18377	LUSC	0.74	0.12	rs329118	15.10	1.02E-04
ukb-b-18377	LUSC	0.64	0.55	rs7132908	20.05	7.53E-06
ukb-b-18377	Lung cancer	0.05	0.32	rs34517439	35.48	2.57E-09
ukb-b-18377	Lung cancer	0.05	0.32	rs75499503	17.05	3.65E-05
ukb-b-18377	Lung cancer	0.05	0.32	rs26491	12.73	3.61E-04
ukb-b-18377	LUSC	0.64	0.55	rs78884689	11.99	5.34E-04

ukb-b-18377	Lung cancer	0.49	0.08	rs62151809	12.23	4.71E-04
ukb-b-18377	LUSC	0.74	0.12	rs75499503	20.61	5.63E-06
ukb-b-19379	LUSC	-0.39	0.28	rs78378222	14.60	1.33E-04
ukb-b-19379	LUSC	-0.30	0.08	rs7531110	13.67	2.18E-04
ukb-b-19379	LUSC	-0.39	0.28	rs67177346	17.43	2.99E-05
ukb-b-19379	LUSC	-0.30	0.08	rs34517439	13.25	2.73E-04
ukb-b-19379	Lung cancer	-0.21	0.05	rs34517439	37.82	7.76E-10
ukb-b-19393	LUSC	0.34	0.06	rs329118	15.51	8.22E-05
ukb-b-19393	LUSC	0.34	0.06	rs7132908	19.81	8.53E-06
ukb-b-19393	Lung cancer	0.00	0.14	rs6711584	11.91	5.58E-04
ukb-b-19393	LUSC	0.09	0.23	rs34517439	12.19	4.81E-04
ukb-b-19393	Lung cancer	0.00	0.14	rs34517439	35.98	1.99E-09
ukb-b-19393	Lung cancer	0.33	0.17	rs3822683	12.02	5.27E-04
ukb-b-19393	Lung cancer	0.27	0.04	rs329118	15.61	7.80E-05
ukb-b-19393	Lung cancer	0.00	0.14	rs1624064	12.03	5.23E-04
ukb-b-19520	Lung cancer	0.08	0.16	rs55830103	11.92	5.55E-04
ukb-b-19520	Lung cancer	0.08	0.16	rs17608150	12.54	3.97E-04
ukb-b-19520	Lung cancer	0.22	0.05	rs116165844	16.94	3.85E-05
ukb-b-19520	Lung cancer	0.08	0.16	rs7194734	13.19	2.82E-04
ukb-b-19520	Lung cancer	0.22	0.05	rs34517439	35.61	2.41E-09
ukb-b-19520	Lung cancer	0.22	0.05	rs1967315	12.61	3.83E-04
ukb-b-19520	Lung cancer	0.22	0.05	rs6920372	16.25	5.55E-05
ukb-b-19520	Lung cancer	0.08	0.16	rs329124	16.61	4.60E-05
ukb-b-19921	LUSC	-0.25	0.07	rs67425403	16.20	5.71E-05
ukb-b-19921	LUSC	-0.25	0.07	rs7531110	13.80	2.03E-04
ukb-b-19921	LUSC	-0.25	0.07	rs56116178	12.10	5.04E-04
ukb-b-19921	LUSC	-0.25	0.07	rs34517439	13.07	3.01E-04
ukb-b-19921	Lung cancer	-0.21	0.14	rs34517439	37.49	9.19E-10
ukb-b-19921	LUSC	-0.46	0.24	rs7132908	17.64	2.67E-05
ukb-b-19921	LUSC	-0.46	0.24	rs329118	16.24	5.58E-05
ukb-b-19921	LUSC	-0.46	0.24	rs78378222	14.97	1.09E-04

ukb-b-19921	Lung cancer	-0.16	0.04	rs7823498	21.78	3.06E-06
ukb-b-19921	Lung cancer	-0.16	0.04	rs329118	16.74	4.29E-05
ukb-b-19925	Lung cancer	0.24	0.05	rs1967315	12.65	3.75E-04
ukb-b-19925	Lung cancer	0.20	0.15	rs2236084	15.49	8.28E-05
ukb-b-19925	Lung cancer	0.24	0.05	rs1624064	13.07	3.00E-04
ukb-b-19925	Lung cancer	0.24	0.05	rs521977	24.28	8.34E-07
ukb-b-19925	Lung cancer	0.20	0.15	rs34517439	35.41	2.68E-09
ukb-b-19925	Lung cancer	0.20	0.15	rs116165844	16.75	4.25E-05
ukb-b-19925	Lung cancer	0.24	0.05	rs329118	16.70	4.37E-05
ukb-b-19925	Lung cancer	0.20	0.15	rs7202258	13.03	3.07E-04
ukb-b-19925	Lung cancer	0.20	0.15	rs17608150	12.69	3.68E-04
ukb-b-19953	LUSC	0.40	0.22	rs75499503	22.25	2.39E-06
ukb-b-19953	Lung cancer	0.30	0.04	rs329118	14.62	1.31E-04
ukb-b-19953	Lung cancer	0.30	0.04	rs75499503	17.13	3.49E-05
ukb-b-19953	Lung cancer	0.11	0.13	rs34517439	34.62	4.01E-09
ukb-b-19953	LUSC	0.43	0.06	rs1165165	15.63	7.71E-05
ukb-b-19953	Lung cancer	0.30	0.04	rs1165165	11.92	5.56E-04
ukb-b-19953	LUSC	0.43	0.06	rs7132908	21.32	3.88E-06
ukb-b-19953	Lung cancer	0.25	0.16	rs3822683	12.11	5.03E-04
ukb-b-19953	Lung cancer	0.30	0.05	rs6707445	11.95	5.47E-04
ukb-b-19953	LUSC	0.40	0.22	rs329118	14.36	1.51E-04
ukb-b-19953	Lung cancer	0.11	0.13	rs17645692	15.10	1.02E-04
ukb-b-20044	Lung cancer	0.03	0.14	rs34517439	36.55	1.49E-09
ukb-b-20044	Lung cancer	0.20	0.04	rs6711584	12.78	3.50E-04
ukb-b-20044	SCLC	0.31	0.33	rs72833620	15.33	9.01E-05
ukb-b-20044	LUSC	-0.02	0.23	rs7132908	17.46	2.94E-05
ukb-b-20044	LUSC	0.25	0.18	rs34517439	12.82	3.43E-04
ukb-b-20188	Lung cancer	0.53	0.20	rs3822683	12.29	4.56E-04
ukb-b-20188	Lung cancer	-0.21	0.21	rs329124	15.17	9.82E-05
ukb-b-20188	Lung cancer	-0.21	0.21	rs75499503	18.12	2.07E-05
ukb-b-20188	LUSC	0.50	0.09	rs75499503	21.74	3.11E-06

ukb-b-20188	LUSC	0.50	0.09	rs12367809	23.17	1.49E-06
ukb-b-20188	LUSC	-0.21	0.36	rs329124	15.56	7.98E-05
ukb-b-20531	Lung cancer	0.43	0.08	rs26491	12.09	5.06E-04
ukb-b-20531	Lung cancer	0.11	0.33	rs34517439	35.93	2.04E-09
ukb-b-20531	LUSC	0.60	0.12	rs7132908	19.23	1.16E-05
ukb-b-20531	LUSC	1.16	0.55	rs75499503	20.39	6.33E-06
ukb-b-20531	LUSC	1.16	0.55	rs34517439	11.99	5.35E-04
ukb-b-20531	Lung cancer	0.43	0.08	rs62151809	12.50	4.06E-04
ukb-b-2134	Lung cancer	-0.65	0.70	rs597808	13.58	2.28E-04
ukb-b-2134	Lung cancer	-0.45	0.10	rs73229090	16.02	6.25E-05
ukb-b-2303	Lung cancer	0.23	0.16	rs3822683	12.07	5.12E-04
ukb-b-2303	LUSC	0.38	0.22	rs7132908	20.68	5.44E-06
ukb-b-2303	Lung cancer	0.11	0.13	rs34517439	34.68	3.89E-09
ukb-b-2303	LUSC	0.41	0.06	rs75499503	22.49	2.11E-06
ukb-b-2303	SCLC	0.68	0.08	rs11000993	13.89	1.94E-04
ukb-b-2303	LUSC	0.41	0.06	rs329118	14.49	1.41E-04
ukb-b-2303	Lung cancer	0.29	0.04	rs329118	14.66	1.29E-04
ukb-b-2303	Lung cancer	0.11	0.13	rs17645692	15.01	1.07E-04
ukb-b-5192	Lung cancer	0.78	0.13	rs75499503	12.41	4.27E-04
ukb-b-5192	LUSC	1.10	0.20	rs75499503	17.32	3.15E-05
ukb-b-6019	LUAD	2.56	0.36	rs10105127	25.35	4.78E-07
ukb-b-6019	Lung cancer	2.77	0.38	rs10105127	32.62	1.12E-08
ukb-b-6306	Lung cancer	0.74	0.15	rs6770476	12.67	3.71E-04
ukb-b-6306	LUSC	1.24	1.52	rs10819083	14.02	1.81E-04
ukb-b-6306	Lung cancer	0.80	1.08	rs11073964	13.72	2.13E-04
ukb-b-6591	LUAD	-0.57	0.10	rs10204104	15.03	1.06E-04
ukb-b-6591	Lung cancer	-0.38	0.44	rs140098	13.06	3.02E-04
ukb-b-6591	Lung cancer	-0.36	0.53	rs30266	12.13	4.96E-04
ukb-b-6591	LUAD	0.03	0.54	rs34517439	24.82	6.29E-07
ukb-b-6591	Lung cancer	-0.38	0.44	rs34517439	31.29	2.22E-08
ukb-b-6704	LUSC	0.35	0.06	rs7132908	20.10	7.35E-06

ukb-b-6704	Lung cancer	0.28	0.04	rs1624064	12.00	5.33E-04
ukb-b-6704	LUSC	0.35	0.06	rs329118	15.21	9.63E-05
ukb-b-6704	LUSC	0.05	0.23	rs34517439	12.26	4.63E-04
ukb-b-6704	Lung cancer	0.28	0.04	rs6707445	12.35	4.40E-04
ukb-b-6704	Lung cancer	-0.02	0.14	rs329118	15.10	1.02E-04
ukb-b-6704	Lung cancer	-0.02	0.14	rs3822683	12.18	4.83E-04
ukb-b-6704	Lung cancer	0.28	0.04	rs34517439	32.66	1.10E-08
ukb-b-7212	Lung cancer	0.36	0.05	rs6711584	12.03	5.22E-04
ukb-b-7212	Lung cancer	0.10	0.18	rs329118	15.09	1.03E-04
ukb-b-7212	Lung cancer	0.10	0.18	rs3822683	12.12	4.99E-04
ukb-b-7212	SCLC	0.60	0.40	rs10824163	14.25	1.60E-04
ukb-b-7212	LUSC	0.23	0.30	rs329118	15.13	1.01E-04
ukb-b-7212	LUSC	0.47	0.08	rs7132908	20.33	6.51E-06
ukb-b-7212	Lung cancer	0.36	0.05	rs34517439	32.84	1.00E-08
ukb-b-7460	Lung cancer	2.46	0.59	rs8042849	21.19	4.16E-06
ukb-b-7460	LUAD	2.14	0.45	rs2026174	14.10	1.73E-04
ukb-b-7460	LUAD	2.14	0.45	rs8042849	17.35	3.10E-05
ukb-b-7859	LUSC	-0.29	0.08	rs78378222	14.47	1.43E-04
ukb-b-7859	LUSC	-0.37	0.29	rs34517439	13.28	2.68E-04
ukb-b-7859	LUSC	-0.29	0.08	rs56116178	12.15	4.92E-04
ukb-b-7859	LUSC	-0.37	0.29	rs67425403	16.39	5.15E-05
ukb-b-7859	LUSC	-0.29	0.08	rs7132908	16.98	3.78E-05
ukb-b-7859	LUSC	-0.37	0.29	rs7531110	13.64	2.21E-04
ukb-b-8338	LUSC	0.36	0.06	rs7132908	20.31	6.59E-06
ukb-b-8338	Lung cancer	0.28	0.04	rs329118	15.10	1.02E-04
ukb-b-8338	Lung cancer	0.01	0.14	rs34517439	35.62	2.40E-09
ukb-b-8338	Lung cancer	0.36	0.17	rs3822683	12.20	4.78E-04
ukb-b-8338	LUSC	0.23	0.17	rs34517439	12.10	5.04E-04
ukb-b-8338	LUSC	0.07	0.22	rs329118	15.08	1.03E-04
ukb-b-8909	LUSC	0.01	0.37	rs1324088	22.68	1.91E-06
ukb-b-8909	LUSC	0.46	0.09	rs9379833	23.46	1.28E-06

ukb-b-8909	Lung cancer	0.47	0.21	rs469735	12.20	4.79E-04
ukb-b-8909	LUSC	0.46	0.09	rs329118	15.69	7.47E-05
ukb-b-8909	Lung cancer	-0.17	0.23	rs9379833	33.11	8.72E-09
ukb-b-8909	LUSC	0.01	0.37	rs7132908	17.93	2.29E-05
ukb-b-8909	Lung cancer	-0.17	0.23	rs329118	15.17	9.82E-05
ukb-b-9093	Lung cancer	0.19	0.16	rs34517439	35.97	2.00E-09
ukb-b-9093	Lung cancer	0.20	0.05	rs329118	16.91	3.92E-05
ukb-b-9093	Lung cancer	0.20	0.05	rs55830103	12.22	4.73E-04
ukb-b-9093	Lung cancer	0.20	0.05	rs116165844	17.01	3.72E-05
ukb-b-9093	Lung cancer	0.19	0.16	rs7194734	13.51	2.37E-04
ukb-b-9093	Lung cancer	0.19	0.16	rs1967315	12.81	3.44E-04
ukb-b-9093	Lung cancer	0.20	0.05	rs9480958	17.25	3.27E-05
ukb-b-9093	Lung cancer	0.19	0.16	rs1624064	13.26	2.72E-04
ukb-b-9093	Lung cancer	0.20	0.05	rs17608150	12.91	3.26E-04
ukb-b-9405	Lung cancer	0.15	0.19	rs2183947	17.36	3.09E-05
ukb-b-9405	LUSC	0.60	0.31	rs2183947	21.40	3.72E-06
ukb-b-9405	LUSC	0.60	0.31	rs78378222	14.01	1.82E-04
ukb-b-9405	Lung cancer	0.15	0.19	rs34517439	35.52	2.53E-09
ukb-b-9405	Lung cancer	0.28	0.06	rs6711584	12.51	4.04E-04
ukb-b-9405	Lung cancer	0.28	0.06	rs329118	15.94	6.54E-05
ukb-b-9405	LUSC	0.37	0.09	rs34517439	12.03	5.25E-04
ukb-b-9405	LUSC	0.36	0.08	rs7132908	18.92	1.36E-05
ukb-b-9405	LUSC	0.36	0.08	rs329118	15.75	7.24E-05
ukb-b-969	Lung cancer	6.27	2.35	rs34517439	15.70	7.40E-05
ukb-d-20116_0	Lung cancer	-1.11	0.70	rs12910916	12.00	5.32E-04
ukb-d-30020_irnt	Lung cancer	-0.22	0.10	rs9273047	24.51	7.38E-07

Supplementary Table 2 The MR analysis results of between exposures and LUAD

id.exposure	method	beta	se	pval	sample_size	or
ebi-a-GCST004441	cML-MA	-0.17	0.05	1.56E-04	3636	8.42E-01
ebi-a-GCST004441	raps	-0.17	0.05	2.72E-04	3636	8.41E-01
ebi-a-GCST004441	IVW	-0.17	0.04	9.01E-05	3636	8.43E-01
ebi-a-GCST004441	conmix	-0.17	0.07	1.07E-02	3636	8.41E-01
ebi-a-GCST004441	divw	-0.17	0.05	1.42E-04	3636	8.41E-01
ebi-a-GCST009965	Wald ratio	1.08	0.08	4.14E-47	5185	2.95E+00
ebi-a-GCST009965	raps	1.08	0.13	4.01E-17	5185	2.95E+00
ebi-a-GCST009966	raps	-0.74	0.16	4.64E-06	4772	4.78E-01
ebi-a-GCST009966	Wald ratio	-0.74	0.12	8.64E-10	4772	4.78E-01
ebi-a-GCST009968	raps	1.71	0.28	1.44E-09	4772	5.51E+00
ebi-a-GCST009968	Wald ratio	1.71	0.12	1.74E-47	4772	5.51E+00
ebi-a-GCST009970	raps	1.08	0.13	1.42E-17	5185	2.94E+00
ebi-a-GCST009970	Wald ratio	1.08	0.07	4.14E-47	5185	2.94E+00
ebi-a-GCST009971	cML-MA	0.15	0.03	8.78E-06	5185	1.16E+00
ebi-a-GCST009971	IVW	0.15	0.03	1.46E-08	5185	1.17E+00
ebi-a-GCST009971	divw	0.16	0.03	4.63E-07	5185	1.17E+00
ebi-a-GCST009971	raps	0.16	0.03	1.00E-09	5185	1.17E+00
ebi-a-GCST009971	conmix	0.16	0.06	3.63E-03	5185	1.18E+00
ebi-a-GCST90000047	divw	-0.55	0.10	5.35E-08	397338	5.74E-01
ebi-a-GCST90000047	conmix	-0.78	0.20	7.31E-05	397338	4.60E-01
ebi-a-GCST90000047	IVW	-0.54	0.07	1.27E-14	397338	5.82E-01
ebi-a-GCST90000047	raps	-0.54	0.10	2.73E-07	397338	5.85E-01
ebi-a-GCST90000047	cML-MA	-0.51	0.13	8.37E-05	397338	6.00E-01
ebi-a-GCST90006885	raps	0.82	0.23	4.66E-04	8555	2.27E+00
ebi-a-GCST90006885	Wald ratio	0.82	0.18	5.69E-06	8555	2.27E+00

ebi-a-GCST90006898	raps	-0.97	0.26	2.15E-04	7763	3.80E-01
ebi-a-GCST90006898	Wald ratio	-0.97	0.18	1.42E-07	7763	3.80E-01
ebi-a-GCST90013883	raps	-0.19	0.04	1.37E-06	407746	8.23E-01
ebi-a-GCST90013883	Wald ratio	-0.19	0.03	1.22E-09	407746	8.23E-01
ebi-a-GCST90013933	Wald ratio	-0.17	0.03	1.22E-09	407746	8.44E-01
ebi-a-GCST90013933	raps	-0.17	0.04	2.63E-06	407746	8.44E-01
ebi-a-GCST90016676	Wald ratio	-1.41	0.34	3.82E-05	25617	2.43E-01
ebi-a-GCST90016676	raps	-1.41	0.44	1.29E-03	25617	2.43E-01
ebi-a-GCST90018902	Wald ratio	1.42	0.10	9.28E-46	477734	4.14E+00
ebi-a-GCST90018902	raps	1.42	0.28	6.13E-07	477734	4.14E+00
ebi-a-GCST90029012	conmix	-1.27	0.34	1.73E-04	470941	2.81E-01
ebi-a-GCST90029012	IVW	-1.05	0.20	2.32E-07	470941	3.49E-01
ebi-a-GCST90029012	raps	-1.06	0.21	4.46E-07	470941	3.46E-01
ebi-a-GCST90029012	cML-MA	-1.10	0.27	4.04E-05	470941	3.34E-01
ebi-a-GCST90029012	divw	-1.08	0.21	2.50E-07	470941	3.41E-01
ebi-a-GCST90029013	cML-MA	-0.09	0.02	7.26E-05	461457	9.13E-01
ebi-a-GCST90029013	IVW	-0.10	0.02	1.51E-08	461457	9.07E-01
ebi-a-GCST90029013	conmix	-0.10	0.03	1.20E-03	461457	9.08E-01
ebi-a-GCST90029013	raps	-0.09	0.02	3.55E-07	461457	9.11E-01
ebi-a-GCST90029013	divw	-0.10	0.02	1.47E-08	461457	9.05E-01
ebi-a-GCST90029014	raps	0.99	0.19	1.26E-07	468170	2.70E+00
ebi-a-GCST90029014	cML-MA	0.98	0.25	6.76E-05	468170	2.67E+00
ebi-a-GCST90029014	conmix	1.63	0.32	4.27E-07	468170	5.10E+00
ebi-a-GCST90029014	IVW	0.95	0.18	7.23E-08	468170	2.59E+00
ebi-a-GCST90029014	divw	0.98	0.18	6.77E-08	468170	2.66E+00
ebi-a-GCST90101889	raps	0.85	0.18	1.28E-06	902	2.34E+00

ebi-a-GCST90101889	Wald ratio	0.85	0.09	5.34E-21	902	2.34E+00
eqtl-a-ENSG00000026036	IVW	-0.84	0.17	7.06E-07	30213	4.32E-01
eqtl-a-ENSG00000026036	raps	-0.85	0.18	1.84E-06	30213	4.28E-01
eqtl-a-ENSG00000041357	divw	0.89	0.07	4.03E-38	31644	2.43E+00
eqtl-a-ENSG00000041357	IVW	0.88	0.06	1.14E-53	31644	2.42E+00
eqtl-a-ENSG00000041357	raps	0.88	0.07	1.49E-36	31644	2.42E+00
eqtl-a-ENSG00000066084	IVW	-0.16	0.04	1.65E-04	31470	8.49E-01
eqtl-a-ENSG00000066084	raps	-0.16	0.04	2.62E-04	31470	8.49E-01
eqtl-a-ENSG00000066084	conmix	-0.17	0.07	1.17E-02	31470	8.39E-01
eqtl-a-ENSG00000066084	cML-MA	-0.16	0.04	8.95E-05	31470	8.48E-01
eqtl-a-ENSG00000066084	divw	-0.16	0.04	1.80E-04	31470	8.49E-01
eqtl-a-ENSG00000071054	Wald ratio	0.91	0.22	3.72E-05	26395	2.49E+00
eqtl-a-ENSG00000071054	raps	0.91	0.27	8.82E-04	26395	2.49E+00
eqtl-a-ENSG00000074527	raps	0.86	0.25	5.73E-04	31470	2.36E+00
eqtl-a-ENSG00000074527	Wald ratio	0.86	0.21	3.15E-05	31470	2.36E+00
eqtl-a-ENSG00000099821	Wald ratio	0.52	0.14	1.24E-04	14263	1.68E+00
eqtl-a-ENSG00000099821	raps	0.52	0.15	3.56E-04	14263	1.68E+00
eqtl-a-ENSG00000100462	divw	-0.11	0.03	1.59E-04	30551	8.92E-01
eqtl-a-ENSG00000100462	conmix	-0.11	0.04	4.74E-03	30551	8.98E-01
eqtl-a-ENSG00000100462	cML-MA	-0.11	0.03	2.29E-04	30551	8.93E-01
eqtl-a-ENSG00000100462	raps	-0.11	0.03	3.07E-04	30551	8.94E-01
eqtl-a-ENSG00000100462	IVW	-0.11	0.03	1.51E-04	30551	8.92E-01
eqtl-a-ENSG00000104047	Wald ratio	-1.50	0.24	8.07E-10	14263	2.24E-01
eqtl-a-ENSG00000104047	raps	-1.50	0.36	3.35E-05	14263	2.24E-01
eqtl-a-ENSG00000118680	raps	-0.45	0.12	1.87E-04	28489	6.38E-01
eqtl-a-ENSG00000118680	Wald ratio	-0.45	0.11	5.72E-05	28489	6.38E-01

eqtl-a-ENSG00000130755	Wald ratio	0.49	0.11	7.99E-06	9188	1.63E+00
eqtl-a-ENSG00000130755	raps	0.49	0.12	4.35E-05	9188	1.63E+00
eqtl-a-ENSG00000135698	cML-MA	0.08	0.04	5.85E-02	31470	1.08E+00
eqtl-a-ENSG00000135698	raps	0.09	0.03	5.03E-04	31470	1.10E+00
eqtl-a-ENSG00000135698	conmix	0.11	0.04	8.79E-03	31470	1.12E+00
eqtl-a-ENSG00000135698	IVW	0.09	0.02	3.48E-05	31470	1.10E+00
eqtl-a-ENSG00000135698	divw	0.09	0.02	2.96E-05	31470	1.10E+00
eqtl-a-ENSG00000137265	Wald ratio	0.44	0.11	1.17E-04	31684	1.56E+00
eqtl-a-ENSG00000137265	raps	0.44	0.12	3.79E-04	31684	1.56E+00
eqtl-a-ENSG00000138593	IVW	-0.62	0.14	6.07E-06	31684	5.37E-01
eqtl-a-ENSG00000138593	raps	-0.63	0.13	8.74E-07	31684	5.34E-01
eqtl-a-ENSG00000142233	divw	0.21	0.05	4.99E-06	31684	1.24E+00
eqtl-a-ENSG00000142233	conmix	0.22	0.09	1.74E-02	31684	1.25E+00
eqtl-a-ENSG00000142233	IVW	0.21	0.05	3.87E-06	31684	1.23E+00
eqtl-a-ENSG00000142233	cML-MA	0.21	0.05	2.89E-05	31684	1.23E+00
eqtl-a-ENSG00000142233	raps	0.21	0.05	8.50E-06	31684	1.24E+00
eqtl-a-ENSG00000145414	Wald ratio	-0.63	0.13	1.20E-06	31684	5.33E-01
eqtl-a-ENSG00000145414	raps	-0.63	0.15	1.88E-05	31684	5.33E-01
eqtl-a-ENSG00000149573	divw	-0.10	0.02	2.75E-05	31684	9.09E-01
eqtl-a-ENSG00000149573	raps	-0.11	0.02	7.51E-06	31684	9.00E-01
eqtl-a-ENSG00000149573	IVW	-0.10	0.02	8.00E-06	31684	9.09E-01
eqtl-a-ENSG00000149573	cML-MA	-0.09	0.03	8.28E-03	31684	9.18E-01
eqtl-a-ENSG00000149573	conmix	-0.13	0.03	1.30E-04	31684	8.81E-01
eqtl-a-ENSG00000156414	cML-MA	-0.09	0.03	1.33E-03	31644	9.17E-01
eqtl-a-ENSG00000156414	raps	-0.09	0.03	3.86E-04	31644	9.12E-01
eqtl-a-ENSG00000156414	conmix	-0.09	0.03	1.60E-03	31644	9.17E-01
eqtl-a-ENSG00000156414	divw	-0.09	0.02	1.18E-04	31644	9.14E-01
eqtl-a-ENSG00000156414	IVW	-0.09	0.02	1.41E-04	31644	9.14E-01

eqtl-a-ENSG00000156958	IVW	-0.26	0.06	3.05E-06	31684	7.71E-01
eqtl-a-ENSG00000156958	raps	-0.26	0.06	8.06E-06	31684	7.71E-01
eqtl-a-ENSG00000160588	divw	-0.26	0.06	9.37E-06	31684	7.72E-01
eqtl-a-ENSG00000160588	raps	-0.26	0.04	1.50E-09	31684	7.69E-01
eqtl-a-ENSG00000160588	IVW	-0.26	0.06	2.25E-05	31684	7.72E-01
eqtl-a-ENSG00000177406	IVW	0.11	0.03	6.52E-05	17261	1.11E+00
eqtl-a-ENSG00000177406	cML-MA	0.11	0.03	5.27E-05	17261	1.11E+00
eqtl-a-ENSG00000177406	raps	0.11	0.03	1.04E-04	17261	1.11E+00
eqtl-a-ENSG00000177406	divw	0.11	0.03	6.83E-05	17261	1.11E+00
eqtl-a-ENSG00000177406	conmix	0.11	0.04	1.92E-03	17261	1.12E+00
eqtl-a-ENSG00000188199	cML-MA	-0.15	0.05	8.36E-04	5502	8.57E-01
eqtl-a-ENSG00000188199	IVW	-0.16	0.04	1.17E-04	5502	8.52E-01
eqtl-a-ENSG00000188199	raps	-0.16	0.04	1.51E-04	5502	8.52E-01
eqtl-a-ENSG00000188199	conmix	-0.18	0.07	1.14E-02	5502	8.39E-01
eqtl-a-ENSG00000188199	divw	-0.16	0.04	9.07E-05	5502	8.51E-01
eqtl-a-ENSG00000188266	Wald ratio	-3.15	0.22	9.28E-46	26395	4.27E-02
eqtl-a-ENSG00000188266	raps	-3.15	0.59	8.97E-08	26395	4.27E-02
eqtl-a-ENSG00000204789	conmix	0.20	0.07	5.45E-03	26395	1.22E+00
eqtl-a-ENSG00000204789	cML-MA	0.19	0.05	1.18E-04	26395	1.21E+00
eqtl-a-ENSG00000204789	IVW	0.19	0.05	6.44E-05	26395	1.21E+00
eqtl-a-ENSG00000204789	divw	0.19	0.05	7.57E-05	26395	1.21E+00
eqtl-a-ENSG00000204789	raps	0.19	0.05	1.15E-04	26395	1.21E+00
eqtl-a-ENSG00000211677	conmix	0.42	0.14	4.04E-03	5502	1.51E+00
eqtl-a-ENSG00000211677	IVW	0.41	0.11	1.01E-04	5502	1.51E+00
eqtl-a-ENSG00000211677	cML-MA	0.42	0.12	2.86E-04	5502	1.53E+00
eqtl-a-ENSG00000211677	divw	0.42	0.11	1.96E-04	5502	1.53E+00
eqtl-a-ENSG00000211677	raps	0.42	0.11	2.42E-04	5502	1.52E+00
eqtl-a-ENSG00000259015	cML-MA	0.22	0.06	1.24E-04	4530	1.24E+00
eqtl-a-ENSG00000259015	IVW	0.22	0.06	7.81E-05	4530	1.25E+00
eqtl-a-ENSG00000259015	divw	0.22	0.06	9.66E-05	4530	1.25E+00

eqtl-a-ENSG00000259015	conmix	0.22	0.08	5.86E-03	4530	1.25E+00
eqtl-a-ENSG00000259015	raps	0.22	0.06	1.45E-04	4530	1.25E+00
finn-b-C3_RESPIRATORY_INTRATHORACIC	Wald ratio	1.00	0.07	7.11E-47	218792	2.72E+00
finn-b-C3_RESPIRATORY_INTRATHORACIC	raps	1.00	0.16	5.60E-10	218792	2.72E+00
finn-b-C3_RESPIRATORY_INTRATHORACIC_EXALLC	raps	0.99	0.16	5.34E-10	176059	2.69E+00
finn-b-C3_RESPIRATORY_INTRATHORACIC_EXALLC	Wald ratio	0.99	0.07	7.11E-47	176059	2.69E+00
finn-b-COPD_HOSPITAL	IVW	0.91	0.21	2.20E-05	218792	2.47E+00
finn-b-COPD_HOSPITAL	raps	0.85	0.19	7.00E-06	218792	2.34E+00
finn-b-COPD_LATER	raps	1.45	0.27	1.28E-07	215284	4.25E+00
finn-b-COPD_LATER	Wald ratio	1.45	0.10	3.14E-47	215284	4.25E+00
finn-b-J10_COPDNAS	raps	1.02	0.12	9.26E-17	193318	2.76E+00
finn-b-J10_COPDNAS	Wald ratio	1.02	0.08	2.02E-41	193318	2.76E+00
finn-b-J10_COPDNAS_INCLAVO	IVW	0.92	0.19	1.81E-06	218792	2.50E+00
finn-b-J10_COPDNAS_INCLAVO	raps	0.88	0.17	1.61E-07	218792	2.41E+00
ieu-a-1126	cML-MA	0.09	0.03	1.43E-03	228951	1.09E+00
ieu-a-1126	divw	0.11	0.03	1.63E-04	228951	1.11E+00
ieu-a-1126	conmix	0.07	0.04	5.04E-02	228951	1.08E+00
ieu-a-1126	raps	0.10	0.03	3.01E-04	228951	1.11E+00
ieu-a-1126	IVW	0.10	0.03	1.48E-04	228951	1.11E+00
ieu-a-1239	IVW	-0.49	0.08	6.57E-10	766345	6.10E-01
ieu-a-1239	cML-MA	-0.51	0.10	8.95E-07	766345	6.02E-01
ieu-a-1239	conmix	-0.96	0.24	8.97E-05	766345	3.85E-01
ieu-a-1239	divw	-0.51	0.08	7.14E-10	766345	6.03E-01
ieu-a-1239	raps	-0.49	0.09	9.36E-09	766345	6.11E-01
ieu-b-142	cML-MA	0.70	0.15	4.96E-06	249752	2.02E+00
ieu-b-142	IVW	0.99	0.10	4.93E-22	249752	2.68E+00
ieu-b-142	conmix	1.15	0.23	9.40E-07	249752	3.15E+00

ieu-b-142	raps	0.90	0.11	5.99E-17	249752	2.45E+00
ieu-b-142	divw	1.00	0.11	4.98E-21	249752	2.71E+00
ieu-b-25	cML-MA	0.70	0.15	4.96E-06	337334	2.02E+00
ieu-b-25	divw	1.00	0.11	4.98E-21	337334	2.71E+00
ieu-b-25	conmix	1.15	0.23	9.40E-07	337334	3.15E+00
ieu-b-25	IVW	0.99	0.10	4.93E-22	337334	2.68E+00
ieu-b-25	raps	0.90	0.11	5.99E-17	337334	2.45E+00
ieu-b-4877	raps	0.41	0.08	6.79E-07	607291	1.51E+00
ieu-b-4877	conmix	0.58	0.13	1.05E-05	607291	1.79E+00
ieu-b-4877	IVW	0.39	0.08	6.97E-07	607291	1.48E+00
ieu-b-4877	cML-MA	0.41	0.10	4.18E-05	607291	1.50E+00
ieu-b-4877	divw	0.40	0.08	7.70E-07	607291	1.50E+00
ieu-b-4879	divw	0.79	0.08	4.80E-21	472174	2.20E+00
ieu-b-4879	cML-MA	0.70	0.11	4.05E-10	472174	2.01E+00
ieu-b-4879	conmix	1.00	0.15	3.30E-11	472174	2.72E+00
ieu-b-4879	raps	0.77	0.08	3.04E-21	472174	2.15E+00
ieu-b-4879	IVW	0.78	0.05	2.56E-60	472174	2.18E+00
prot-a-1051	raps	-0.11	0.02	5.26E-07	3301	8.99E-01
prot-a-1051	conmix	-0.10	0.03	6.84E-04	3301	9.03E-01
prot-a-1051	cML-MA	-0.10	0.02	1.34E-08	3301	9.02E-01
prot-a-1051	IVW	-0.11	0.02	8.13E-09	3301	8.99E-01
prot-a-1051	divw	-0.11	0.02	1.20E-08	3301	8.99E-01
prot-a-1091	Wald ratio	-0.41	0.09	5.04E-06	3301	6.64E-01
prot-a-1091	raps	-0.41	0.11	1.56E-04	3301	6.64E-01
prot-a-1255	Wald ratio	-0.54	0.14	1.17E-04	3301	5.84E-01
prot-a-1255	raps	-0.54	0.17	1.29E-03	3301	5.84E-01
prot-a-1256	Wald ratio	-0.57	0.15	1.17E-04	3301	5.64E-01
prot-a-1621	raps	-0.17	0.04	6.31E-05	3301	8.43E-01

prot-a-1621	Wald ratio	-0.17	0.04	2.44E-05	3301	8.43E-01
prot-c-5102_55_3	IVW	-0.10	0.03	1.51E-04	3080	9.05E-01
prot-c-5102_55_3	raps	-0.10	0.03	3.46E-04	3080	9.05E-01
ukb-a-205	raps	17.11	6.31	6.71E-03	292053	2.70E+07
ukb-a-205	IVW	30.41	7.57	5.89E-05	292053	1.61E+13
ukb-a-205	divw	31.11	6.66	3.04E-06	292053	3.23E+13
ukb-a-237	conmix	1.17	0.41	4.16E-03	101726	3.21E+00
ukb-a-237	raps	0.90	0.23	8.67E-05	101726	2.47E+00
ukb-a-237	cML-MA	0.87	0.24	2.32E-04	101726	2.39E+00
ukb-a-237	IVW	0.87	0.21	4.48E-05	101726	2.38E+00
ukb-a-237	divw	0.89	0.22	7.11E-05	101726	2.44E+00
ukb-a-238	cML-MA	0.68	0.22	2.25E-03	101726	1.97E+00
ukb-a-238	raps	0.71	0.20	4.34E-04	101726	2.03E+00
ukb-a-238	conmix	1.21	0.54	2.39E-02	101726	3.35E+00
ukb-a-238	IVW	0.71	0.19	1.50E-04	101726	2.04E+00
ukb-a-238	divw	0.73	0.20	1.97E-04	101726	2.08E+00
ukb-a-28	raps	1.41	0.39	3.62E-04	241447	4.08E+00
ukb-a-28	divw	1.49	0.38	1.04E-04	241447	4.44E+00
ukb-a-28	IVW	1.45	0.38	1.09E-04	241447	4.28E+00
ukb-a-28	conmix	2.01	0.87	2.13E-02	241447	7.44E+00
ukb-a-28	cML-MA	1.35	0.46	3.46E-03	241447	3.84E+00
ukb-a-298	raps	15.73	3.43	4.46E-06	147970	6.79E+06
ukb-a-298	Wald ratio	15.73	1.73	1.12E-19	147970	6.79E+06
ukb-a-328	cML-MA	2.47	0.24	1.84E-24	78291	1.18E+01
ukb-a-328	raps	2.55	0.20	9.81E-38	78291	1.28E+01
ukb-a-328	conmix	2.53	0.33	2.78E-14	78291	1.26E+01
ukb-a-328	divw	2.56	0.19	1.61E-43	78291	1.30E+01
ukb-a-328	IVW	2.54	0.18	4.44E-47	78291	1.27E+01
ukb-a-342	cML-MA	1.63	0.33	9.89E-07	23205	5.13E+00

ukb-a-342	raps	1.99	0.41	1.26E-06	23205	7.31E+00
ukb-a-342	divw	2.29	0.40	7.10E-09	23205	9.89E+00
ukb-a-342	IVW	2.26	0.42	5.46E-08	23205	9.59E+00
ukb-a-342	conmix	2.92	0.96	2.28E-03	23205	1.85E+01
ukb-a-397	IVW	-0.67	0.17	1.37E-04	334070	5.14E-01
ukb-a-397	cML-MA	-0.65	0.24	7.77E-03	334070	5.24E-01
ukb-a-397	raps	-0.65	0.19	4.94E-04	334070	5.24E-01
ukb-a-397	conmix	-1.01	0.31	1.07E-03	334070	3.65E-01
ukb-a-397	divw	-0.68	0.18	1.38E-04	334070	5.06E-01
ukb-a-40	raps	-32.48	5.57	5.51E-09	328694	7.83E-15
ukb-a-40	Wald ratio	-32.48	2.29	9.28E-46	328694	7.83E-15
ukb-b-10831	raps	1.13	0.54	3.68E-02	142387	3.09E+00
ukb-b-10831	conmix	1.80	0.95	5.83E-02	142387	6.02E+00
ukb-b-10831	IVW	1.47	0.39	1.68E-04	142387	4.34E+00
ukb-b-10831	cML-MA	1.11	0.43	9.67E-03	142387	3.03E+00
ukb-b-10831	divw	1.50	0.54	5.17E-03	142387	4.48E+00
ukb-b-11231	Wald ratio	75.25	19.53	1.17E-04	463010	4.81E+32
ukb-b-11231	raps	75.25	23.05	1.10E-03	463010	4.81E+32
ukb-b-11615	conmix	-1.62	0.73	2.65E-02	458079	1.98E-01
ukb-b-11615	raps	-1.28	0.34	1.76E-04	458079	2.79E-01
ukb-b-11615	cML-MA	-1.26	0.42	2.37E-03	458079	2.82E-01
ukb-b-11615	IVW	-1.30	0.32	5.70E-05	458079	2.73E-01
ukb-b-11615	divw	-1.33	0.33	5.98E-05	458079	2.64E-01
ukb-b-13532	Wald ratio	-190.37	25.68	1.24E-13	462933	2.10E-83
ukb-b-13532	raps	-190.37	41.99	5.80E-06	462933	2.10E-83
ukb-b-13952	Wald ratio	78.92	5.47	4.14E-47	361823	1.89E+34
ukb-b-13952	raps	78.92	12.36	1.74E-10	361823	1.89E+34

ukb-b-14521	cML-MA	16.75	7.87	3.33E-02	401624	1.88E+07
ukb-b-14521	divw	28.76	5.90	1.08E-06	401624	3.08E+12
ukb-b-14521	raps	27.93	6.66	2.78E-05	401624	1.35E+12
ukb-b-14521	conmix	35.49	10.07	4.22E-04	401624	2.60E+15
ukb-b-14521	IVW	28.22	6.57	1.77E-05	401624	1.79E+12
ukb-b-16019	raps	41.04	7.92	2.17E-07	461378	6.67E+17
ukb-b-16019	Wald ratio	41.04	4.38	7.58E-21	461378	6.67E+17
ukb-b-16489	IVW	-0.99	0.17	7.26E-09	458079	3.72E-01
ukb-b-16489	cML-MA	-1.00	0.23	1.93E-05	458079	3.68E-01
ukb-b-16489	conmix	-1.21	0.30	5.40E-05	458079	2.99E-01
ukb-b-16489	divw	-1.01	0.18	8.12E-09	458079	3.63E-01
ukb-b-16489	raps	-0.99	0.18	3.24E-08	458079	3.73E-01
ukb-b-16751	Wald ratio	88.31	21.55	4.15E-05	463010	2.25E+38
ukb-b-16751	raps	88.31	27.24	1.19E-03	463010	2.25E+38
ukb-b-1997	raps	5.40	1.67	1.24E-03	133020	2.22E+02
ukb-b-1997	Wald ratio	5.40	1.32	4.15E-05	133020	2.22E+02
ukb-b-469	raps	1.67	0.26	1.90E-10	33229	5.31E+00
ukb-b-469	divw	1.68	0.26	7.81E-11	33229	5.38E+00
ukb-b-469	IVW	1.66	0.23	2.88E-13	33229	5.25E+00
ukb-b-4801	Wald ratio	10.70	0.79	2.02E-41	104420	4.42E+04
ukb-b-4801	raps	10.70	2.05	1.70E-07	104420	4.42E+04
ukb-b-5174	cML-MA	1.35	0.38	3.90E-04	327634	3.86E+00
ukb-b-5174	IVW	1.40	0.32	1.40E-05	327634	4.06E+00
ukb-b-5174	raps	1.42	0.34	3.14E-05	327634	4.14E+00
ukb-b-5174	conmix	1.72	0.52	8.21E-04	327634	5.61E+00
ukb-b-5174	divw	1.43	0.33	1.82E-05	327634	4.19E+00
ukb-b-6019	cML-MA	2.69	0.26	2.58E-25	108946	1.47E+01

ukb-b-6019	raps	2.74	0.22	1.56E-35	108946	1.55E+01
ukb-b-6019	IVW	2.75	0.17	1.56E-57	108946	1.57E+01
ukb-b-6019	divw	2.78	0.19	4.70E-49	108946	1.61E+01
ukb-b-6019	conmix	2.75	0.36	2.54E-14	108946	1.57E+01
ukb-b-6591	divw	-0.54	0.09	2.29E-09	406457	5.80E-01
ukb-b-6591	cML-MA	-0.50	0.13	6.03E-05	406457	6.04E-01
ukb-b-6591	IVW	-0.53	0.06	2.46E-17	406457	5.87E-01
ukb-b-6591	conmix	-0.68	0.21	1.15E-03	406457	5.06E-01
ukb-b-6591	raps	-0.51	0.09	4.04E-08	406457	5.99E-01
ukb-b-7460	cML-MA	1.06	0.34	2.03E-03	142387	2.89E+00
ukb-b-7460	IVW	1.33	0.21	1.97E-10	142387	3.77E+00
ukb-b-7460	divw	1.36	0.42	1.09E-03	142387	3.89E+00
ukb-b-7460	conmix	1.76	0.76	2.11E-02	142387	5.83E+00
ukb-b-7460	raps	1.05	0.42	1.27E-02	142387	2.86E+00
ukb-b-7647	Wald ratio	38.40	4.10	7.58E-21	446149	4.74E+16
ukb-b-7647	raps	38.40	7.44	2.49E-07	446149	4.74E+16
ukb-b-8133	Wald ratio	-8.94	0.62	4.14E-47	123894	1.31E-04
ukb-b-8133	raps	-8.94	0.96	1.86E-20	123894	1.31E-04
ukb-d-COPD_EARLYANDLATER	raps	195.56	39.09	5.65E-07	361194	8.50E+84
ukb-d-COPD_EARLYANDLATER	Wald ratio	195.56	16.71	1.25E-31	361194	8.50E+84
ukb-d-D22	divw	-20.29	4.51	6.75E-06	361194	1.54E-09
ukb-d-D22	conmix	-22.25	6.37	4.83E-04	361194	2.18E-10
ukb-d-D22	IVW	-19.82	4.24	2.90E-06	361194	2.46E-09
ukb-d-D22	raps	-20.55	4.59	7.44E-06	361194	1.19E-09
ukb-d-D22	cML-MA	-19.85	4.34	4.90E-06	361194	2.41E-09
ukb-d-K11_OTHDIG	raps	15.01	3.87	1.04E-04	361194	3.32E+06
ukb-d-K11_OTHDIG	IVW	14.98	3.66	4.18E-05	361194	3.20E+06

supplementary Table 3 The MR analysis results of between exposures and SCLC

id.exposure	method	beta	se	pval	sample_size	or
ebi-a-GCST90093341	Wald ratio	-0.74	0.18	4.38E-05	2745	4.76E-01
ebi-a-GCST90093341	raps	-0.74	0.23	1.02E-03	2745	4.76E-01
ebi-a-GCST009968	Wald ratio	2.01	0.22	1.23E-20	4772	7.44E+00
ebi-a-GCST009968	raps	2.01	0.37	7.04E-08	4772	7.44E+00
ebi-a-GCST009970	Wald ratio	1.28	0.14	9.13E-21	5185	3.59E+00
ebi-a-GCST009970	raps	1.28	0.18	3.73E-12	5185	3.59E+00
ebi-a-GCST009971	IVW	0.19	0.04	6.45E-06	5185	1.21E+00
ebi-a-GCST009971	cML-MA	0.19	0.04	6.24E-06	5185	1.21E+00
ebi-a-GCST009971	raps	0.19	0.04	1.27E-05	5185	1.21E+00
ebi-a-GCST009971	conmix	0.19	0.06	9.35E-04	5185	1.21E+00
ebi-a-GCST009971	divw	0.19	0.04	7.36E-06	5185	1.21E+00
eqtl-a-ENSG00000211785	raps	1.03	0.31	9.54E-04	5502	2.79E+00
eqtl-a-ENSG00000211785	Wald ratio	1.03	0.28	2.56E-04	5502	2.79E+00
eqtl-a-ENSG00000198563	IVW	-0.45	0.12	1.50E-04	8269	6.40E-01
eqtl-a-ENSG00000198563	raps	-0.45	0.12	2.80E-04	8269	6.40E-01
eqtl-a-ENSG00000204592	Wald ratio	1.27	0.27	1.88E-06	13344	3.56E+00
eqtl-a-ENSG00000204592	raps	1.27	0.31	3.37E-05	13344	3.56E+00
eqtl-a-ENSG00000082641	divw	0.44	0.11	1.03E-04	14263	1.56E+00
eqtl-a-ENSG00000082641	IVW	0.44	0.11	7.96E-05	14263	1.55E+00
eqtl-a-ENSG00000082641	raps	0.44	0.12	1.54E-04	14263	1.55E+00
ukb-a-343	IVW	-2.26	0.38	3.26E-09	23265	1.04E-01
ukb-a-343	raps	-2.26	0.37	1.17E-09	23265	1.05E-01
ukb-a-343	divw	-2.31	0.32	1.05E-12	23265	9.96E-02
eqtl-a-ENSG00000124549	IVW	1.14	0.22	1.60E-07	25690	3.14E+00
eqtl-a-ENSG00000124549	raps	1.15	0.25	3.75E-06	25690	3.14E+00
eqtl-a-ENSG00000100281	raps	1.33	0.38	4.25E-04	26196	3.79E+00
eqtl-a-ENSG00000100281	Wald ratio	1.33	0.32	4.08E-05	26196	3.79E+00
eqtl-a-ENSG00000188266	raps	-3.69	0.76	1.22E-06	26395	2.48E-02

eqtl-a-ENSG00000188266	Wald ratio	-3.69	0.41	9.14E-20	26395	2.48E-02
eqtl-a-ENSG00000162623	Wald ratio	1.17	0.30	8.62E-05	26609	3.24E+00
eqtl-a-ENSG00000162623	raps	1.17	0.34	4.69E-04	26609	3.24E+00
eqtl-a-ENSG00000213694	conmix	-0.36	0.16	2.76E-02	26609	7.01E-01
eqtl-a-ENSG00000213694	divw	-0.27	0.07	1.99E-04	26609	7.64E-01
eqtl-a-ENSG00000213694	IVW	-0.27	0.07	1.83E-04	26609	7.66E-01
eqtl-a-ENSG00000213694	cML-MA	-0.26	0.09	2.39E-03	26609	7.72E-01
eqtl-a-ENSG00000213694	raps	-0.27	0.07	2.90E-04	26609	7.65E-01
eqtl-a-ENSG00000065911	divw	0.40	0.11	1.58E-04	31470	1.49E+00
eqtl-a-ENSG00000065911	raps	0.40	0.11	2.31E-04	31470	1.48E+00
eqtl-a-ENSG00000065911	cML-MA	0.41	0.11	1.02E-04	31470	1.51E+00
eqtl-a-ENSG00000065911	conmix	0.41	0.16	1.11E-02	31470	1.51E+00
eqtl-a-ENSG00000065911	IVW	0.39	0.10	1.31E-04	31470	1.48E+00
eqtl-a-ENSG00000124508	conmix	0.31	0.13	1.92E-02	31470	1.37E+00
eqtl-a-ENSG00000124508	divw	0.29	0.07	1.80E-05	31470	1.34E+00
eqtl-a-ENSG00000124508	raps	0.29	0.07	2.98E-05	31470	1.34E+00
eqtl-a-ENSG00000124508	cML-MA	0.28	0.08	2.20E-04	31470	1.32E+00
eqtl-a-ENSG00000124508	IVW	0.29	0.07	1.60E-05	31470	1.34E+00
eqtl-a-ENSG00000145416	raps	0.24	0.06	1.21E-05	31470	1.28E+00
eqtl-a-ENSG00000145416	conmix	0.26	0.11	1.48E-02	31470	1.29E+00
eqtl-a-ENSG00000145416	IVW	0.24	0.05	6.53E-06	31470	1.28E+00
eqtl-a-ENSG00000145416	divw	0.24	0.05	7.13E-06	31470	1.28E+00
eqtl-a-ENSG00000145416	cML-MA	0.24	0.07	2.88E-04	31470	1.27E+00
eqtl-a-ENSG00000041357	raps	1.02	0.12	2.54E-16	31644	2.76E+00
eqtl-a-ENSG00000041357	Wald ratio	1.02	0.11	9.14E-20	31644	2.76E+00
eqtl-a-ENSG00000096654	Wald ratio	1.70	0.45	1.29E-04	31684	5.50E+00
eqtl-a-ENSG00000096654	raps	1.70	0.55	1.90E-03	31684	5.50E+00
eqtl-a-ENSG00000137218	conmix	0.51	0.25	4.16E-02	31684	1.67E+00
eqtl-a-ENSG00000137218	divw	0.46	0.11	3.32E-05	31684	1.58E+00
eqtl-a-ENSG00000137218	IVW	0.46	0.11	2.41E-05	31684	1.58E+00

eqtl-a-ENSG00000137218	raps	0.46	0.11	5.30E-05	31684	1.58E+00
eqtl-a-ENSG00000137218	cML-MA	0.44	0.12	3.30E-04	31684	1.56E+00
eqtl-a-ENSG00000168411	conmix	-0.19	0.07	1.24E-02	31684	8.30E-01
eqtl-a-ENSG00000168411	divw	-0.20	0.05	5.07E-05	31684	8.17E-01
eqtl-a-ENSG00000168411	cML-MA	-0.19	0.05	4.31E-04	31684	8.25E-01
eqtl-a-ENSG00000168411	raps	-0.20	0.05	1.16E-04	31684	8.21E-01
eqtl-a-ENSG00000168411	IVW	-0.20	0.05	9.17E-05	31684	8.18E-01
eqtl-a-ENSG00000186354	Wald ratio	-1.13	0.30	1.64E-04	31684	3.24E-01
eqtl-a-ENSG00000186354	raps	-1.13	0.34	8.25E-04	31684	3.24E-01
eqtl-a-ENSG00000186470	conmix	-0.11	0.05	2.82E-02	31684	9.00E-01
eqtl-a-ENSG00000186470	cML-MA	-0.11	0.05	2.04E-02	31684	8.96E-01
eqtl-a-ENSG00000186470	IVW	-0.14	0.04	1.88E-04	31684	8.70E-01
eqtl-a-ENSG00000186470	divw	-0.14	0.04	8.66E-05	31684	8.70E-01
eqtl-a-ENSG00000186470	raps	-0.13	0.04	1.55E-03	31684	8.82E-01
ukb-b-2732	divw	-2.62	0.34	1.35E-14	33304	7.26E-02
ukb-b-2732	IVW	-2.58	0.32	6.13E-16	33304	7.61E-02
ukb-b-2732	raps	-2.60	0.35	5.23E-14	33304	7.45E-02
ukb-a-328	raps	2.26	0.55	4.59E-05	78291	9.60E+00
ukb-a-328	cML-MA	1.76	0.71	1.33E-02	78291	5.80E+00
ukb-a-328	IVW	2.56	0.49	2.09E-07	78291	1.30E+01
ukb-a-328	conmix	2.91	1.00	3.63E-03	78291	1.84E+01
ukb-a-328	divw	2.59	0.51	5.12E-07	78291	1.33E+01
ukb-a-237	cML-MA	0.80	0.62	0.198987938	101726	2.22E+00
ukb-a-237	IVW	2.17	0.55	8.94E-05	101726	8.77E+00
ukb-a-237	raps	2.18	0.58	1.67E-04	101726	8.87E+00
ukb-a-237	conmix	3.17	1.31	1.54E-02	101726	2.39E+01
ukb-a-237	divw	2.20	0.56	9.46E-05	101726	9.04E+00
ukb-a-238	divw	2.24	0.48	2.80E-06	101726	9.42E+00
ukb-a-238	IVW	2.21	0.46	1.74E-06	101726	9.13E+00
ukb-a-238	raps	2.23	0.48	3.44E-06	101726	9.34E+00

ukb-a-238	conmix	3.05	0.92	9.11E-04	101726	2.11E+01
ukb-a-238	cML-MA	1.25	0.56	2.65E-02	101726	3.49E+00
ukb-b-4801	Wald ratio	13.12	1.45	1.72E-19	104420	5.01E+05
ukb-b-4801	raps	13.12	2.74	1.72E-06	104420	5.01E+05
ukb-b-6019	conmix	3.33	1.12	3.05E-03	108946	2.79E+01
ukb-b-6019	divw	2.84	0.56	4.68E-07	108946	1.72E+01
ukb-b-6019	raps	2.53	0.58	1.38E-05	108946	1.26E+01
ukb-b-6019	IVW	2.82	0.53	1.18E-07	108946	1.67E+01
ukb-b-6019	cML-MA	1.89	0.71	7.93E-03	108946	6.63E+00
ukb-b-10831	raps	2.87	0.59	1.09E-06	142387	1.76E+01
ukb-b-10831	cML-MA	1.61	0.68	1.86E-02	142387	5.01E+00
ukb-b-10831	divw	2.88	0.55	1.38E-07	142387	1.78E+01
ukb-b-10831	conmix	3.64	1.26	3.79E-03	142387	3.79E+01
ukb-b-10831	IVW	2.84	0.56	3.21E-07	142387	1.72E+01
ukb-b-7460	IVW	2.63	0.44	1.98E-09	142387	1.39E+01
ukb-b-7460	divw	2.66	0.43	6.98E-10	142387	1.44E+01
ukb-b-7460	cML-MA	1.72	0.62	5.89E-03	142387	5.58E+00
ukb-b-7460	conmix	3.37	0.97	4.96E-04	142387	2.89E+01
ukb-b-7460	raps	2.61	0.46	1.21E-08	142387	1.36E+01
finn-b-DM_SEVERAL_COMPLICATIONS	raps	-0.33	0.08	5.88E-05	168722	7.21E-01
finn-b-DM_SEVERAL_COMPLICATIONS	cML-MA	-0.29	0.09	1.33E-03	168722	7.48E-01
finn-b-DM_SEVERAL_COMPLICATIONS	IVW	-0.30	0.08	1.60E-04	168722	7.37E-01
finn-b-DM_SEVERAL_COMPLICATIONS	conmix	-0.43	0.13	1.48E-03	168722	6.52E-01
finn-b-DM_SEVERAL_COMPLICATIONS	divw	-0.31	0.08	8.76E-05	168722	7.33E-01
finn-b-KRA_PSY_DEMENTIA_EXMORE	cML-MA	-0.14	0.04	2.85E-04	172517	8.73E-01
finn-b-KRA_PSY_DEMENTIA_EXMORE	divw	-0.14	0.04	1.47E-04	172517	8.71E-01
finn-b-KRA_PSY_DEMENTIA_EXMORE	IVW	-0.14	0.04	1.38E-04	172517	8.71E-01
finn-b-KRA_PSY_DEMENTIA_EXMORE	conmix	-0.14	0.04	2.33E-03	172517	8.74E-01
finn-b-KRA_PSY_DEMENTIA_EXMORE	raps	-0.14	0.04	2.39E-04	172517	8.72E-01
finn-b-C3_RESPIRATORY_INTRATHORACIC_EXALLC	Wald ratio	1.15	0.13	6.51E-20	176059	3.15E+00

finn-b-C3_RESPIRATORY_INTRATHORACIC_EXALLC	raps	1.15	0.21	4.59E-08	176059	3.15E+00
finn-b-J10_COPDNAS	raps	1.25	0.18	1.00E-11	193318	3.48E+00
finn-b-J10_COPDNAS	Wald ratio	1.25	0.14	1.72E-19	193318	3.48E+00
ebi-a-GCST90000045	cML-MA	-0.84	0.29	3.81E-03	214547	4.33E-01
ebi-a-GCST90000045	conmix	-2.12	0.48	8.49E-06	214547	1.20E-01
ebi-a-GCST90000045	raps	-0.89	0.23	1.38E-04	214547	4.11E-01
ebi-a-GCST90000045	IVW	-0.83	0.22	1.47E-04	214547	4.35E-01
ebi-a-GCST90000045	divw	-0.85	0.22	1.39E-04	214547	4.25E-01
finn-b-COPD_LATER	raps	1.68	0.35	1.56E-06	215284	5.37E+00
finn-b-COPD_LATER	Wald ratio	1.68	0.18	5.15E-20	215284	5.37E+00
finn-b-AD_LO	divw	-0.10	0.03	7.73E-05	217541	9.03E-01
finn-b-AD_LO	cML-MA	-0.10	0.03	1.15E-04	217541	9.03E-01
finn-b-AD_LO	conmix	-0.10	0.03	2.28E-03	217541	9.03E-01
finn-b-AD_LO	IVW	-0.10	0.03	7.09E-05	217541	9.03E-01
finn-b-AD_LO	raps	-0.10	0.03	1.43E-04	217541	9.04E-01
finn-b-C3_RESPIRATORY_INTRATHORACIC	raps	1.16	0.21	4.72E-08	218792	3.20E+00
finn-b-C3_RESPIRATORY_INTRATHORACIC	Wald ratio	1.16	0.13	6.51E-20	218792	3.20E+00
finn-b-G6_AD_WIDE	raps	-0.11	0.03	2.49E-04	218792	8.95E-01
finn-b-G6_AD_WIDE	conmix	-0.12	0.04	2.77E-03	218792	8.89E-01
finn-b-G6_AD_WIDE	divw	-0.11	0.03	2.06E-04	218792	8.96E-01
finn-b-G6_AD_WIDE	IVW	-0.11	0.03	1.97E-04	218792	8.97E-01
finn-b-G6_AD_WIDE	cML-MA	-0.11	0.03	1.47E-03	218792	8.99E-01
finn-b-J10_COPDNAS_INCLAVO	raps	1.02	0.26	1.09E-04	218792	2.78E+00
finn-b-J10_COPDNAS_INCLAVO	IVW	1.09	0.30	2.44E-04	218792	2.98E+00
finn-b-KRA_PSY_DEMENTIA	IVW	-0.14	0.04	1.65E-04	218792	8.73E-01
finn-b-KRA_PSY_DEMENTIA	raps	-0.14	0.04	2.89E-04	218792	8.73E-01
finn-b-KRA_PSY_DEMENTIA	conmix	-0.14	0.05	2.25E-03	218792	8.70E-01
finn-b-KRA_PSY_DEMENTIA	cML-MA	-0.13	0.04	1.45E-03	218792	8.76E-01
finn-b-KRA_PSY_DEMENTIA	divw	-0.14	0.04	1.76E-04	218792	8.72E-01
ieu-b-142	divw	1.19	0.16	5.76E-14	249752	3.29E+00

ieu-b-142	IVW	1.18	0.16	3.86E-14	249752	3.25E+00
ieu-b-142	raps	1.22	0.17	2.30E-13	249752	3.39E+00
ieu-b-142	conmix	1.42	0.31	6.38E-06	249752	4.12E+00
ieu-b-142	cML-MA	1.06	0.26	4.75E-05	249752	2.90E+00
ebi-a-GCST006250	IVW	-0.48	0.13	1.99E-04	269867	6.20E-01
ebi-a-GCST006250	conmix	-0.56	0.24	1.89E-02	269867	5.70E-01
ebi-a-GCST006250	divw	-0.49	0.13	1.92E-04	269867	6.13E-01
ebi-a-GCST006250	cML-MA	-0.47	0.17	7.65E-03	269867	6.28E-01
ebi-a-GCST006250	raps	-0.45	0.14	8.68E-04	269867	6.35E-01
ieu-a-1001	conmix	-2.30	0.47	1.20E-06	293723	1.00E-01
ieu-a-1001	IVW	-1.29	0.25	4.21E-07	293723	2.76E-01
ieu-a-1001	cML-MA	-1.33	0.33	5.25E-05	293723	2.64E-01
ieu-a-1001	divw	-1.32	0.26	3.51E-07	293723	2.66E-01
ieu-a-1001	raps	-1.33	0.27	1.16E-06	293723	2.64E-01
ebi-a-GCST006368	conmix	1.24	0.24	3.13E-07	315347	3.47E+00
ebi-a-GCST006368	IVW	0.73	0.13	6.98E-08	315347	2.07E+00
ebi-a-GCST006368	raps	0.73	0.14	7.87E-08	315347	2.07E+00
ebi-a-GCST006368	divw	0.74	0.14	6.05E-08	315347	2.10E+00
ebi-a-GCST006368	cML-MA	0.72	0.18	9.44E-05	315347	2.05E+00
ieu-a-835	raps	0.61	0.17	3.03E-04	322154	1.84E+00
ieu-a-835	cML-MA	0.59	0.21	4.61E-03	322154	1.81E+00
ieu-a-835	conmix	0.98	0.28	5.37E-04	322154	2.66E+00
ieu-a-835	IVW	0.62	0.17	1.68E-04	322154	1.86E+00
ieu-a-835	divw	0.63	0.17	1.48E-04	322154	1.88E+00
ukb-a-243	raps	8.30	2.21	1.75E-04	326730	4.02E+03
ukb-a-243	IVW	8.28	2.00	3.33E-05	326730	3.95E+03
ukb-a-40	raps	-38.07	7.31	1.93E-07	328694	2.93E-17
ukb-a-40	Wald ratio	-38.07	4.18	9.14E-20	328694	2.93E-17
ukb-a-265	divw	0.56	0.11	1.16E-06	330762	1.75E+00
ukb-a-265	cML-MA	0.51	0.15	5.16E-04	330762	1.66E+00

ukb-a-265	raps	0.53	0.12	4.48E-06	330762	1.70E+00
ukb-a-265	conmix	0.97	0.21	5.00E-06	330762	2.63E+00
ukb-a-265	IVW	0.55	0.11	1.14E-06	330762	1.73E+00
ukb-a-291	divw	0.47	0.11	2.03E-05	331093	1.61E+00
ukb-a-291	cML-MA	0.43	0.15	3.46E-03	331093	1.54E+00
ukb-a-291	raps	0.44	0.11	9.14E-05	331093	1.56E+00
ukb-a-291	IVW	0.47	0.11	1.98E-05	331093	1.59E+00
ukb-a-291	conmix	0.86	0.21	3.85E-05	331093	2.37E+00
ukb-a-290	divw	0.54	0.14	1.14E-04	331113	1.71E+00
ukb-a-290	conmix	0.95	0.24	7.65E-05	331113	2.59E+00
ukb-a-290	cML-MA	0.49	0.18	6.62E-03	331113	1.64E+00
ukb-a-290	raps	0.50	0.14	4.57E-04	331113	1.65E+00
ukb-a-290	IVW	0.53	0.14	1.10E-04	331113	1.69E+00
ukb-a-264	raps	0.66	0.17	1.01E-04	331117	1.94E+00
ukb-a-264	cML-MA	0.63	0.22	4.55E-03	331117	1.89E+00
ukb-a-264	conmix	1.30	0.25	1.31E-07	331117	3.67E+00
ukb-a-264	divw	0.69	0.17	3.54E-05	331117	2.00E+00
ukb-a-264	IVW	0.68	0.16	3.33E-05	331117	1.97E+00
ukb-a-287	cML-MA	0.56	0.14	6.99E-05	331164	1.74E+00
ukb-a-287	IVW	0.55	0.11	2.37E-07	331164	1.74E+00
ukb-a-287	divw	0.57	0.11	2.36E-07	331164	1.76E+00
ukb-a-287	raps	0.55	0.11	7.35E-07	331164	1.74E+00
ukb-a-287	conmix	0.83	0.16	3.28E-07	331164	2.28E+00
ukb-a-286	cML-MA	0.82	0.20	5.12E-05	331198	2.28E+00
ukb-a-286	conmix	1.72	0.32	6.91E-08	331198	5.60E+00
ukb-a-286	IVW	0.78	0.16	5.35E-07	331198	2.18E+00
ukb-a-286	divw	0.80	0.16	5.50E-07	331198	2.22E+00
ukb-a-286	raps	0.80	0.16	7.28E-07	331198	2.23E+00
ukb-a-283	divw	0.62	0.11	2.55E-08	331226	1.86E+00
ukb-a-283	IVW	0.61	0.11	2.63E-08	331226	1.83E+00

ukb-a-283	cML-MA	0.61	0.16	7.71E-05	331226	1.85E+00
ukb-a-283	conmix	0.94	0.17	2.95E-08	331226	2.57E+00
ukb-a-283	raps	0.60	0.11	1.21E-07	331226	1.82E+00
ukb-a-282	IVW	0.83	0.16	2.63E-07	331249	2.29E+00
ukb-a-282	cML-MA	0.82	0.22	1.99E-04	331249	2.28E+00
ukb-a-282	raps	0.83	0.17	6.34E-07	331249	2.29E+00
ukb-a-282	conmix	1.77	0.34	1.62E-07	331249	5.89E+00
ukb-a-282	divw	0.84	0.16	2.73E-07	331249	2.33E+00
ukb-a-279	IVW	0.80	0.13	7.39E-10	331275	2.23E+00
ukb-a-279	conmix	1.50	0.24	3.97E-10	331275	4.48E+00
ukb-a-279	divw	0.82	0.13	7.21E-10	331275	2.27E+00
ukb-a-279	cML-MA	0.84	0.19	1.12E-05	331275	2.31E+00
ukb-a-279	raps	0.82	0.14	1.41E-09	331275	2.27E+00
ukb-a-278	cML-MA	1.10	0.26	2.06E-05	331278	2.99E+00
ukb-a-278	IVW	1.00	0.20	4.01E-07	331278	2.71E+00
ukb-a-278	conmix	2.14	0.35	1.07E-09	331278	8.49E+00
ukb-a-278	divw	1.02	0.20	3.97E-07	331278	2.77E+00
ukb-a-278	raps	1.08	0.20	1.25E-07	331278	2.94E+00
ukb-a-275	cML-MA	0.76	0.18	1.85E-05	331293	2.15E+00
ukb-a-275	divw	0.77	0.14	1.66E-08	331293	2.16E+00
ukb-a-275	raps	0.76	0.14	2.87E-08	331293	2.14E+00
ukb-a-275	IVW	0.75	0.13	1.68E-08	331293	2.12E+00
ukb-a-275	conmix	1.40	0.24	3.25E-09	331293	4.06E+00
ukb-a-274	IVW	1.07	0.21	1.98E-07	331296	2.91E+00
ukb-a-274	cML-MA	1.12	0.27	3.61E-05	331296	3.06E+00
ukb-a-274	divw	1.09	0.21	2.05E-07	331296	2.97E+00
ukb-a-274	raps	1.13	0.21	1.01E-07	331296	3.09E+00
ukb-a-274	conmix	1.88	0.34	2.99E-08	331296	6.58E+00
ukb-a-397	conmix	-3.19	0.55	5.68E-09	334070	4.12E-02
ukb-a-397	IVW	-1.71	0.33	1.50E-07	334070	1.80E-01

ukb-a-397	raps	-1.68	0.35	1.59E-06	334070	1.86E-01
ukb-a-397	cML-MA	-1.72	0.42	4.60E-05	334070	1.79E-01
ukb-a-397	divw	-1.76	0.33	1.48E-07	334070	1.73E-01
ukb-a-398	cML-MA	3.18	0.97	1.10E-03	334070	2.40E+01
ukb-a-398	raps	3.24	0.76	1.99E-05	334070	2.56E+01
ukb-a-398	divw	3.23	0.72	7.98E-06	334070	2.52E+01
ukb-a-398	IVW	3.15	0.71	9.31E-06	334070	2.33E+01
ukb-a-398	conmix	4.98	1.34	2.04E-04	334070	1.45E+02
ukb-a-399	divw	-3.25	0.65	4.67E-07	334070	3.87E-02
ukb-a-399	conmix	-5.82	1.31	8.57E-06	334070	2.98E-03
ukb-a-399	cML-MA	-3.24	0.80	5.38E-05	334070	3.92E-02
ukb-a-399	raps	-3.27	0.70	2.74E-06	334070	3.80E-02
ukb-a-399	IVW	-3.17	0.63	4.77E-07	334070	4.21E-02
ukb-a-248	cML-MA	0.63	0.13	5.48E-07	336107	1.88E+00
ukb-a-248	divw	0.63	0.10	1.26E-10	336107	1.87E+00
ukb-a-248	IVW	0.61	0.10	1.27E-10	336107	1.85E+00
ukb-a-248	conmix	1.19	0.19	1.78E-10	336107	3.29E+00
ukb-a-248	raps	0.63	0.10	3.88E-10	336107	1.87E+00
ukb-a-249	conmix	1.00	0.27	1.94E-04	336227	2.72E+00
ukb-a-249	divw	0.43	0.10	3.53E-05	336227	1.53E+00
ukb-a-249	cML-MA	0.42	0.12	7.49E-04	336227	1.52E+00
ukb-a-249	raps	0.41	0.10	8.57E-05	336227	1.51E+00
ukb-a-249	IVW	0.42	0.07	4.63E-09	336227	1.52E+00
ukb-a-382	divw	0.77	0.14	3.44E-08	336639	2.17E+00
ukb-a-382	cML-MA	0.76	0.17	1.20E-05	336639	2.13E+00
ukb-a-382	raps	0.77	0.14	9.73E-08	336639	2.15E+00
ukb-a-382	conmix	1.47	0.27	4.05E-08	336639	4.33E+00
ukb-a-382	IVW	0.76	0.14	3.39E-08	336639	2.14E+00
ieu-b-25	IVW	1.18	0.16	3.86E-14	337334	3.25E+00
ieu-b-25	conmix	1.42	0.31	6.38E-06	337334	4.12E+00

ieu-b-25	raps	1.22	0.17	2.30E-13	337334	3.39E+00
ieu-b-25	divw	1.19	0.16	5.76E-14	337334	3.29E+00
ieu-b-25	cML-MA	1.06	0.26	4.75E-05	337334	2.90E+00
ebi-a-GCST90018947	IVW	0.62	0.10	1.44E-10	359983	1.86E+00
ebi-a-GCST90018947	divw	0.63	0.10	1.51E-10	359983	1.88E+00
ebi-a-GCST90018947	conmix	1.29	0.21	3.27E-10	359983	3.65E+00
ebi-a-GCST90018947	cML-MA	0.63	0.13	4.35E-07	359983	1.88E+00
ebi-a-GCST90018947	raps	0.64	0.10	2.36E-10	359983	1.89E+00
ebi-a-GCST90018949	divw	0.42	0.10	2.38E-05	360116	1.52E+00
ebi-a-GCST90018949	raps	0.37	0.10	3.58E-04	360116	1.44E+00
ebi-a-GCST90018949	conmix	0.30	0.18	0.103733451	360116	1.35E+00
ebi-a-GCST90018949	cML-MA	0.37	0.14	7.73E-03	360116	1.44E+00
ebi-a-GCST90018949	IVW	0.41	0.10	2.28E-05	360116	1.51E+00
ukb-d-COPD_EARLYANDLATER	Wald ratio	237.23	30.61	9.12E-15	361194	1.06E+103
ukb-d-COPD_EARLYANDLATER	raps	237.23	52.93	7.41E-06	361194	1.06E+103
ukb-b-13952	Wald ratio	93.56	10.01	9.13E-21	361823	4.29E+40
ukb-b-13952	raps	93.56	16.61	1.78E-08	361823	4.29E+40
ebi-a-GCST90000047	conmix	-1.45	0.34	2.67E-05	397338	2.35E-01
ebi-a-GCST90000047	cML-MA	-0.88	0.22	8.41E-05	397338	4.17E-01
ebi-a-GCST90000047	raps	-0.89	0.18	4.34E-07	397338	4.12E-01
ebi-a-GCST90000047	divw	-0.93	0.18	1.43E-07	397338	3.95E-01
ebi-a-GCST90000047	IVW	-0.91	0.17	1.43E-07	397338	4.04E-01
ukb-b-7408	conmix	-1.41	0.39	3.54E-04	397751	2.45E-01
ukb-b-7408	IVW	-1.03	0.27	1.07E-04	397751	3.56E-01
ukb-b-7408	raps	-1.10	0.28	9.41E-05	397751	3.34E-01
ukb-b-7408	divw	-1.06	0.27	1.15E-04	397751	3.47E-01
ukb-b-7408	cML-MA	-1.04	0.30	5.75E-04	397751	3.52E-01
ebi-a-GCST90013975	raps	0.56	0.14	3.99E-05	401772	1.75E+00
ebi-a-GCST90013975	divw	0.57	0.13	1.98E-05	401772	1.76E+00
ebi-a-GCST90013975	cML-MA	0.52	0.18	4.44E-03	401772	1.68E+00

ebi-a-GCST90013975	IVW	0.56	0.13	1.94E-05	401772	1.74E+00
ebi-a-GCST90013975	conmix	1.32	0.24	1.95E-08	401772	3.75E+00
ukb-b-6591	divw	-0.99	0.18	3.07E-08	406457	3.71E-01
ukb-b-6591	IVW	-0.97	0.17	2.78E-08	406457	3.79E-01
ukb-b-6591	cML-MA	-1.00	0.22	5.49E-06	406457	3.68E-01
ukb-b-6591	conmix	-1.53	0.33	3.91E-06	406457	2.16E-01
ukb-b-6591	raps	-0.98	0.18	3.82E-08	406457	3.75E-01
ebi-a-GCST90013922	divw	2.31	0.38	7.94E-10	407521	1.00E+01
ebi-a-GCST90013922	raps	2.38	0.40	2.03E-09	407521	1.08E+01
ebi-a-GCST90013922	cML-MA	2.14	0.42	3.63E-07	407521	8.49E+00
ebi-a-GCST90013922	conmix	2.74	0.65	2.14E-05	407521	1.55E+01
ebi-a-GCST90013922	IVW	2.26	0.40	1.09E-08	407521	9.60E+00
ebi-a-GCST90013972	IVW	2.26	0.39	8.86E-09	407521	9.62E+00
ebi-a-GCST90013972	divw	2.31	0.37	6.36E-10	407521	1.00E+01
ebi-a-GCST90013972	cML-MA	2.15	0.42	2.55E-07	407521	8.57E+00
ebi-a-GCST90013972	conmix	2.74	0.64	2.01E-05	407521	1.55E+01
ebi-a-GCST90013972	raps	2.38	0.39	1.66E-09	407521	1.08E+01
ebi-a-GCST90013870	IVW	0.59	0.09	2.68E-11	407609	1.80E+00
ebi-a-GCST90013870	cML-MA	0.63	0.12	5.16E-07	407609	1.87E+00
ebi-a-GCST90013870	conmix	1.25	0.19	6.42E-11	407609	3.47E+00
ebi-a-GCST90013870	divw	0.60	0.09	2.69E-11	407609	1.82E+00
ebi-a-GCST90013870	raps	0.62	0.09	8.18E-12	407609	1.85E+00
ebi-a-GCST90013974	cML-MA	0.62	0.11	3.14E-08	407609	1.85E+00
ebi-a-GCST90013974	IVW	0.59	0.09	2.96E-11	407609	1.80E+00
ebi-a-GCST90013974	divw	0.60	0.09	2.99E-11	407609	1.83E+00
ebi-a-GCST90013974	raps	0.61	0.09	1.05E-11	407609	1.85E+00
ebi-a-GCST90013974	conmix	1.23	0.19	1.32E-10	407609	3.41E+00
ebi-a-GCST90014020	conmix	0.97	0.25	1.12E-04	407661	2.65E+00
ebi-a-GCST90014020	IVW	0.58	0.12	2.02E-06	407661	1.79E+00
ebi-a-GCST90014020	raps	0.57	0.13	8.60E-06	407661	1.77E+00

ebi-a-GCST90014020	cML-MA	0.55	0.14	1.47E-04	407661	1.73E+00
ebi-a-GCST90014020	divw	0.59	0.12	2.00E-06	407661	1.81E+00
ebi-a-GCST90014021	conmix	0.46	0.27	8.59E-02	407662	1.59E+00
ebi-a-GCST90014021	raps	0.35	0.11	1.14E-03	407662	1.42E+00
ebi-a-GCST90014021	IVW	0.40	0.10	1.02E-04	407662	1.50E+00
ebi-a-GCST90014021	divw	0.41	0.11	1.03E-04	407662	1.51E+00
ebi-a-GCST90014021	cML-MA	0.32	0.14	2.08E-02	407662	1.38E+00
ebi-a-GCST90000048	raps	-0.28	0.08	3.24E-04	418758	7.58E-01
ebi-a-GCST90000048	divw	-0.30	0.07	4.33E-05	418758	7.40E-01
ebi-a-GCST90000048	cML-MA	-0.28	0.09	1.09E-03	418758	7.56E-01
ebi-a-GCST90000048	conmix	-0.57	0.21	7.16E-03	418758	5.65E-01
ebi-a-GCST90000048	IVW	-0.29	0.07	4.64E-05	418758	7.46E-01
ukb-b-12018	IVW	22.10	5.95	2.01E-04	423692	3.97E+09
ukb-b-12018	divw	22.81	6.60	5.51E-04	423692	8.05E+09
ukb-b-12018	raps	22.40	6.59	6.76E-04	423692	5.34E+09
ieu-b-5118	divw	1.02	0.16	7.58E-11	453169	2.77E+00
ieu-b-5118	cML-MA	1.03	0.19	4.76E-08	453169	2.79E+00
ieu-b-5118	conmix	2.36	0.35	1.08E-11	453169	1.06E+01
ieu-b-5118	IVW	1.00	0.15	7.15E-11	453169	2.71E+00
ieu-b-5118	raps	1.05	0.16	3.19E-11	453169	2.85E+00
ukb-b-19393	divw	0.53	0.09	1.42E-08	454137	1.70E+00
ukb-b-19393	cML-MA	0.53	0.13	3.79E-05	454137	1.69E+00
ukb-b-19393	conmix	1.00	0.18	1.16E-08	454137	2.72E+00
ukb-b-19393	IVW	0.52	0.09	1.37E-08	454137	1.69E+00
ukb-b-19393	raps	0.52	0.10	7.72E-08	454137	1.68E+00
ukb-b-20044	conmix	0.75	0.19	7.58E-05	454588	2.11E+00
ukb-b-20044	divw	0.40	0.10	3.24E-05	454588	1.49E+00
ukb-b-20044	cML-MA	0.34	0.12	3.47E-03	454588	1.41E+00
ukb-b-20044	IVW	0.39	0.09	3.10E-05	454588	1.48E+00
ukb-b-20044	raps	0.35	0.10	3.21E-04	454588	1.42E+00

ukb-b-8909	IVW	0.60	0.14	8.99E-06	454633	1.82E+00
ukb-b-8909	conmix	1.20	0.28	2.29E-05	454633	3.33E+00
ukb-b-8909	cML-MA	0.56	0.16	5.92E-04	454633	1.76E+00
ukb-b-8909	divw	0.61	0.14	9.52E-06	454633	1.84E+00
ukb-b-8909	raps	0.57	0.14	5.70E-05	454633	1.76E+00
ukb-b-8338	IVW	0.65	0.09	1.26E-12	454684	1.92E+00
ukb-b-8338	cML-MA	0.64	0.14	3.59E-06	454684	1.90E+00
ukb-b-8338	raps	0.64	0.10	2.16E-11	454684	1.90E+00
ukb-b-8338	conmix	1.35	0.22	1.28E-09	454684	3.85E+00
ukb-b-8338	divw	0.67	0.09	1.29E-12	454684	1.95E+00
ukb-b-20188	cML-MA	0.69	0.17	7.44E-05	454724	2.00E+00
ukb-b-20188	IVW	0.70	0.13	6.38E-08	454724	2.01E+00
ukb-b-20188	raps	0.71	0.14	1.88E-07	454724	2.03E+00
ukb-b-20188	conmix	1.30	0.24	1.17E-07	454724	3.65E+00
ukb-b-20188	divw	0.71	0.13	6.46E-08	454724	2.04E+00
ukb-b-6704	raps	0.57	0.09	4.75E-10	454757	1.78E+00
ukb-b-6704	cML-MA	0.56	0.12	8.42E-06	454757	1.74E+00
ukb-b-6704	divw	0.59	0.09	9.63E-11	454757	1.80E+00
ukb-b-6704	IVW	0.58	0.09	9.62E-11	454757	1.78E+00
ukb-b-6704	conmix	1.25	0.22	1.13E-08	454757	3.51E+00
ukb-b-12854	conmix	1.97	0.32	6.40E-10	454789	7.17E+00
ukb-b-12854	cML-MA	0.84	0.18	4.19E-06	454789	2.31E+00
ukb-b-12854	IVW	0.82	0.13	4.92E-10	454789	2.28E+00
ukb-b-12854	raps	0.83	0.14	3.26E-09	454789	2.30E+00
ukb-b-12854	divw	0.84	0.14	5.18E-10	454789	2.32E+00
ukb-b-7212	raps	0.81	0.12	2.16E-11	454823	2.24E+00
ukb-b-7212	cML-MA	0.80	0.17	1.33E-06	454823	2.23E+00
ukb-b-7212	divw	0.81	0.12	6.51E-12	454823	2.25E+00
ukb-b-7212	conmix	1.73	0.26	3.56E-11	454823	5.65E+00
ukb-b-7212	IVW	0.80	0.12	6.28E-12	454823	2.22E+00

ukb-b-18377	conmix	2.27	0.39	3.72E-09	454826	9.73E+00
ukb-b-18377	cML-MA	1.11	0.23	1.43E-06	454826	3.03E+00
ukb-b-18377	IVW	1.05	0.17	9.86E-10	454826	2.86E+00
ukb-b-18377	divw	1.07	0.18	1.05E-09	454826	2.93E+00
ukb-b-18377	raps	1.10	0.18	7.05E-10	454826	3.00E+00
ukb-b-18096	IVW	0.77	0.12	1.13E-10	454846	2.16E+00
ukb-b-18096	conmix	1.65	0.27	5.19E-10	454846	5.19E+00
ukb-b-18096	raps	0.79	0.12	8.11E-11	454846	2.21E+00
ukb-b-18096	divw	0.78	0.12	1.18E-10	454846	2.19E+00
ukb-b-18096	cML-MA	0.79	0.16	1.62E-06	454846	2.20E+00
ukb-b-20531	cML-MA	0.96	0.22	1.53E-05	454854	2.60E+00
ukb-b-20531	conmix	1.70	0.27	4.31E-10	454854	5.47E+00
ukb-b-20531	IVW	0.93	0.17	4.75E-08	454854	2.54E+00
ukb-b-20531	divw	0.95	0.17	5.00E-08	454854	2.59E+00
ukb-b-20531	raps	0.97	0.18	4.24E-08	454854	2.64E+00
ukb-b-2303	cML-MA	0.72	0.11	1.45E-10	454884	2.05E+00
ukb-b-2303	conmix	1.34	0.17	1.59E-14	454884	3.82E+00
ukb-b-2303	divw	0.71	0.09	1.22E-16	454884	2.02E+00
ukb-b-2303	raps	0.71	0.09	3.85E-16	454884	2.04E+00
ukb-b-2303	IVW	0.69	0.08	1.23E-16	454884	2.00E+00
ukb-b-12039	cML-MA	0.39	0.13	2.64E-03	454893	1.48E+00
ukb-b-12039	raps	0.38	0.09	5.27E-05	454893	1.46E+00
ukb-b-12039	divw	0.39	0.09	1.91E-05	454893	1.47E+00
ukb-b-12039	conmix	0.65	0.19	4.60E-04	454893	1.92E+00
ukb-b-12039	IVW	0.38	0.09	1.87E-05	454893	1.46E+00
ebi-a-GCST90025994	conmix	1.30	0.22	4.74E-09	457756	3.67E+00
ebi-a-GCST90025994	cML-MA	0.55	0.13	4.71E-05	457756	1.73E+00
ebi-a-GCST90025994	raps	0.54	0.10	1.67E-08	457756	1.72E+00
ebi-a-GCST90025994	divw	0.50	0.10	2.21E-07	457756	1.64E+00
ebi-a-GCST90025994	IVW	0.49	0.09	2.13E-07	457756	1.63E+00

ukb-b-11615	IVW	-2.60	0.66	8.00E-05	458079	7.42E-02
ukb-b-11615	cML-MA	-2.36	0.84	4.84E-03	458079	9.45E-02
ukb-b-11615	raps	-2.41	0.73	9.71E-04	458079	8.95E-02
ukb-b-11615	conmix	-7.30	2.45	2.91E-03	458079	6.78E-04
ukb-b-11615	divw	-2.67	0.67	7.48E-05	458079	6.94E-02
ukb-b-16489	conmix	-3.20	0.50	2.44E-10	458079	4.10E-02
ukb-b-16489	cML-MA	-2.21	0.37	3.24E-09	458079	1.10E-01
ukb-b-16489	IVW	-2.16	0.30	3.77E-13	458079	1.15E-01
ukb-b-16489	divw	-2.21	0.31	3.88E-13	458079	1.09E-01
ukb-b-16489	raps	-2.21	0.30	3.30E-13	458079	1.09E-01
ukb-b-17729	raps	2.91	0.70	3.42E-05	458079	1.84E+01
ukb-b-17729	IVW	2.82	0.67	2.21E-05	458079	1.68E+01
ukb-b-17729	cML-MA	2.92	0.84	5.18E-04	458079	1.85E+01
ukb-b-17729	divw	2.89	0.68	2.03E-05	458079	1.80E+01
ukb-b-17729	conmix	4.51	1.15	9.33E-05	458079	9.10E+01
ukb-b-3855	cML-MA	-17.01	4.55	1.88E-04	459354	4.12E-08
ukb-b-3855	conmix	-18.85	5.52	6.44E-04	459354	6.50E-09
ukb-b-3855	IVW	-16.89	4.10	3.85E-05	459354	4.62E-08
ukb-b-3855	raps	-17.04	4.42	1.14E-04	459354	3.97E-08
ukb-b-3855	divw	-17.37	4.40	7.76E-05	459354	2.86E-08
ebi-a-GCST90029013	divw	-0.18	0.03	3.23E-08	461457	8.38E-01
ebi-a-GCST90029013	cML-MA	-0.18	0.04	9.05E-06	461457	8.33E-01
ebi-a-GCST90029013	raps	-0.18	0.03	4.79E-08	461457	8.35E-01
ebi-a-GCST90029013	IVW	-0.17	0.03	3.35E-08	461457	8.41E-01
ebi-a-GCST90029013	conmix	-0.32	0.06	2.47E-07	461457	7.23E-01
ukb-b-19953	IVW	0.67	0.08	7.16E-16	461460	1.96E+00
ukb-b-19953	raps	0.70	0.09	4.12E-16	461460	2.02E+00
ukb-b-19953	cML-MA	0.70	0.11	3.37E-11	461460	2.02E+00
ukb-b-19953	divw	0.69	0.09	7.25E-16	461460	1.99E+00
ukb-b-19953	conmix	1.40	0.19	3.81E-14	461460	4.07E+00

ukb-b-11842	conmix	0.69	0.21	1.08E-03	461632	1.99E+00
ukb-b-11842	IVW	0.40	0.09	6.88E-06	461632	1.49E+00
ukb-b-11842	cML-MA	0.39	0.12	1.73E-03	461632	1.48E+00
ukb-b-11842	raps	0.39	0.09	2.57E-05	461632	1.48E+00
ukb-b-11842	divw	0.41	0.09	7.11E-06	461632	1.50E+00
ukb-b-9405	raps	0.72	0.12	4.49E-09	462166	2.05E+00
ukb-b-9405	IVW	0.70	0.12	2.19E-09	462166	2.02E+00
ukb-b-9405	conmix	1.72	0.28	8.01E-10	462166	5.60E+00
ukb-b-9405	cML-MA	0.72	0.16	5.38E-06	462166	2.05E+00
ukb-b-9405	divw	0.72	0.12	2.20E-09	462166	2.04E+00
ebi-a-GCST90029014	divw	1.60	0.32	7.26E-07	468170	4.97E+00
ebi-a-GCST90029014	IVW	1.56	0.32	7.80E-07	468170	4.78E+00
ebi-a-GCST90029014	raps	1.45	0.33	9.15E-06	468170	4.26E+00
ebi-a-GCST90029014	conmix	1.31	0.52	1.22E-02	468170	3.72E+00
ebi-a-GCST90029014	cML-MA	1.44	0.39	2.58E-04	468170	4.21E+00
ebi-a-GCST90029012	divw	-2.21	0.36	1.00E-09	470941	1.09E-01
ebi-a-GCST90029012	IVW	-2.16	0.35	9.94E-10	470941	1.15E-01
ebi-a-GCST90029012	cML-MA	-2.13	0.46	3.06E-06	470941	1.19E-01
ebi-a-GCST90029012	conmix	-3.25	0.62	1.88E-07	470941	3.88E-02
ebi-a-GCST90029012	raps	-2.16	0.37	3.98E-09	470941	1.16E-01
ebi-a-GCST90018902	raps	1.66	0.36	4.68E-06	477734	5.28E+00
ebi-a-GCST90018902	Wald ratio	1.66	0.18	9.14E-20	477734	5.28E+00
ebi-a-GCST90029007	conmix	1.25	0.17	8.74E-13	532396	3.48E+00
ebi-a-GCST90029007	cML-MA	0.64	0.11	1.94E-08	532396	1.90E+00
ebi-a-GCST90029007	IVW	0.62	0.08	1.70E-14	532396	1.86E+00
ebi-a-GCST90029007	raps	0.64	0.08	1.00E-14	532396	1.90E+00
ebi-a-GCST90029007	divw	0.63	0.08	1.69E-14	532396	1.88E+00
ebi-a-GCST90000514	raps	0.62	0.15	5.43E-05	602604	1.86E+00
ebi-a-GCST90000514	divw	0.60	0.15	7.15E-05	602604	1.82E+00
ebi-a-GCST90000514	conmix	1.22	0.30	4.15E-05	602604	3.40E+00

ebi-a-GCST90000514	IVW	0.58	0.15	8.06E-05	602604	1.79E+00
ebi-a-GCST90000514	cML-MA	0.60	0.18	1.07E-03	602604	1.81E+00
ieu-b-40	divw	0.58	0.09	7.47E-11	681275	1.78E+00
ieu-b-40	cML-MA	0.59	0.11	2.86E-07	681275	1.80E+00
ieu-b-40	raps	0.58	0.09	1.01E-10	681275	1.79E+00
ieu-b-40	IVW	0.57	0.09	7.55E-11	681275	1.77E+00
ieu-b-40	conmix	1.27	0.20	1.63E-10	681275	3.55E+00
ieu-a-1239	cML-MA	-0.95	0.18	1.71E-07	766345	3.86E-01
ieu-a-1239	IVW	-0.93	0.14	1.99E-11	766345	3.95E-01
ieu-a-1239	raps	-0.96	0.14	2.20E-11	766345	3.84E-01
ieu-a-1239	conmix	-1.74	0.28	4.92E-10	766345	1.76E-01
ieu-a-1239	divw	-0.95	0.14	1.97E-11	766345	3.87E-01
prot-c-5102_55_3	raps	-0.18	0.05	2.42E-04	3080	8.32E-01
prot-c-5102_55_3	IVW	-0.18	0.05	1.35E-04	3080	8.32E-01
ukb-d-30710_raw	IVW	0.13	0.03	2.31E-04	469000	1.14E+00
ukb-d-30710_raw	conmix	0.13	0.04	3.74E-03	469000	1.14E+00
ukb-d-30710_raw	raps	0.13	0.04	2.61E-04	469000	1.14E+00
ukb-d-30710_raw	divw	0.13	0.03	2.14E-04	469000	1.14E+00
ukb-d-30710_raw	cML-MA	0.11	0.05	2.32E-02	469000	1.12E+00

supplementary Table 4 The MR analysis results of between exposures and LC

id.exposure	method	beta	se	pval	sample_size	or
ebi-a-GCST90101889	Wald ratio	0.51	0.07	1.55E-14	902	1.67E+00
ebi-a-GCST90101889	raps	0.51	0.11	5.69E-06	902	1.67E+00
ebi-a-GCST90093332	Wald ratio	-0.40	0.07	4.00E-09	1145	6.68E-01
ebi-a-GCST90093332	raps	-0.40	0.10	7.48E-05	1145	6.68E-01
ebi-a-GCST90093341	Wald ratio	-0.30	0.07	3.66E-05	2745	7.43E-01
ebi-a-GCST90093341	raps	-0.30	0.09	9.39E-04	2745	7.43E-01
prot-a-1051	conmix	-0.07	0.02	8.15E-04	3301	9.36E-01
prot-a-1051	divw	-0.07	0.02	1.69E-03	3301	9.37E-01
prot-a-1051	IVW	-0.07	0.01	1.15E-06	3301	9.37E-01
prot-a-1051	raps	-0.07	0.01	2.63E-06	3301	9.37E-01
prot-a-1051	cML-MA	-0.06	0.02	3.44E-04	3301	9.39E-01
prot-a-1124	IVW	0.11	0.03	2.75E-04	3301	1.11E+00
prot-a-1124	raps	0.11	0.03	5.29E-04	3301	1.11E+00
prot-a-1238	IVW	-0.09	0.02	5.39E-05	3301	9.18E-01
prot-a-1238	raps	-0.09	0.02	1.06E-04	3301	9.18E-01
prot-a-1347	raps	-0.16	0.04	1.72E-04	3301	8.53E-01
prot-a-1347	IVW	-0.16	0.04	1.02E-04	3301	8.54E-01
prot-a-2470	divw	-0.05	0.01	1.35E-03	3301	9.54E-01
prot-a-2470	conmix	-0.05	0.02	7.85E-04	3301	9.51E-01
prot-a-2470	IVW	-0.05	0.01	3.55E-04	3301	9.54E-01
prot-a-2470	raps	-0.05	0.01	5.25E-04	3301	9.54E-01
prot-a-2470	cML-MA	-0.05	0.01	1.58E-04	3301	9.55E-01
prot-a-2481	IVW	0.22	0.05	1.27E-05	3301	1.25E+00
prot-a-2481	raps	0.22	0.06	7.31E-05	3301	1.25E+00

prot-a-725	raps	0.07	0.02	1.11E-04	3301	1.07E+00
prot-a-725	Wald ratio	0.07	0.02	6.52E-05	3301	1.07E+00
prot-a-885	Wald ratio	0.40	0.10	4.06E-05	3301	1.49E+00
prot-a-885	raps	0.40	0.12	1.34E-03	3301	1.49E+00
eqtl-a-ENSG00000259015	IVW	0.15	0.04	3.34E-04	4530	1.16E+00
eqtl-a-ENSG00000259015	cML-MA	0.14	0.04	2.39E-04	4530	1.15E+00
eqtl-a-ENSG00000259015	conmix	0.16	0.08	4.17E-02	4530	1.17E+00
eqtl-a-ENSG00000259015	raps	0.15	0.04	5.43E-04	4530	1.16E+00
eqtl-a-ENSG00000259015	divw	0.15	0.07	4.85E-02	4530	1.16E+00
ebi-a-GCST009966	raps	-0.79	0.14	3.58E-08	4772	4.52E-01
ebi-a-GCST009966	Wald ratio	-0.79	0.09	5.03E-19	4772	4.52E-01
ebi-a-GCST009968	raps	1.82	0.29	1.79E-10	4772	6.19E+00
ebi-a-GCST009968	Wald ratio	1.82	0.09	6.61E-102	4772	6.19E+00
eqtl-a-ENSG00000223534	conmix	-0.16	0.04	4.43E-04	4994	8.55E-01
eqtl-a-ENSG00000223534	IVW	-0.14	0.04	5.69E-04	4994	8.70E-01
eqtl-a-ENSG00000223534	divw	-0.14	0.03	2.26E-05	4994	8.69E-01
eqtl-a-ENSG00000223534	raps	-0.14	0.02	5.57E-12	4994	8.66E-01
eqtl-a-ENSG00000223534	cML-MA	-0.13	0.04	3.67E-03	4994	8.78E-01
eqtl-a-ENSG00000239415	raps	0.11	0.03	4.05E-04	4994	1.11E+00
eqtl-a-ENSG00000239415	cML-MA	0.10	0.03	1.12E-04	4994	1.11E+00
eqtl-a-ENSG00000239415	divw	0.11	0.03	1.71E-03	4994	1.11E+00
eqtl-a-ENSG00000239415	conmix	0.11	0.01	9.04E-15	4994	1.11E+00
eqtl-a-ENSG00000239415	IVW	0.11	0.03	2.63E-04	4994	1.11E+00

ebi-a-GCST009965	Wald ratio	1.16	0.05	1.16E-101	5185	3.19E+00
ebi-a-GCST009965	raps	1.16	0.12	6.48E-21	5185	3.19E+00
ebi-a-GCST009970	Wald ratio	1.16	0.05	1.16E-101	5185	3.18E+00
ebi-a-GCST009970	raps	1.16	0.12	1.30E-21	5185	3.18E+00
ebi-a-GCST009971	cML-MA	0.13	0.06	3.29E-02	5185	1.14E+00
ebi-a-GCST009971	conmix	0.18	0.08	1.96E-02	5185	1.20E+00
ebi-a-GCST009971	divw	0.16	0.07	1.99E-02	5185	1.17E+00
ebi-a-GCST009971	raps	0.17	0.02	5.88E-25	5185	1.19E+00
ebi-a-GCST009971	IVW	0.16	0.03	4.80E-10	5185	1.17E+00
eqtl-a-ENSG00000166763	IVW	-0.11	0.03	2.58E-04	5502	8.93E-01
eqtl-a-ENSG00000166763	raps	-0.11	0.03	3.99E-04	5502	8.93E-01
eqtl-a-ENSG00000211785	raps	0.94	0.15	1.14E-09	5502	2.57E+00
eqtl-a-ENSG00000211785	Wald ratio	0.94	0.11	5.71E-18	5502	2.57E+00
eqtl-a-ENSG00000227598	conmix	0.15	0.05	1.88E-03	5502	1.16E+00
eqtl-a-ENSG00000227598	IVW	0.13	0.03	6.42E-07	5502	1.14E+00
eqtl-a-ENSG00000227598	divw	0.13	0.04	6.32E-04	5502	1.14E+00
eqtl-a-ENSG00000227598	cML-MA	0.12	0.02	9.92E-10	5502	1.12E+00
eqtl-a-ENSG00000227598	raps	0.14	0.03	1.24E-07	5502	1.14E+00
eqtl-a-ENSG00000260103	Wald ratio	-0.31	0.08	3.44E-05	5502	7.32E-01
eqtl-a-ENSG00000260103	raps	-0.31	0.08	1.09E-04	5502	7.32E-01
ebi-a-GCST90006924	Wald ratio	0.34	0.08	4.55E-05	5915	1.41E+00
ebi-a-GCST90006924	raps	0.34	0.10	4.29E-04	5915	1.41E+00
ebi-a-GCST90027596	Wald ratio	-0.29	0.08	5.05E-04	7738	7.47E-01

ebi-a-GCST90027596	raps	-0.29	0.10	3.96E-03	7738	7.47E-01
ebi-a-GCST90006898	IVW	-0.38	0.10	2.50E-04	7763	6.83E-01
ebi-a-GCST90006898	raps	-0.38	0.12	1.59E-03	7763	6.83E-01
met-a-510	raps	1.35	0.42	1.47E-03	7805	3.84E+00
met-a-510	Wald ratio	1.35	0.35	1.31E-04	7805	3.84E+00
bbj-a-73	conmix	0.01	0.00	2.04E-10	8383	1.01E+00
bbj-a-73	raps	-0.05	0.02	2.76E-03	8383	9.53E-01
bbj-a-73	divw	-0.06	0.02	1.56E-02	8383	9.42E-01
bbj-a-73	cML-MA	-0.04	0.01	1.54E-08	8383	9.61E-01
bbj-a-73	IVW	-0.06	0.02	3.27E-04	8383	9.43E-01
ubm-a-3048	raps	0.61	0.20	2.02E-03	8411	1.84E+00
ubm-a-3048	Wald ratio	0.61	0.16	1.39E-04	8411	1.84E+00
ebi-a-GCST90006921	raps	0.66	0.19	5.04E-04	8735	1.93E+00
ebi-a-GCST90006921	IVW	0.66	0.17	1.42E-04	8735	1.93E+00
ebi-a-GCST90006923	Wald ratio	1.00	0.26	1.38E-04	8735	2.73E+00
ebi-a-GCST90006923	raps	1.00	0.32	1.50E-03	8735	2.73E+00
eqtl-a-ENSG00000130755	raps	0.31	0.09	2.48E-04	9188	1.37E+00
eqtl-a-ENSG00000130755	Wald ratio	0.31	0.08	7.80E-05	9188	1.37E+00
eqtl-a-ENSG00000175164	raps	0.07	0.02	3.59E-04	9188	1.07E+00
eqtl-a-ENSG00000175164	cML-MA	0.07	0.02	2.85E-03	9188	1.07E+00
eqtl-a-ENSG00000175164	divw	0.07	0.02	1.26E-05	9188	1.08E+00
eqtl-a-ENSG00000175164	IVW	0.07	0.02	2.26E-04	9188	1.07E+00
eqtl-a-ENSG00000175164	conmix	0.07	0.02	7.98E-06	9188	1.07E+00
eqtl-a-ENSG00000204592	Wald ratio	0.49	0.11	3.84E-06	13344	1.63E+00

eqtl-a-ENSG00000204592	raps	0.49	0.12	5.24E-05	13344	1.63E+00
eqtl-a-ENSG00000243753	Wald ratio	0.50	0.12	2.56E-05	13344	1.65E+00
eqtl-a-ENSG00000243753	raps	0.50	0.14	2.41E-04	13344	1.65E+00
ebi-a-GCST90060127	IVW	0.23	0.06	2.00E-04	13814	1.26E+00
ebi-a-GCST90060127	raps	0.24	0.07	4.20E-04	13814	1.27E+00
ebi-a-GCST90060294	IVW	0.69	0.16	2.14E-05	13814	2.00E+00
ebi-a-GCST90060294	raps	0.70	0.19	3.01E-04	13814	2.01E+00
ebi-a-GCST90060337	raps	0.38	0.11	9.09E-04	13814	1.46E+00
ebi-a-GCST90060337	IVW	0.38	0.11	3.77E-04	13814	1.46E+00
ebi-a-GCST90060342	IVW	0.28	0.08	4.08E-04	13814	1.33E+00
ebi-a-GCST90060342	raps	0.29	0.09	9.39E-04	13814	1.33E+00
ebi-a-GCST90060514	raps	0.17	0.05	1.02E-03	13814	1.18E+00
ebi-a-GCST90060514	IVW	0.17	0.05	5.54E-04	13814	1.18E+00
ebi-a-GCST90060514	conmix	0.21	0.07	4.27E-03	13814	1.24E+00
ebi-a-GCST90060514	cML-MA	0.16	0.05	8.19E-04	13814	1.18E+00
ebi-a-GCST90060514	divw	0.17	0.05	1.31E-03	13814	1.18E+00
eqtl-a-ENSG00000101695	raps	-0.19	0.06	1.00E-03	14263	8.30E-01
eqtl-a-ENSG00000101695	divw	-0.19	0.09	2.53E-02	14263	8.25E-01
eqtl-a-ENSG00000101695	IVW	-0.19	0.05	4.81E-04	14263	8.27E-01
eqtl-a-ENSG00000112812	raps	0.17	0.05	1.55E-04	14263	1.19E+00
eqtl-a-ENSG00000112812	IVW	0.17	0.04	8.23E-05	14263	1.19E+00
eqtl-a-ENSG00000141298	conmix	0.09	0.02	8.52E-07	14263	1.09E+00
eqtl-a-ENSG00000141298	raps	0.09	0.02	9.72E-05	14263	1.09E+00
eqtl-a-ENSG00000141298	cML-MA	0.09	0.03	9.24E-03	14263	1.09E+00
eqtl-a-ENSG00000141298	IVW	0.09	0.02	5.58E-05	14263	1.09E+00
eqtl-a-ENSG00000141298	divw	0.09	0.03	6.70E-03	14263	1.10E+00
eqtl-a-ENSG00000179361	IVW	-0.31	0.07	4.92E-06	14263	7.32E-01

eqtl-a-ENSG00000179361	raps	-0.31	0.07	2.13E-05	14263	7.32E-01
ebi-a-GCST90012024	divw	-0.13	0.05	1.68E-02	21758	8.79E-01
ebi-a-GCST90012024	IVW	-0.13	0.03	2.61E-04	21758	8.80E-01
ebi-a-GCST90012024	conmix	-0.18	0.03	1.51E-07	21758	8.37E-01
ebi-a-GCST90012024	raps	-0.15	0.03	2.23E-05	21758	8.63E-01
ebi-a-GCST90012024	cML-MA	-0.13	0.06	3.60E-02	21758	8.76E-01
ukb-a-342	cML-MA	1.68	0.64	8.50E-03	23205	5.34E+00
ukb-a-342	raps	2.14	0.44	1.19E-06	23205	8.53E+00
ukb-a-342	conmix	3.15	0.88	3.67E-04	23205	2.34E+01
ukb-a-342	divw	2.47	1.44	8.54E-02	23205	1.18E+01
ukb-a-342	IVW	2.44	0.48	3.74E-07	23205	1.14E+01
ukb-a-344	IVW	0.70	0.17	3.35E-05	23332	2.02E+00
ukb-a-344	raps	0.70	0.19	2.81E-04	23332	2.02E+00
eqtl-a-ENSG00000106009	conmix	0.14	0.03	8.16E-07	25192	1.16E+00
eqtl-a-ENSG00000106009	cML-MA	0.13	0.02	8.82E-11	25192	1.14E+00
eqtl-a-ENSG00000106009	IVW	0.13	0.03	3.02E-05	25192	1.14E+00
eqtl-a-ENSG00000106009	raps	0.14	0.03	4.92E-05	25192	1.15E+00
eqtl-a-ENSG00000106009	divw	0.14	0.03	4.05E-05	25192	1.14E+00
ebi-a-GCST90016675	raps	-0.37	0.10	1.85E-04	25617	6.93E-01
ebi-a-GCST90016675	IVW	-0.36	0.10	2.78E-04	25617	7.00E-01
ebi-a-GCST90016675	divw	-0.36	0.09	3.59E-05	25617	6.95E-01
ebi-a-GCST90016675	cML-MA	-0.35	0.12	3.39E-03	25617	7.07E-01
ebi-a-GCST90016675	conmix	-0.41	0.10	1.81E-05	25617	6.65E-01
eqtl-a-ENSG00000174007	raps	-0.08	0.02	1.43E-04	25690	9.22E-01
eqtl-a-ENSG00000174007	conmix	-0.09	0.02	1.79E-04	25690	9.15E-01
eqtl-a-ENSG00000174007	cML-MA	-0.08	0.19	0.685033937	25690	9.25E-01

eqtl-a-ENSG00000174007	divw	-0.08	0.02	4.72E-05	25690	9.22E-01
eqtl-a-ENSG00000174007	IVW	-0.08	0.02	9.09E-05	25690	9.23E-01
eqtl-a-ENSG00000197077	raps	0.09	0.03	5.37E-04	26181	1.09E+00
eqtl-a-ENSG00000197077	cML-MA	0.08	0.02	1.08E-06	26181	1.08E+00
eqtl-a-ENSG00000197077	IVW	0.08	0.02	3.33E-04	26181	1.09E+00
eqtl-a-ENSG00000197077	divw	0.08	0.02	3.09E-06	26181	1.09E+00
eqtl-a-ENSG00000197077	conmix	0.15	0.03	3.39E-06	26181	1.16E+00
eqtl-a-ENSG00000002016	Wald ratio	0.47	0.12	9.75E-05	26395	1.60E+00
eqtl-a-ENSG00000002016	raps	0.47	0.14	6.14E-04	26395	1.60E+00
eqtl-a-ENSG00000188266	Wald ratio	-3.37	0.16	5.37E-98	26395	3.43E-02
eqtl-a-ENSG00000188266	raps	-3.37	0.60	2.44E-08	26395	3.43E-02
eqtl-a-ENSG00000026297	divw	0.07	0.03	1.79E-02	26609	1.07E+00
eqtl-a-ENSG00000026297	conmix	0.07	0.08	0.388389176	26609	1.08E+00
eqtl-a-ENSG00000026297	cML-MA	0.06	0.01	2.74E-14	26609	1.06E+00
eqtl-a-ENSG00000026297	IVW	0.07	0.02	2.32E-05	26609	1.07E+00
eqtl-a-ENSG00000026297	raps	0.07	0.02	3.50E-05	26609	1.07E+00
eqtl-a-ENSG00000167004	raps	0.30	0.09	9.70E-04	26609	1.34E+00
eqtl-a-ENSG00000167004	Wald ratio	0.30	0.08	4.24E-04	26609	1.34E+00
eqtl-a-ENSG00000177706	divw	-0.10	0.02	1.72E-09	26609	9.04E-01
eqtl-a-ENSG00000177706	cML-MA	-0.10	0.03	2.55E-03	26609	9.07E-01
eqtl-a-ENSG00000177706	IVW	-0.10	0.03	1.09E-04	26609	9.04E-01
eqtl-a-ENSG00000177706	raps	-0.10	0.03	2.17E-04	26609	9.06E-01
eqtl-a-ENSG00000177706	conmix	-0.10	0.03	7.12E-04	26609	9.03E-01
eqtl-a-ENSG00000189298	IVW	0.16	0.04	4.35E-06	26609	1.18E+00
eqtl-a-ENSG00000189298	raps	0.16	0.04	9.91E-06	26609	1.18E+00

ubm-b-1447	IVW	-0.61	0.17	3.99E-04	30056	5.41E-01
ubm-b-1447	raps	-0.62	0.20	1.50E-03	30056	5.38E-01
eqtl-a-ENSG00000100462	raps	-0.08	0.02	6.37E-04	30551	9.26E-01
eqtl-a-ENSG00000100462	IVW	-0.08	0.02	4.43E-04	30551	9.26E-01
eqtl-a-ENSG00000100462	divw	-0.08	0.03	3.31E-03	30551	9.26E-01
eqtl-a-ENSG00000100462	conmix	-0.08	0.01	1.11E-08	30551	9.27E-01
eqtl-a-ENSG00000100462	cML-MA	-0.08	0.04	5.20E-02	30551	9.26E-01
eqtl-a-ENSG00000119403	cML-MA	0.11	0.01	3.96E-14	30935	1.11E+00
eqtl-a-ENSG00000119403	conmix	0.12	0.03	5.19E-06	30935	1.12E+00
eqtl-a-ENSG00000119403	IVW	0.11	0.03	2.13E-04	30935	1.11E+00
eqtl-a-ENSG00000119403	divw	0.11	0.04	5.27E-03	30935	1.11E+00
eqtl-a-ENSG00000119403	raps	0.11	0.03	4.20E-04	30935	1.11E+00
eqtl-a-ENSG00000198643	Wald ratio	-0.17	0.05	5.63E-04	31050	8.45E-01
eqtl-a-ENSG00000198643	raps	-0.17	0.05	9.18E-04	31050	8.45E-01
ubm-b-1564	raps	-0.90	0.30	2.75E-03	31356	4.09E-01
ubm-b-1564	Wald ratio	-0.90	0.25	3.78E-04	31356	4.09E-01
ubm-b-1571	IVW	0.56	0.16	4.76E-04	31356	1.75E+00
ubm-b-1571	divw	0.58	0.16	1.96E-04	31356	1.78E+00
ubm-b-1571	raps	0.57	0.16	3.80E-04	31356	1.78E+00
eqtl-a-ENSG00000169045	IVW	-0.11	0.02	8.59E-06	31430	8.96E-01
eqtl-a-ENSG00000169045	divw	-0.11	0.04	3.17E-03	31430	8.96E-01
eqtl-a-ENSG00000169045	raps	-0.11	0.03	1.77E-05	31430	8.92E-01
eqtl-a-ENSG00000169045	conmix	-0.13	0.03	3.63E-05	31430	8.78E-01
eqtl-a-ENSG00000169045	cML-MA	-0.11	0.03	1.96E-03	31430	8.98E-01
eqtl-a-ENSG00000087053	Wald ratio	-0.31	0.08	2.61E-04	31470	7.34E-01

eqtl-a-ENSG00000087053	raps	-0.31	0.09	7.05E-04	31470	7.34E-01
eqtl-a-ENSG00000119402	Wald ratio	0.26	0.07	4.03E-04	31470	1.30E+00
eqtl-a-ENSG00000119402	raps	0.26	0.08	8.56E-04	31470	1.30E+00
eqtl-a-ENSG00000145416	conmix	0.08	0.03	1.01E-02	31470	1.08E+00
eqtl-a-ENSG00000145416	divw	0.08	0.01	1.34E-08	31470	1.08E+00
eqtl-a-ENSG00000145416	IVW	0.08	0.02	1.69E-04	31470	1.08E+00
eqtl-a-ENSG00000145416	raps	0.08	0.02	2.57E-04	31470	1.08E+00
eqtl-a-ENSG00000145416	cML-MA	0.08	0.02	1.78E-05	31470	1.08E+00
eqtl-a-ENSG00000158406	raps	0.09	0.02	1.24E-04	31470	1.09E+00
eqtl-a-ENSG00000158406	divw	0.09	0.04	1.99E-02	31470	1.10E+00
eqtl-a-ENSG00000158406	IVW	0.09	0.02	1.10E-05	31470	1.10E+00
eqtl-a-ENSG00000158406	cML-MA	0.05	0.02	4.97E-03	31470	1.05E+00
eqtl-a-ENSG00000158406	conmix	0.11	0.03	2.37E-04	31470	1.11E+00
eqtl-a-ENSG00000169554	raps	0.39	0.12	1.24E-03	31470	1.48E+00
eqtl-a-ENSG00000169554	IVW	0.39	0.11	3.77E-04	31470	1.47E+00
eqtl-a-ENSG00000041357	raps	0.94	0.06	7.05E-54	31644	2.57E+00
eqtl-a-ENSG00000041357	IVW	0.94	0.05	1.98E-77	31644	2.56E+00
eqtl-a-ENSG00000119487	raps	0.28	0.08	5.41E-04	31644	1.32E+00
eqtl-a-ENSG00000119487	Wald ratio	0.28	0.07	2.27E-04	31644	1.32E+00
eqtl-a-ENSG00000138496	IVW	-0.27	0.08	4.05E-04	31644	7.61E-01
eqtl-a-ENSG00000138496	raps	-0.27	0.08	8.92E-04	31644	7.61E-01
eqtl-a-ENSG00000156414	cML-MA	-0.08	0.02	1.01E-05	31644	9.24E-01
eqtl-a-ENSG00000156414	divw	-0.08	0.02	1.91E-04	31644	9.23E-01
eqtl-a-ENSG00000156414	conmix	-0.09	0.03	7.88E-04	31644	9.14E-01
eqtl-a-ENSG00000156414	raps	-0.08	0.02	1.50E-05	31644	9.22E-01
eqtl-a-ENSG00000156414	IVW	-0.08	0.02	6.06E-06	31644	9.23E-01

eqtl-a-ENSG00000174749	IVW	-0.09	0.03	2.56E-04	31644	9.11E-01
eqtl-a-ENSG00000174749	raps	-0.09	0.03	9.39E-04	31644	9.10E-01
eqtl-a-ENSG00000174749	divw	-0.09	0.04	2.92E-02	31644	9.11E-01
eqtl-a-ENSG00000174749	cML-MA	-0.09	0.02	4.37E-08	31644	9.14E-01
eqtl-a-ENSG00000174749	conmix	-0.10	0.03	9.73E-05	31644	9.06E-01
eqtl-a-ENSG00000010818	raps	0.27	0.08	6.54E-04	31684	1.31E+00
eqtl-a-ENSG00000010818	divw	0.27	0.15	6.67E-02	31684	1.32E+00
eqtl-a-ENSG00000010818	IVW	0.27	0.07	2.96E-04	31684	1.31E+00
eqtl-a-ENSG00000092330	Wald ratio	0.81	0.17	3.18E-06	31684	2.24E+00
eqtl-a-ENSG00000092330	raps	0.81	0.22	1.82E-04	31684	2.24E+00
eqtl-a-ENSG00000095261	IVW	-0.14	0.04	1.50E-04	31684	8.71E-01
eqtl-a-ENSG00000095261	raps	-0.14	0.04	2.52E-04	31684	8.71E-01
eqtl-a-ENSG00000095261	divw	-0.14	0.05	6.35E-03	31684	8.71E-01
eqtl-a-ENSG00000101574	IVW	-0.17	0.05	3.74E-04	31684	8.47E-01
eqtl-a-ENSG00000101574	raps	-0.17	0.05	6.25E-04	31684	8.46E-01
eqtl-a-ENSG00000101574	cML-MA	-0.15	0.03	6.90E-09	31684	8.57E-01
eqtl-a-ENSG00000101574	conmix	-0.23	0.03	6.99E-13	31684	7.98E-01
eqtl-a-ENSG00000101574	divw	-0.17	0.07	1.26E-02	31684	8.46E-01
eqtl-a-ENSG00000103160	conmix	0.09	0.03	1.08E-03	31684	1.09E+00
eqtl-a-ENSG00000103160	IVW	0.08	0.02	4.40E-04	31684	1.09E+00
eqtl-a-ENSG00000103160	divw	0.09	0.03	2.24E-03	31684	1.09E+00
eqtl-a-ENSG00000103160	raps	0.09	0.02	6.43E-04	31684	1.09E+00
eqtl-a-ENSG00000103160	cML-MA	0.08	0.01	1.74E-20	31684	1.08E+00
eqtl-a-ENSG00000107890	raps	0.11	0.03	1.35E-04	31684	1.12E+00
eqtl-a-ENSG00000107890	IVW	0.11	0.03	8.15E-05	31684	1.12E+00
eqtl-a-ENSG00000107890	conmix	0.11	0.03	7.36E-04	31684	1.12E+00

eqtl-a-ENSG00000107890	divw	0.11	0.04	3.06E-03	31684	1.12E+00
eqtl-a-ENSG00000107890	cML-MA	0.11	0.03	8.83E-04	31684	1.12E+00
eqtl-a-ENSG00000111906	conmix	-0.05	0.02	7.06E-03	31684	9.48E-01
eqtl-a-ENSG00000111906	raps	-0.05	0.01	6.80E-04	31684	9.55E-01
eqtl-a-ENSG00000111906	divw	-0.05	0.01	7.81E-04	31684	9.56E-01
eqtl-a-ENSG00000111906	cML-MA	-0.04	0.01	2.37E-05	31684	9.59E-01
eqtl-a-ENSG00000111906	IVW	-0.05	0.01	4.39E-04	31684	9.56E-01
eqtl-a-ENSG00000134851	raps	-0.33	0.10	7.83E-04	31684	7.20E-01
eqtl-a-ENSG00000134851	Wald ratio	-0.33	0.09	2.83E-04	31684	7.20E-01
eqtl-a-ENSG00000139531	cML-MA	0.07	0.02	3.88E-05	31684	1.08E+00
eqtl-a-ENSG00000139531	divw	0.08	0.02	3.71E-05	31684	1.08E+00
eqtl-a-ENSG00000139531	IVW	0.08	0.02	2.97E-04	31684	1.08E+00
eqtl-a-ENSG00000139531	raps	0.08	0.02	4.13E-04	31684	1.08E+00
eqtl-a-ENSG00000139531	conmix	0.08	0.02	1.34E-04	31684	1.08E+00
eqtl-a-ENSG00000141499	raps	0.58	0.19	2.25E-03	31684	1.79E+00
eqtl-a-ENSG00000141499	Wald ratio	0.58	0.16	3.27E-04	31684	1.79E+00
eqtl-a-ENSG00000145414	Wald ratio	-0.41	0.09	1.31E-05	31684	6.62E-01
eqtl-a-ENSG00000145414	raps	-0.41	0.11	9.22E-05	31684	6.62E-01
eqtl-a-ENSG00000149573	cML-MA	-0.05	0.01	4.61E-04	31684	9.50E-01
eqtl-a-ENSG00000149573	conmix	-0.09	0.03	3.68E-03	31684	9.16E-01
eqtl-a-ENSG00000149573	raps	-0.06	0.02	7.88E-05	31684	9.37E-01
eqtl-a-ENSG00000149573	divw	-0.06	0.02	1.09E-02	31684	9.41E-01
eqtl-a-ENSG00000149573	IVW	-0.06	0.02	1.76E-04	31684	9.41E-01
eqtl-a-ENSG00000165905	IVW	0.24	0.07	5.17E-04	31684	1.26E+00

eqtl-a-ENSG00000165905	raps	0.24	0.07	9.46E-04	31684	1.26E+00
eqtl-a-ENSG00000165905	divw	0.24	0.07	8.10E-04	31684	1.27E+00
eqtl-a-ENSG00000166037	IVW	-0.09	0.03	2.43E-04	31684	9.11E-01
eqtl-a-ENSG00000166037	conmix	-0.10	0.01	1.02E-13	31684	9.06E-01
eqtl-a-ENSG00000166037	divw	-0.09	0.03	3.31E-04	31684	9.11E-01
eqtl-a-ENSG00000166037	raps	-0.09	0.03	3.71E-04	31684	9.11E-01
eqtl-a-ENSG00000166037	cML-MA	-0.09	0.02	7.19E-09	31684	9.12E-01
eqtl-a-ENSG00000167483	conmix	0.21	0.06	7.88E-04	31684	1.24E+00
eqtl-a-ENSG00000167483	cML-MA	0.16	0.03	8.82E-11	31684	1.18E+00
eqtl-a-ENSG00000167483	raps	0.17	0.05	1.05E-03	31684	1.19E+00
eqtl-a-ENSG00000167483	IVW	0.17	0.05	3.92E-04	31684	1.19E+00
eqtl-a-ENSG00000167483	divw	0.18	0.03	7.58E-08	31684	1.19E+00
eqtl-a-ENSG00000168411	cML-MA	-0.07	0.03	3.02E-03	31684	9.28E-01
eqtl-a-ENSG00000168411	conmix	-0.08	0.03	8.64E-03	31684	9.27E-01
eqtl-a-ENSG00000168411	raps	-0.08	0.02	8.86E-05	31684	9.26E-01
eqtl-a-ENSG00000168411	divw	-0.08	0.02	6.64E-04	31684	9.24E-01
eqtl-a-ENSG00000168411	IVW	-0.08	0.02	5.71E-05	31684	9.24E-01
eqtl-a-ENSG00000171055	conmix	0.05	0.07	0.476393512	31684	1.05E+00
eqtl-a-ENSG00000171055	divw	0.05	0.02	2.37E-02	31684	1.05E+00
eqtl-a-ENSG00000171055	raps	0.05	0.01	6.37E-04	31684	1.05E+00
eqtl-a-ENSG00000171055	IVW	0.05	0.01	4.32E-04	31684	1.05E+00
eqtl-a-ENSG00000171055	cML-MA	0.04	0.01	1.13E-13	31684	1.04E+00
eqtl-a-ENSG00000177508	raps	0.07	0.02	1.01E-03	31684	1.07E+00
eqtl-a-ENSG00000177508	cML-MA	0.07	0.02	3.81E-05	31684	1.07E+00
eqtl-a-ENSG00000177508	IVW	0.07	0.02	4.45E-04	31684	1.07E+00
eqtl-a-ENSG00000177508	divw	0.07	0.01	9.79E-11	31684	1.07E+00

eqtl-a-ENSG00000177508	conmix	0.06	0.01	1.44E-05	31684	1.06E+00
eqtl-a-ENSG00000184056	divw	0.10	0.03	3.94E-03	31684	1.10E+00
eqtl-a-ENSG00000184056	conmix	0.11	0.02	2.07E-07	31684	1.11E+00
eqtl-a-ENSG00000184056	cML-MA	0.10	0.04	7.19E-03	31684	1.10E+00
eqtl-a-ENSG00000184056	raps	0.10	0.02	9.59E-05	31684	1.10E+00
eqtl-a-ENSG00000184056	IVW	0.10	0.03	1.86E-04	31684	1.10E+00
eqtl-a-ENSG00000185361	raps	0.40	0.12	8.14E-04	31684	1.49E+00
eqtl-a-ENSG00000185361	Wald ratio	0.40	0.11	2.15E-04	31684	1.49E+00
eqtl-a-ENSG00000196812	raps	0.23	0.05	2.35E-06	31684	1.26E+00
eqtl-a-ENSG00000196812	divw	0.24	0.08	2.97E-03	31684	1.27E+00
eqtl-a-ENSG00000196812	IVW	0.23	0.05	2.39E-06	31684	1.26E+00
eqtl-a-ENSG00000198518	IVW	0.31	0.05	2.33E-10	31684	1.37E+00
eqtl-a-ENSG00000198518	raps	0.31	0.05	3.90E-09	31684	1.37E+00
ubm-b-789	Wald ratio	-0.93	0.27	5.33E-04	31967	3.95E-01
ubm-b-789	raps	-0.93	0.32	3.72E-03	31967	3.95E-01
ubm-b-289	cML-MA	0.36	0.09	9.60E-05	31968	1.43E+00
ubm-b-289	IVW	0.36	0.10	3.33E-04	31968	1.43E+00
ubm-b-289	conmix	0.41	0.12	6.11E-04	31968	1.51E+00
ubm-b-289	raps	0.36	0.11	7.09E-04	31968	1.43E+00
ubm-b-289	divw	0.36	0.12	3.37E-03	31968	1.44E+00
ubm-b-847	cML-MA	0.46	0.11	6.74E-05	31968	1.58E+00
ubm-b-847	conmix	0.55	0.17	1.30E-03	31968	1.74E+00
ubm-b-847	IVW	0.44	0.13	4.75E-04	31968	1.56E+00
ubm-b-847	divw	0.46	0.12	1.12E-04	31968	1.58E+00
ubm-b-847	raps	0.45	0.14	1.13E-03	31968	1.57E+00
ukb-b-469	divw	1.79	0.47	1.60E-04	33229	5.97E+00

ukb-b-469	raps	1.77	0.26	1.15E-11	33229	5.84E+00
ukb-b-469	IVW	1.76	0.27	4.05E-11	33229	5.82E+00
ukb-b-2732	IVW	-2.08	0.60	5.62E-04	33304	1.25E-01
ukb-b-2732	raps	-1.92	0.58	9.54E-04	33304	1.46E-01
ukb-b-2732	divw	-2.12	0.55	1.10E-04	33304	1.20E-01
ukb-a-328	divw	2.71	1.17	1.99E-02	78291	1.51E+01
ukb-a-328	conmix	2.77	0.80	5.55E-04	78291	1.59E+01
ukb-a-328	IVW	2.69	0.21	1.01E-36	78291	1.47E+01
ukb-a-328	cML-MA	2.49	0.44	2.03E-08	78291	1.21E+01
ukb-a-328	raps	2.60	0.24	2.30E-27	78291	1.35E+01
ukb-a-237	IVW	0.99	0.16	1.80E-10	101726	2.69E+00
ukb-a-237	cML-MA	0.97	0.18	5.28E-08	101726	2.64E+00
ukb-a-237	divw	2.12	0.93	2.20E-02	101726	8.35E+00
ukb-a-237	conmix	0.99	0.30	1.12E-03	101726	2.68E+00
ukb-a-237	raps	1.58	0.33	2.06E-06	101726	4.88E+00
ukb-a-238	divw	1.98	0.41	1.57E-06	101726	7.23E+00
ukb-a-238	conmix	0.95	0.19	8.68E-07	101726	2.59E+00
ukb-a-238	IVW	0.95	0.14	3.04E-12	101726	2.60E+00
ukb-a-238	raps	1.39	0.26	8.29E-08	101726	4.02E+00
ukb-a-238	cML-MA	0.95	0.38	1.15E-02	101726	2.59E+00
ukb-b-6019	divw	2.99	1.35	2.66E-02	108946	1.99E+01
ukb-b-6019	cML-MA	2.72	0.97	5.04E-03	108946	1.52E+01
ukb-b-6019	raps	2.88	0.24	1.31E-32	108946	1.78E+01
ukb-b-6019	IVW	2.97	0.20	1.54E-47	108946	1.94E+01
ukb-b-6019	conmix	3.03	1.40	3.03E-02	108946	2.08E+01
ukb-b-19842	Wald ratio	-5.35	1.08	7.81E-07	113988	4.77E-03

ukb-b-19842	raps	-5.35	1.36	8.54E-05	113988	4.77E-03
finn-b-I9_CARDARR	raps	-0.16	0.05	3.37E-03	118055	8.53E-01
finn-b-I9_CARDARR	Wald ratio	-0.16	0.05	4.29E-04	118055	8.53E-01
ukb-b-10831	raps	2.43	0.48	5.38E-07	142387	1.13E+01
ukb-b-10831	cML-MA	1.42	0.44	1.22E-03	142387	4.12E+00
ukb-b-10831	conmix	1.44	0.40	3.56E-04	142387	4.20E+00
ukb-b-10831	IVW	2.83	0.45	3.12E-10	142387	1.70E+01
ukb-b-10831	divw	2.86	0.77	2.07E-04	142387	1.75E+01
ukb-b-7460	cML-MA	1.30	0.31	3.59E-05	142387	3.66E+00
ukb-b-7460	IVW	1.50	0.29	1.98E-07	142387	4.47E+00
ukb-b-7460	divw	2.46	0.30	2.02E-16	142387	1.17E+01
ukb-b-7460	conmix	1.43	0.48	2.93E-03	142387	4.19E+00
ukb-b-7460	raps	1.95	0.33	4.24E-09	142387	7.03E+00
ukb-a-298	raps	14.59	3.01	1.26E-06	147970	2.18E+06
ukb-a-298	Wald ratio	14.59	1.26	6.17E-31	147970	2.18E+06
ebi-a-GCST90018982	IVW	-0.28	0.07	1.67E-04	153639	7.57E-01
ebi-a-GCST90018982	raps	-0.26	0.08	1.69E-03	153639	7.75E-01
ebi-a-GCST90018982	cML-MA	-0.25	0.09	5.66E-03	153639	7.78E-01
ebi-a-GCST90018982	conmix	-0.46	0.19	1.41E-02	153639	6.32E-01
ebi-a-GCST90018982	divw	-0.28	0.13	3.30E-02	153639	7.53E-01
finn-b-DM_PERIPHATHERO	raps	-0.17	0.04	9.03E-05	168832	8.40E-01
finn-b-DM_PERIPHATHERO	IVW	-0.17	0.04	2.68E-05	168832	8.41E-01
finn-b-DM_PERIPHATHERO	conmix	-0.20	0.07	3.29E-03	168832	8.21E-01
finn-b-DM_PERIPHATHERO	divw	-0.18	0.04	5.53E-05	168832	8.38E-01
finn-b-DM_PERIPHATHERO	cML-MA	-0.18	0.06	5.82E-03	168832	8.38E-01

ukb-b-12405	divw	-0.55	0.23	1.72E-02	170498	5.77E-01
ukb-b-12405	cML-MA	-0.51	0.18	4.59E-03	170498	5.99E-01
ukb-b-12405	IVW	-0.53	0.12	5.02E-06	170498	5.86E-01
ukb-b-12405	conmix	-0.84	0.31	6.31E-03	170498	4.30E-01
ukb-b-12405	raps	-0.54	0.12	9.41E-06	170498	5.86E-01
ebi-a-GCST004622	IVW	-0.09	0.03	1.35E-03	170641	9.16E-01
ebi-a-GCST004622	raps	-0.09	0.03	2.76E-03	170641	9.16E-01
ebi-a-GCST004622	conmix	-0.12	0.04	4.68E-03	170641	8.87E-01
ebi-a-GCST004622	cML-MA	-0.08	0.02	9.86E-06	170641	9.22E-01
ebi-a-GCST004622	divw	-0.10	0.02	2.97E-08	170641	9.01E-01
ebi-a-GCST004604	raps	-0.16	0.04	4.18E-04	173039	8.56E-01
ebi-a-GCST004604	conmix	-0.28	0.07	1.39E-04	173039	7.57E-01
ebi-a-GCST004604	divw	-0.17	0.06	7.19E-03	173039	8.45E-01
ebi-a-GCST004604	cML-MA	-0.16	0.07	2.94E-02	173039	8.56E-01
ebi-a-GCST004604	IVW	-0.15	0.04	3.77E-04	173039	8.63E-01
finn-b-C3_RESPIRATORY_INTRATHORACIC_EXALLC	raps	1.06	0.16	5.08E-11	176059	2.88E+00
finn-b-C3_RESPIRATORY_INTRATHORACIC_EXALLC	Wald ratio	1.06	0.05	2.02E-100	176059	2.88E+00
finn-b-RHEU_ARTHRITIS_OTH	conmix	0.22	0.08	8.15E-03	177943	1.25E+00
finn-b-RHEU_ARTHRITIS_OTH	cML-MA	0.18	0.08	2.66E-02	177943	1.19E+00
finn-b-RHEU_ARTHRITIS_OTH	IVW	0.18	0.05	2.47E-04	177943	1.20E+00

finn-b-RHEU_ARTHRITIS_OTH	raps	0.19	0.06	9.24E-04	177943	1.20E+00
finn-b-RHEU_ARTHRITIS_OTH	divw	0.19	0.12	0.117568985	177943	1.21E+00
ebi-a-GCST90000046	cML-MA	-0.40	0.51	0.4267958	182791	6.69E-01
ebi-a-GCST90000046	divw	-0.42	0.11	8.02E-05	182791	6.58E-01
ebi-a-GCST90000046	conmix	-0.73	0.15	5.89E-07	182791	4.84E-01
ebi-a-GCST90000046	IVW	-0.41	0.09	2.59E-06	182791	6.65E-01
ebi-a-GCST90000046	raps	-0.42	0.09	6.14E-06	182791	6.60E-01
finn-b-DM_NEPHROPATHY_EXMORE	Wald ratio	0.21	0.04	2.47E-08	184987	1.24E+00
finn-b-DM_NEPHROPATHY_EXMORE	raps	0.21	0.04	1.18E-06	184987	1.24E+00
finn-b-I9_REVASC	IVW	-0.08	0.02	1.48E-05	200111	9.26E-01
finn-b-I9_REVASC	conmix	-0.11	0.05	2.61E-02	200111	8.93E-01
finn-b-I9_REVASC	cML-MA	-0.08	0.04	5.23E-02	200111	9.26E-01
finn-b-I9_REVASC	divw	-0.09	0.03	1.01E-03	200111	9.17E-01
finn-b-I9_REVASC	raps	-0.09	0.02	5.72E-05	200111	9.17E-01
finn-b-DM_VITREOUS_BLEEDING	Wald ratio	0.14	0.04	1.50E-04	205549	1.14E+00
finn-b-DM_VITREOUS_BLEEDING	raps	0.14	0.04	6.66E-04	205549	1.14E+00
finn-b-D3_ANAEMIA_B12_DEF	divw	-0.11	0.02	1.55E-07	212822	8.92E-01
finn-b-D3_ANAEMIA_B12_DEF	raps	-0.11	0.03	3.48E-04	212822	8.93E-01

finn-b-D3_ANAEMIA_B12_DEF	IVW	-0.11	0.03	3.96E-04	212822	8.94E-01
finn-b-H7_VITRHAEMORR	raps	0.14	0.04	6.78E-04	213085	1.15E+00
finn-b-H7_VITRHAEMORR	Wald ratio	0.14	0.04	1.50E-04	213085	1.15E+00
ebi-a-GCST90000045	conmix	-1.05	0.11	2.48E-20	214547	3.51E-01
ebi-a-GCST90000045	IVW	-0.60	0.10	8.71E-10	214547	5.47E-01
ebi-a-GCST90000045	cML-MA	-0.57	0.22	1.06E-02	214547	5.64E-01
ebi-a-GCST90000045	divw	-0.62	0.20	2.15E-03	214547	5.38E-01
ebi-a-GCST90000045	raps	-0.61	0.10	7.15E-09	214547	5.45E-01
finn-b-COPD_LATER	Wald ratio	1.54	0.07	3.62E-101	215284	4.68E+00
finn-b-COPD_LATER	raps	1.54	0.28	3.89E-08	215284	4.68E+00
finn-b-AD_LO	raps	-0.04	0.01	4.89E-04	217541	9.65E-01
finn-b-AD_LO	cML-MA	-0.04	0.00	1.79E-13	217541	9.64E-01
finn-b-AD_LO	IVW	-0.04	0.01	2.07E-04	217541	9.64E-01
finn-b-AD_LO	conmix	-0.04	0.01	3.85E-04	217541	9.61E-01
finn-b-AD_LO	divw	-0.04	0.01	8.67E-04	217541	9.64E-01
finn-b-R18_ABNORMAL_FINDI_SECRE_SMEARS_CERVIX_UTERI_VAGINA_VULVA	Wald ratio	0.60	0.15	5.17E-05	217622	1.82E+00
finn-b-R18_ABNORMAL_FINDI_SECRE_SMEARS_CERVIX_UTERI_VAGINA_VULVA	raps	0.60	0.18	7.98E-04	217622	1.82E+00
finn-b-AB1_INFECTIONS	raps	0.77	0.23	1.03E-03	218792	2.16E+00
finn-b-AB1_INFECTIONS	Wald ratio	0.77	0.21	2.37E-04	218792	2.16E+00

finn-b-AB1_VIRAL_SKIN_MUCOUS_MEMBRANE	raps	0.43	0.15	3.33E-03	218792	1.54E+00
finn-b-AB1_VIRAL_SKIN_MUCOUS_MEMBRANE	Wald ratio	0.43	0.12	4.64E-04	218792	1.54E+00
finn-b-C3_RESPIRATORY_INTRATHORACIC	Wald ratio	1.07	0.05	2.02E-100	218792	2.92E+00
finn-b-C3_RESPIRATORY_INTRATHORACIC	raps	1.07	0.16	5.39E-11	218792	2.92E+00
finn-b-COPD_HOSPITAL	raps	0.49	0.13	1.82E-04	218792	1.63E+00
finn-b-COPD_HOSPITAL	Wald ratio	0.49	0.10	7.01E-07	218792	1.63E+00
finn-b-E4_GLUCOPANCREAS	raps	0.16	0.05	4.92E-04	218792	1.17E+00
finn-b-E4_GLUCOPANCREAS	Wald ratio	0.16	0.04	5.47E-05	218792	1.17E+00
finn-b-G6_ALZHEIMER	raps	-0.04	0.01	1.09E-03	218792	9.63E-01
finn-b-G6_ALZHEIMER	cML-MA	-0.04	0.01	5.79E-03	218792	9.63E-01
finn-b-G6_ALZHEIMER	conmix	-0.04	0.01	8.44E-05	218792	9.60E-01
finn-b-G6_ALZHEIMER	IVW	-0.04	0.01	5.69E-04	218792	9.63E-01
finn-b-J10_COPDNAS_INCLAVO	raps	0.51	0.14	2.02E-04	218792	1.67E+00
finn-b-J10_COPDNAS_INCLAVO	Wald ratio	0.51	0.10	7.01E-07	218792	1.67E+00

finn-b-R18_ABNORMAL_FINDI_EXAMI_OTHER_BODY_FLUIDS_SUBST_TISSU_WO_DIAGNOSIS	Wald ratio	0.55	0.14	1.19E-04	218792	1.74E+00
finn-b-R18_ABNORMAL_FINDI_EXAMI_OTHER_BODY_FLUIDS_SUBST_TISSU_WO_DIAGNOSIS	raps	0.55	0.17	9.60E-04	218792	1.74E+00
ukb-a-505	IVW	-0.82	0.19	2.60E-05	226899	4.41E-01
ukb-a-505	conmix	-1.21	0.18	9.06E-12	226899	2.97E-01
ukb-a-505	divw	-0.84	0.11	1.94E-15	226899	4.31E-01
ukb-a-505	raps	-0.77	0.21	2.72E-04	226899	4.63E-01
ukb-a-505	cML-MA	-0.69	0.17	5.20E-05	226899	5.01E-01
ukb-b-16878	cML-MA	-1.41	0.80	7.79E-02	235645	2.43E-01
ukb-b-16878	raps	-1.56	0.29	5.56E-08	235645	2.10E-01
ukb-b-16878	IVW	-1.54	0.27	1.52E-08	235645	2.13E-01
ukb-b-16878	divw	-1.82	0.70	9.15E-03	235645	1.62E-01
ukb-b-16878	conmix	-1.46	0.58	1.25E-02	235645	2.33E-01
ieu-b-5117	divw	0.38	0.18	4.13E-02	246511	1.46E+00
ieu-b-5117	IVW	0.33	0.09	1.88E-04	246511	1.39E+00
ieu-b-5117	cML-MA	0.34	0.31	0.283652074	246511	1.40E+00
ieu-b-5117	conmix	0.51	0.13	6.62E-05	246511	1.67E+00
ieu-b-5117	raps	0.38	0.09	4.64E-05	246511	1.47E+00
ieu-b-142	conmix	0.96	0.39	1.47E-02	249752	2.60E+00

ieu-b-142	cML-MA	0.88	0.30	3.26E-03	249752	2.42E+00
ieu-b-142	IVW	1.09	0.12	1.12E-20	249752	2.97E+00
ieu-b-142	raps	1.08	0.11	3.59E-23	249752	2.94E+00
ieu-b-142	divw	1.10	0.42	9.43E-03	249752	3.00E+00
ukb-a-217	raps	-5.19	1.46	3.63E-04	259921	5.58E-03
ukb-a-217	IVW	-5.18	1.43	3.03E-04	259921	5.63E-03
ebi-a-GCST006250	cML-MA	-0.22	0.09	1.81E-02	269867	7.99E-01
ebi-a-GCST006250	raps	-0.24	0.06	5.68E-05	269867	7.84E-01
ebi-a-GCST006250	divw	-0.26	0.14	5.39E-02	269867	7.69E-01
ebi-a-GCST006250	IVW	-0.26	0.06	1.02E-05	269867	7.74E-01
ebi-a-GCST006250	conmix	-0.52	0.10	6.25E-07	269867	5.94E-01
ukb-a-201	cML-MA	-1.39	0.55	1.11E-02	292053	2.48E-01
ukb-a-201	IVW	-1.48	0.38	1.05E-04	292053	2.27E-01
ukb-a-201	conmix	-1.96	0.46	2.34E-05	292053	1.41E-01
ukb-a-201	divw	-1.51	0.50	2.45E-03	292053	2.22E-01
ukb-a-201	raps	-1.41	0.43	1.16E-03	292053	2.44E-01
ukb-a-202	raps	3.26	0.76	1.97E-05	292053	2.61E+01
ukb-a-202	conmix	3.24	1.16	5.19E-03	292053	2.54E+01
ukb-a-202	divw	3.30	0.88	1.66E-04	292053	2.72E+01
ukb-a-202	IVW	3.24	0.70	3.57E-06	292053	2.55E+01
ukb-a-202	cML-MA	3.19	1.23	9.21E-03	292053	2.43E+01
ukb-a-205	divw	34.10	7.60	7.31E-06	292053	6.45E+14
ukb-a-205	IVW	33.33	6.11	5.01E-08	292053	2.99E+14
ukb-a-205	raps	27.52	5.08	6.02E-08	292053	8.99E+11
ieu-a-1001	raps	-0.68	0.12	2.72E-08	293723	5.08E-01
ieu-a-1001	conmix	-1.33	0.31	2.31E-05	293723	2.64E-01

ieu-a-1001	IVW	-0.70	0.12	1.36E-09	293723	4.95E-01
ieu-a-1001	cML-MA	-0.67	1.42	0.638628274	293723	5.12E-01
ieu-a-1001	divw	-0.72	0.16	6.39E-06	293723	4.86E-01
ebi-a-GCST90014239	Wald ratio	-1.41	0.40	5.06E-04	303612	2.45E-01
ebi-a-GCST90014239	raps	-1.41	0.48	3.56E-03	303612	2.45E-01
ukb-b-6134	divw	-0.82	0.31	9.00E-03	307897	4.39E-01
ukb-b-6134	IVW	-0.80	0.15	1.67E-07	307897	4.49E-01
ukb-b-6134	raps	-0.78	0.16	1.05E-06	307897	4.57E-01
ukb-b-6134	cML-MA	-0.75	0.28	7.53E-03	307897	4.72E-01
ukb-b-6134	conmix	-1.04	0.30	4.91E-04	307897	3.55E-01
ukb-a-5	raps	0.74	0.18	3.26E-05	319740	2.10E+00
ukb-a-5	IVW	0.62	0.16	1.59E-04	319740	1.86E+00
ukb-a-5	conmix	1.05	0.38	5.45E-03	319740	2.85E+00
ukb-a-5	cML-MA	0.68	0.26	9.08E-03	319740	1.98E+00
ukb-a-5	divw	0.74	0.30	1.44E-02	319740	2.09E+00
ukb-b-5174	IVW	1.38	0.25	4.91E-08	327634	3.99E+00
ukb-b-5174	divw	1.41	0.50	5.03E-03	327634	4.11E+00
ukb-b-5174	conmix	1.81	0.49	1.99E-04	327634	6.10E+00
ukb-b-5174	raps	1.33	0.26	3.13E-07	327634	3.77E+00
ukb-b-5174	cML-MA	1.29	0.62	3.77E-02	327634	3.63E+00
ukb-a-40	raps	-34.74	5.66	8.55E-10	328694	8.21E-16
ukb-a-40	Wald ratio	-34.74	1.65	5.37E-98	328694	8.21E-16
ukb-a-265	conmix	0.26	0.07	4.72E-04	330762	1.29E+00
ukb-a-265	raps	0.22	0.05	1.74E-05	330762	1.24E+00

ukb-a-265	IVW	0.23	0.05	2.59E-06	330762	1.26E+00
ukb-a-265	divw	0.24	0.07	2.95E-04	330762	1.27E+00
ukb-a-265	cML-MA	0.21	0.02	1.51E-25	330762	1.23E+00
ukb-a-291	IVW	0.19	0.05	3.58E-05	331093	1.21E+00
ukb-a-291	raps	0.19	0.05	9.97E-05	331093	1.21E+00
ukb-a-291	cML-MA	0.18	0.05	7.32E-04	331093	1.20E+00
ukb-a-291	divw	0.21	0.06	4.77E-04	331093	1.24E+00
ukb-a-291	conmix	0.26	0.07	2.77E-04	331093	1.29E+00
ukb-a-264	raps	0.29	0.07	8.52E-05	331117	1.33E+00
ukb-a-264	divw	0.28	0.04	1.12E-10	331117	1.32E+00
ukb-a-264	conmix	0.83	0.15	1.23E-08	331117	2.30E+00
ukb-a-264	cML-MA	0.27	0.06	2.26E-06	331117	1.30E+00
ukb-a-264	IVW	0.27	0.07	9.19E-05	331117	1.31E+00
ukb-a-287	conmix	0.37	0.04	3.18E-18	331164	1.44E+00
ukb-a-287	divw	0.21	0.02	4.04E-22	331164	1.23E+00
ukb-a-287	IVW	0.20	0.05	2.13E-05	331164	1.23E+00
ukb-a-287	cML-MA	0.19	0.03	7.25E-13	331164	1.21E+00
ukb-a-287	raps	0.19	0.05	1.06E-04	331164	1.21E+00
ukb-a-286	divw	0.27	0.07	1.48E-04	331198	1.31E+00
ukb-a-286	IVW	0.27	0.07	1.20E-04	331198	1.31E+00
ukb-a-286	cML-MA	0.28	0.06	1.09E-05	331198	1.33E+00
ukb-a-286	conmix	0.80	0.16	2.37E-07	331198	2.23E+00
ukb-a-286	raps	0.28	0.07	1.03E-04	331198	1.32E+00
ukb-a-284	cML-MA	0.15	0.02	3.87E-14	331221	1.16E+00
ukb-a-284	conmix	0.27	0.04	7.70E-14	331221	1.31E+00

ukb-a-284	divw	0.20	0.05	9.47E-05	331221	1.22E+00
ukb-a-284	raps	0.15	0.06	1.32E-02	331221	1.16E+00
ukb-a-284	IVW	0.19	0.06	8.72E-04	331221	1.21E+00
ukb-a-283	conmix	0.44	0.12	1.73E-04	331226	1.55E+00
ukb-a-283	cML-MA	0.20	0.04	1.17E-05	331226	1.22E+00
ukb-a-283	raps	0.21	0.05	3.14E-05	331226	1.23E+00
ukb-a-283	IVW	0.22	0.05	4.38E-06	331226	1.25E+00
ukb-a-283	divw	0.23	0.06	4.95E-05	331226	1.26E+00
ukb-a-282	cML-MA	0.36	0.08	2.21E-06	331249	1.43E+00
ukb-a-282	raps	0.35	0.07	1.10E-06	331249	1.42E+00
ukb-a-282	IVW	0.34	0.07	1.33E-06	331249	1.40E+00
ukb-a-282	conmix	0.92	0.11	3.67E-17	331249	2.52E+00
ukb-a-282	divw	0.35	0.06	1.75E-09	331249	1.41E+00
ukb-a-281	IVW	0.20	0.05	2.78E-04	331253	1.22E+00
ukb-a-281	divw	0.20	0.02	4.90E-30	331253	1.22E+00
ukb-a-281	cML-MA	0.16	0.04	1.22E-04	331253	1.17E+00
ukb-a-281	conmix	0.06	0.01	1.15E-09	331253	1.06E+00
ukb-a-281	raps	0.16	0.06	4.50E-03	331253	1.17E+00
ukb-a-279	IVW	0.33	0.06	9.15E-08	331275	1.39E+00
ukb-a-279	raps	0.32	0.06	1.73E-07	331275	1.38E+00
ukb-a-279	cML-MA	0.32	0.08	1.45E-04	331275	1.37E+00
ukb-a-279	divw	0.34	0.07	1.50E-06	331275	1.40E+00
ukb-a-279	conmix	0.57	0.17	5.97E-04	331275	1.77E+00
ukb-a-278	IVW	0.46	0.09	9.93E-08	331278	1.58E+00
ukb-a-278	cML-MA	0.51	0.14	1.52E-04	331278	1.67E+00
ukb-a-278	raps	0.49	0.09	5.47E-08	331278	1.64E+00

ukb-a-278	conmix	0.75	0.20	2.07E-04	331278	2.12E+00
ukb-a-278	divw	0.49	0.14	5.35E-04	331278	1.63E+00
ukb-a-272	cML-MA	-0.22	0.05	3.48E-05	331279	8.02E-01
ukb-a-272	divw	-0.26	0.07	3.04E-04	331279	7.71E-01
ukb-a-272	IVW	-0.24	0.05	8.91E-06	331279	7.84E-01
ukb-a-272	conmix	-0.30	0.08	8.99E-05	331279	7.43E-01
ukb-a-272	raps	-0.23	0.06	7.38E-05	331279	7.95E-01
ukb-a-277	cML-MA	0.16	0.03	1.98E-06	331285	1.17E+00
ukb-a-277	IVW	0.21	0.06	2.03E-04	331285	1.23E+00
ukb-a-277	conmix	-0.04	0.01	3.74E-04	331285	9.63E-01
ukb-a-277	raps	0.16	0.06	3.67E-03	331285	1.18E+00
ukb-a-277	divw	0.21	0.06	4.96E-04	331285	1.23E+00
ukb-a-273	cML-MA	-0.20	0.05	1.76E-04	331292	8.20E-01
ukb-a-273	divw	-0.24	0.05	3.54E-06	331292	7.86E-01
ukb-a-273	conmix	-0.05	0.01	4.45E-04	331292	9.55E-01
ukb-a-273	raps	-0.22	0.06	1.42E-04	331292	8.03E-01
ukb-a-273	IVW	-0.22	0.05	3.24E-05	331292	7.99E-01
ukb-a-275	raps	0.32	0.06	2.34E-07	331293	1.37E+00
ukb-a-275	conmix	0.52	0.08	1.25E-09	331293	1.68E+00
ukb-a-275	divw	0.33	0.07	5.56E-06	331293	1.39E+00
ukb-a-275	cML-MA	0.31	0.08	8.90E-05	331293	1.37E+00
ukb-a-275	IVW	0.32	0.06	1.77E-07	331293	1.38E+00
ukb-a-274	cML-MA	0.43	0.10	1.03E-05	331296	1.53E+00
ukb-a-274	IVW	0.43	0.09	6.30E-07	331296	1.53E+00
ukb-a-274	divw	0.44	0.07	7.99E-10	331296	1.55E+00
ukb-a-274	conmix	0.69	0.14	4.09E-07	331296	1.98E+00

ukb-a-274	raps	0.44	0.09	6.66E-07	331296	1.56E+00
ukb-a-397	cML-MA	-0.96	0.37	8.49E-03	334070	3.82E-01
ukb-a-397	raps	-1.03	0.14	7.43E-13	334070	3.58E-01
ukb-a-397	conmix	-1.57	0.42	2.02E-04	334070	2.08E-01
ukb-a-397	divw	-1.11	0.22	2.83E-07	334070	3.28E-01
ukb-a-397	IVW	-1.09	0.14	1.98E-14	334070	3.37E-01
ukb-a-398	cML-MA	1.59	0.41	1.16E-04	334070	4.89E+00
ukb-a-398	IVW	1.67	0.28	4.31E-09	334070	5.31E+00
ukb-a-398	divw	1.71	0.75	2.25E-02	334070	5.53E+00
ukb-a-398	raps	1.67	0.30	3.36E-08	334070	5.30E+00
ukb-a-398	conmix	2.75	1.03	7.75E-03	334070	1.56E+01
ukb-a-399	raps	-1.63	0.28	4.02E-09	334070	1.97E-01
ukb-a-399	conmix	-2.43	1.04	1.93E-02	334070	8.82E-02
ukb-a-399	IVW	-1.57	0.26	1.80E-09	334070	2.08E-01
ukb-a-399	divw	-1.61	0.66	1.49E-02	334070	2.00E-01
ukb-a-399	cML-MA	-1.60	0.51	1.88E-03	334070	2.02E-01
ukb-a-248	IVW	0.23	0.05	5.81E-07	336107	1.25E+00
ukb-a-248	conmix	0.40	0.11	2.42E-04	336107	1.49E+00
ukb-a-248	divw	0.23	0.07	4.60E-04	336107	1.26E+00
ukb-a-248	raps	0.23	0.05	1.05E-06	336107	1.26E+00
ukb-a-248	cML-MA	0.21	0.06	4.46E-04	336107	1.23E+00
ukb-a-249	cML-MA	0.14	0.04	2.33E-04	336227	1.15E+00
ukb-a-249	conmix	0.22	0.06	4.58E-04	336227	1.24E+00
ukb-a-249	raps	0.15	0.04	8.39E-04	336227	1.16E+00
ukb-a-249	IVW	0.18	0.04	3.32E-05	336227	1.20E+00
ukb-a-249	divw	0.18	0.05	1.17E-04	336227	1.20E+00

ukb-a-382	raps	0.21	0.06	5.94E-04	336639	1.23E+00
ukb-a-382	divw	0.21	0.07	1.31E-03	336639	1.24E+00
ukb-a-382	cML-MA	0.21	0.30	0.487740507	336639	1.23E+00
ukb-a-382	IVW	0.21	0.06	3.14E-04	336639	1.23E+00
ukb-a-382	conmix	0.44	0.13	6.18E-04	336639	1.56E+00
ukb-a-434	IVW	-5.38	1.50	3.41E-04	336683	4.61E-03
ukb-a-434	raps	-5.88	1.63	3.11E-04	336683	2.80E-03
ukb-a-434	cML-MA	-5.40	2.03	7.64E-03	336683	4.50E-03
ukb-a-434	conmix	-6.70	2.32	3.80E-03	336683	1.23E-03
ukb-a-434	divw	-5.47	2.42	2.40E-02	336683	4.22E-03
ukb-a-132	IVW	-3.45	0.95	2.86E-04	337159	3.19E-02
ukb-a-132	raps	-3.46	1.01	5.77E-04	337159	3.13E-02
ukb-a-132	cML-MA	-3.41	1.14	2.68E-03	337159	3.30E-02
ukb-a-132	divw	-3.51	0.78	7.66E-06	337159	2.98E-02
ukb-a-132	conmix	-3.45	1.00	5.31E-04	337159	3.18E-02
ukb-a-142	cML-MA	-3.96	1.08	2.53E-04	337159	1.90E-02
ukb-a-142	conmix	-3.29	0.69	1.85E-06	337159	3.73E-02
ukb-a-142	divw	-4.19	1.33	1.61E-03	337159	1.52E-02
ukb-a-142	IVW	-4.15	1.08	1.18E-04	337159	1.58E-02
ukb-a-142	raps	-3.92	1.12	4.56E-04	337159	1.99E-02
ukb-a-180	divw	-24.57	3.80	1.03E-10	337159	2.15E-11
ukb-a-180	raps	-24.25	7.03	5.63E-04	337159	2.95E-11
ukb-a-180	IVW	-23.94	6.90	5.17E-04	337159	3.99E-11
ieu-b-25	IVW	1.09	0.12	1.12E-20	337334	2.97E+00

ieu-b-25	divw	1.10	0.42	8.98E-03	337334	3.00E+00
ieu-b-25	raps	1.08	0.11	3.59E-23	337334	2.94E+00
ieu-b-25	conmix	0.96	0.35	6.58E-03	337334	2.60E+00
ieu-b-25	cML-MA	0.88	0.38	2.12E-02	337334	2.42E+00
ukb-d-1448_4	raps	19.68	3.63	6.09E-08	348424	3.51E+08
ukb-d-1448_4	Wald ratio	19.68	3.18	6.12E-10	348424	3.51E+08
ukb-d-30020_irnt	raps	-0.16	0.05	6.54E-04	350474	8.54E-01
ukb-d-30020_irnt	divw	-0.16	0.05	4.59E-04	350474	8.49E-01
ukb-d-30020_irnt	cML-MA	-0.15	0.04	2.40E-05	350474	8.57E-01
ukb-d-30020_irnt	IVW	-0.14	0.05	1.58E-03	350474	8.67E-01
ukb-d-30020_irnt	conmix	-0.25	0.07	1.73E-04	350474	7.79E-01
ukb-d-20116_0	raps	-1.26	0.22	1.56E-08	359706	2.83E-01
ukb-d-20116_0	cML-MA	-1.19	0.32	2.23E-04	359706	3.05E-01
ukb-d-20116_0	IVW	-1.29	0.21	1.14E-09	359706	2.75E-01
ukb-d-20116_0	conmix	-2.74	6.58	0.677118569	359706	6.46E-02
ukb-d-20116_0	divw	-1.32	0.32	3.07E-05	359706	2.67E-01
ebi-a-GCST90018947	conmix	0.40	0.07	4.29E-08	359983	1.49E+00
ebi-a-GCST90018947	IVW	0.26	0.04	1.12E-09	359983	1.30E+00
ebi-a-GCST90018947	raps	0.27	0.04	1.11E-09	359983	1.31E+00
ebi-a-GCST90018947	cML-MA	0.25	0.05	3.87E-07	359983	1.29E+00
ebi-a-GCST90018947	divw	0.27	0.05	6.40E-07	359983	1.31E+00
ebi-a-GCST90018949	cML-MA	0.15	0.04	5.88E-04	360116	1.16E+00
ebi-a-GCST90018949	divw	0.20	0.05	1.43E-04	360116	1.22E+00
ebi-a-GCST90018949	IVW	0.19	0.04	5.09E-06	360116	1.21E+00
ebi-a-GCST90018949	conmix	0.16	0.02	1.29E-21	360116	1.18E+00

ebi-a-GCST90018949	raps	0.16	0.04	1.84E-04	360116	1.18E+00
ukb-d-I9_PAD	raps	-43.36	13.04	8.82E-04	361194	1.48E-19
ukb-d-I9_PAD	IVW	-43.29	11.80	2.43E-04	361194	1.58E-19
ukb-b-13952	raps	84.58	12.48	1.23E-11	361823	5.42E+36
ukb-b-13952	Wald ratio	84.58	3.95	1.16E-101	361823	5.42E+36
ebi-a-GCST90000047	cML-MA	-0.62	0.06	5.02E-24	397338	5.37E-01
ebi-a-GCST90000047	conmix	-0.86	0.24	3.86E-04	397338	4.23E-01
ebi-a-GCST90000047	divw	-0.67	0.18	2.48E-04	397338	5.12E-01
ebi-a-GCST90000047	IVW	-0.63	0.07	9.73E-18	397338	5.31E-01
ebi-a-GCST90000047	raps	-0.65	0.08	2.04E-17	397338	5.21E-01
ukb-b-7408	cML-MA	-0.59	0.85	0.488919841	397751	5.56E-01
ukb-b-7408	divw	-0.58	0.17	5.81E-04	397751	5.60E-01
ukb-b-7408	raps	-0.59	0.14	1.97E-05	397751	5.54E-01
ukb-b-7408	conmix	-1.06	0.18	1.65E-09	397751	3.46E-01
ukb-b-7408	IVW	-0.56	0.13	2.33E-05	397751	5.68E-01
ukb-b-14521	IVW	29.41	6.38	4.04E-06	401624	5.93E+12
ukb-b-14521	cML-MA	17.06	6.00	4.44E-03	401624	2.58E+07
ukb-b-14521	divw	29.97	9.24	1.19E-03	401624	1.04E+13
ukb-b-14521	conmix	36.26	9.66	1.74E-04	401624	5.56E+15
ukb-b-14521	raps	29.07	6.49	7.45E-06	401624	4.23E+12
ebi-a-GCST90013975	divw	0.29	0.08	5.93E-04	401772	1.33E+00
ebi-a-GCST90013975	raps	0.30	0.06	1.65E-06	401772	1.34E+00
ebi-a-GCST90013975	cML-MA	0.29	0.08	3.86E-04	401772	1.34E+00
ebi-a-GCST90013975	IVW	0.28	0.06	1.84E-06	401772	1.33E+00

ebi-a-GCST90013975	conmix	0.73	0.19	1.59E-04	401772	2.07E+00
ukb-b-15169	raps	3.11	0.67	3.48E-06	402586	2.24E+01
ukb-b-15169	conmix	3.80	1.09	4.73E-04	402586	4.46E+01
ukb-b-15169	cML-MA	3.06	1.20	1.08E-02	402586	2.14E+01
ukb-b-15169	IVW	3.09	0.61	3.55E-07	402586	2.19E+01
ukb-b-15169	divw	3.14	1.26	1.29E-02	402586	2.30E+01
ukb-b-6591	cML-MA	-0.66	0.10	2.57E-11	406457	5.17E-01
ukb-b-6591	conmix	-0.91	0.28	1.09E-03	406457	4.03E-01
ukb-b-6591	raps	-0.69	0.08	1.57E-19	406457	5.02E-01
ukb-b-6591	divw	-0.70	0.12	1.21E-08	406457	4.95E-01
ukb-b-6591	IVW	-0.69	0.08	2.20E-19	406457	5.03E-01
ebi-a-GCST90013922	raps	2.01	0.35	1.03E-08	407521	7.45E+00
ebi-a-GCST90013922	IVW	2.09	0.34	1.04E-09	407521	8.12E+00
ebi-a-GCST90013922	divw	2.14	0.48	8.78E-06	407521	8.46E+00
ebi-a-GCST90013922	conmix	2.83	1.06	7.79E-03	407521	1.69E+01
ebi-a-GCST90013922	cML-MA	1.34	0.37	2.47E-04	407521	3.82E+00
ebi-a-GCST90013972	cML-MA	1.35	0.44	2.19E-03	407521	3.86E+00
ebi-a-GCST90013972	conmix	2.82	0.35	4.31E-16	407521	1.67E+01
ebi-a-GCST90013972	raps	2.02	0.35	7.45E-09	407521	7.52E+00
ebi-a-GCST90013972	divw	2.14	0.45	2.40E-06	407521	8.50E+00
ebi-a-GCST90013972	IVW	2.10	0.34	6.83E-10	407521	8.15E+00
ebi-a-GCST90013870	cML-MA	0.23	0.06	3.42E-05	407609	1.26E+00
ebi-a-GCST90013870	raps	0.25	0.04	9.36E-09	407609	1.28E+00
ebi-a-GCST90013870	conmix	0.40	0.08	2.69E-06	407609	1.49E+00
ebi-a-GCST90013870	divw	0.25	0.06	2.63E-05	407609	1.28E+00
ebi-a-GCST90013870	IVW	0.24	0.04	1.05E-08	407609	1.27E+00

ebi-a-GCST90013974	divw	0.25	0.05	6.50E-07	407609	1.29E+00
ebi-a-GCST90013974	conmix	0.43	0.10	7.58E-06	407609	1.54E+00
ebi-a-GCST90013974	raps	0.25	0.04	4.70E-09	407609	1.29E+00
ebi-a-GCST90013974	IVW	0.25	0.04	7.38E-09	407609	1.28E+00
ebi-a-GCST90013974	cML-MA	0.24	0.06	9.88E-05	407609	1.27E+00
ebi-a-GCST90014020	cML-MA	0.22	0.06	2.31E-04	407661	1.25E+00
ebi-a-GCST90014020	IVW	0.25	0.06	1.11E-05	407661	1.29E+00
ebi-a-GCST90014020	conmix	0.45	0.12	3.10E-04	407661	1.56E+00
ebi-a-GCST90014020	divw	0.26	0.06	4.37E-05	407661	1.29E+00
ebi-a-GCST90014020	raps	0.24	0.06	3.70E-05	407661	1.27E+00
ebi-a-GCST90014021	cML-MA	0.12	0.04	6.71E-04	407662	1.13E+00
ebi-a-GCST90014021	conmix	0.18	0.03	1.17E-07	407662	1.20E+00
ebi-a-GCST90014021	raps	0.14	0.04	1.62E-03	407662	1.15E+00
ebi-a-GCST90014021	IVW	0.17	0.04	1.68E-04	407662	1.18E+00
ebi-a-GCST90014021	divw	0.17	0.05	2.85E-04	407662	1.18E+00
ebi-a-GCST90013879	raps	0.15	0.03	1.89E-06	407746	1.16E+00
ebi-a-GCST90013879	Wald ratio	0.15	0.03	1.70E-09	407746	1.16E+00
ebi-a-GCST90013929	Wald ratio	0.13	0.02	1.70E-09	407746	1.14E+00
ebi-a-GCST90013929	raps	0.13	0.03	3.79E-06	407746	1.14E+00
ebi-a-GCST90096909	cML-MA	-1.01	0.24	3.80E-05	409125	3.66E-01
ebi-a-GCST90096909	conmix	-1.23	0.15	5.76E-17	409125	2.92E-01
ebi-a-GCST90096909	IVW	-1.18	0.33	3.54E-04	409125	3.09E-01
ebi-a-GCST90096909	divw	-1.21	0.21	1.54E-08	409125	3.00E-01
ebi-a-GCST90096909	raps	-1.10	0.34	1.15E-03	409125	3.33E-01
ebi-a-GCST90000048	raps	-0.15	0.03	2.52E-08	418758	8.58E-01

ebi-a-GCST90000048	IVW	-0.15	0.02	1.26E-09	418758	8.62E-01
ebi-a-GCST90000048	cML-MA	-0.15	0.04	7.85E-05	418758	8.62E-01
ebi-a-GCST90000048	conmix	-0.21	0.06	3.45E-04	418758	8.10E-01
ebi-a-GCST90000048	divw	-0.16	0.12	0.158533865	418758	8.50E-01
ukb-b-969	divw	0.99	0.33	3.11E-03	419314	2.69E+00
ukb-b-969	raps	0.84	0.24	6.35E-04	419314	2.31E+00
ukb-b-969	cML-MA	0.60	0.27	2.44E-02	419314	1.82E+00
ukb-b-969	conmix	0.16	0.06	6.30E-03	419314	1.18E+00
ukb-b-969	IVW	0.81	0.22	2.22E-04	419314	2.24E+00
ukb-b-12018	IVW	9.54	2.38	5.99E-05	423692	1.39E+04
ukb-b-12018	raps	9.74	2.66	2.51E-04	423692	1.70E+04
ukb-b-12018	divw	9.85	2.96	8.64E-04	423692	1.89E+04
ukb-b-11188	Wald ratio	33.68	8.78	1.25E-04	424873	4.23E+14
ukb-b-11188	raps	33.68	10.16	9.18E-04	424873	4.23E+14
ukb-b-2134	divw	-0.47	0.08	1.48E-08	424960	6.27E-01
ukb-b-2134	IVW	-0.45	0.10	1.95E-06	424960	6.35E-01
ukb-b-2134	cML-MA	-0.46	0.09	1.86E-07	424960	6.30E-01
ukb-b-2134	conmix	-0.69	0.21	7.48E-04	424960	5.00E-01
ukb-b-2134	raps	-0.49	0.10	3.34E-07	424960	6.15E-01
ebi-a-GCST90025972	raps	-0.09	0.03	8.15E-04	437291	9.10E-01
ebi-a-GCST90025972	cML-MA	-0.10	0.02	3.97E-05	437291	9.07E-01
ebi-a-GCST90025972	conmix	-0.15	0.03	3.84E-06	437291	8.63E-01
ebi-a-GCST90025972	divw	-0.09	0.02	5.46E-09	437291	9.10E-01
ebi-a-GCST90025972	IVW	-0.10	0.03	2.66E-04	437291	9.07E-01
ukb-b-5192	raps	0.77	0.13	1.25E-09	437887	2.16E+00

ukb-b-5192	cML-MA	0.74	1.73	0.66904476	437887	2.10E+00
ukb-b-5192	divw	0.78	0.33	1.93E-02	437887	2.18E+00
ukb-b-5192	conmix	1.03	0.31	1.07E-03	437887	2.79E+00
ukb-b-5192	IVW	0.70	0.12	1.89E-08	437887	2.01E+00
ukb-b-7647	raps	35.31	6.43	3.93E-08	446149	2.16E+15
ukb-b-7647	Wald ratio	35.31	2.99	3.88E-32	446149	2.16E+15
ebi-a-GCST90012794	divw	2.52	0.99	1.08E-02	451097	1.24E+01
ebi-a-GCST90012794	IVW	2.45	0.53	3.51E-06	451097	1.16E+01
ebi-a-GCST90012794	cML-MA	2.32	1.08	3.24E-02	451097	1.01E+01
ebi-a-GCST90012794	conmix	4.35	2.13	4.16E-02	451097	7.73E+01
ebi-a-GCST90012794	raps	2.37	0.60	6.83E-05	451097	1.07E+01
ieu-b-5118	cML-MA	0.30	0.08	4.38E-04	453169	1.35E+00
ieu-b-5118	IVW	0.34	0.07	1.80E-06	453169	1.41E+00
ieu-b-5118	conmix	0.73	0.19	9.51E-05	453169	2.08E+00
ieu-b-5118	raps	0.34	0.07	3.27E-06	453169	1.40E+00
ieu-b-5118	divw	0.35	0.09	1.98E-04	453169	1.42E+00
ukb-b-19393	cML-MA	0.25	0.07	3.71E-04	454137	1.28E+00
ukb-b-19393	IVW	0.26	0.04	3.06E-10	454137	1.30E+00
ukb-b-19393	raps	0.25	0.04	2.80E-09	454137	1.29E+00
ukb-b-19393	conmix	0.37	0.05	4.41E-13	454137	1.45E+00
ukb-b-19393	divw	0.27	0.07	1.93E-04	454137	1.30E+00
ukb-b-20044	IVW	0.20	0.04	7.76E-07	454588	1.23E+00
ukb-b-20044	cML-MA	0.18	0.03	3.54E-09	454588	1.20E+00
ukb-b-20044	raps	0.19	0.04	1.31E-05	454588	1.21E+00
ukb-b-20044	divw	0.21	0.05	2.28E-05	454588	1.23E+00

ukb-b-20044	conmix	0.24	0.07	2.91E-04	454588	1.27E+00
ukb-b-16407	cML-MA	0.16	0.02	9.80E-19	454613	1.18E+00
ukb-b-16407	divw	0.19	0.04	1.43E-05	454613	1.21E+00
ukb-b-16407	conmix	0.04	0.00	9.80E-19	454613	1.04E+00
ukb-b-16407	raps	0.17	0.05	1.31E-03	454613	1.19E+00
ukb-b-16407	IVW	0.18	0.05	3.06E-04	454613	1.20E+00
ukb-b-8909	conmix	0.71	0.16	1.13E-05	454633	2.04E+00
ukb-b-8909	IVW	0.31	0.06	4.12E-07	454633	1.36E+00
ukb-b-8909	cML-MA	0.29	0.07	6.60E-05	454633	1.34E+00
ukb-b-8909	divw	0.31	0.05	1.44E-10	454633	1.37E+00
ukb-b-8909	raps	0.30	0.06	1.75E-06	454633	1.35E+00
ukb-b-9093	cML-MA	0.13	0.02	2.48E-08	454655	1.14E+00
ukb-b-9093	IVW	0.18	0.05	4.09E-04	454655	1.20E+00
ukb-b-9093	divw	0.18	0.01	5.45E-46	454655	1.20E+00
ukb-b-9093	raps	0.14	0.05	7.05E-03	454655	1.15E+00
ukb-b-9093	conmix	0.19	0.03	3.61E-09	454655	1.21E+00
ukb-b-19925	conmix	0.10	0.02	2.67E-08	454672	1.11E+00
ukb-b-19925	divw	0.23	0.03	3.37E-19	454672	1.26E+00
ukb-b-19925	IVW	0.23	0.05	6.00E-06	454672	1.25E+00
ukb-b-19925	raps	0.18	0.05	4.02E-04	454672	1.20E+00
ukb-b-19925	cML-MA	0.17	0.03	1.48E-07	454672	1.19E+00
ukb-b-8338	divw	0.29	0.02	4.25E-36	454684	1.34E+00
ukb-b-8338	cML-MA	0.26	0.06	2.18E-05	454684	1.29E+00
ukb-b-8338	conmix	0.39	0.09	1.26E-05	454684	1.48E+00
ukb-b-8338	raps	0.27	0.04	2.41E-10	454684	1.31E+00
ukb-b-8338	IVW	0.28	0.04	1.01E-11	454684	1.33E+00

ukb-b-20188	cML-MA	0.36	0.10	4.08E-04	454724	1.43E+00
ukb-b-20188	conmix	0.79	0.20	1.20E-04	454724	2.20E+00
ukb-b-20188	raps	0.36	0.06	6.94E-09	454724	1.43E+00
ukb-b-20188	divw	0.34	0.10	3.38E-04	454724	1.41E+00
ukb-b-20188	IVW	0.34	0.06	2.26E-08	454724	1.40E+00
ukb-b-16698	conmix	0.27	0.08	3.41E-04	454746	1.31E+00
ukb-b-16698	IVW	0.20	0.05	1.31E-04	454746	1.22E+00
ukb-b-16698	raps	0.16	0.05	2.07E-03	454746	1.18E+00
ukb-b-16698	cML-MA	0.17	0.04	3.38E-06	454746	1.18E+00
ukb-b-16698	divw	0.20	0.02	7.72E-16	454746	1.22E+00
ukb-b-19520	divw	0.21	0.05	6.86E-05	454753	1.23E+00
ukb-b-19520	cML-MA	0.17	0.04	1.82E-06	454753	1.19E+00
ukb-b-19520	IVW	0.21	0.05	6.98E-05	454753	1.23E+00
ukb-b-19520	raps	0.18	0.05	7.98E-04	454753	1.20E+00
ukb-b-19520	conmix	0.22	0.03	1.47E-13	454753	1.25E+00
ukb-b-6704	IVW	0.28	0.04	5.83E-11	454757	1.32E+00
ukb-b-6704	raps	0.26	0.04	1.41E-09	454757	1.30E+00
ukb-b-6704	divw	0.28	0.04	2.89E-10	454757	1.33E+00
ukb-b-6704	cML-MA	0.26	0.04	1.33E-09	454757	1.29E+00
ukb-b-6704	conmix	0.33	0.08	4.41E-05	454757	1.39E+00
ukb-b-12854	divw	0.37	0.11	8.55E-04	454789	1.44E+00
ukb-b-12854	raps	0.39	0.06	1.35E-09	454789	1.47E+00
ukb-b-12854	IVW	0.36	0.06	4.47E-09	454789	1.43E+00
ukb-b-12854	conmix	0.82	0.22	2.65E-04	454789	2.27E+00
ukb-b-12854	cML-MA	0.40	0.08	1.41E-06	454789	1.49E+00
ukb-b-7212	conmix	0.59	0.18	9.19E-04	454823	1.80E+00

ukb-b-7212	cML-MA	0.35	0.08	4.40E-06	454823	1.42E+00
ukb-b-7212	IVW	0.36	0.05	6.09E-12	454823	1.44E+00
ukb-b-7212	raps	0.36	0.05	1.49E-11	454823	1.44E+00
ukb-b-7212	divw	0.37	0.10	1.27E-04	454823	1.45E+00
ukb-b-18377	raps	0.52	0.08	4.93E-11	454826	1.69E+00
ukb-b-18377	conmix	0.95	0.20	1.21E-06	454826	2.58E+00
ukb-b-18377	cML-MA	0.50	0.13	6.72E-05	454826	1.65E+00
ukb-b-18377	divw	0.52	0.14	2.03E-04	454826	1.68E+00
ukb-b-18377	IVW	0.51	0.08	5.96E-11	454826	1.66E+00
ukb-b-19921	conmix	-0.10	0.01	7.42E-14	454840	9.05E-01
ukb-b-19921	divw	-0.17	0.03	8.75E-07	454840	8.47E-01
ukb-b-19921	IVW	-0.16	0.04	1.86E-04	454840	8.50E-01
ukb-b-19921	raps	-0.16	0.04	2.78E-04	454840	8.51E-01
ukb-b-19921	cML-MA	-0.15	0.03	6.85E-06	454840	8.58E-01
ukb-b-18096	cML-MA	0.32	0.06	1.84E-07	454846	1.38E+00
ukb-b-18096	divw	0.36	0.07	3.61E-08	454846	1.43E+00
ukb-b-18096	raps	0.35	0.05	1.70E-10	454846	1.42E+00
ukb-b-18096	IVW	0.35	0.05	4.25E-11	454846	1.42E+00
ukb-b-18096	conmix	0.56	0.15	2.16E-04	454846	1.75E+00
ukb-b-19379	divw	-0.21	0.03	4.76E-11	454850	8.08E-01
ukb-b-19379	cML-MA	-0.16	0.03	1.48E-07	454850	8.50E-01
ukb-b-19379	conmix	0.10	0.03	7.02E-05	454850	1.11E+00
ukb-b-19379	IVW	-0.20	0.05	6.56E-05	454850	8.20E-01
ukb-b-19379	raps	-0.18	0.05	5.43E-04	454850	8.34E-01
ukb-b-20531	raps	0.45	0.08	1.15E-08	454854	1.57E+00

ukb-b-20531	cML-MA	0.43	0.12	2.21E-04	454854	1.53E+00
ukb-b-20531	IVW	0.44	0.08	7.70E-09	454854	1.56E+00
ukb-b-20531	conmix	0.80	0.20	6.18E-05	454854	2.22E+00
ukb-b-20531	divw	0.45	0.11	6.58E-05	454854	1.57E+00
ukb-b-16446	raps	0.11	0.05	1.32E-02	454874	1.12E+00
ukb-b-16446	divw	0.16	0.04	2.03E-05	454874	1.17E+00
ukb-b-16446	IVW	0.16	0.05	5.51E-04	454874	1.17E+00
ukb-b-16446	conmix	0.03	0.00	7.23E-12	454874	1.03E+00
ukb-b-16446	cML-MA	0.11	0.02	6.67E-11	454874	1.12E+00
ukb-b-2303	divw	0.30	0.09	1.01E-03	454884	1.35E+00
ukb-b-2303	IVW	0.29	0.04	1.24E-13	454884	1.34E+00
ukb-b-2303	cML-MA	0.29	0.07	2.40E-05	454884	1.34E+00
ukb-b-2303	conmix	0.51	0.10	1.86E-07	454884	1.67E+00
ukb-b-2303	raps	0.30	0.04	9.16E-14	454884	1.35E+00
ukb-b-14540	divw	0.16	0.03	4.92E-07	454888	1.17E+00
ukb-b-14540	raps	0.12	0.05	1.19E-02	454888	1.13E+00
ukb-b-14540	conmix	0.08	0.01	1.14E-11	454888	1.08E+00
ukb-b-14540	cML-MA	0.12	0.02	3.69E-12	454888	1.13E+00
ukb-b-14540	IVW	0.15	0.05	9.40E-04	454888	1.17E+00
ukb-b-12039	IVW	0.16	0.04	3.81E-05	454893	1.18E+00
ukb-b-12039	cML-MA	0.14	0.02	3.54E-10	454893	1.15E+00
ukb-b-12039	divw	0.17	0.04	1.99E-04	454893	1.18E+00
ukb-b-12039	raps	0.14	0.04	6.57E-04	454893	1.15E+00
ukb-b-12039	conmix	0.18	0.05	7.70E-05	454893	1.20E+00
ebi-a-GCST90025994	conmix	0.36	0.10	3.27E-04	457756	1.43E+00

ebi-a-GCST90025994	cML-MA	0.19	0.04	1.25E-07	457756	1.21E+00
ebi-a-GCST90025994	IVW	0.21	0.04	1.35E-06	457756	1.23E+00
ebi-a-GCST90025994	divw	0.21	0.03	2.41E-14	457756	1.24E+00
ebi-a-GCST90025994	raps	0.21	0.04	2.90E-06	457756	1.23E+00
ukb-b-11615	conmix	-2.64	0.89	2.92E-03	458079	7.15E-02
ukb-b-11615	divw	-1.94	0.59	9.49E-04	458079	1.43E-01
ukb-b-11615	raps	-1.92	0.27	1.33E-12	458079	1.46E-01
ukb-b-11615	IVW	-1.90	0.26	5.42E-13	458079	1.50E-01
ukb-b-11615	cML-MA	-1.86	0.35	9.34E-08	458079	1.55E-01
ukb-b-13799	IVW	-2.31	0.54	1.69E-05	458079	9.90E-02
ukb-b-13799	cML-MA	-2.30	0.85	6.68E-03	458079	1.01E-01
ukb-b-13799	conmix	-3.34	0.53	3.69E-10	458079	3.56E-02
ukb-b-13799	divw	-2.38	0.73	1.16E-03	458079	9.28E-02
ukb-b-13799	raps	-2.31	0.57	5.89E-05	458079	9.95E-02
ukb-b-16489	IVW	-1.25	0.13	9.25E-22	458079	2.86E-01
ukb-b-16489	conmix	-2.17	0.38	8.80E-09	458079	1.14E-01
ukb-b-16489	cML-MA	-1.26	0.19	7.16E-11	458079	2.83E-01
ukb-b-16489	raps	-1.26	0.14	2.23E-20	458079	2.83E-01
ukb-b-16489	divw	-1.28	0.27	2.06E-06	458079	2.77E-01
ukb-b-17729	divw	2.03	0.54	1.67E-04	458079	7.61E+00
ukb-b-17729	raps	1.99	0.32	8.89E-10	458079	7.31E+00
ukb-b-17729	conmix	3.90	2.06	5.78E-02	458079	4.95E+01
ukb-b-17729	cML-MA	1.95	0.37	1.50E-07	458079	6.99E+00
ukb-b-17729	IVW	1.98	0.30	4.75E-11	458079	7.26E+00
ukb-b-6306	conmix	1.04	3.52	0.767362835	460844	2.83E+00

ukb-b-6306	cML-MA	0.81	1.21	0.503346791	460844	2.24E+00
ukb-b-6306	IVW	0.79	0.14	2.83E-08	460844	2.20E+00
ukb-b-6306	raps	0.81	0.15	4.62E-08	460844	2.24E+00
ukb-b-6306	divw	0.81	0.09	1.29E-18	460844	2.25E+00
ukb-b-2399	IVW	-23.00	5.88	9.28E-05	461036	1.03E-10
ukb-b-2399	raps	-22.97	6.66	5.67E-04	461036	1.06E-10
ukb-b-3599	IVW	9.03	2.58	4.78E-04	461046	8.31E+03
ukb-b-3599	divw	9.05	3.01	2.59E-03	461046	8.56E+03
ukb-b-3599	raps	10.02	2.21	5.70E-06	461046	2.26E+04
ukb-b-20261	conmix	2.93	0.68	1.76E-05	461066	1.87E+01
ukb-b-20261	divw	1.01	0.26	7.52E-05	461066	2.76E+00
ukb-b-20261	cML-MA	0.92	0.35	8.11E-03	461066	2.50E+00
ukb-b-20261	raps	1.02	0.30	5.86E-04	461066	2.78E+00
ukb-b-20261	IVW	0.99	0.27	3.05E-04	461066	2.69E+00
ukb-b-1553	raps	-24.22	5.97	5.04E-05	461369	3.03E-11
ukb-b-1553	Wald ratio	-24.22	3.92	6.49E-10	461369	3.03E-11
ukb-b-4667	cML-MA	-2.32	0.64	2.75E-04	461369	9.80E-02
ukb-b-4667	conmix	-2.97	0.82	3.11E-04	461369	5.11E-02
ukb-b-4667	divw	-2.41	0.31	1.30E-14	461369	9.02E-02
ukb-b-4667	IVW	-2.34	0.65	2.91E-04	461369	9.61E-02
ukb-b-4667	raps	-2.38	0.68	4.96E-04	461369	9.23E-02
ukb-b-16019	Wald ratio	37.74	3.20	3.88E-32	461378	2.45E+16
ukb-b-16019	raps	37.74	6.83	3.30E-08	461378	2.45E+16

ebi-a-GCST90029013	IVW	-0.12	0.01	3.82E-22	461457	8.84E-01
ebi-a-GCST90029013	cML-MA	-0.13	0.03	2.93E-04	461457	8.81E-01
ebi-a-GCST90029013	conmix	-0.19	0.04	6.14E-06	461457	8.23E-01
ebi-a-GCST90029013	raps	-0.13	0.01	1.67E-21	461457	8.80E-01
ebi-a-GCST90029013	divw	-0.13	0.03	2.33E-04	461457	8.81E-01
ukb-b-19953	cML-MA	0.30	0.06	9.19E-07	461460	1.35E+00
ukb-b-19953	conmix	0.49	0.12	7.24E-05	461460	1.63E+00
ukb-b-19953	IVW	0.30	0.04	5.78E-14	461460	1.35E+00
ukb-b-19953	raps	0.31	0.04	4.53E-14	461460	1.36E+00
ukb-b-19953	divw	0.30	0.08	3.34E-04	461460	1.36E+00
ukb-b-15590	cML-MA	0.14	0.03	9.52E-08	462117	1.14E+00
ukb-b-15590	divw	0.17	0.04	1.45E-06	462117	1.18E+00
ukb-b-15590	conmix	0.28	0.08	2.85E-04	462117	1.32E+00
ukb-b-15590	IVW	0.17	0.04	6.98E-05	462117	1.18E+00
ukb-b-15590	raps	0.14	0.04	1.04E-03	462117	1.15E+00
ukb-b-9405	cML-MA	0.22	0.06	4.82E-04	462166	1.25E+00
ukb-b-9405	raps	0.25	0.06	1.49E-05	462166	1.28E+00
ukb-b-9405	divw	0.27	0.06	1.12E-05	462166	1.31E+00
ukb-b-9405	IVW	0.27	0.06	1.67E-06	462166	1.31E+00
ukb-b-9405	conmix	0.20	0.05	3.22E-05	462166	1.22E+00
ukb-b-11268	raps	-1.41	0.41	5.04E-04	462933	2.44E-01
ukb-b-11268	conmix	-1.74	0.68	1.09E-02	462933	1.75E-01
ukb-b-11268	cML-MA	-1.39	0.64	2.88E-02	462933	2.49E-01
ukb-b-11268	IVW	-1.40	0.40	4.97E-04	462933	2.46E-01
ukb-b-11268	divw	-1.42	0.61	1.97E-02	462933	2.41E-01
ukb-b-17670	raps	34.28	9.01	1.42E-04	462933	7.69E+14

ukb-b-17670	conmix	35.00	13.78	1.11E-02	462933	1.58E+15
ukb-b-17670	IVW	34.99	9.52	2.37E-04	462933	1.57E+15
ukb-b-17670	divw	35.52	11.66	2.31E-03	462933	2.68E+15
ukb-b-17670	cML-MA	33.13	10.24	1.21E-03	462933	2.46E+14
ukb-b-19698	raps	-35.66	11.45	1.85E-03	462933	3.24E-16
ukb-b-19698	Wald ratio	-35.66	9.97	3.48E-04	462933	3.24E-16
ukb-b-10911	raps	-26.76	9.11	3.31E-03	463010	2.38E-12
ukb-b-10911	Wald ratio	-26.76	7.60	4.29E-04	463010	2.38E-12
ukb-b-14206	Wald ratio	-28.08	8.04	4.74E-04	463010	6.37E-13
ukb-b-14206	raps	-28.08	9.12	2.09E-03	463010	6.37E-13
ukb-b-15797	Wald ratio	-26.88	7.69	4.74E-04	463010	2.11E-12
ukb-b-15797	raps	-26.88	8.78	2.20E-03	463010	2.11E-12
ukb-b-18802	raps	-52.15	16.99	2.14E-03	463010	2.24E-23
ukb-b-18802	Wald ratio	-52.15	14.86	4.49E-04	463010	2.24E-23
ukb-b-2205	raps	-11.64	3.18	2.52E-04	463010	8.81E-06
ukb-b-2205	IVW	-11.64	2.98	9.14E-05	463010	8.80E-06
ukb-b-8184	raps	-20.14	5.61	3.30E-04	463010	1.80E-09
ukb-b-8184	IVW	-20.15	5.08	7.32E-05	463010	1.77E-09

ebi-a-GCST90018848	Wald ratio	-1.53	0.25	1.66E-09	467253	2.16E-01
ebi-a-GCST90018848	raps	-1.53	0.38	4.65E-05	467253	2.16E-01
ebi-a-GCST90029014	conmix	1.83	0.38	1.69E-06	468170	6.26E+00
ebi-a-GCST90029014	IVW	1.37	0.13	1.73E-25	468170	3.93E+00
ebi-a-GCST90029014	divw	1.40	0.25	2.37E-08	468170	4.07E+00
ebi-a-GCST90029014	raps	1.38	0.14	1.03E-23	468170	3.97E+00
ebi-a-GCST90029014	cML-MA	1.33	0.27	1.01E-06	468170	3.76E+00
ebi-a-GCST90029012	conmix	-2.12	1.09	5.24E-02	470941	1.20E-01
ebi-a-GCST90029012	raps	-1.34	0.16	4.75E-17	470941	2.61E-01
ebi-a-GCST90029012	divw	-1.41	0.75	5.84E-02	470941	2.43E-01
ebi-a-GCST90029012	cML-MA	-1.29	0.34	1.16E-04	470941	2.75E-01
ebi-a-GCST90029012	IVW	-1.38	0.16	2.36E-18	470941	2.51E-01
ieu-b-4879	divw	0.49	0.08	3.27E-09	472174	1.64E+00
ieu-b-4879	raps	0.48	0.06	6.73E-15	472174	1.61E+00
ieu-b-4879	IVW	0.49	0.06	3.43E-14	472174	1.63E+00
ieu-b-4879	cML-MA	0.43	0.46	0.347526201	472174	1.54E+00
ieu-b-4879	conmix	0.61	0.42	0.147838579	472174	1.85E+00
ebi-a-GCST90018902	raps	1.52	0.29	2.31E-07	477734	4.56E+00
ebi-a-GCST90018902	Wald ratio	1.52	0.07	5.37E-98	477734	4.56E+00
ebi-a-GCST90018890	cML-MA	-0.22	0.06	1.41E-04	483078	8.00E-01
ebi-a-GCST90018890	conmix	-0.22	0.04	6.79E-07	483078	8.03E-01
ebi-a-GCST90018890	IVW	-0.22	0.05	4.72E-05	483078	8.05E-01
ebi-a-GCST90018890	divw	-0.22	0.07	7.88E-04	483078	8.02E-01
ebi-a-GCST90018890	raps	-0.22	0.06	1.28E-04	483078	8.04E-01
ebi-a-GCST90029007	conmix	0.42	0.06	7.30E-11	532396	1.52E+00

ebi-a-GCST90029007	cML-MA	0.26	0.04	4.90E-11	532396	1.30E+00
ebi-a-GCST90029007	divw	0.28	0.07	4.03E-05	532396	1.32E+00
ebi-a-GCST90029007	IVW	0.27	0.04	5.47E-13	532396	1.32E+00
ebi-a-GCST90029007	raps	0.28	0.04	1.08E-12	532396	1.32E+00
ebi-a-GCST90029025	IVW	0.16	0.05	4.37E-04	534045	1.17E+00
ebi-a-GCST90029025	conmix	0.05	0.01	1.81E-06	534045	1.05E+00
ebi-a-GCST90029025	divw	0.16	0.04	2.26E-04	534045	1.18E+00
ebi-a-GCST90029025	raps	0.12	0.05	7.40E-03	534045	1.13E+00
ebi-a-GCST90029025	cML-MA	0.12	0.03	4.48E-04	534045	1.13E+00
ebi-a-GCST90000050	IVW	-0.13	0.03	1.24E-05	542901	8.76E-01
ebi-a-GCST90000050	cML-MA	-0.13	0.05	4.46E-03	542901	8.79E-01
ebi-a-GCST90000050	divw	-0.14	0.04	3.71E-04	542901	8.73E-01
ebi-a-GCST90000050	conmix	-0.22	0.07	1.41E-03	542901	8.01E-01
ebi-a-GCST90000050	raps	-0.13	0.03	3.74E-05	542901	8.76E-01
ebi-a-GCST90000514	cML-MA	0.30	0.12	1.44E-02	602604	1.35E+00
ebi-a-GCST90000514	divw	0.34	0.08	7.68E-06	602604	1.41E+00
ebi-a-GCST90000514	IVW	0.33	0.06	2.80E-07	602604	1.40E+00
ebi-a-GCST90000514	conmix	0.21	0.04	7.32E-07	602604	1.24E+00
ebi-a-GCST90000514	raps	0.32	0.07	2.04E-06	602604	1.38E+00
ieu-b-4877	raps	0.51	0.07	1.73E-14	607291	1.66E+00
ieu-b-4877	conmix	0.74	0.19	8.85E-05	607291	2.10E+00
ieu-b-4877	IVW	0.47	0.06	4.81E-14	607291	1.60E+00
ieu-b-4877	divw	0.48	0.16	2.99E-03	607291	1.62E+00
ieu-b-4877	cML-MA	0.51	0.20	1.25E-02	607291	1.66E+00
ieu-b-40	divw	0.17	0.03	8.99E-09	681275	1.18E+00
ieu-b-40	IVW	0.16	0.04	1.42E-05	681275	1.18E+00

ieu-b-40	conmix	0.06	0.02	2.88E-04	681275	1.06E+00
ieu-b-40	raps	0.16	0.04	4.36E-05	681275	1.18E+00
ieu-b-40	cML-MA	0.15	0.03	2.61E-08	681275	1.16E+00
ieu-a-1239	raps	-0.68	0.06	8.58E-28	766345	5.05E-01
ieu-a-1239	cML-MA	-0.66	0.16	4.40E-05	766345	5.15E-01
ieu-a-1239	divw	-0.70	0.19	2.58E-04	766345	4.98E-01
ieu-a-1239	IVW	-0.67	0.06	6.31E-30	766345	5.13E-01
ieu-a-1239	conmix	-1.06	0.31	6.99E-04	766345	3.45E-01
prot-c-4964_67_1	raps	0.04	0.01	4.91E-04	3080	1.04E+00
prot-c-4964_67_1	IVW	0.04	0.01	3.14E-04	3080	1.04E+00
prot-c-5102_55_3	raps	-0.15	0.02	1.33E-12	3080	8.57E-01
prot-c-5102_55_3	IVW	-0.15	0.02	5.54E-17	3080	8.57E-01

supplementary Table 5 The MR analysis results of between exposures and LUSC

id.exposure	method	beta	se	pval	sample_size	or
ebi-a-GCST90093332	Wald ratio	-0.62	0.11	1.85E-08	1145	5.37E-01
ebi-a-GCST90093332	raps	-0.62	0.16	1.07E-04	1145	5.37E-01
ebi-a-GCST90093341	raps	-0.56	0.15	2.23E-04	2745	5.70E-01
ebi-a-GCST90093341	Wald ratio	-0.56	0.11	9.43E-07	2745	5.70E-01
prot-a-1347	IVW	-0.31	0.09	3.45E-04	3301	7.32E-01
prot-a-1347	raps	-0.32	0.07	4.97E-06	3301	7.30E-01
prot-a-21	Wald ratio	0.55	0.12	8.17E-06	3301	1.73E+00
prot-a-21	raps	0.55	0.15	3.19E-04	3301	1.73E+00
prot-a-2481	IVW	0.35	0.09	9.79E-05	3301	1.41E+00
prot-a-2481	raps	0.35	0.09	1.10E-04	3301	1.42E+00
prot-a-3203	divw	0.07	0.02	4.70E-04	3301	1.07E+00
prot-a-3203	raps	0.07	0.02	1.15E-03	3301	1.07E+00
prot-a-3203	cML-MA	0.07	0.02	7.03E-04	3301	1.07E+00
prot-a-3203	conmix	0.07	0.03	1.99E-02	3301	1.07E+00
prot-a-3203	IVW	0.07	0.02	4.55E-04	3301	1.07E+00
prot-a-710	divw	-0.10	0.03	3.17E-04	3301	9.09E-01
prot-a-710	IVW	-0.09	0.03	2.93E-04	3301	9.10E-01
prot-a-710	cML-MA	-0.09	0.03	5.06E-04	3301	9.11E-01
prot-a-710	raps	-0.09	0.03	4.47E-04	3301	9.10E-01
prot-a-710	conmix	-0.10	0.04	5.52E-03	3301	9.02E-01
prot-a-746	Wald ratio	-0.47	0.13	2.09E-04	3301	6.24E-01
prot-a-746	raps	-0.47	0.15	1.67E-03	3301	6.24E-01
prot-a-885	raps	0.62	0.20	1.65E-03	3301	1.85E+00
prot-a-885	Wald ratio	0.62	0.15	6.67E-05	3301	1.85E+00
ebi-a-GCST009967	raps	2.43	0.45	5.36E-08	4676	1.13E+01
ebi-a-GCST009967	Wald ratio	2.43	0.18	3.82E-40	4676	1.13E+01
ebi-a-GCST009966	raps	-0.93	0.20	2.11E-06	4772	3.93E-01
ebi-a-GCST009966	Wald ratio	-0.93	0.14	7.18E-11	4772	3.93E-01
ebi-a-GCST009968	raps	1.82	0.31	2.72E-09	4772	6.17E+00
ebi-a-GCST009968	Wald ratio	1.82	0.14	3.82E-40	4772	6.17E+00
eqtl-a-ENSG00000223534	cML-MA	-0.22	0.06	7.65E-05	4994	8.02E-01
eqtl-a-ENSG00000223534	raps	-0.25	0.03	9.65E-14	4994	7.80E-01
eqtl-a-ENSG00000223534	divw	-0.22	0.06	6.34E-05	4994	7.99E-01
eqtl-a-ENSG00000223534	IVW	-0.22	0.06	7.07E-05	4994	8.00E-01
eqtl-a-ENSG00000223534	conmix	-0.28	0.11	7.32E-03	4994	7.53E-01
ebi-a-GCST009970	raps	1.15	0.14	1.64E-16	5185	3.16E+00
ebi-a-GCST009970	Wald ratio	1.15	0.09	8.66E-40	5185	3.16E+00
ebi-a-GCST009971	divw	0.19	0.05	1.40E-04	5185	1.21E+00
ebi-a-GCST009971	conmix	0.22	0.06	7.78E-05	5185	1.25E+00
ebi-a-GCST009971	raps	0.21	0.03	9.71E-16	5185	1.23E+00
ebi-a-GCST009971	IVW	0.21	0.03	4.33E-12	5185	1.23E+00
ebi-a-GCST009971	cML-MA	0.20	0.04	8.51E-06	5185	1.22E+00
eqtl-a-ENSG00000166763	raps	-0.19	0.05	3.07E-04	5502	8.30E-01

eqtl-a-ENSG00000166763	IVW	-0.19	0.05	1.94E-04	5502	8.30E-01
eqtl-a-ENSG00000211785	raps	1.45	0.24	2.92E-09	5502	4.25E+00
eqtl-a-ENSG00000211785	Wald ratio	1.45	0.18	1.72E-16	5502	4.25E+00
eqtl-a-ENSG00000250366	IVW	0.48	0.14	4.85E-04	5502	1.62E+00
eqtl-a-ENSG00000250366	raps	0.48	0.15	1.13E-03	5502	1.62E+00
eqtl-a-ENSG00000260228	cML-MA	-0.19	0.06	9.48E-04	5502	8.25E-01
eqtl-a-ENSG00000260228	IVW	-0.20	0.06	4.58E-04	5502	8.16E-01
eqtl-a-ENSG00000260228	divw	-0.20	0.06	5.13E-04	5502	8.15E-01
eqtl-a-ENSG00000260228	raps	-0.20	0.06	7.15E-04	5502	8.15E-01
eqtl-a-ENSG00000260228	conmix	-0.22	0.09	2.16E-02	5502	8.05E-01
ebi-a-GCST90006924	raps	0.55	0.15	3.91E-04	5915	1.73E+00
ebi-a-GCST90006924	Wald ratio	0.55	0.13	3.89E-05	5915	1.73E+00
ebi-a-GCST90006929	Wald ratio	0.69	0.14	6.31E-07	7595	1.99E+00
ebi-a-GCST90006929	raps	0.69	0.16	1.23E-05	7595	1.99E+00
eqtl-a-ENSG00000229515	divw	-0.16	0.04	2.86E-04	7761	8.56E-01
eqtl-a-ENSG00000229515	IVW	-0.15	0.04	2.68E-04	7761	8.56E-01
eqtl-a-ENSG00000229515	conmix	-0.15	0.07	2.63E-02	7761	8.57E-01
eqtl-a-ENSG00000229515	raps	-0.16	0.04	4.08E-04	7761	8.56E-01
eqtl-a-ENSG00000229515	cML-MA	-0.15	0.04	6.40E-04	7761	8.58E-01
ebi-a-GCST90006921	raps	1.68	0.33	3.51E-07	8735	5.35E+00
ebi-a-GCST90006921	IVW	1.68	0.28	2.10E-09	8735	5.35E+00
ebi-a-GCST90006923	raps	1.65	0.51	1.18E-03	8735	5.21E+00
ebi-a-GCST90006923	Wald ratio	1.65	0.42	8.53E-05	8735	5.21E+00
eqtl-a-ENSG00000155026	raps	0.70	0.22	1.63E-03	8906	2.01E+00
eqtl-a-ENSG00000155026	Wald ratio	0.70	0.20	4.05E-04	8906	2.01E+00
eqtl-a-ENSG00000175164	raps	0.11	0.03	6.54E-04	9188	1.12E+00
eqtl-a-ENSG00000175164	cML-MA	0.11	0.03	7.10E-04	9188	1.12E+00
eqtl-a-ENSG00000175164	IVW	0.11	0.03	4.58E-04	9188	1.12E+00
eqtl-a-ENSG00000175164	conmix	0.11	0.04	2.52E-03	9188	1.12E+00
eqtl-a-ENSG00000175164	divw	0.11	0.03	4.70E-04	9188	1.12E+00
ebi-a-GCST90019476	divw	0.24	0.06	1.83E-04	10708	1.27E+00
ebi-a-GCST90019476	conmix	0.27	0.11	1.39E-02	10708	1.31E+00
ebi-a-GCST90019476	cML-MA	0.23	0.07	4.11E-04	10708	1.26E+00
ebi-a-GCST90019476	raps	0.24	0.07	2.72E-04	10708	1.27E+00
ebi-a-GCST90019476	IVW	0.24	0.06	1.49E-04	10708	1.27E+00
eqtl-a-ENSG00000074657	IVW	-0.27	0.07	1.96E-04	11496	7.63E-01
eqtl-a-ENSG00000074657	raps	-0.27	0.08	3.53E-04	11496	7.63E-01
eqtl-a-ENSG00000074657	divw	-0.27	0.07	2.42E-04	11496	7.61E-01
eqtl-a-ENSG00000074657	cML-MA	-0.27	0.08	3.92E-04	11496	7.65E-01
eqtl-a-ENSG00000074657	conmix	-0.28	0.11	7.28E-03	11496	7.52E-01
eqtl-a-ENSG00000204592	raps	0.78	0.19	4.69E-05	13344	2.19E+00
eqtl-a-ENSG00000204592	Wald ratio	0.78	0.17	3.22E-06	13344	2.19E+00
eqtl-a-ENSG00000243753	raps	0.88	0.22	7.86E-05	13344	2.41E+00
eqtl-a-ENSG00000243753	Wald ratio	0.88	0.19	3.87E-06	13344	2.41E+00
ebi-a-GCST90060467	IVW	-0.73	0.20	2.41E-04	13814	4.82E-01
ebi-a-GCST90060467	raps	-0.73	0.22	7.08E-04	13814	4.82E-01

ebi-a-GCST90060470	raps	-0.75	0.22	7.79E-04	13814	4.71E-01
ebi-a-GCST90060470	IVW	-0.75	0.21	2.64E-04	13814	4.72E-01
eqtl-a-ENSG00000072163	raps	0.24	0.07	2.05E-04	14263	1.28E+00
eqtl-a-ENSG00000072163	cML-MA	0.24	0.07	6.17E-04	14263	1.28E+00
eqtl-a-ENSG00000072163	divw	0.25	0.06	1.39E-04	14263	1.28E+00
eqtl-a-ENSG00000072163	IVW	0.24	0.06	1.16E-04	14263	1.28E+00
eqtl-a-ENSG00000072163	conmix	0.27	0.11	1.16E-02	14263	1.31E+00
eqtl-a-ENSG00000140650	IVW	0.17	0.05	2.87E-04	14263	1.19E+00
eqtl-a-ENSG00000140650	raps	0.17	0.05	4.17E-04	14263	1.19E+00
eqtl-a-ENSG00000140650	cML-MA	0.17	0.05	9.72E-04	14263	1.18E+00
eqtl-a-ENSG00000140650	divw	0.17	0.05	3.10E-04	14263	1.19E+00
eqtl-a-ENSG00000140650	conmix	0.18	0.07	7.08E-03	14263	1.19E+00
eqtl-a-ENSG00000204435	raps	0.44	0.12	2.22E-04	14263	1.55E+00
eqtl-a-ENSG00000204435	Wald ratio	0.44	0.11	8.54E-05	14263	1.55E+00
ukb-a-342	IVW	2.48	0.50	7.20E-07	23205	1.19E+01
ukb-a-342	cML-MA	2.07	0.54	1.35E-04	23205	7.92E+00
ukb-a-342	divw	2.51	0.48	1.86E-07	23205	1.24E+01
ukb-a-342	raps	2.28	0.48	2.51E-06	23205	9.75E+00
ukb-a-342	conmix	2.78	0.54	2.15E-07	23205	1.62E+01
ukb-a-343	IVW	-1.92	0.52	2.13E-04	23265	1.46E-01
ukb-a-343	divw	-1.96	0.45	1.44E-05	23265	1.41E-01
ukb-a-343	raps	-1.80	0.49	2.41E-04	23265	1.65E-01
ebi-a-GCST90016675	cML-MA	-0.47	0.14	8.72E-04	25617	6.26E-01
ebi-a-GCST90016675	conmix	-0.55	0.19	4.18E-03	25617	5.75E-01
ebi-a-GCST90016675	divw	-0.49	0.14	5.37E-04	25617	6.13E-01
ebi-a-GCST90016675	IVW	-0.48	0.14	3.91E-04	25617	6.19E-01
ebi-a-GCST90016675	raps	-0.48	0.14	7.47E-04	25617	6.17E-01
eqtl-a-ENSG00000260276	divw	0.12	0.03	2.49E-04	25690	1.13E+00
eqtl-a-ENSG00000260276	IVW	0.12	0.03	2.40E-04	25690	1.13E+00
eqtl-a-ENSG00000260276	cML-MA	0.12	0.04	1.48E-03	25690	1.12E+00
eqtl-a-ENSG00000260276	raps	0.12	0.03	4.51E-04	25690	1.12E+00
eqtl-a-ENSG00000260276	conmix	0.11	0.04	6.47E-03	25690	1.11E+00
eqtl-a-ENSG00000205978	divw	-0.23	0.06	9.20E-05	26196	7.92E-01
eqtl-a-ENSG00000205978	raps	-0.23	0.06	1.53E-04	26196	7.94E-01
eqtl-a-ENSG00000205978	cML-MA	-0.23	0.06	1.32E-04	26196	7.92E-01
eqtl-a-ENSG00000205978	conmix	-0.24	0.09	9.71E-03	26196	7.89E-01
eqtl-a-ENSG00000205978	IVW	-0.23	0.06	8.00E-05	26196	7.93E-01
eqtl-a-ENSG00000188266	raps	-3.35	0.64	1.38E-07	26395	3.51E-02
eqtl-a-ENSG00000188266	Wald ratio	-3.35	0.26	1.97E-38	26395	3.51E-02
eqtl-a-ENSG00000167004	raps	0.47	0.14	1.18E-03	26609	1.60E+00
eqtl-a-ENSG00000167004	Wald ratio	0.47	0.14	5.39E-04	26609	1.60E+00
eqtl-a-ENSG00000189298	raps	0.20	0.06	5.07E-04	26609	1.23E+00
eqtl-a-ENSG00000189298	IVW	0.20	0.06	3.22E-04	26609	1.23E+00
eqtl-a-ENSG00000063438	IVW	-0.30	0.08	1.62E-04	30935	7.37E-01
eqtl-a-ENSG00000063438	cML-MA	-0.30	0.08	3.58E-04	30935	7.43E-01
eqtl-a-ENSG00000063438	conmix	-0.33	0.15	2.76E-02	30935	7.19E-01

eqtl-a-ENSG00000063438	divw	-0.31	0.08	2.11E-04	30935	7.35E-01
eqtl-a-ENSG00000063438	raps	-0.31	0.08	3.03E-04	30935	7.36E-01
eqtl-a-ENSG00000060709	IVW	0.21	0.06	4.19E-04	31470	1.23E+00
eqtl-a-ENSG00000060709	conmix	0.20	0.08	1.48E-02	31470	1.23E+00
eqtl-a-ENSG00000060709	raps	0.21	0.06	7.10E-04	31470	1.23E+00
eqtl-a-ENSG00000060709	divw	0.21	0.06	4.63E-04	31470	1.23E+00
eqtl-a-ENSG00000060709	cML-MA	0.20	0.06	1.46E-03	31470	1.22E+00
eqtl-a-ENSG00000134193	raps	-0.81	0.18	8.37E-06	31470	4.44E-01
eqtl-a-ENSG00000134193	IVW	-0.80	0.23	3.90E-04	31470	4.49E-01
eqtl-a-ENSG00000145476	IVW	0.10	0.02	1.06E-04	31470	1.10E+00
eqtl-a-ENSG00000145476	divw	0.10	0.02	1.06E-04	31470	1.10E+00
eqtl-a-ENSG00000145476	raps	0.10	0.03	1.04E-04	31470	1.10E+00
eqtl-a-ENSG00000145476	conmix	0.11	0.03	9.01E-04	31470	1.12E+00
eqtl-a-ENSG00000145476	cML-MA	0.09	0.03	4.60E-04	31470	1.10E+00
eqtl-a-ENSG00000041357	divw	0.94	0.08	2.30E-33	31644	2.56E+00
eqtl-a-ENSG00000041357	IVW	0.94	0.07	1.54E-44	31644	2.55E+00
eqtl-a-ENSG00000041357	raps	0.94	0.08	5.42E-32	31644	2.55E+00
eqtl-a-ENSG00000159873	cML-MA	-0.33	0.09	1.18E-04	31644	7.17E-01
eqtl-a-ENSG00000159873	divw	-0.33	0.08	1.95E-05	31644	7.17E-01
eqtl-a-ENSG00000159873	IVW	-0.33	0.09	1.84E-04	31644	7.19E-01
eqtl-a-ENSG00000159873	conmix	-0.39	0.15	9.86E-03	31644	6.77E-01
eqtl-a-ENSG00000159873	raps	-0.35	0.08	4.99E-06	31644	7.04E-01
eqtl-a-ENSG00000163788	raps	-0.40	0.11	3.40E-04	31644	6.73E-01
eqtl-a-ENSG00000163788	Wald ratio	-0.40	0.10	1.56E-04	31644	6.73E-01
eqtl-a-ENSG00000173744	raps	-0.29	0.08	6.19E-04	31644	7.51E-01
eqtl-a-ENSG00000173744	IVW	-0.29	0.08	3.57E-04	31644	7.52E-01
eqtl-a-ENSG00000100450	conmix	0.28	0.10	4.06E-03	31684	1.33E+00
eqtl-a-ENSG00000100450	cML-MA	0.26	0.07	4.72E-04	31684	1.30E+00
eqtl-a-ENSG00000100450	divw	0.27	0.07	2.50E-04	31684	1.31E+00
eqtl-a-ENSG00000100450	raps	0.26	0.07	3.57E-04	31684	1.30E+00
eqtl-a-ENSG00000100450	IVW	0.26	0.07	2.04E-04	31684	1.30E+00
eqtl-a-ENSG00000106305	raps	-0.18	0.05	4.15E-04	31684	8.36E-01
eqtl-a-ENSG00000106305	divw	-0.18	0.05	2.93E-04	31684	8.35E-01
eqtl-a-ENSG00000106305	conmix	-0.18	0.07	5.76E-03	31684	8.35E-01
eqtl-a-ENSG00000106305	cML-MA	-0.19	0.05	1.05E-04	31684	8.29E-01
eqtl-a-ENSG00000106305	IVW	-0.18	0.05	2.70E-04	31684	8.36E-01
eqtl-a-ENSG00000107593	raps	0.17	0.04	1.54E-05	31684	1.18E+00
eqtl-a-ENSG00000107593	IVW	0.17	0.04	8.25E-06	31684	1.18E+00
eqtl-a-ENSG00000107890	cML-MA	0.21	0.05	6.81E-05	31684	1.24E+00
eqtl-a-ENSG00000107890	IVW	0.22	0.05	2.36E-05	31684	1.25E+00
eqtl-a-ENSG00000107890	conmix	0.21	0.10	2.68E-02	31684	1.24E+00
eqtl-a-ENSG00000107890	divw	0.22	0.05	2.16E-06	31684	1.25E+00
eqtl-a-ENSG00000107890	raps	0.22	0.05	1.02E-05	31684	1.25E+00
eqtl-a-ENSG00000108384	cML-MA	0.08	0.02	1.17E-04	31684	1.09E+00
eqtl-a-ENSG00000108384	IVW	0.09	0.02	2.49E-05	31684	1.09E+00
eqtl-a-ENSG00000108384	raps	0.09	0.02	4.07E-05	31684	1.09E+00

eqtl-a-ENSG00000108384	conmix	0.09	0.02	1.50E-04	31684	1.09E+00
eqtl-a-ENSG00000108384	divw	0.09	0.02	2.55E-05	31684	1.09E+00
eqtl-a-ENSG00000111725	IVW	-0.11	0.03	1.55E-04	31684	9.00E-01
eqtl-a-ENSG00000111725	conmix	-0.20	0.06	1.36E-03	31684	8.21E-01
eqtl-a-ENSG00000111725	raps	-0.10	0.03	7.34E-05	31684	9.01E-01
eqtl-a-ENSG00000111725	cML-MA	-0.10	0.03	3.57E-03	31684	9.06E-01
eqtl-a-ENSG00000111725	divw	-0.11	0.03	6.40E-05	31684	9.00E-01
eqtl-a-ENSG00000111906	raps	-0.07	0.02	2.21E-04	31684	9.28E-01
eqtl-a-ENSG00000111906	cML-MA	-0.08	0.02	2.97E-04	31684	9.28E-01
eqtl-a-ENSG00000111906	divw	-0.07	0.02	1.72E-04	31684	9.28E-01
eqtl-a-ENSG00000111906	IVW	-0.07	0.02	1.70E-04	31684	9.29E-01
eqtl-a-ENSG00000111906	conmix	-0.07	0.02	3.02E-04	31684	9.29E-01
eqtl-a-ENSG00000134758	cML-MA	-0.13	0.04	1.33E-03	31684	8.76E-01
eqtl-a-ENSG00000134758	raps	-0.14	0.04	7.49E-04	31684	8.73E-01
eqtl-a-ENSG00000134758	IVW	-0.14	0.04	5.18E-04	31684	8.73E-01
eqtl-a-ENSG00000134758	conmix	-0.13	0.05	1.71E-02	31684	8.78E-01
eqtl-a-ENSG00000134758	divw	-0.14	0.04	5.41E-04	31684	8.73E-01
eqtl-a-ENSG00000137338	Wald ratio	0.39	0.09	3.15E-05	31684	1.47E+00
eqtl-a-ENSG00000137338	raps	0.39	0.10	8.16E-05	31684	1.47E+00
eqtl-a-ENSG00000162736	IVW	0.12	0.03	4.18E-05	31684	1.13E+00
eqtl-a-ENSG00000162736	divw	0.12	0.03	1.52E-04	31684	1.13E+00
eqtl-a-ENSG00000162736	conmix	0.14	0.04	1.21E-03	31684	1.15E+00
eqtl-a-ENSG00000162736	cML-MA	0.12	0.04	1.73E-03	31684	1.13E+00
eqtl-a-ENSG00000162736	raps	0.13	0.03	6.82E-05	31684	1.14E+00
eqtl-a-ENSG00000164961	Wald ratio	0.93	0.23	5.25E-05	31684	2.52E+00
eqtl-a-ENSG00000164961	raps	0.93	0.27	6.09E-04	31684	2.52E+00
eqtl-a-ENSG00000196812	divw	0.38	0.09	4.02E-05	31684	1.46E+00
eqtl-a-ENSG00000196812	raps	0.37	0.11	7.26E-04	31684	1.44E+00
eqtl-a-ENSG00000196812	IVW	0.38	0.11	5.09E-04	31684	1.46E+00
eqtl-a-ENSG00000198518	IVW	0.45	0.08	1.06E-08	31684	1.57E+00
eqtl-a-ENSG00000198518	raps	0.45	0.08	8.23E-08	31684	1.57E+00
eqtl-a-ENSG00000205726	cML-MA	-0.10	0.03	2.12E-03	31684	9.08E-01
eqtl-a-ENSG00000205726	divw	-0.11	0.03	1.84E-04	31684	9.00E-01
eqtl-a-ENSG00000205726	raps	-0.10	0.03	8.16E-04	31684	9.04E-01
eqtl-a-ENSG00000205726	IVW	-0.11	0.03	3.11E-04	31684	9.00E-01
eqtl-a-ENSG00000205726	conmix	-0.10	0.04	1.09E-02	31684	9.09E-01
ubm-b-2149	raps	1.51	0.51	2.95E-03	31688	4.52E+00
ubm-b-2149	Wald ratio	1.51	0.43	4.34E-04	31688	4.52E+00
ubm-b-2246	raps	-1.62	0.55	3.51E-03	31688	1.99E-01
ubm-b-2246	Wald ratio	-1.62	0.46	4.34E-04	31688	1.99E-01
ubm-b-3277	divw	-0.77	0.23	6.85E-04	31688	4.62E-01
ubm-b-3277	raps	-0.76	0.23	9.49E-04	31688	4.67E-01
ubm-b-3277	IVW	-0.75	0.21	3.46E-04	31688	4.71E-01
ubm-b-789	Wald ratio	-1.60	0.43	1.89E-04	31967	2.01E-01
ubm-b-789	raps	-1.60	0.52	2.19E-03	31967	2.01E-01
ubm-b-571	Wald ratio	-1.59	0.41	1.07E-04	31968	2.03E-01

ubm-b-571	raps	-1.59	0.52	2.00E-03	31968	2.03E-01
ubm-b-623	Wald ratio	1.14	0.32	4.50E-04	31968	3.12E+00
ubm-b-623	raps	1.14	0.37	2.09E-03	31968	3.12E+00
ubm-b-687	raps	1.07	0.34	1.37E-03	31968	2.93E+00
ubm-b-687	IVW	1.07	0.30	3.03E-04	31968	2.92E+00
ukb-b-469	raps	1.91	0.43	1.08E-05	33229	6.76E+00
ukb-b-469	IVW	1.92	0.47	3.67E-05	33229	6.83E+00
ukb-b-469	divw	1.95	0.39	7.16E-07	33229	7.03E+00
ukb-b-2732	IVW	-2.14	0.53	4.93E-05	33304	1.17E-01
ukb-b-2732	raps	-2.03	0.51	6.90E-05	33304	1.31E-01
ukb-b-2732	divw	-2.18	0.46	2.28E-06	33304	1.13E-01
ukb-b-1572	IVW	2.42	0.57	1.88E-05	33404	1.12E+01
ukb-b-1572	raps	2.21	0.56	9.15E-05	33404	9.10E+00
ukb-b-1572	divw	2.45	0.49	5.36E-07	33404	1.16E+01
ebi-a-GCST90019017	raps	-0.09	0.02	3.15E-06	44161	9.17E-01
ebi-a-GCST90019017	IVW	-0.08	0.02	1.26E-05	44161	9.25E-01
ebi-a-GCST90019017	divw	-0.08	0.02	1.03E-05	44161	9.24E-01
ebi-a-GCST90019017	cML-MA	-0.08	0.03	1.32E-03	44161	9.21E-01
ebi-a-GCST90019017	conmix	-0.14	0.04	8.59E-05	44161	8.70E-01
ieu-b-104	conmix	0.16	0.20	0.414219	46368	1.18E+00
ieu-b-104	cML-MA	-0.28	0.17	0.10702071	46368	7.56E-01
ieu-b-104	IVW	-0.45	0.13	7.49E-04	46368	6.36E-01
ieu-b-104	raps	-0.46	0.14	1.04E-03	46368	6.30E-01
ieu-b-104	divw	-0.59	0.16	3.07E-04	46368	5.54E-01
ukb-a-328	raps	2.66	0.26	6.71E-24	78291	1.43E+01
ukb-a-328	conmix	2.86	0.61	2.41E-06	78291	1.75E+01
ukb-a-328	IVW	2.68	0.26	8.68E-25	78291	1.45E+01
ukb-a-328	divw	2.70	0.26	4.46E-26	78291	1.49E+01
ukb-a-328	cML-MA	2.59	0.31	4.25E-17	78291	1.33E+01
ukb-a-237	divw	2.26	0.42	9.34E-08	101726	9.58E+00
ukb-a-237	conmix	3.17	1.29	1.38E-02	101726	2.38E+01
ukb-a-237	cML-MA	1.07	0.34	1.38E-03	101726	2.93E+00
ukb-a-237	IVW	2.23	0.41	4.38E-08	101726	9.28E+00
ukb-a-237	raps	2.02	0.44	3.61E-06	101726	7.56E+00
ukb-a-238	IVW	1.35	0.34	6.92E-05	101726	3.85E+00
ukb-a-238	conmix	1.26	0.45	5.09E-03	101726	3.53E+00
ukb-a-238	cML-MA	1.07	0.29	2.33E-04	101726	2.93E+00
ukb-a-238	raps	1.82	0.35	2.91E-07	101726	6.16E+00
ukb-a-238	divw	2.13	0.36	3.09E-09	101726	8.44E+00
ukb-b-4801	Wald ratio	12.43	0.92	2.49E-41	104420	2.51E+05
ukb-b-4801	raps	12.43	2.38	1.71E-07	104420	2.51E+05
finn-b-CD2_BENIGN_LEIOMYOMA_UTERI_EXALLC	cML-MA	-0.13	0.07	5.23E-02	107042	8.74E-01
finn-b-CD2_BENIGN_LEIOMYOMA_UTERI_EXALLC	divw	-0.16	0.05	5.40E-04	107042	8.50E-01
finn-b-CD2_BENIGN_LEIOMYOMA_UTERI_EXALLC	raps	-0.15	0.05	1.88E-03	107042	8.61E-01
finn-b-CD2_BENIGN_LEIOMYOMA_UTERI_EXALLC	conmix	-0.29	0.12	1.29E-02	107042	7.49E-01
finn-b-CD2_BENIGN_LEIOMYOMA_UTERI_EXALLC	IVW	-0.16	0.05	5.28E-04	107042	8.52E-01

ukb-b-6019	IVW	2.90	0.32	4.57E-20	108946	1.82E+01
ukb-b-6019	raps	2.85	0.36	2.51E-15	108946	1.73E+01
ukb-b-6019	conmix	3.22	0.73	1.08E-05	108946	2.51E+01
ukb-b-6019	divw	2.93	0.35	3.49E-17	108946	1.86E+01
ukb-b-6019	cML-MA	2.75	0.41	2.33E-11	108946	1.56E+01
ukb-b-12841	conmix	-4.86	1.54	1.64E-03	112583	7.75E-03
ukb-b-12841	cML-MA	-4.95	1.35	2.36E-04	112583	7.06E-03
ukb-b-12841	IVW	-4.86	1.26	1.13E-04	112583	7.72E-03
ukb-b-12841	raps	-4.90	1.36	3.00E-04	112583	7.42E-03
ukb-b-12841	divw	-4.98	1.35	2.21E-04	112583	6.88E-03
ukb-b-19842	raps	-8.62	2.19	8.31E-05	113988	1.80E-04
ukb-b-19842	Wald ratio	-8.62	1.74	7.33E-07	113988	1.80E-04
finn-b-I9_CARDARR	Wald ratio	-0.30	0.07	2.49E-05	118055	7.38E-01
finn-b-I9_CARDARR	raps	-0.30	0.09	8.98E-04	118055	7.38E-01
finn-b-Z21_SPECIAL_SCREEN_EXAM_OTH_DISEA_DISORD	raps	0.29	0.08	1.24E-04	119765	1.33E+00
finn-b-Z21_SPECIAL_SCREEN_EXAM_OTH_DISEA_DISORD	Wald ratio	0.29	0.06	4.63E-06	119765	1.33E+00
finn-b-CD2_BENIGN_LEIOMYOMA_UTERI	cML-MA	-0.17	0.06	6.40E-03	123579	8.45E-01
finn-b-CD2_BENIGN_LEIOMYOMA_UTERI	conmix	-0.28	0.09	1.14E-03	123579	7.54E-01
finn-b-CD2_BENIGN_LEIOMYOMA_UTERI	divw	-0.18	0.05	6.17E-05	123579	8.33E-01
finn-b-CD2_BENIGN_LEIOMYOMA_UTERI	IVW	-0.18	0.04	5.44E-05	123579	8.36E-01
finn-b-CD2_BENIGN_LEIOMYOMA_UTERI	raps	-0.18	0.05	1.09E-04	123579	8.36E-01
ukb-b-8133	raps	-8.61	1.87	4.21E-06	123894	1.83E-04
ukb-b-8133	IVW	-8.55	2.29	1.93E-04	123894	1.93E-04
ukb-b-10831	raps	2.64	0.42	3.49E-10	142387	1.40E+01
ukb-b-10831	conmix	3.62	1.01	3.34E-04	142387	3.72E+01
ukb-b-10831	cML-MA	1.67	0.39	2.06E-05	142387	5.33E+00
ukb-b-10831	IVW	2.73	0.39	2.20E-12	142387	1.53E+01
ukb-b-10831	divw	2.76	0.39	8.51E-13	142387	1.58E+01
ukb-b-7460	divw	2.56	0.30	2.17E-17	142387	1.30E+01
ukb-b-7460	raps	2.35	0.31	5.00E-14	142387	1.05E+01
ukb-b-7460	cML-MA	1.69	0.35	1.30E-06	142387	5.41E+00
ukb-b-7460	conmix	1.88	0.45	2.67E-05	142387	6.56E+00
ukb-b-7460	IVW	2.53	0.30	8.59E-17	142387	1.25E+01
ukb-a-298	raps	17.44	3.85	5.85E-06	147970	3.75E+07
ukb-a-298	Wald ratio	17.44	2.01	4.12E-18	147970	3.75E+07
ebi-a-GCST90018982	IVW	-0.53	0.12	6.94E-06	153639	5.91E-01
ebi-a-GCST90018982	raps	-0.54	0.13	1.85E-05	153639	5.85E-01
ebi-a-GCST90018982	cML-MA	-0.45	0.15	2.44E-03	153639	6.37E-01
ebi-a-GCST90018982	conmix	-0.82	0.24	4.90E-04	153639	4.39E-01
ebi-a-GCST90018982	divw	-0.54	0.12	5.62E-06	153639	5.85E-01
ebi-a-GCST90018992	IVW	-0.93	0.24	1.15E-04	164520	3.94E-01
ebi-a-GCST90018992	raps	-0.93	0.27	5.61E-04	164520	3.96E-01
ebi-a-GCST90018992	divw	-0.95	0.25	1.56E-04	164520	3.86E-01
finn-b-DM_PERIPHATHERO	divw	-0.34	0.07	1.98E-06	168832	7.10E-01
finn-b-DM_PERIPHATHERO	IVW	-0.34	0.07	3.59E-07	168832	7.15E-01
finn-b-DM_PERIPHATHERO	raps	-0.34	0.07	3.19E-06	168832	7.13E-01

finn-b-DM_PERIPHATHERO	conmix	-0.34	0.09	1.77E-04	168832	7.14E-01
finn-b-DM_PERIPHATHERO	cML-MA	-0.34	0.07	8.60E-07	168832	7.12E-01
ukb-b-12405	conmix	-1.08	0.28	1.17E-04	170498	3.39E-01
ukb-b-12405	IVW	-0.75	0.16	1.36E-06	170498	4.72E-01
ukb-b-12405	cML-MA	-0.72	0.19	2.12E-04	170498	4.88E-01
ukb-b-12405	raps	-0.75	0.16	4.58E-06	170498	4.70E-01
ukb-b-12405	divw	-0.77	0.16	1.70E-06	170498	4.62E-01
ebi-a-GCST004604	IVW	-0.22	0.06	7.08E-04	173039	8.06E-01
ebi-a-GCST004604	cML-MA	-0.21	0.09	1.54E-02	173039	8.12E-01
ebi-a-GCST004604	divw	-0.25	0.07	4.01E-04	173039	7.79E-01
ebi-a-GCST004604	raps	-0.23	0.07	1.29E-03	173039	7.98E-01
ebi-a-GCST004604	conmix	-0.26	0.09	4.32E-03	173039	7.68E-01
finn-b-C3_RESPIRATORY_INTRATHORACIC_EXALLC	Wald ratio	1.06	0.08	5.61E-40	176059	2.88E+00
finn-b-C3_RESPIRATORY_INTRATHORACIC_EXALLC	raps	1.06	0.17	1.05E-09	176059	2.88E+00
finn-b-OTHER_SYSTCON_FG	raps	0.68	0.18	1.27E-04	176980	1.97E+00
finn-b-OTHER_SYSTCON_FG	Wald ratio	0.68	0.14	9.08E-07	176980	1.97E+00
ebi-a-GCST90000046	cML-MA	-0.51	0.15	9.08E-04	182791	6.00E-01
ebi-a-GCST90000046	divw	-0.53	0.13	3.84E-05	182791	5.88E-01
ebi-a-GCST90000046	raps	-0.52	0.14	1.89E-04	182791	5.97E-01
ebi-a-GCST90000046	conmix	-1.05	0.29	2.50E-04	182791	3.49E-01
ebi-a-GCST90000046	IVW	-0.52	0.13	4.39E-05	182791	5.96E-01
finn-b-DM_NEPHROPATHY_EXMORE	Wald ratio	0.28	0.06	4.63E-06	184987	1.32E+00
finn-b-DM_NEPHROPATHY_EXMORE	raps	0.28	0.07	3.64E-05	184987	1.32E+00
finn-b-I9_POSTAMI	Wald ratio	-0.33	0.08	6.71E-05	189274	7.16E-01
finn-b-I9_POSTAMI	raps	-0.33	0.11	1.56E-03	189274	7.16E-01
finn-b-J10_COPDNAS	Wald ratio	1.18	0.09	2.49E-41	193318	3.26E+00
finn-b-J10_COPDNAS	raps	1.18	0.14	9.56E-17	193318	3.26E+00
finn-b-I9_CABG	IVW	-0.14	0.03	9.55E-05	193619	8.73E-01
finn-b-I9_CABG	cML-MA	-0.11	0.04	7.45E-03	193619	8.95E-01
finn-b-I9_CABG	raps	-0.13	0.03	1.33E-04	193619	8.80E-01
finn-b-I9_CABG	conmix	-0.16	0.08	3.83E-02	193619	8.52E-01
finn-b-I9_CABG	divw	-0.14	0.04	1.23E-04	193619	8.71E-01
finn-b-CD2_BENIGN_EXALLC	IVW	-0.32	0.09	3.39E-04	195551	7.25E-01
finn-b-CD2_BENIGN_EXALLC	conmix	-0.53	0.24	2.54E-02	195551	5.86E-01
finn-b-CD2_BENIGN_EXALLC	raps	-0.30	0.09	1.49E-03	195551	7.43E-01
finn-b-CD2_BENIGN_EXALLC	divw	-0.33	0.09	3.37E-04	195551	7.21E-01
finn-b-CD2_BENIGN_EXALLC	cML-MA	-0.27	0.13	4.38E-02	195551	7.64E-01
ukb-a-345	raps	3.96	1.34	3.16E-03	199690	5.24E+01
ukb-a-345	Wald ratio	3.96	1.13	4.34E-04	199690	5.24E+01
finn-b-I9_REVASC	IVW	-0.13	0.03	4.18E-05	200111	8.76E-01
finn-b-I9_REVASC	conmix	-0.23	0.06	4.50E-05	200111	7.95E-01
finn-b-I9_REVASC	cML-MA	-0.12	0.05	9.57E-03	200111	8.83E-01
finn-b-I9_REVASC	raps	-0.13	0.03	4.42E-05	200111	8.76E-01
finn-b-I9_REVASC	divw	-0.14	0.03	4.31E-05	200111	8.74E-01
finn-b-DM_VITREOUS_BLEEDING	Wald ratio	0.30	0.06	2.74E-07	205549	1.35E+00
finn-b-DM_VITREOUS_BLEEDING	raps	0.30	0.07	1.39E-05	205549	1.35E+00

finn-b-I9_ANGINA	IVW	-0.23	0.06	4.72E-05	206008	7.94E-01
finn-b-I9_ANGINA	conmix	-0.37	0.13	4.22E-03	206008	6.89E-01
finn-b-I9_ANGINA	divw	-0.23	0.06	5.93E-05	206008	7.91E-01
finn-b-I9_ANGINA	raps	-0.22	0.06	1.46E-04	206008	8.04E-01
finn-b-I9_ANGINA	cML-MA	-0.19	0.08	1.75E-02	206008	8.25E-01
finn-b-I9_CORATHER	conmix	-0.21	0.08	8.63E-03	211203	8.08E-01
finn-b-I9_CORATHER	raps	-0.18	0.05	2.24E-04	211203	8.34E-01
finn-b-I9_CORATHER	divw	-0.20	0.05	1.24E-04	211203	8.17E-01
finn-b-I9_CORATHER	cML-MA	-0.17	0.06	4.48E-03	211203	8.45E-01
finn-b-I9_CORATHER	IVW	-0.20	0.05	1.78E-04	211203	8.21E-01
finn-b-H7_VITRHAEMORR	Wald ratio	0.30	0.06	2.74E-07	213085	1.36E+00
finn-b-H7_VITRHAEMORR	raps	0.30	0.07	1.45E-05	213085	1.36E+00
ebi-a-GCST90000045	divw	-0.85	0.13	1.11E-10	214547	4.29E-01
ebi-a-GCST90000045	raps	-0.84	0.13	3.80E-10	214547	4.34E-01
ebi-a-GCST90000045	IVW	-0.83	0.13	1.42E-10	214547	4.38E-01
ebi-a-GCST90000045	cML-MA	-0.83	0.17	7.96E-07	214547	4.36E-01
ebi-a-GCST90000045	conmix	-1.18	0.24	6.13E-07	214547	3.07E-01
finn-b-COPD_LATER	raps	1.54	0.30	1.89E-07	215284	4.66E+00
finn-b-COPD_LATER	Wald ratio	1.54	0.12	8.52E-40	215284	4.66E+00
finn-b-F5_DEMENTIA	conmix	-0.07	0.04	8.40E-02	216771	9.33E-01
finn-b-F5_DEMENTIA	divw	-0.08	0.02	3.02E-04	216771	9.23E-01
finn-b-F5_DEMENTIA	IVW	-0.08	0.02	4.98E-04	216771	9.24E-01
finn-b-F5_DEMENTIA	cML-MA	-0.07	0.03	1.17E-02	216771	9.34E-01
finn-b-F5_DEMENTIA	raps	-0.07	0.02	1.44E-03	216771	9.29E-01
finn-b-R18_ABNORMAL_FINDI_SECRE_SMEARS_CERVIX_UTERI_VAGINA_VULVA	raps	0.89	0.28	1.46E-03	217622	2.43E+00
finn-b-R18_ABNORMAL_FINDI_SECRE_SMEARS_CERVIX_UTERI_VAGINA_VULVA	Wald ratio	0.89	0.24	1.64E-04	217622	2.43E+00
finn-b-AB1_VIRAL_HEMOR_FEVER_NOS	Wald ratio	0.25	0.06	7.51E-06	218501	1.29E+00
finn-b-AB1_VIRAL_HEMOR_FEVER_NOS	raps	0.25	0.07	1.44E-04	218501	1.29E+00
finn-b-AB1_ARTHROPOD	raps	0.26	0.07	1.15E-04	218792	1.30E+00
finn-b-AB1_ARTHROPOD	Wald ratio	0.26	0.06	7.51E-06	218792	1.30E+00
finn-b-AB1_INFECTIONS	raps	1.69	0.40	2.88E-05	218792	5.40E+00
finn-b-AB1_INFECTIONS	Wald ratio	1.69	0.34	6.07E-07	218792	5.40E+00
finn-b-C3_RESPIRATORY_INTRATHORACIC	raps	1.07	0.18	1.10E-09	218792	2.92E+00
finn-b-C3_RESPIRATORY_INTRATHORACIC	Wald ratio	1.07	0.08	5.61E-40	218792	2.92E+00
finn-b-E4_GLUCOPANCREAS	raps	0.22	0.07	1.85E-03	218792	1.24E+00
finn-b-E4_GLUCOPANCREAS	Wald ratio	0.22	0.06	4.52E-04	218792	1.24E+00
finn-b-F5_DEMENTIA_INCLAVO	divw	-0.08	0.02	2.71E-04	218792	9.23E-01
finn-b-F5_DEMENTIA_INCLAVO	IVW	-0.08	0.02	3.06E-04	218792	9.23E-01
finn-b-F5_DEMENTIA_INCLAVO	conmix	-0.06	0.04	7.71E-02	218792	9.38E-01
finn-b-F5_DEMENTIA_INCLAVO	raps	-0.07	0.02	1.08E-03	218792	9.29E-01
finn-b-F5_DEMENTIA_INCLAVO	cML-MA	-0.07	0.03	7.83E-03	218792	9.30E-01
finn-b-I9_CABG_EXNONE	divw	-0.15	0.04	4.32E-04	218792	8.64E-01
finn-b-I9_CABG_EXNONE	conmix	-0.19	0.11	8.30E-02	218792	8.23E-01
finn-b-I9_CABG_EXNONE	raps	-0.13	0.04	1.63E-03	218792	8.76E-01
finn-b-I9_CABG_EXNONE	cML-MA	-0.11	0.05	3.60E-02	218792	8.93E-01
finn-b-I9_CABG_EXNONE	IVW	-0.14	0.04	4.15E-04	218792	8.66E-01

finn-b-I9_CORATHER_EXNONE	conmix	-0.13	0.07	8.58E-02	218792	8.79E-01
finn-b-I9_CORATHER_EXNONE	raps	-0.19	0.05	3.03E-04	218792	8.27E-01
finn-b-I9_CORATHER_EXNONE	divw	-0.23	0.06	4.94E-05	218792	7.97E-01
finn-b-I9_CORATHER_EXNONE	cML-MA	-0.17	0.07	1.28E-02	218792	8.44E-01
finn-b-I9_CORATHER_EXNONE	IVW	-0.22	0.06	5.14E-05	218792	8.00E-01
finn-b-J10_COPDNAS_INCLAVO	IVW	1.03	0.28	2.47E-04	218792	2.81E+00
finn-b-J10_COPDNAS_INCLAVO	raps	0.95	0.26	2.49E-04	218792	2.57E+00
finn-b-R18_ABNORMAL_FINDI_EXAMI_OTHER_BODY_FLUIDS_SUBST_TISSU_WO_DIAGNOSIS	Wald ratio	0.87	0.22	1.12E-04	218792	2.38E+00
finn-b-R18_ABNORMAL_FINDI_EXAMI_OTHER_BODY_FLUIDS_SUBST_TISSU_WO_DIAGNOSIS	raps	0.87	0.26	9.28E-04	218792	2.38E+00
ukb-b-16878	raps	-1.97	0.44	8.66E-06	235645	1.40E-01
ukb-b-16878	IVW	-2.12	0.42	4.30E-07	235645	1.20E-01
ukb-b-16878	cML-MA	-1.86	0.55	7.44E-04	235645	1.56E-01
ukb-b-16878	conmix	-2.15	0.60	3.34E-04	235645	1.16E-01
ukb-b-16878	divw	-2.17	0.43	5.82E-07	235645	1.14E-01
ieu-b-5117	conmix	1.08	0.28	1.07E-04	246511	2.93E+00
ieu-b-5117	raps	0.58	0.13	3.91E-06	246511	1.79E+00
ieu-b-5117	cML-MA	0.58	0.15	1.84E-04	246511	1.78E+00
ieu-b-5117	divw	0.55	0.13	1.71E-05	246511	1.73E+00
ieu-b-5117	IVW	0.59	0.12	8.11E-07	246511	1.81E+00
ieu-b-142	raps	1.21	0.12	1.21E-23	249752	3.37E+00
ieu-b-142	conmix	1.34	0.23	4.44E-09	249752	3.83E+00
ieu-b-142	IVW	1.20	0.09	9.18E-40	249752	3.31E+00
ieu-b-142	divw	1.19	0.13	3.80E-20	249752	3.28E+00
ieu-b-142	cML-MA	1.15	0.18	4.99E-11	249752	3.16E+00
ebi-a-GCST90018934	raps	-0.15	0.04	5.39E-04	258718	8.60E-01
ebi-a-GCST90018934	divw	-0.16	0.04	2.48E-04	258718	8.52E-01
ebi-a-GCST90018934	cML-MA	-0.13	0.06	2.94E-02	258718	8.75E-01
ebi-a-GCST90018934	conmix	-0.29	0.09	1.20E-03	258718	7.46E-01
ebi-a-GCST90018934	IVW	-0.16	0.04	3.08E-04	258718	8.55E-01
ebi-a-GCST006250	conmix	-0.74	0.18	3.80E-05	269867	4.75E-01
ebi-a-GCST006250	raps	-0.37	0.10	1.54E-04	269867	6.89E-01
ebi-a-GCST006250	divw	-0.38	0.10	7.79E-05	269867	6.84E-01
ebi-a-GCST006250	cML-MA	-0.36	0.12	2.04E-03	269867	7.01E-01
ebi-a-GCST006250	IVW	-0.37	0.09	7.74E-05	269867	6.90E-01
ukb-b-12687	raps	3.98	1.30	2.24E-03	273111	5.36E+01
ukb-b-12687	Wald ratio	3.98	1.13	4.34E-04	273111	5.36E+01
ukb-a-201	conmix	-4.19	1.52	5.67E-03	292053	1.51E-02
ukb-a-201	IVW	-2.39	0.58	4.06E-05	292053	9.12E-02
ukb-a-201	cML-MA	-1.99	0.74	7.26E-03	292053	1.36E-01
ukb-a-201	divw	-2.44	0.58	2.55E-05	292053	8.75E-02
ukb-a-201	raps	-2.28	0.59	1.08E-04	292053	1.02E-01
ukb-a-202	IVW	5.33	1.01	1.39E-07	292053	2.07E+02
ukb-a-202	raps	5.38	1.12	1.55E-06	292053	2.17E+02
ukb-a-202	cML-MA	5.29	1.25	2.41E-05	292053	1.98E+02

ukb-a-202	conmix	5.95	1.73	5.90E-04	292053	3.85E+02
ukb-a-202	divw	5.45	1.10	6.78E-07	292053	2.32E+02
ukb-a-205	divw	39.20	4.16	4.18E-21	292053	1.05E+17
ukb-a-205	IVW	38.31	2.35	1.32E-59	292053	4.35E+16
ukb-a-205	raps	38.51	4.10	5.76E-21	292053	5.28E+16
ieu-a-1001	IVW	-0.80	0.16	1.29E-06	293723	4.50E-01
ieu-a-1001	raps	-0.81	0.18	5.58E-06	293723	4.43E-01
ieu-a-1001	conmix	-0.75	0.32	1.97E-02	293723	4.73E-01
ieu-a-1001	cML-MA	-0.74	0.22	8.55E-04	293723	4.76E-01
ieu-a-1001	divw	-0.89	0.18	6.70E-07	293723	4.09E-01
ebi-a-GCST90018990	raps	-0.10	0.03	9.32E-04	305582	9.07E-01
ebi-a-GCST90018990	divw	-0.10	0.03	2.35E-04	305582	9.02E-01
ebi-a-GCST90018990	conmix	-0.10	0.04	1.49E-02	305582	9.02E-01
ebi-a-GCST90018990	cML-MA	-0.10	0.03	3.45E-03	305582	9.07E-01
ebi-a-GCST90018990	IVW	-0.10	0.03	2.44E-04	305582	9.03E-01
ukb-b-6134	conmix	-1.83	0.52	4.76E-04	307897	1.60E-01
ukb-b-6134	IVW	-1.10	0.28	1.12E-04	307897	3.34E-01
ukb-b-6134	divw	-1.13	0.29	9.31E-05	307897	3.24E-01
ukb-b-6134	cML-MA	-1.11	0.33	9.51E-04	307897	3.31E-01
ukb-b-6134	raps	-1.15	0.30	1.43E-04	307897	3.15E-01
ebi-a-GCST007432	conmix	-0.38	0.13	3.68E-03	321047	6.85E-01
ebi-a-GCST007432	divw	-0.42	0.10	1.41E-05	321047	6.54E-01
ebi-a-GCST007432	cML-MA	-0.32	0.10	1.36E-03	321047	7.23E-01
ebi-a-GCST007432	raps	-0.35	0.09	7.29E-05	321047	7.06E-01
ebi-a-GCST007432	IVW	-0.33	0.08	3.21E-05	321047	7.16E-01
ukb-a-40	Wald ratio	-34.50	2.66	1.97E-38	328694	1.04E-15
ukb-a-40	raps	-34.50	6.02	1.00E-08	328694	1.04E-15
ukb-a-265	raps	0.31	0.07	3.75E-05	330762	1.36E+00
ukb-a-265	IVW	0.30	0.07	2.14E-05	330762	1.35E+00
ukb-a-265	divw	0.31	0.08	4.06E-05	330762	1.36E+00
ukb-a-265	conmix	0.48	0.15	1.36E-03	330762	1.62E+00
ukb-a-265	cML-MA	0.30	0.09	5.30E-04	330762	1.35E+00
ukb-a-291	divw	0.25	0.07	5.07E-04	331093	1.28E+00
ukb-a-291	raps	0.26	0.07	3.86E-04	331093	1.29E+00
ukb-a-291	conmix	0.41	0.20	4.04E-02	331093	1.50E+00
ukb-a-291	IVW	0.25	0.07	2.21E-04	331093	1.29E+00
ukb-a-291	cML-MA	0.25	0.09	5.37E-03	331093	1.29E+00
ukb-a-264	IVW	0.47	0.10	3.51E-06	331117	1.60E+00
ukb-a-264	raps	0.51	0.11	3.03E-06	331117	1.67E+00
ukb-a-264	divw	0.50	0.11	4.81E-06	331117	1.66E+00
ukb-a-264	cML-MA	0.47	0.13	1.66E-04	331117	1.60E+00
ukb-a-264	conmix	0.61	0.23	6.98E-03	331117	1.84E+00
ukb-a-287	IVW	0.28	0.07	2.74E-05	331164	1.32E+00
ukb-a-287	conmix	0.41	0.24	8.34E-02	331164	1.51E+00
ukb-a-287	cML-MA	0.28	0.09	2.43E-03	331164	1.32E+00
ukb-a-287	divw	0.29	0.07	8.24E-05	331164	1.33E+00

ukb-a-287	raps	0.29	0.07	9.33E-05	331164	1.33E+00
ukb-a-286	raps	0.38	0.11	5.98E-04	331198	1.47E+00
ukb-a-286	cML-MA	0.34	0.13	9.54E-03	331198	1.40E+00
ukb-a-286	IVW	0.34	0.11	1.38E-03	331198	1.40E+00
ukb-a-286	conmix	0.27	0.18	0.12343897	331198	1.31E+00
ukb-a-286	divw	0.39	0.11	4.52E-04	331198	1.48E+00
ukb-a-283	raps	0.28	0.07	1.35E-04	331226	1.33E+00
ukb-a-283	divw	0.28	0.07	1.58E-04	331226	1.32E+00
ukb-a-283	IVW	0.27	0.07	9.18E-05	331226	1.31E+00
ukb-a-283	conmix	0.20	0.13	0.11572779	331226	1.22E+00
ukb-a-283	cML-MA	0.27	0.10	5.49E-03	331226	1.31E+00
ukb-a-282	raps	0.42	0.11	1.92E-04	331249	1.52E+00
ukb-a-282	divw	0.43	0.11	1.44E-04	331249	1.53E+00
ukb-a-282	IVW	0.41	0.10	7.89E-05	331249	1.51E+00
ukb-a-282	cML-MA	0.41	0.14	2.95E-03	331249	1.50E+00
ukb-a-282	conmix	0.57	0.25	2.43E-02	331249	1.77E+00
ukb-a-279	divw	0.45	0.09	7.30E-07	331275	1.57E+00
ukb-a-279	raps	0.46	0.09	4.63E-07	331275	1.59E+00
ukb-a-279	conmix	0.72	0.20	2.32E-04	331275	2.05E+00
ukb-a-279	IVW	0.44	0.09	2.89E-07	331275	1.55E+00
ukb-a-279	cML-MA	0.46	0.11	2.22E-05	331275	1.58E+00
ukb-a-278	raps	0.64	0.13	1.80E-06	331278	1.90E+00
ukb-a-278	divw	0.61	0.13	4.81E-06	331278	1.85E+00
ukb-a-278	IVW	0.60	0.13	1.71E-06	331278	1.82E+00
ukb-a-278	conmix	1.07	0.29	2.30E-04	331278	2.92E+00
ukb-a-278	cML-MA	0.63	0.16	8.27E-05	331278	1.88E+00
ukb-a-275	conmix	0.72	0.19	1.94E-04	331293	2.05E+00
ukb-a-275	raps	0.46	0.09	4.45E-07	331293	1.59E+00
ukb-a-275	cML-MA	0.45	0.11	2.62E-05	331293	1.57E+00
ukb-a-275	IVW	0.44	0.09	3.51E-07	331293	1.55E+00
ukb-a-275	divw	0.45	0.09	8.53E-07	331293	1.56E+00
ukb-a-274	divw	0.58	0.13	9.00E-06	331296	1.79E+00
ukb-a-274	conmix	1.11	0.27	5.60E-05	331296	3.02E+00
ukb-a-274	IVW	0.58	0.12	2.04E-06	331296	1.79E+00
ukb-a-274	raps	0.61	0.13	3.83E-06	331296	1.84E+00
ukb-a-274	cML-MA	0.57	0.16	4.00E-04	331296	1.77E+00
ukb-a-397	IVW	-1.33	0.19	1.48E-12	334070	2.65E-01
ukb-a-397	cML-MA	-1.26	0.24	1.45E-07	334070	2.83E-01
ukb-a-397	divw	-1.45	0.19	7.79E-14	334070	2.34E-01
ukb-a-397	raps	-1.32	0.20	1.80E-11	334070	2.66E-01
ukb-a-397	conmix	-2.15	0.48	6.52E-06	334070	1.16E-01
ukb-a-398	raps	2.73	0.47	5.19E-09	334070	1.53E+01
ukb-a-398	IVW	2.54	0.45	1.95E-08	334070	1.26E+01
ukb-a-398	conmix	4.47	0.89	6.05E-07	334070	8.70E+01
ukb-a-398	divw	2.60	0.46	1.53E-08	334070	1.35E+01
ukb-a-398	cML-MA	2.71	0.59	4.27E-06	334070	1.51E+01

ukb-a-399	divw	-2.30	0.40	9.58E-09	334070	1.00E-01
ukb-a-399	raps	-2.28	0.41	2.58E-08	334070	1.02E-01
ukb-a-399	conmix	-3.25	0.74	1.03E-05	334070	3.89E-02
ukb-a-399	IVW	-2.24	0.39	7.01E-09	334070	1.06E-01
ukb-a-399	cML-MA	-2.26	0.47	1.42E-06	334070	1.05E-01
ukb-a-248	cML-MA	0.31	0.08	1.77E-04	336107	1.36E+00
ukb-a-248	conmix	0.48	0.17	5.17E-03	336107	1.61E+00
ukb-a-248	raps	0.33	0.07	2.01E-06	336107	1.39E+00
ukb-a-248	divw	0.36	0.07	2.02E-07	336107	1.43E+00
ukb-a-248	IVW	0.36	0.06	3.47E-08	336107	1.43E+00
ukb-a-249	divw	0.28	0.07	2.79E-05	336227	1.32E+00
ukb-a-249	conmix	0.24	0.12	3.42E-02	336227	1.28E+00
ukb-a-249	IVW	0.28	0.06	8.43E-06	336227	1.32E+00
ukb-a-249	raps	0.26	0.07	7.41E-05	336227	1.30E+00
ukb-a-249	cML-MA	0.26	0.08	1.38E-03	336227	1.30E+00
ukb-a-382	conmix	0.59	0.22	6.73E-03	336639	1.80E+00
ukb-a-382	divw	0.33	0.09	5.04E-04	336639	1.39E+00
ukb-a-382	IVW	0.32	0.09	2.29E-04	336639	1.38E+00
ukb-a-382	raps	0.32	0.09	5.32E-04	336639	1.38E+00
ukb-a-382	cML-MA	0.29	0.13	2.05E-02	336639	1.34E+00
ukb-a-434	conmix	-18.57	7.90	1.87E-02	336683	8.61E-09
ukb-a-434	raps	-10.98	3.32	9.50E-04	336683	1.71E-05
ukb-a-434	IVW	-10.94	3.08	3.87E-04	336683	1.77E-05
ukb-a-434	divw	-11.14	3.06	2.77E-04	336683	1.46E-05
ukb-a-434	cML-MA	-9.83	3.28	2.74E-03	336683	5.39E-05
ukb-a-132	conmix	-9.40	3.06	2.13E-03	337159	8.23E-05
ukb-a-132	divw	-7.06	1.63	1.42E-05	337159	8.56E-04
ukb-a-132	IVW	-6.92	1.72	5.87E-05	337159	9.84E-04
ukb-a-132	cML-MA	-6.77	1.75	1.15E-04	337159	1.15E-03
ukb-a-132	raps	-6.85	1.89	2.97E-04	337159	1.06E-03
ukb-a-176	conmix	-7.54	2.76	6.33E-03	337159	5.34E-04
ukb-a-176	raps	-6.38	1.71	1.89E-04	337159	1.70E-03
ukb-a-176	divw	-6.54	1.79	2.62E-04	337159	1.44E-03
ukb-a-176	cML-MA	-5.47	1.87	3.48E-03	337159	4.23E-03
ukb-a-176	IVW	-5.45	1.41	1.13E-04	337159	4.31E-03
ieu-b-25	raps	1.21	0.12	1.21E-23	337334	3.37E+00
ieu-b-25	conmix	1.34	0.23	4.44E-09	337334	3.83E+00
ieu-b-25	cML-MA	1.15	0.18	4.99E-11	337334	3.16E+00
ieu-b-25	IVW	1.20	0.09	9.18E-40	337334	3.31E+00
ieu-b-25	divw	1.19	0.13	3.80E-20	337334	3.28E+00
ieu-b-24	IVW	-3.10	0.66	2.44E-06	341427	4.49E-02
ieu-b-24	divw	-3.19	0.75	2.10E-05	341427	4.10E-02
ieu-b-24	raps	-3.11	0.74	2.97E-05	341427	4.46E-02
ebi-a-GCST90104006	conmix	-0.02	0.05	0.65287993	349222	9.79E-01
ebi-a-GCST90104006	raps	-0.11	0.03	2.14E-05	349222	8.95E-01
ebi-a-GCST90104006	divw	-0.12	0.03	4.22E-06	349222	8.85E-01

ebi-a-GCST90104006	IVW	-0.11	0.03	5.97E-06	349222	8.92E-01
ebi-a-GCST90104006	cML-MA	-0.09	0.04	1.02E-02	349222	9.10E-01
ieu-b-5113	conmix	0.16	0.20	0.414219	353315	1.18E+00
ieu-b-5113	raps	-0.46	0.14	1.04E-03	353315	6.30E-01
ieu-b-5113	IVW	-0.45	0.13	7.49E-04	353315	6.36E-01
ieu-b-5113	cML-MA	-0.28	0.17	0.10702071	353315	7.56E-01
ieu-b-5113	divw	-0.59	0.16	3.07E-04	353315	5.54E-01
ukb-d-20116_0	raps	-1.88	0.37	2.79E-07	359706	1.53E-01
ukb-d-20116_0	IVW	-1.87	0.34	3.45E-08	359706	1.54E-01
ukb-d-20116_0	divw	-1.92	0.35	3.01E-08	359706	1.47E-01
ukb-d-20116_0	conmix	-2.64	0.78	6.78E-04	359706	7.14E-02
ukb-d-20116_0	cML-MA	-1.80	0.38	2.47E-06	359706	1.65E-01
ebi-a-GCST90018947	raps	0.39	0.07	9.62E-09	359983	1.47E+00
ebi-a-GCST90018947	divw	0.39	0.07	7.13E-09	359983	1.48E+00
ebi-a-GCST90018947	IVW	0.37	0.06	5.92E-09	359983	1.45E+00
ebi-a-GCST90018947	conmix	0.61	0.14	1.22E-05	359983	1.83E+00
ebi-a-GCST90018947	cML-MA	0.37	0.09	2.95E-05	359983	1.45E+00
ebi-a-GCST90018949	cML-MA	0.25	0.08	1.83E-03	360116	1.28E+00
ebi-a-GCST90018949	conmix	0.24	0.10	1.36E-02	360116	1.27E+00
ebi-a-GCST90018949	IVW	0.26	0.06	4.60E-05	360116	1.30E+00
ebi-a-GCST90018949	divw	0.27	0.07	5.15E-05	360116	1.31E+00
ebi-a-GCST90018949	raps	0.26	0.07	1.21E-04	360116	1.29E+00
ukb-d-COPD_EARLYANDLATER	Wald ratio	212.20	19.52	1.58E-27	361194	1.43E+92
ukb-d-COPD_EARLYANDLATER	raps	212.20	43.06	8.30E-07	361194	1.43E+92
ukb-d-I9_CORATHER	conmix	-9.67	3.29	3.26E-03	361194	6.34E-05
ukb-d-I9_CORATHER	cML-MA	-5.20	1.73	2.67E-03	361194	5.50E-03
ukb-d-I9_CORATHER	divw	-6.17	1.40	1.01E-05	361194	2.09E-03
ukb-d-I9_CORATHER	raps	-5.72	1.43	6.10E-05	361194	3.27E-03
ukb-d-I9_CORATHER	IVW	-6.08	1.39	1.19E-05	361194	2.29E-03
ukb-d-I9_IHD	conmix	-5.83	2.14	6.50E-03	361194	2.92E-03
ukb-d-I9_IHD	raps	-4.97	1.12	8.56E-06	361194	6.96E-03
ukb-d-I9_IHD	divw	-5.23	1.08	1.42E-06	361194	5.36E-03
ukb-d-I9_IHD	IVW	-5.16	1.07	1.47E-06	361194	5.75E-03
ukb-d-I9_IHD	cML-MA	-4.52	1.39	1.17E-03	361194	1.09E-02
ukb-d-III_BLOOD_IMMUN	Wald ratio	45.53	9.74	2.93E-06	361194	5.92E+19
ukb-d-III_BLOOD_IMMUN	raps	45.53	11.67	9.60E-05	361194	5.92E+19
ukb-b-13952	raps	84.15	13.44	3.85E-10	361823	3.51E+36
ukb-b-13952	Wald ratio	84.15	6.37	8.66E-40	361823	3.51E+36
ebi-a-GCST011365	divw	-0.19	0.05	1.39E-04	395795	8.30E-01
ebi-a-GCST011365	conmix	-0.14	0.05	8.53E-03	395795	8.65E-01
ebi-a-GCST011365	cML-MA	-0.12	0.07	7.84E-02	395795	8.84E-01
ebi-a-GCST011365	IVW	-0.17	0.05	3.02E-04	395795	8.48E-01
ebi-a-GCST011365	raps	-0.16	0.05	5.41E-04	395795	8.51E-01
ebi-a-GCST90000047	conmix	-1.15	0.16	2.47E-12	397338	3.17E-01
ebi-a-GCST90000047	raps	-0.88	0.11	1.50E-16	397338	4.17E-01
ebi-a-GCST90000047	divw	-0.86	0.11	7.47E-15	397338	4.24E-01

ebi-a-GCST90000047	cML-MA	-0.88	0.14	1.27E-10	397338	4.14E-01
ebi-a-GCST90000047	IVW	-0.86	0.11	2.64E-16	397338	4.21E-01
ukb-b-17685	cML-MA	4.88	1.38	4.05E-04	397732	1.31E+02
ukb-b-17685	conmix	6.75	3.38	4.59E-02	397732	8.58E+02
ukb-b-17685	IVW	4.77	1.18	5.06E-05	397732	1.17E+02
ukb-b-17685	divw	7.82	2.06	1.50E-04	397732	2.49E+03
ukb-b-17685	raps	6.61	1.76	1.71E-04	397732	7.43E+02
ukb-b-7408	cML-MA	-0.97	0.22	1.29E-05	397751	3.80E-01
ukb-b-7408	raps	-0.99	0.18	6.94E-08	397751	3.72E-01
ukb-b-7408	IVW	-0.99	0.17	3.74E-09	397751	3.71E-01
ukb-b-7408	conmix	-1.59	0.42	1.41E-04	397751	2.04E-01
ukb-b-7408	divw	-1.02	0.17	4.82E-09	397751	3.62E-01
ukb-b-14521	divw	31.52	6.00	1.48E-07	401624	4.89E+13
ukb-b-14521	IVW	30.93	6.68	3.59E-06	401624	2.71E+13
ukb-b-14521	conmix	38.35	10.57	2.87E-04	401624	4.51E+16
ukb-b-14521	cML-MA	22.32	9.78	2.24E-02	401624	4.93E+09
ukb-b-14521	raps	30.52	6.82	7.58E-06	401624	1.80E+13
ebi-a-GCST90013975	IVW	0.44	0.09	6.32E-07	401772	1.55E+00
ebi-a-GCST90013975	cML-MA	0.46	0.12	1.69E-04	401772	1.58E+00
ebi-a-GCST90013975	conmix	0.74	0.22	6.97E-04	401772	2.09E+00
ebi-a-GCST90013975	raps	0.48	0.09	5.42E-07	401772	1.61E+00
ebi-a-GCST90013975	divw	0.45	0.09	1.93E-06	401772	1.56E+00
ukb-b-15169	conmix	5.21	1.54	6.92E-04	402586	1.83E+02
ukb-b-15169	divw	4.73	0.97	1.02E-06	402586	1.13E+02
ukb-b-15169	raps	4.60	1.08	2.10E-05	402586	9.96E+01
ukb-b-15169	IVW	4.64	0.92	4.42E-07	402586	1.04E+02
ukb-b-15169	cML-MA	4.49	1.11	5.07E-05	402586	8.93E+01
ukb-b-6591	IVW	-0.84	0.10	6.42E-18	406457	4.30E-01
ukb-b-6591	cML-MA	-0.85	0.12	5.50E-12	406457	4.26E-01
ukb-b-6591	raps	-0.86	0.10	1.01E-17	406457	4.25E-01
ukb-b-6591	divw	-0.86	0.10	6.43E-18	406457	4.21E-01
ukb-b-6591	conmix	-1.30	0.19	5.87E-12	406457	2.72E-01
ebi-a-GCST90013922	IVW	1.59	0.44	2.65E-04	407521	4.90E+00
ebi-a-GCST90013922	divw	2.07	0.42	8.50E-07	407521	7.94E+00
ebi-a-GCST90013922	raps	1.99	0.45	8.95E-06	407521	7.32E+00
ebi-a-GCST90013922	conmix	1.64	0.53	1.93E-03	407521	5.17E+00
ebi-a-GCST90013922	cML-MA	1.17	0.36	1.09E-03	407521	3.22E+00
ebi-a-GCST90013972	conmix	1.65	0.54	2.01E-03	407521	5.22E+00
ebi-a-GCST90013972	IVW	1.59	0.44	2.75E-04	407521	4.91E+00
ebi-a-GCST90013972	divw	2.07	0.42	7.78E-07	407521	7.96E+00
ebi-a-GCST90013972	raps	2.00	0.45	7.88E-06	407521	7.40E+00
ebi-a-GCST90013972	cML-MA	1.17	0.36	1.18E-03	407521	3.22E+00
ukb-b-18408	IVW	-2.19	0.57	1.11E-04	407557	1.12E-01
ukb-b-18408	raps	-1.93	0.58	9.13E-04	407557	1.46E-01
ukb-b-18408	cML-MA	-1.64	0.72	2.34E-02	407557	1.94E-01
ukb-b-18408	divw	-2.22	0.57	8.49E-05	407557	1.08E-01

ukb-b-18408	conmix	-2.03	0.85	1.67E-02	407557	1.31E-01
ebi-a-GCST90013870	divw	0.38	0.06	3.68E-09	407609	1.46E+00
ebi-a-GCST90013870	raps	0.38	0.06	2.47E-09	407609	1.47E+00
ebi-a-GCST90013870	conmix	0.65	0.13	1.41E-06	407609	1.91E+00
ebi-a-GCST90013870	IVW	0.37	0.06	9.59E-10	407609	1.45E+00
ebi-a-GCST90013870	cML-MA	0.37	0.07	3.97E-07	407609	1.44E+00
ebi-a-GCST90013974	divw	0.37	0.06	7.69E-09	407609	1.45E+00
ebi-a-GCST90013974	IVW	0.36	0.06	2.14E-09	407609	1.44E+00
ebi-a-GCST90013974	cML-MA	0.36	0.08	1.39E-05	407609	1.43E+00
ebi-a-GCST90013974	conmix	0.58	0.12	1.91E-06	407609	1.79E+00
ebi-a-GCST90013974	raps	0.38	0.06	6.11E-09	407609	1.46E+00
ebi-a-GCST90014020	conmix	0.78	0.15	2.06E-07	407661	2.17E+00
ebi-a-GCST90014020	cML-MA	0.43	0.11	5.99E-05	407661	1.54E+00
ebi-a-GCST90014020	IVW	0.42	0.08	3.30E-07	407661	1.52E+00
ebi-a-GCST90014020	raps	0.44	0.09	2.49E-07	407661	1.55E+00
ebi-a-GCST90014020	divw	0.41	0.09	2.67E-06	407661	1.51E+00
ebi-a-GCST006696	raps	-1.67	0.49	6.68E-04	412937	1.88E-01
ebi-a-GCST006696	conmix	-1.66	0.60	5.94E-03	412937	1.90E-01
ebi-a-GCST006696	cML-MA	-1.73	0.48	3.48E-04	412937	1.78E-01
ebi-a-GCST006696	IVW	-1.66	0.47	3.90E-04	412937	1.89E-01
ebi-a-GCST006696	divw	-1.68	0.48	4.82E-04	412937	1.86E-01
ebi-a-GCST90000048	IVW	-0.21	0.04	8.03E-08	418758	8.13E-01
ebi-a-GCST90000048	cML-MA	-0.22	0.05	3.11E-06	418758	8.06E-01
ebi-a-GCST90000048	conmix	-0.30	0.06	1.63E-06	418758	7.38E-01
ebi-a-GCST90000048	raps	-0.23	0.04	3.98E-08	418758	7.97E-01
ebi-a-GCST90000048	divw	-0.23	0.04	1.71E-08	418758	7.96E-01
ukb-b-5192	divw	1.12	0.21	6.06E-08	437887	3.08E+00
ukb-b-5192	raps	1.06	0.20	1.12E-07	437887	2.89E+00
ukb-b-5192	conmix	1.16	0.30	1.11E-04	437887	3.18E+00
ukb-b-5192	cML-MA	0.97	0.27	2.62E-04	437887	2.65E+00
ukb-b-5192	IVW	0.99	0.19	2.75E-07	437887	2.68E+00
ukb-b-7647	raps	44.76	8.67	2.46E-07	446149	2.76E+19
ukb-b-7647	Wald ratio	44.76	4.77	6.72E-21	446149	2.76E+19
ukb-b-1489	divw	-0.75	0.21	3.16E-04	451486	4.70E-01
ukb-b-1489	raps	-0.76	0.21	3.53E-04	451486	4.68E-01
ukb-b-1489	conmix	-1.36	0.42	1.17E-03	451486	2.57E-01
ukb-b-1489	cML-MA	-0.74	0.26	5.19E-03	451486	4.78E-01
ukb-b-1489	IVW	-0.73	0.21	3.48E-04	451486	4.80E-01
ieu-b-5118	raps	0.63	0.11	5.62E-09	453169	1.88E+00
ieu-b-5118	cML-MA	0.61	0.13	4.26E-06	453169	1.83E+00
ieu-b-5118	conmix	1.11	0.22	4.81E-07	453169	3.04E+00
ieu-b-5118	IVW	0.61	0.10	1.55E-09	453169	1.84E+00
ieu-b-5118	divw	0.62	0.11	6.95E-09	453169	1.86E+00
ukb-b-19393	IVW	0.34	0.06	3.57E-08	454137	1.40E+00
ukb-b-19393	conmix	0.62	0.15	3.89E-05	454137	1.85E+00
ukb-b-19393	raps	0.36	0.06	3.00E-08	454137	1.43E+00

ukb-b-19393	divw	0.34	0.06	6.43E-08	454137	1.41E+00
ukb-b-19393	cML-MA	0.36	0.08	1.14E-05	454137	1.43E+00
ukb-b-20044	cML-MA	0.31	0.08	6.86E-05	454588	1.36E+00
ukb-b-20044	conmix	0.56	0.14	7.27E-05	454588	1.75E+00
ukb-b-20044	IVW	0.30	0.06	6.14E-07	454588	1.35E+00
ukb-b-20044	raps	0.31	0.06	1.56E-06	454588	1.36E+00
ukb-b-20044	divw	0.30	0.06	1.46E-06	454588	1.35E+00
ukb-b-16407	cML-MA	0.32	0.10	1.71E-03	454613	1.38E+00
ukb-b-16407	divw	0.30	0.08	5.95E-05	454613	1.35E+00
ukb-b-16407	conmix	0.68	0.15	1.22E-05	454613	1.96E+00
ukb-b-16407	raps	0.33	0.08	1.58E-05	454613	1.39E+00
ukb-b-16407	IVW	0.26	0.07	2.85E-04	454613	1.30E+00
ukb-b-8909	IVW	0.43	0.09	5.70E-07	454633	1.54E+00
ukb-b-8909	cML-MA	0.49	0.11	5.37E-06	454633	1.63E+00
ukb-b-8909	raps	0.49	0.09	6.80E-08	454633	1.64E+00
ukb-b-8909	conmix	0.83	0.16	1.46E-07	454633	2.30E+00
ukb-b-8909	divw	0.47	0.09	4.07E-07	454633	1.60E+00
ukb-b-8338	IVW	0.36	0.06	1.74E-09	454684	1.44E+00
ukb-b-8338	cML-MA	0.37	0.08	1.35E-05	454684	1.45E+00
ukb-b-8338	divw	0.37	0.06	3.48E-09	454684	1.45E+00
ukb-b-8338	conmix	0.60	0.18	7.00E-04	454684	1.82E+00
ukb-b-8338	raps	0.38	0.06	2.53E-09	454684	1.47E+00
ukb-b-20188	raps	0.54	0.09	2.80E-09	454724	1.72E+00
ukb-b-20188	cML-MA	0.55	0.13	1.07E-05	454724	1.74E+00
ukb-b-20188	conmix	0.94	0.20	3.08E-06	454724	2.55E+00
ukb-b-20188	IVW	0.50	0.09	1.10E-08	454724	1.64E+00
ukb-b-20188	divw	0.51	0.09	3.27E-08	454724	1.66E+00
ukb-b-6704	cML-MA	0.36	0.09	8.97E-05	454757	1.43E+00
ukb-b-6704	IVW	0.35	0.06	1.22E-08	454757	1.42E+00
ukb-b-6704	conmix	0.59	0.17	4.95E-04	454757	1.81E+00
ukb-b-6704	divw	0.36	0.06	2.10E-08	454757	1.43E+00
ukb-b-6704	raps	0.37	0.07	1.32E-08	454757	1.45E+00
ukb-b-12854	cML-MA	0.53	0.12	6.33E-06	454789	1.71E+00
ukb-b-12854	IVW	0.50	0.09	1.58E-08	454789	1.65E+00
ukb-b-12854	divw	0.51	0.09	4.38E-08	454789	1.67E+00
ukb-b-12854	conmix	0.81	0.19	1.81E-05	454789	2.25E+00
ukb-b-12854	raps	0.53	0.09	1.29E-08	454789	1.70E+00
ukb-b-7212	cML-MA	0.51	0.11	7.02E-06	454823	1.67E+00
ukb-b-7212	divw	0.48	0.08	4.15E-09	454823	1.61E+00
ukb-b-7212	raps	0.51	0.08	7.69E-10	454823	1.66E+00
ukb-b-7212	IVW	0.48	0.08	5.90E-10	454823	1.62E+00
ukb-b-7212	conmix	0.87	0.19	2.93E-06	454823	2.38E+00
ukb-b-18377	cML-MA	0.79	0.15	1.86E-07	454826	2.19E+00
ukb-b-18377	divw	0.76	0.12	3.29E-10	454826	2.14E+00
ukb-b-18377	conmix	1.24	0.23	3.61E-08	454826	3.46E+00
ukb-b-18377	IVW	0.74	0.11	4.96E-11	454826	2.11E+00

ukb-b-18377	raps	0.80	0.12	2.25E-11	454826	2.23E+00
ukb-b-7859	conmix	-0.13	0.12	0.30240966	454826	8.81E-01
ukb-b-7859	raps	-0.27	0.08	1.19E-03	454826	7.67E-01
ukb-b-7859	IVW	-0.29	0.08	1.33E-04	454826	7.45E-01
ukb-b-7859	cML-MA	-0.24	0.10	2.04E-02	454826	7.84E-01
ukb-b-7859	divw	-0.30	0.08	2.89E-04	454826	7.42E-01
ukb-b-19921	conmix	-0.21	0.11	5.27E-02	454840	8.07E-01
ukb-b-19921	divw	-0.25	0.07	4.15E-04	454840	7.77E-01
ukb-b-19921	cML-MA	-0.23	0.09	1.08E-02	454840	7.94E-01
ukb-b-19921	raps	-0.24	0.07	6.47E-04	454840	7.85E-01
ukb-b-19921	IVW	-0.24	0.07	3.19E-04	454840	7.85E-01
ukb-b-18096	raps	0.46	0.09	7.16E-08	454846	1.58E+00
ukb-b-18096	IVW	0.46	0.08	8.96E-09	454846	1.58E+00
ukb-b-18096	divw	0.44	0.08	1.25E-07	454846	1.55E+00
ukb-b-18096	cML-MA	0.45	0.10	1.28E-05	454846	1.57E+00
ukb-b-18096	conmix	0.92	0.21	1.18E-05	454846	2.51E+00
ukb-b-19379	IVW	-0.28	0.08	1.80E-04	454850	7.53E-01
ukb-b-19379	divw	-0.31	0.08	1.21E-04	454850	7.36E-01
ukb-b-19379	raps	-0.28	0.08	4.87E-04	454850	7.57E-01
ukb-b-19379	cML-MA	-0.25	0.10	9.55E-03	454850	7.77E-01
ukb-b-19379	conmix	-0.36	0.18	4.52E-02	454850	6.95E-01
ukb-b-20531	raps	0.65	0.12	5.05E-08	454854	1.91E+00
ukb-b-20531	cML-MA	0.64	0.15	1.24E-05	454854	1.90E+00
ukb-b-20531	conmix	0.77	0.21	2.45E-04	454854	2.16E+00
ukb-b-20531	divw	0.61	0.12	2.67E-07	454854	1.85E+00
ukb-b-20531	IVW	0.58	0.11	2.48E-07	454854	1.79E+00
ukb-b-2303	IVW	0.41	0.06	1.62E-12	454884	1.50E+00
ukb-b-2303	raps	0.41	0.06	4.01E-11	454884	1.50E+00
ukb-b-2303	conmix	0.58	0.16	1.98E-04	454884	1.79E+00
ukb-b-2303	cML-MA	0.40	0.08	1.34E-07	454884	1.49E+00
ukb-b-2303	divw	0.42	0.06	6.99E-12	454884	1.52E+00
ukb-b-12039	divw	0.24	0.06	4.51E-05	454893	1.28E+00
ukb-b-12039	cML-MA	0.25	0.08	1.99E-03	454893	1.28E+00
ukb-b-12039	conmix	0.21	0.12	9.12E-02	454893	1.23E+00
ukb-b-12039	IVW	0.24	0.06	2.29E-05	454893	1.28E+00
ukb-b-12039	raps	0.24	0.06	6.85E-05	454893	1.27E+00
ebi-a-GCST90025994	divw	0.32	0.07	1.83E-06	457756	1.37E+00
ebi-a-GCST90025994	IVW	0.32	0.06	3.67E-07	457756	1.38E+00
ebi-a-GCST90025994	raps	0.33	0.07	9.01E-07	457756	1.39E+00
ebi-a-GCST90025994	cML-MA	0.32	0.08	8.18E-05	457756	1.38E+00
ebi-a-GCST90025994	conmix	0.54	0.12	9.47E-06	457756	1.72E+00
ukb-b-11615	raps	-2.79	0.40	2.76E-12	458079	6.11E-02
ukb-b-11615	cML-MA	-2.72	0.46	3.40E-09	458079	6.60E-02
ukb-b-11615	IVW	-2.63	0.38	3.29E-12	458079	7.20E-02
ukb-b-11615	conmix	-3.69	0.63	5.39E-09	458079	2.50E-02
ukb-b-11615	divw	-2.70	0.39	4.62E-12	458079	6.73E-02

ukb-b-13799	IVW	-3.55	0.83	2.01E-05	458079	2.86E-02
ukb-b-13799	conmix	-6.23	2.05	2.35E-03	458079	1.97E-03
ukb-b-13799	cML-MA	-3.58	1.03	4.98E-04	458079	2.77E-02
ukb-b-13799	divw	-3.65	0.87	2.71E-05	458079	2.59E-02
ukb-b-13799	raps	-3.71	0.93	6.73E-05	458079	2.45E-02
ukb-b-16489	IVW	-1.59	0.18	1.66E-19	458079	2.04E-01
ukb-b-16489	raps	-1.70	0.19	7.96E-20	458079	1.83E-01
ukb-b-16489	conmix	-2.77	0.39	6.37E-13	458079	6.26E-02
ukb-b-16489	cML-MA	-1.64	0.21	8.07E-15	458079	1.93E-01
ukb-b-16489	divw	-1.73	0.19	3.06E-20	458079	1.77E-01
ukb-b-17729	cML-MA	3.06	0.50	9.63E-10	458079	2.13E+01
ukb-b-17729	IVW	2.96	0.43	7.05E-12	458079	1.92E+01
ukb-b-17729	raps	3.07	0.45	7.61E-12	458079	2.14E+01
ukb-b-17729	divw	3.03	0.44	5.87E-12	458079	2.07E+01
ukb-b-17729	conmix	4.92	0.92	9.45E-08	458079	1.37E+02
ukb-b-1867	IVW	25.84	6.48	6.67E-05	459354	1.67E+11
ukb-b-1867	raps	25.89	7.50	5.54E-04	459354	1.76E+11
ukb-b-6306	conmix	1.42	0.48	3.01E-03	460844	4.15E+00
ukb-b-6306	cML-MA	1.00	0.26	1.48E-04	460844	2.72E+00
ukb-b-6306	divw	1.15	0.22	2.69E-07	460844	3.15E+00
ukb-b-6306	raps	1.04	0.23	3.97E-06	460844	2.83E+00
ukb-b-6306	IVW	1.00	0.20	8.13E-07	460844	2.71E+00
ukb-b-16019	raps	47.85	9.22	2.14E-07	461378	6.02E+20
ukb-b-16019	Wald ratio	47.85	5.10	6.72E-21	461378	6.02E+20
ukb-b-11075	divw	-6.11	1.66	2.34E-04	461384	2.21E-03
ukb-b-11075	IVW	-5.96	1.54	1.13E-04	461384	2.58E-03
ukb-b-11075	conmix	-6.93	2.63	8.48E-03	461384	9.76E-04
ukb-b-11075	cML-MA	-5.84	1.64	3.74E-04	461384	2.91E-03
ukb-b-11075	raps	-6.03	1.69	3.56E-04	461384	2.40E-03
ebi-a-GCST90029013	cML-MA	-0.16	0.03	1.91E-08	461457	8.56E-01
ebi-a-GCST90029013	divw	-0.16	0.02	1.80E-15	461457	8.51E-01
ebi-a-GCST90029013	IVW	-0.16	0.02	1.97E-15	461457	8.54E-01
ebi-a-GCST90029013	raps	-0.16	0.02	3.59E-14	461457	8.54E-01
ebi-a-GCST90029013	conmix	-0.23	0.04	1.87E-08	461457	7.98E-01
ukb-b-19953	IVW	0.43	0.06	2.09E-13	461460	1.53E+00
ukb-b-19953	conmix	0.63	0.14	9.40E-06	461460	1.87E+00
ukb-b-19953	cML-MA	0.42	0.07	2.36E-09	461460	1.53E+00
ukb-b-19953	divw	0.44	0.06	7.53E-13	461460	1.56E+00
ukb-b-19953	raps	0.43	0.06	4.60E-12	461460	1.54E+00
ukb-b-11842	cML-MA	0.24	0.08	3.27E-03	461632	1.27E+00
ukb-b-11842	IVW	0.23	0.06	5.58E-05	461632	1.26E+00
ukb-b-11842	divw	0.23	0.06	1.03E-04	461632	1.26E+00
ukb-b-11842	raps	0.23	0.06	1.44E-04	461632	1.26E+00
ukb-b-11842	conmix	0.23	0.11	3.53E-02	461632	1.26E+00
ukb-b-15590	IVW	0.25	0.06	2.87E-05	462117	1.28E+00
ukb-b-15590	raps	0.25	0.06	7.84E-05	462117	1.29E+00

ukb-b-15590	conmix	0.17	0.16	0.26838458	462117	1.19E+00
ukb-b-15590	divw	0.26	0.06	2.60E-05	462117	1.30E+00
ukb-b-15590	cML-MA	0.25	0.08	2.48E-03	462117	1.28E+00
ukb-b-9405	divw	0.37	0.08	1.38E-05	462166	1.44E+00
ukb-b-9405	cML-MA	0.34	0.10	6.14E-04	462166	1.41E+00
ukb-b-9405	IVW	0.34	0.08	1.12E-05	462166	1.41E+00
ukb-b-9405	raps	0.37	0.08	8.10E-06	462166	1.45E+00
ukb-b-9405	conmix	0.63	0.15	2.62E-05	462166	1.88E+00
ukb-b-19698	raps	-75.20	19.63	1.28E-04	462933	2.20E-33
ukb-b-19698	Wald ratio	-75.20	15.94	2.38E-06	462933	2.20E-33
ukb-b-8755	IVW	-5.36	1.53	4.40E-04	462933	4.69E-03
ukb-b-8755	raps	-5.63	1.66	7.11E-04	462933	3.59E-03
ukb-b-8755	conmix	-8.39	3.59	1.95E-02	462933	2.26E-04
ukb-b-8755	cML-MA	-4.77	1.76	6.83E-03	462933	8.50E-03
ukb-b-8755	divw	-5.46	1.48	2.17E-04	462933	4.23E-03
ukb-b-10756	Wald ratio	- 106.15	26.63	6.71E-05	463010	7.96E-47
ukb-b-10756	raps	- 106.15	33.00	1.30E-03	463010	7.96E-47
ukb-b-10911	raps	-51.19	15.38	8.72E-04	463010	5.87E-23
ukb-b-10911	Wald ratio	-51.19	12.14	2.49E-05	463010	5.87E-23
ukb-b-14206	raps	-55.29	15.26	2.92E-04	463010	9.72E-25
ukb-b-14206	Wald ratio	-55.29	12.85	1.67E-05	463010	9.72E-25
ukb-b-15797	raps	-52.93	14.72	3.22E-04	463010	1.03E-23
ukb-b-15797	Wald ratio	-52.93	12.30	1.67E-05	463010	1.03E-23
ukb-b-1668	IVW	-6.85	1.96	4.57E-04	463010	1.05E-03
ukb-b-1668	cML-MA	-5.54	2.61	3.36E-02	463010	3.94E-03
ukb-b-1668	conmix	-10.24	4.90	3.69E-02	463010	3.59E-05
ukb-b-1668	raps	-6.65	2.08	1.39E-03	463010	1.29E-03
ukb-b-1668	divw	-6.96	1.96	3.74E-04	463010	9.47E-04
ukb-b-18802	Wald ratio	- 100.90	23.77	2.18E-05	463010	1.51E-44
ukb-b-18802	raps	- 100.90	28.36	3.75E-04	463010	1.51E-44
ebi-a-GCST90018848	raps	-2.39	0.59	5.65E-05	467253	9.17E-02
ebi-a-GCST90018848	Wald ratio	-2.39	0.41	3.87E-09	467253	9.17E-02
ebi-a-GCST90029014	raps	1.55	0.22	1.35E-12	468170	4.70E+00
ebi-a-GCST90029014	cML-MA	1.50	0.25	1.54E-09	468170	4.48E+00
ebi-a-GCST90029014	divw	1.65	0.21	1.12E-14	468170	5.19E+00
ebi-a-GCST90029014	IVW	1.55	0.20	1.18E-14	468170	4.72E+00
ebi-a-GCST90029014	conmix	1.88	0.37	3.67E-07	468170	6.55E+00
ebi-a-GCST90018793	conmix	-0.15	0.07	5.05E-02	470931	8.64E-01
ebi-a-GCST90018793	IVW	-0.22	0.05	1.45E-05	470931	8.01E-01
ebi-a-GCST90018793	raps	-0.20	0.05	9.67E-05	470931	8.19E-01
ebi-a-GCST90018793	cML-MA	-0.16	0.07	1.66E-02	470931	8.48E-01
ebi-a-GCST90018793	divw	-0.23	0.05	1.31E-05	470931	7.98E-01
ebi-a-GCST90029012	conmix	-3.12	0.46	1.96E-11	470941	4.42E-02

ebi-a-GCST90029012	IVW	-1.85	0.21	9.27E-19	470941	1.58E-01
ebi-a-GCST90029012	raps	-1.88	0.22	1.68E-17	470941	1.53E-01
ebi-a-GCST90029012	divw	-1.89	0.21	1.31E-18	470941	1.51E-01
ebi-a-GCST90029012	cML-MA	-1.90	0.28	1.91E-11	470941	1.50E-01
ebi-a-GCST90012877	IVW	-0.11	0.03	3.80E-04	472868	8.97E-01
ebi-a-GCST90012877	divw	-0.11	0.03	3.79E-04	472868	8.96E-01
ebi-a-GCST90012877	conmix	-0.13	0.06	4.07E-02	472868	8.82E-01
ebi-a-GCST90012877	raps	-0.11	0.03	1.21E-03	472868	9.00E-01
ebi-a-GCST90012877	cML-MA	-0.10	0.04	6.74E-03	472868	9.03E-01
ebi-a-GCST90018902	raps	1.51	0.31	8.49E-07	477734	4.52E+00
ebi-a-GCST90018902	Wald ratio	1.51	0.12	1.97E-38	477734	4.52E+00
ebi-a-GCST90018890	raps	-0.40	0.09	1.65E-05	483078	6.69E-01
ebi-a-GCST90018890	divw	-0.40	0.09	1.13E-05	483078	6.70E-01
ebi-a-GCST90018890	conmix	-0.49	0.17	3.41E-03	483078	6.15E-01
ebi-a-GCST90018890	cML-MA	-0.41	0.10	4.46E-05	483078	6.67E-01
ebi-a-GCST90018890	IVW	-0.39	0.09	1.32E-05	483078	6.75E-01
ebi-a-GCST90029007	raps	0.44	0.06	7.08E-14	532396	1.55E+00
ebi-a-GCST90029007	IVW	0.43	0.06	1.38E-14	532396	1.53E+00
ebi-a-GCST90029007	cML-MA	0.43	0.08	1.33E-08	532396	1.54E+00
ebi-a-GCST90029007	conmix	0.61	0.11	7.21E-08	532396	1.84E+00
ebi-a-GCST90029007	divw	0.43	0.06	9.09E-14	532396	1.54E+00
ebi-a-GCST90000050	cML-MA	-0.17	0.06	5.06E-03	542901	8.48E-01
ebi-a-GCST90000050	raps	-0.17	0.05	2.09E-04	542901	8.42E-01
ebi-a-GCST90000050	divw	-0.18	0.05	1.18E-04	542901	8.34E-01
ebi-a-GCST90000050	conmix	-0.21	0.07	1.66E-03	542901	8.13E-01
ebi-a-GCST90000050	IVW	-0.18	0.05	1.30E-04	542901	8.38E-01
ebi-a-GCST90000514	cML-MA	0.44	0.10	1.02E-05	602604	1.55E+00
ebi-a-GCST90000514	divw	0.49	0.08	5.42E-09	602604	1.63E+00
ebi-a-GCST90000514	conmix	0.57	0.13	1.87E-05	602604	1.76E+00
ebi-a-GCST90000514	IVW	0.45	0.08	7.34E-09	602604	1.56E+00
ebi-a-GCST90000514	raps	0.46	0.08	5.52E-08	602604	1.59E+00
ieu-b-4877	raps	0.65	0.11	5.04E-10	607291	1.92E+00
ieu-b-4877	divw	0.64	0.10	5.16E-10	607291	1.90E+00
ieu-b-4877	IVW	0.66	0.09	1.02E-12	607291	1.94E+00
ieu-b-4877	cML-MA	0.66	0.13	2.40E-07	607291	1.93E+00
ieu-b-4877	conmix	0.97	0.20	8.02E-07	607291	2.63E+00
ieu-b-40	divw	0.34	0.06	3.56E-09	681275	1.40E+00
ieu-b-40	IVW	0.35	0.06	3.63E-10	681275	1.41E+00
ieu-b-40	raps	0.33	0.06	1.15E-08	681275	1.40E+00
ieu-b-40	conmix	0.43	0.12	2.29E-04	681275	1.53E+00
ieu-b-40	cML-MA	0.33	0.07	6.46E-07	681275	1.39E+00
ieu-a-1239	IVW	-0.89	0.09	9.21E-24	766345	4.09E-01
ieu-a-1239	cML-MA	-0.87	0.11	7.76E-15	766345	4.19E-01
ieu-a-1239	conmix	-1.55	0.22	4.45E-12	766345	2.12E-01
ieu-a-1239	divw	-0.91	0.09	9.37E-24	766345	4.01E-01
ieu-a-1239	raps	-0.88	0.09	6.45E-21	766345	4.14E-01

prot-c-5102_55_3	raps	-0.23	0.03	3.10E-11	3080	7.96E-01
prot-c-5102_55_3	IVW	-0.23	0.03	3.40E-13	3080	7.96E-01
prot-c-5312_49_3	raps	-0.22	0.07	1.93E-03	3080	7.99E-01
prot-c-5312_49_3	Wald ratio	-0.22	0.06	4.34E-04	3080	7.99E-01
prot-c-5494_52_3	Wald ratio	0.22	0.06	4.34E-04	3080	1.24E+00
prot-c-5494_52_3	raps	0.22	0.07	1.81E-03	3080	1.24E+00

supplementary Table 6 The heterogeneity test of between exposures and all subtypes of lung cancer

id.exposure	method	Q	Q_pval	I2	type
ieu-b-24	MR Egger	0.00	1.00	0	LUSC
eqtl-a-ENSG00000165905	MR Egger	0.00	0.99	0	Lung cancer
eqtl-a-ENSG00000041357	MR Egger	0.00	0.94	0	LUAD
prot-c-4964_67_1	IVW	0.01	0.94	0	Lung cancer
prot-c-5102_55_3	IVW	0.01	0.92	0	LUAD
prot-a-2481	IVW	0.01	0.91	0	Lung cancer
ebi-a-GCST90006921	IVW	0.01	0.91	0	LUSC
eqtl-a-ENSG00000165905	IVW	0.03	0.98	0	Lung cancer
eqtl-a-ENSG00000198518	IVW	0.03	0.85	0	LUSC
eqtl-a-ENSG00000112812	IVW	0.04	0.85	0	Lung cancer
ukb-b-2399	IVW	0.04	0.83	0	Lung cancer
eqtl-a-ENSG00000250366	IVW	0.05	0.83	0	LUSC
eqtl-a-ENSG00000010818	IVW	0.12	0.94	0	Lung cancer
eqtl-a-ENSG00000072163	MR Egger	0.28	0.99	0	LUSC
eqtl-a-ENSG00000010818	MR Egger	0.07	0.79	0	Lung cancer
prot-a-1124	IVW	0.08	0.78	0	Lung cancer
ebi-a-GCST90006921	IVW	0.08	0.78	0	Lung cancer
eqtl-a-ENSG00000138496	IVW	0.10	0.75	0	Lung cancer
eqtl-a-ENSG00000106305	MR Egger	0.21	0.90	0	LUSC
eqtl-a-ENSG00000177406	MR Egger	0.64	1.00	0	LUAD
ukb-b-2732	MR Egger	0.12	0.73	0	SCLC
ukb-a-180	MR Egger	0.13	0.72	0	Lung cancer
prot-a-1238	IVW	0.13	0.72	0	Lung cancer
eqtl-a-ENSG00000106305	IVW	0.39	0.94	0	LUSC
ebi-a-GCST90060467	IVW	0.13	0.72	0	LUSC
ieu-b-24	IVW	0.29	0.87	0	LUSC
eqtl-a-ENSG00000082641	IVW	0.30	0.86	0	SCLC
ukb-b-12018	MR Egger	0.17	0.68	0	SCLC
ukb-a-343	MR Egger	0.17	0.68	0	SCLC
ebi-a-GCST90018890	IVW	0.54	0.91	0	Lung cancer
eqtl-a-ENSG00000177406	IVW	1.31	0.99	0	LUAD
ukb-d-K11_OTHDIG	IVW	0.19	0.66	0	LUAD
eqtl-a-ENSG00000041357	IVW	0.40	0.82	0	LUSC
ukb-b-12018	MR Egger	0.21	0.65	0	Lung cancer
prot-a-1051	MR Egger	0.43	0.81	0	LUAD
ukb-a-243	IVW	0.21	0.64	0	SCLC
ebi-a-GCST90060127	MR Egger	0.22	0.64	0	Lung cancer
eqtl-a-ENSG00000065911	IVW	0.66	0.88	0	SCLC
ebi-a-GCST90018890	MR Egger	0.45	0.80	0	Lung cancer
eqtl-a-ENSG00000137218	MR Egger	0.46	0.80	0	SCLC
eqtl-a-ENSG00000204789	IVW	1.17	0.95	0	LUAD
eqtl-a-ENSG00000239415	IVW	0.70	0.87	0	Lung cancer
ebi-a-GCST009971	MR Egger	1.42	0.96	0	SCLC
ukb-b-11075	MR Egger	0.48	0.79	0	LUSC

eqtl-a-ENSG00000074657	IVW	0.99	0.91	0	LUSC
eqtl-a-ENSG00000259015	IVW	0.76	0.86	0	LUAD
eqtl-a-ENSG00000107593	IVW	0.25	0.62	0	LUSC
eqtl-a-ENSG00000063438	MR Egger	0.51	0.78	0	LUSC
ebi-a-GCST90060337	IVW	0.26	0.61	0	Lung cancer
eqtl-a-ENSG00000204789	MR Egger	1.11	0.89	0	LUAD
eqtl-a-ENSG00000082641	MR Egger	0.28	0.60	0	SCLC
eqtl-a-ENSG00000100450	IVW	1.12	0.89	0	LUSC
eqtl-a-ENSG00000196812	MR Egger	0.29	0.59	0	LUSC
ebi-a-GCST009971	IVW	2.02	0.96	0	SCLC
eqtl-a-ENSG00000065911	MR Egger	0.59	0.75	0	SCLC
ebi-a-GCST90060470	IVW	0.31	0.58	0	LUSC
eqtl-a-ENSG00000145416	MR Egger	2.17	0.95	0	SCLC
eqtl-a-ENSG00000259015	MR Egger	0.62	0.73	0	LUAD
eqtl-a-ENSG00000041357	MR Egger	0.32	0.57	0	LUSC
eqtl-a-ENSG00000074657	MR Egger	0.95	0.81	0	LUSC
prot-a-710	IVW	1.30	0.86	0	LUSC
eqtl-a-ENSG00000179361	IVW	0.33	0.57	0	Lung cancer
eqtl-a-ENSG00000239415	MR Egger	0.67	0.72	0	Lung cancer
ukb-b-3855	MR Egger	1.34	0.85	0	SCLC
eqtl-a-ENSG00000166763	IVW	0.34	0.56	0	Lung cancer
eqtl-a-ENSG00000260228	MR Egger	0.68	0.71	0	LUSC
eqtl-a-ENSG00000108384	MR Egger	4.83	0.99	0	LUSC
eqtl-a-ENSG00000259015	MR Egger	0.69	0.71	0	Lung cancer
eqtl-a-ENSG00000107890	IVW	1.41	0.84	0	Lung cancer
eqtl-a-ENSG00000072163	IVW	1.77	0.88	0	LUSC
prot-a-2470	IVW	1.43	0.84	0	Lung cancer
prot-a-2470	MR Egger	1.08	0.78	0	Lung cancer
finn-b-DM_PERIPHATHERO	MR Egger	0.73	0.69	0	LUSC
eqtl-a-ENSG00000108384	IVW	5.54	0.99	0	LUSC
eqtl-a-ENSG00000100450	MR Egger	1.11	0.78	0	LUSC
ukb-a-344	IVW	0.37	0.54	0	Lung cancer
eqtl-a-ENSG00000145416	IVW	3.38	0.95	0	Lung cancer
finn-b-DM_PERIPHATHERO	IVW	1.13	0.77	0	LUSC
eqtl-a-ENSG00000260228	IVW	1.14	0.77	0	LUSC
eqtl-a-ENSG00000145416	IVW	3.09	0.93	0	SCLC
eqtl-a-ENSG00000189298	IVW	0.39	0.53	0	LUSC
eqtl-a-ENSG00000145416	MR Egger	3.17	0.92	0	Lung cancer
eqtl-a-ENSG00000100462	IVW	2.78	0.90	0	Lung cancer
ukb-a-132	IVW	1.60	0.81	0	Lung cancer
prot-a-710	MR Egger	1.21	0.75	0	LUSC
ukb-b-3855	IVW	2.02	0.85	0	SCLC
ebi-a-GCST90060342	IVW	0.41	0.52	0	Lung cancer
ebi-a-GCST006696	IVW	1.23	0.75	0	LUSC
eqtl-a-ENSG00000066084	IVW	1.66	0.80	0	LUAD
eqtl-a-ENSG00000166763	IVW	0.41	0.52	0	LUSC

ukb-b-12841	IVW	1.27	0.74	0	LUSC
ebi-a-GCST90016675	IVW	1.79	0.77	0	LUSC
ebi-a-GCST90060127	IVW	0.91	0.63	0	Lung cancer
eqtl-a-ENSG00000107890	MR Egger	1.37	0.71	0	Lung cancer
eqtl-a-ENSG00000213694	MR Egger	2.75	0.84	0	SCLC
eqtl-a-ENSG00000173744	IVW	0.46	0.50	0	LUSC
ukb-b-1867	IVW	0.46	0.50	0	LUSC
eqtl-a-ENSG00000100462	MR Egger	2.77	0.84	0	Lung cancer
eqtl-a-ENSG00000259015	IVW	1.39	0.71	0	Lung cancer
ukb-a-132	MR Egger	1.40	0.70	0	Lung cancer
ebi-a-GCST90019476	IVW	1.89	0.76	0	LUSC
eqtl-a-ENSG00000063438	IVW	1.43	0.70	0	LUSC
prot-c-5102_55_3	IVW	0.49	0.48	0	Lung cancer
ebi-a-GCST006696	MR Egger	0.99	0.61	0	LUSC
eqtl-a-ENSG00000229515	IVW	2.48	0.78	0	LUSC
finn-b-AD_LO	MR Egger	5.52	0.90	0	SCLC
finn-b-DM_PERIPHATHERO	IVW	1.52	0.68	0	Lung cancer
eqtl-a-ENSG00000066084	MR Egger	1.53	0.68	0	LUAD
eqtl-a-ENSG00000124549	IVW	0.51	0.48	0	SCLC
eqtl-a-ENSG00000111906	IVW	8.19	0.94	0	LUSC
eqtl-a-ENSG00000229515	MR Egger	2.07	0.72	0	LUSC
finn-b-AD_LO	IVW	6.27	0.90	0	SCLC
eqtl-a-ENSG00000106009	MR Egger	2.11	0.72	0	Lung cancer
ukb-b-8184	IVW	0.53	0.47	0	Lung cancer
ebi-a-GCST90016675	MR Egger	1.60	0.66	0	LUSC
eqtl-a-ENSG00000111906	MR Egger	8.18	0.92	0	LUSC
eqtl-a-ENSG00000140650	IVW	2.27	0.69	0	LUSC
eqtl-a-ENSG00000177706	MR Egger	2.28	0.68	0	Lung cancer
eqtl-a-ENSG00000169554	IVW	0.58	0.45	0	Lung cancer
eqtl-a-ENSG00000095261	IVW	1.17	0.56	0	Lung cancer
ukb-b-12018	IVW	1.19	0.55	0	SCLC
eqtl-a-ENSG00000166037	MR Egger	1.19	0.55	0	Lung cancer
eqtl-a-ENSG00000106009	IVW	3.01	0.70	0	Lung cancer
eqtl-a-ENSG00000177508	MR Egger	6.63	0.83	0	Lung cancer
eqtl-a-ENSG00000041357	IVW	1.24	0.54	0	LUAD
ebi-a-GCST90019476	MR Egger	1.89	0.60	0	LUSC
ukb-b-12841	MR Egger	1.26	0.53	0	LUSC
prot-a-1051	IVW	1.89	0.60	0	Lung cancer
ebi-a-GCST90006898	IVW	0.64	0.42	0	Lung cancer
ubm-b-687	IVW	0.64	0.42	0	LUSC
finn-b-AD_LO	MR Egger	7.09	0.79	0	Lung cancer
ukb-a-202	IVW	1.95	0.58	0	Lung cancer
eqtl-a-ENSG00000166037	IVW	1.95	0.58	0	Lung cancer
eqtl-a-ENSG00000095261	MR Egger	0.65	0.42	0	Lung cancer
eqtl-a-ENSG00000175164	IVW	4.57	0.71	0	LUSC
eqtl-a-ENSG00000211677	IVW	1.96	0.58	0	LUAD

finn-b-AD_LO	IVW	7.92	0.79	0	Lung cancer
eqtl-a-ENSG00000213694	IVW	4.64	0.70	0	SCLC
eqtl-a-ENSG00000137218	IVW	1.99	0.57	0	SCLC
eqtl-a-ENSG00000134758	IVW	3.37	0.64	0	LUSC
eqtl-a-ENSG00000060709	IVW	4.04	0.67	0	LUSC
eqtl-a-ENSG00000142233	IVW	2.03	0.57	0	LUAD
ukb-a-238	MR Egger	5.51	0.70	0	Lung cancer
eqtl-a-ENSG00000177508	IVW	8.33	0.76	0	Lung cancer
ubm-b-1447	IVW	0.70	0.40	0	Lung cancer
ukb-b-5174	MR Egger	11.22	0.80	0	LUAD
ukb-a-202	MR Egger	1.41	0.49	0	Lung cancer
ukb-b-4667	IVW	10.57	0.78	0	Lung cancer
eqtl-a-ENSG00000189298	IVW	0.71	0.40	0	Lung cancer
eqtl-a-ENSG00000175164	MR Egger	4.25	0.64	0	LUSC
eqtl-a-ENSG00000198518	IVW	0.71	0.40	0	Lung cancer
ukb-b-2205	IVW	0.72	0.40	0	Lung cancer
eqtl-a-ENSG00000171055	IVW	13.68	0.80	0	Lung cancer
ukb-a-399	MR Egger	36.12	0.91	0	LUSC
eqtl-a-ENSG00000140650	MR Egger	2.22	0.53	0	LUSC
eqtl-a-ENSG00000171055	MR Egger	13.33	0.77	0	Lung cancer
ukb-b-7408	IVW	32.22	0.89	0	SCLC
eqtl-a-ENSG00000156958	IVW	0.75	0.39	0	LUAD
ukb-b-4667	MR Egger	10.54	0.72	0	Lung cancer
finn-b-DM_PERIPHATHERO	MR Egger	1.51	0.47	0	Lung cancer
ukb-b-7408	MR Egger	31.77	0.87	0	SCLC
finn-b-CD2_BENIGN_LEIOMYOMA_UTERI	MR Egger	15.20	0.76	0	LUSC
ukb-b-5174	IVW	12.96	0.74	0	LUAD
ukb-a-399	IVW	38.60	0.88	0	LUSC
finn-b-G6_AD_WIDE	IVW	13.92	0.73	0	SCLC
ebi-a-GCST90060294	IVW	0.79	0.37	0	Lung cancer
eqtl-a-ENSG00000175164	IVW	6.32	0.61	0	Lung cancer
ukb-b-469	IVW	1.60	0.45	0	LUAD
finn-b-CD2_BENIGN_LEIOMYOMA_UTERI	IVW	16.93	0.72	0	LUSC
eqtl-a-ENSG00000060709	MR Egger	4.04	0.54	0	LUSC
eqtl-a-ENSG00000134758	MR Egger	3.23	0.52	0	LUSC
finn-b-G6_AD_WIDE	MR Egger	13.77	0.68	0	SCLC
eqtl-a-ENSG00000100462	IVW	5.69	0.58	0	LUAD
eqtl-a-ENSG00000119403	IVW	7.33	0.60	0	Lung cancer
eqtl-a-ENSG00000174007	IVW	11.44	0.65	0	Lung cancer
eqtl-a-ENSG00000174007	MR Egger	10.66	0.64	0	Lung cancer
eqtl-a-ENSG00000145476	MR Egger	9.85	0.63	0	LUSC
eqtl-a-ENSG00000141298	MR Egger	6.56	0.58	0	Lung cancer
ubm-b-3277	IVW	1.65	0.44	0	LUSC
eqtl-a-ENSG00000205978	IVW	3.31	0.51	0	LUSC
ukb-b-12405	IVW	28.14	0.75	0	LUSC
ukb-b-13799	MR Egger	11.61	0.64	0	LUSC

eqtl-a-ENSG00000103160	IVW	4.17	0.52	0	Lung cancer
ukb-b-16878	IVW	22.54	0.71	0	Lung cancer
finn-b-KRA_PSY_DEMENTIA	MR Egger	7.51	0.58	0	SCLC
ukb-b-12405	MR Egger	27.56	0.73	0	LUSC
finn-b-KRA_PSY_DEMENTIA	IVW	8.39	0.59	0	SCLC
finn-b-CD2_BENIGN_LEIOMYOMA_UTERI_EXALLC	MR Egger	13.45	0.64	0	LUSC
ukb-b-11075	IVW	2.53	0.47	0	LUSC
eqtl-a-ENSG00000101574	MR Egger	1.69	0.43	0	Lung cancer
ukb-b-6019	MR Egger	5.08	0.53	0	LUAD
ukb-a-238	IVW	7.65	0.57	0	Lung cancer
finn-b-KRA_PSY_DEMENTIA_EXMORE	MR Egger	6.82	0.56	0	SCLC
prot-a-1051	MR Egger	1.72	0.42	0	Lung cancer
ukb-d-D22	IVW	5.17	0.52	0	LUAD
ukb-a-237	MR Egger	5.19	0.52	0	Lung cancer
ukb-b-16878	MR Egger	22.48	0.66	0	Lung cancer
finn-b-KRA_PSY_DEMENTIA_EXMORE	IVW	7.79	0.56	0	SCLC
eqtl-a-ENSG00000101574	IVW	2.62	0.45	0	Lung cancer
ebi-a-GCST90018890	MR Egger	1.76	0.41	0	LUSC
ebi-a-GCST90000048	IVW	53.14	0.72	0	LUSC
eqtl-a-ENSG00000139531	IVW	8.92	0.54	0	Lung cancer
ukb-b-13799	MR Egger	11.60	0.56	0	Lung cancer
ebi-a-GCST90000048	MR Egger	52.74	0.70	0	LUSC
ukb-a-202	IVW	3.58	0.47	0	LUSC
eqtl-a-ENSG00000175164	MR Egger	6.27	0.51	0	Lung cancer
eqtl-a-ENSG00000124508	IVW	4.48	0.48	0	SCLC
eqtl-a-ENSG00000260276	IVW	7.18	0.52	0	LUSC
eqtl-a-ENSG00000119403	MR Egger	7.19	0.52	0	Lung cancer
ubm-b-847	IVW	2.70	0.44	0	Lung cancer
finn-b-D3_ANAEMIA_B12_DEF	MR Egger	0.91	0.34	0	Lung cancer
ukb-a-397	IVW	170.21	0.81	0	LUSC
ubm-b-289	IVW	2.73	0.44	0	Lung cancer
eqtl-a-ENSG00000168411	MR Egger	5.46	0.49	0	Lung cancer
ukb-a-397	MR Egger	169.56	0.80	0	LUSC
ukb-b-11615	IVW	70.23	0.69	0	LUAD
ukb-b-11615	MR Egger	69.68	0.68	0	LUAD
eqtl-a-ENSG00000026297	IVW	14.70	0.55	0	Lung cancer
ukb-b-13799	IVW	12.92	0.53	0	Lung cancer
ukb-a-238	IVW	8.38	0.50	0	LUAD
eqtl-a-ENSG00000205726	MR Egger	8.41	0.49	0	LUSC
eqtl-a-ENSG00000135698	MR Egger	11.24	0.51	0	LUAD
eqtl-a-ENSG00000167483	IVW	4.69	0.45	0	Lung cancer
eqtl-a-ENSG00000198563	IVW	0.94	0.33	0	SCLC
ukb-b-16878	MR Egger	27.39	0.55	0	LUSC
eqtl-a-ENSG00000100462	MR Egger	5.67	0.46	0	LUAD
ubm-b-3277	MR Egger	0.95	0.33	0	LUSC

ukb-b-15169	IVW	4.73	0.45	0	Lung cancer
prot-a-3203	IVW	3.80	0.43	0	LUSC
eqtl-a-ENSG00000142233	MR Egger	1.90	0.39	0	LUAD
finn-b-DM_SEVERAL_COMPLICATIONS	MR Egger	5.70	0.46	0	SCLC
prot-a-1051	IVW	2.85	0.41	0	LUAD
ukb-a-176	IVW	14.31	0.50	0	LUSC
eqtl-a-ENSG00000103160	MR Egger	3.83	0.43	0	Lung cancer
ukb-a-176	MR Egger	13.42	0.49	0	LUSC
ukb-b-16878	IVW	28.79	0.53	0	LUSC
eqtl-a-ENSG00000139531	MR Egger	8.66	0.47	0	Lung cancer
ukb-b-15169	IVW	5.78	0.45	0	LUSC
eqtl-a-ENSG00000177706	IVW	4.87	0.43	0	Lung cancer
eqtl-a-ENSG00000197077	MR Egger	10.71	0.47	0	Lung cancer
eqtl-a-ENSG00000026297	MR Egger	14.65	0.48	0	Lung cancer
eqtl-a-ENSG00000211677	MR Egger	1.96	0.38	0	LUAD
ieu-b-4877	IVW	78.91	0.51	0	LUAD
ukb-a-237	IVW	6.92	0.44	0	LUAD
ebi-a-GCST90029012	MR Egger	224.70	0.53	0	LUSC
ebi-a-GCST90029012	IVW	225.88	0.53	0	LUSC
ieu-b-4877	MR Egger	78.32	0.50	0	LUAD
ebi-a-GCST90018934	MR Egger	45.66	0.49	0	LUSC
finn-b-I9_REVASC	IVW	24.82	0.47	0	Lung cancer
eqtl-a-ENSG00000124508	MR Egger	3.98	0.41	0	SCLC
finn-b-CD2_BENIGN_EXALLC	MR Egger	11.94	0.45	0	LUSC
ukb-d-D22	MR Egger	4.98	0.42	0	LUAD
ebi-a-GCST90012024	MR Egger	7.98	0.44	0	Lung cancer
ukb-b-13799	IVW	15.03	0.45	2.1427307232717301E-3	LUSC
ebi-a-GCST004441	IVW	3.01	0.39	2.5701166581864701E-3	LUAD
eqtl-a-ENSG00000145476	IVW	13.04	0.44	3.1279948399249402E-3	LUSC
ukb-b-11615	IVW	73.27	0.47	3.6397970645778198E-3	LUSC
ukb-a-237	IVW	7.03	0.43	4.6955855894434302E-3	Lung cancer
eqtl-a-ENSG00000260276	MR Egger	7.04	0.43	4.9816999284765101E-3	LUSC
ukb-a-142	IVW	14.09	0.44	6.4065639798240297E-3	Lung cancer
ukb-b-7408	IVW	44.29	0.46	6.4991812693483801E-3	LUSC
finn-b-CD2_BENIGN_LEIOMYOMA_UTERI_EXALLC	IVW	17.14	0.44	8.3708295968590897E-3	LUSC
eqtl-a-ENSG00000169045	MR Egger	8.10	0.42	1.2298930226449701E-2	Lung cancer
ebi-a-GCST90000048	MR Egger	58.72	0.45	1.23109926395198E-2	Lung cancer

ukb-a-399	IVW	48.79	0.44	1.6112149189642901E-2	SCLC
ukb-b-10831	MR Egger	10.17	0.43	1.63061117648951E-2	LUAD
ukb-b-11615	MR Egger	73.25	0.44	1.7130487136578301E-2	LUSC
finn-b-CD2_BENIGN_EXALLC	IVW	13.25	0.43	1.8867814915709401E-2	LUSC
ukb-a-398	MR Egger	57.18	0.43	2.0606525223377001E-2	LUSC
eqtl-a-ENSG00000135698	IVW	13.29	0.43	2.1629536797737499E-2	LUAD
finn-b-I9_REVASC	MR Egger	24.54	0.43	2.1813776991004299E-2	Lung cancer
ebi-a-GCST90000048	IVW	60.33	0.43	2.2078702226134599E-2	Lung cancer
ukb-b-16489	IVW	312.93	0.38	2.2152752222283401E-2	LUSC
finn-b-F5_DEMENTIA_INCLAVO	IVW	14.33	0.43	2.30558369811628E-2	LUSC
ebi-a-GCST006250	IVW	177.38	0.39	2.4697916053887999E-2	SCLC
ukb-b-12018	IVW	2.05	0.36	2.50661506704975E-2	Lung cancer
ukb-a-399	MR Egger	48.22	0.42	2.5242910654653401E-2	SCLC
ukb-b-16489	MR Egger	312.93	0.36	2.5342717822338701E-2	LUSC
eqtl-a-ENSG00000156414	IVW	16.43	0.42	2.6156876146499499E-2	LUAD
ebi-a-GCST006250	MR Egger	177.06	0.38	2.85764464130745E-2	SCLC
ukb-b-7408	MR Egger	44.27	0.42	2.87780768980705E-2	LUSC
ukb-b-6134	IVW	40.17	0.42	2.9117283780711701E-2	Lung cancer
ukb-b-6306	IVW	106.34	0.39	3.1364160745906998E-2	LUSC
ebi-a-GCST90000046	MR Egger	63.00	0.41	3.1717208850505198E-2	LUSC
ukb-b-6306	MR Egger	105.53	0.39	3.3411496954488099E-2	LUSC
eqtl-a-ENSG00000141298	IVW	9.32	0.41	3.4358644096607598E-2	Lung cancer
eqtl-a-ENSG00000188199	MR Egger	4.14	0.39	3.4891899557409199E-2	LUAD
ebi-a-GCST90029013	IVW	238.52	0.34	3.5718103828609897E-2	LUSC
ebi-a-GCST90029007	IVW	737.79	0.24	3.6315606087556199E-2	SCLC
ebi-a-GCST90029007	MR Egger	737.65	0.23	3.7483107754307098E-2	SCLC
ebi-a-GCST90029013	MR Egger	238.45	0.32	3.9634228929149098E-2	LUSC
eqtl-a-ENSG00000188199	IVW	5.22	0.39	4.14084150895574E-2	LUAD

ukb-a-505	IVW	17.75	0.40	4.2421094183225101E-2	Lung cancer
ebi-a-GCST90000045	IVW	79.44	0.37	4.3259898562584102E-2	LUSC
ukb-a-238	MR Egger	8.38	0.40	4.5003526216886802E-2	LUAD
prot-c-5102_55_3	IVW	1.05	0.31	4.51023278652753E-2	SCLC
ebi-a-GCST90029013	IVW	239.03	0.29	4.6134336743599798E-2	SCLC
ukb-a-434	IVW	8.39	0.40	4.7005968813366002E-2	Lung cancer
ukb-a-28	IVW	10.51	0.40	4.8908863584220599E-2	LUAD
finn-b-F5_DEMENTIA_INCLAVO	MR Egger	13.68	0.40	4.9855830083083398E-2	LUSC
ebi-a-GCST90029013	MR Egger	238.98	0.28	5.0130416697960103E-2	SCLC
ukb-b-6134	MR Egger	40.10	0.38	5.2431392848168203E-2	Lung cancer
ukb-b-2303	IVW	666.21	0.16	5.2854822796178098E-2	SCLC
ebi-a-GCST90029013	MR Egger	245.05	0.27	5.3241347044910098E-2	LUAD
ebi-a-GCST90018992	MR Egger	1.06	0.30	5.3399439336377197E-2	LUSC
ebi-a-GCST90000045	MR Egger	79.26	0.35	5.3696516111179003E-2	LUSC
ukb-b-2303	MR Egger	666.18	0.15	5.43043587370442E-2	SCLC
ukb-b-17729	MR Egger	88.85	0.34	5.4535141810619303E-2	SCLC
ieu-b-4877	IVW	87.87	0.34	5.5374439415060803E-2	LUSC
ebi-a-GCST90000514	IVW	74.28	0.34	5.7576358611123299E-2	LUSC
ebi-a-GCST90000046	IVW	65.88	0.34	5.8838367222250998E-2	LUSC
ukb-b-19953	IVW	681.47	0.12	6.08583990989272E-2	SCLC
ukb-b-19953	MR Egger	681.36	0.12	6.2167708583129901E-2	SCLC
ebi-a-GCST90029013	IVW	249.17	0.22	6.4883777303226003E-2	LUAD
ebi-a-GCST90000514	MR Egger	73.80	0.32	6.5088658354600107E-2	LUSC
ukb-d-20116_0	MR Egger	65.32	0.33	6.6068338760485207E-2	Lung cancer
ieu-b-4877	MR Egger	87.85	0.31	6.6552071766770096E-2	LUSC
prot-a-1347	IVW	1.07	0.30	6.75695258956545E-2	Lung cancer
ieu-a-1239	MR Egger	411.08	0.16	6.83065835938097E-2	SCLC
eqtl-a-ENSG00000168411	IVW	7.51	0.38	6.8391028467523304E-2	Lung cancer

eqtl-a-ENSG00000196812	MR Egger	1.07	0.30	6.8914125263656395E-2	Lung cancer
ukb-b-6704	IVW	624.10	0.10	6.9055923790659099E-2	SCLC
ukb-b-6704	MR Egger	623.31	0.10	6.9486210563321604E-2	SCLC
ieu-a-1239	IVW	413.11	0.15	7.0456069092620296E-2	SCLC
ukb-a-202	MR Egger	3.23	0.36	7.1868213790471397E-2	LUSC
eqtl-a-ENSG00000156414	IVW	17.25	0.37	7.2691322946852696E-2	Lung cancer
eqtl-a-ENSG00000168411	IVW	5.40	0.37	7.3618732381393895E-2	SCLC
eqtl-a-ENSG00000205726	IVW	10.80	0.37	7.4185590005316895E-2	LUSC
ebi-a-GCST90019017	MR Egger	62.67	0.31	7.4445090607389505E-2	LUSC
ukb-a-28	MR Egger	9.73	0.37	7.4665053763160003E-2	LUAD
ukb-a-142	MR Egger	14.09	0.37	7.7056228998929194E-2	Lung cancer
ebi-a-GCST90013870	IVW	586.34	0.08	7.9027020641672802E-2	SCLC
eqtl-a-ENSG00000156414	MR Egger	16.30	0.36	7.9829125065437295E-2	LUAD
ukb-b-17729	IVW	92.44	0.27	8.0452833721588998E-2	SCLC
ebi-a-GCST90013870	MR Egger	586.33	0.08	8.0718196456879304E-2	SCLC
ukb-b-5174	MR Egger	16.33	0.36	8.1609089941750507E-2	Lung cancer
finn-b-F5_DEMENTIA	IVW	14.19	0.36	8.3731348466301106E-2	LUSC
ebi-a-GCST90013974	IVW	588.32	0.07	8.3825132749086095E-2	SCLC
eqtl-a-ENSG00000111906	IVW	17.47	0.36	8.4130274148579695E-2	Lung cancer
ebi-a-GCST90013974	MR Egger	588.30	0.07	8.5506817165398805E-2	SCLC
ebi-a-GCST90012024	IVW	9.85	0.36	8.5972858009033495E-2	Lung cancer
ukb-b-5174	IVW	17.52	0.35	8.6525191255644396E-2	Lung cancer
eqtl-a-ENSG00000205978	MR Egger	3.29	0.35	8.7293327845348601E-2	LUSC
ebi-a-GCST90104006	IVW	85.47	0.26	8.7409341333749294E-2	LUSC
ieu-a-1239	IVW	418.62	0.10	8.7477902666058899E-2	LUSC

ukb-d-I9_PAD	IVW	1.10	0.29	8.8366172906107293E-2	Lung cancer
ieu-a-1239	MR Egger	418.33	0.09	8.9233140582287304E-2	LUSC
ukb-a-398	MR Egger	61.54	0.28	9.0053666530752099E-2	SCLC
eqtl-a-ENSG00000156414	MR Egger	16.49	0.35	9.0457101581727803E-2	Lung cancer
ebi-a-GCST90019017	IVW	64.98	0.28	9.1967130795348906E-2	LUSC
ebi-a-GCST90029014	IVW	141.28	0.20	9.3985333491666795E-2	LUSC
eqtl-a-ENSG00000162736	MR Egger	17.66	0.34	9.4149690569918607E-2	LUSC
ukb-a-248	IVW	402.25	0.08	9.5097346834875696E-2	SCLC
ukb-b-20188	MR Egger	545.41	0.05	9.6100169469435295E-2	SCLC
ukb-a-248	MR Egger	401.70	0.08	9.6335248915135702E-2	SCLC
ebi-a-GCST90104006	MR Egger	85.24	0.24	9.6700388780013996E-2	LUSC
ukb-b-20188	IVW	547.06	0.05	9.6985748187692802E-2	SCLC
ebi-a-GCST90014020	IVW	422.23	0.07	9.76544500097076E-2	SCLC
ukb-d-20116_0	IVW	68.74	0.26	9.8007043594162693E-2	Lung cancer
ukb-a-505	MR Egger	17.75	0.34	9.8560216753304106E-2	Lung cancer
ebi-a-GCST90029014	IVW	142.01	0.19	9.8677355626375293E-2	SCLC
ebi-a-GCST90014020	MR Egger	422.23	0.07	0.100014863794422	SCLC
eqtl-a-ENSG00000101695	MR Egger	1.11	0.29	0.100222630529093	Lung cancer
ebi-a-GCST90029014	MR Egger	141.21	0.18	0.100649589857167	SCLC
ebi-a-GCST90029014	MR Egger	141.26	0.18	0.100969249370489	LUSC
ebi-a-GCST90018934	IVW	52.31	0.28	0.10147389951718799	LUSC
ebi-a-GCST90018890	IVW	3.35	0.34	0.105799791203244	LUSC
eqtl-a-ENSG00000196812	IVW	2.24	0.33	0.105906067050485	Lung cancer
ukb-a-398	IVW	63.78	0.25	0.106327133449942	SCLC
ukb-b-8338	IVW	633.85	0.02	0.107040566038502	SCLC
ukb-b-8338	MR Egger	632.85	0.02	0.107211002199276	SCLC
ebi-a-GCST90029013	MR Egger	259.97	0.10	0.10757824464080699	Lung cancer
eqtl-a-ENSG00000167483	MR Egger	4.49	0.34	0.109499911202305	Lung cancer
ebi-a-GCST90029014	IVW	140.48	0.16	0.110190647835917	LUAD
prot-c-5102_55_3	IVW	1.12	0.29	0.110823890718545	LUSC
ebi-a-GCST90029014	MR Egger	139.64	0.16	0.11201125096079299	LUAD
finn-b-DM_SEVERAL_COMPLICATIONS	IVW	7.89	0.34	0.113127827290485	SCLC
ukb-b-7212	IVW	639.46	0.02	0.113309802831665	SCLC
ukb-a-398	IVW	64.32	0.24	0.11373795306482599	LUSC

ukb-b-7212	MR Egger	638.70	0.02	0.11381865230676801	SCLC
ebi-a-GCST90013975	IVW	538.28	0.03	0.113849329788865	SCLC
finn-b-F5_DEMENTIA	MR Egger	13.55	0.33	0.114278706696021	LUSC
ebi-a-GCST90029013	IVW	263.18	0.08	0.11466830806821	Lung cancer
eqtl-a-ENSG00000197077	IVW	13.56	0.33	0.114864446766031	Lung cancer
ebi-a-GCST90013975	MR Egger	537.87	0.03	0.11502562429313901	SCLC
ukb-b-17685	MR Egger	12.44	0.33	0.116027413919675	LUSC
ukb-a-279	MR Egger	366.81	0.05	0.116708724794662	SCLC
ukb-b-5192	IVW	122.48	0.16	0.118223732292818	LUSC
ukb-a-237	MR Egger	6.81	0.34	0.118715625486755	LUAD
ebi-a-GCST90018992	IVW	2.27	0.32	0.119030457659446	LUSC
ukb-b-9405	IVW	505.21	0.03	0.119179911719548	SCLC
ukb-b-9405	MR Egger	504.82	0.02	0.120474580100237	SCLC
ukb-a-279	IVW	370.21	0.04	0.12211722479788401	SCLC
ukb-b-19393	MR Egger	671.99	0.01	0.123501531599465	SCLC
ukb-b-12039	IVW	844.88	0.00	0.124133420401113	SCLC
ukb-b-7460	MR Egger	11.42	0.33	0.124302943569795	SCLC
ukb-b-5192	MR Egger	122.20	0.15	0.124393233086523	LUSC
ukb-b-12039	MR Egger	844.04	0.00	0.12445198981003699	SCLC
ukb-b-19393	IVW	674.08	0.01	0.124728396875265	SCLC
ieu-b-5117	IVW	182.86	0.10	0.12498989864497501	LUSC
ukb-b-17729	IVW	98.33	0.17	0.12538895053684099	LUSC
eqtl-a-ENSG00000101695	IVW	2.29	0.32	0.12754930727675001	Lung cancer
eqtl-a-ENSG00000149573	MR Egger	29.81	0.28	0.12793159937833001	LUAD
ukb-b-16489	IVW	338.41	0.04	0.12828246958398301	SCLC
ukb-d-30710_raw	MR Egger	66.56	0.21	0.12864553550101701	SCLC
ieu-b-5117	MR Egger	182.60	0.10	0.12925719056357099	LUSC
ukb-b-17729	MR Egger	97.63	0.16	0.12939344423565599	LUSC
ukb-b-6591	IVW	245.81	0.07	0.129405111571395	LUSC
ieu-b-142	MR Egger	24.13	0.29	0.129876468794099	LUSC
ieu-b-25	MR Egger	24.13	0.29	0.129876468794099	LUSC
ukb-b-8338	IVW	645.52	0.01	0.13093883171736301	LUSC
ukb-b-16489	MR Egger	338.38	0.04	0.13115981173800301	SCLC
eqtl-a-ENSG00000227598	IVW	5.75	0.33	0.131188381998529	Lung cancer
ukb-b-12854	MR Egger	564.23	0.01	0.13156548749990499	SCLC
ebi-a-GCST90000047	IVW	238.50	0.07	0.132087378032685	LUSC
eqtl-a-ENSG00000111725	MR Egger	13.83	0.31	0.132152216502539	LUSC
ukb-b-8338	MR Egger	645.39	0.01	0.13230570210063999	LUSC
ukb-b-6591	MR Egger	245.81	0.06	0.13347142232986201	LUSC
ukb-b-15169	MR Egger	5.77	0.33	0.13389335489953599	LUSC
ukb-b-12854	IVW	567.15	0.01	0.134265638464065	SCLC
ukb-b-15169	MR Egger	4.62	0.33	0.13435949829079599	Lung cancer
eqtl-a-ENSG00000111906	MR Egger	17.33	0.30	0.13438201936445199	Lung cancer
eqtl-a-ENSG00000184056	IVW	8.09	0.32	0.134498580841866	Lung cancer
ukb-a-249	MR Egger	532.66	0.01	0.13452621952906199	LUSC
ebi-a-GCST90000050	IVW	68.23	0.19	0.13525911018224501	LUSC

ukb-a-399	IVW	57.85	0.21	0.135686112582761	Lung cancer
ukb-a-249	IVW	534.54	0.01	0.13570299349766701	LUSC
ukb-b-11842	MR Egger	880.82	0.00	0.13603324961996099	SCLC
ukb-b-11842	IVW	882.17	0.00	0.13621779981941301	SCLC
ebi-a-GCST90000047	MR Egger	238.50	0.06	0.13627257016034999	LUSC
ukb-b-6019	IVW	8.11	0.32	0.13692367300716801	LUAD
ieu-a-835	IVW	79.98	0.17	0.137255489520202	SCLC
ieu-a-835	MR Egger	78.82	0.17	0.13731827755302101	SCLC
ukb-a-217	IVW	1.16	0.28	0.13766240437376201	Lung cancer
ukb-a-287	MR Egger	386.63	0.02	0.13870166329705499	LUSC
ukb-a-287	IVW	388.11	0.02	0.139418972981429	LUSC
ukb-b-19379	IVW	754.49	0.00	0.139812806525658	LUSC
ukb-b-19379	MR Egger	753.81	0.00	0.140370896067156	LUSC
prot-a-2481	IVW	1.16	0.28	0.140519532994032	LUSC
ukb-b-15590	MR Egger	645.03	0.00	0.14112715084841601	LUSC
ebi-a-GCST90000045	IVW	85.03	0.16	0.141512133366412	SCLC
ebi-a-GCST90025994	IVW	671.39	0.00	0.142074930015503	SCLC
ukb-b-7859	MR Egger	733.24	0.00	0.14216782737841399	LUSC
ukb-a-382	IVW	293.85	0.04	0.14243147926559899	LUSC
ebi-a-GCST90029012	IVW	267.05	0.04	0.14247683030462899	SCLC
ukb-b-7859	IVW	734.83	0.00	0.142656737970007	LUSC
ukb-b-15590	IVW	647.45	0.00	0.14278469326293899	LUSC
ukb-d-30710_raw	IVW	68.84	0.18	0.14288789546036401	SCLC
ukb-a-399	MR Egger	57.19	0.20	0.14321348427841099	Lung cancer
ebi-a-GCST90025994	MR Egger	671.38	0.00	0.14355650300607301	SCLC
ieu-a-1001	IVW	72.42	0.17	0.143912482663519	SCLC
ukb-a-382	MR Egger	293.52	0.03	0.14485789936381599	LUSC
ebi-a-GCST90029012	MR Egger	266.84	0.04	0.145540092218859	SCLC
ieu-a-1001	MR Egger	71.67	0.16	0.14884437593147501	SCLC
ebi-a-GCST90029007	IVW	833.04	0.00	0.14890092834393501	LUSC
ebi-a-GCST90000050	MR Egger	68.18	0.17	0.14929266663583801	LUSC
ebi-a-GCST90029007	MR Egger	832.27	0.00	0.14930948947827499	LUSC
ukb-b-12039	MR Egger	878.19	0.00	0.14938753400792201	LUSC
ieu-b-4877	IVW	92.97	0.13	0.15022042101744301	Lung cancer
ukb-b-12039	IVW	880.39	0.00	0.150377527131174	LUSC
ebi-a-GCST90000045	MR Egger	84.76	0.14	0.15051623677589099	SCLC
ieu-b-40	IVW	931.58	0.00	0.15090085470332201	SCLC
ebi-a-GCST90018949	IVW	744.88	0.00	0.15154117723013399	SCLC
ieu-b-40	MR Egger	931.31	0.00	0.151729354860732	SCLC
ieu-b-40	IVW	934.88	0.00	0.15176468831881401	LUSC
ukb-a-205	IVW	2.36	0.31	0.15190391238356199	LUSC
ebi-a-GCST90014020	MR Egger	441.03	0.01	0.15199054509836801	LUSC
ebi-a-GCST90014020	IVW	442.51	0.01	0.152556989428936	LUSC
ieu-b-40	MR Egger	934.65	0.00	0.15262320265109999	LUSC
ebi-a-GCST90018949	MR Egger	744.86	0.00	0.15286136863428701	SCLC
ukb-b-18096	IVW	641.59	0.00	0.15366847808352799	SCLC

ieu-a-1001	IVW	70.91	0.16	0.15380125505528899	LUSC
ubm-b-289	MR Egger	2.36	0.31	0.154325751081864	Lung cancer
ukb-b-11268	IVW	37.86	0.22	0.15468356084837501	Lung cancer
ukb-b-18096	MR Egger	641.57	0.00	0.15520005200366899	SCLC
ebi-a-GCST90018947	IVW	620.40	0.00	0.155379202525216	SCLC
ukb-b-10831	MR Egger	8.29	0.31	0.155753769309246	SCLC
ebi-a-GCST90018947	MR Egger	620.39	0.00	0.15698508084485399	SCLC
ebi-a-GCST90029014	IVW	150.80	0.07	0.15780146116219801	Lung cancer
ukb-a-434	MR Egger	8.32	0.30	0.15899592742101201	Lung cancer
ukb-b-1489	MR Egger	60.69	0.17	0.15969285372685699	LUSC
eqtl-a-ENSG00000174749	IVW	9.52	0.30	0.15980528128169499	Lung cancer
ieu-b-4877	MR Egger	92.95	0.12	0.16079916259240901	Lung cancer
ieu-b-5118	IVW	549.90	0.00	0.16166116469372199	SCLC
ebi-a-GCST90029014	MR Egger	150.34	0.07	0.16187871624094399	Lung cancer
ieu-a-1001	MR Egger	70.40	0.15	0.16195623256759001	LUSC
ukb-a-397	IVW	219.63	0.04	0.16223129095018901	SCLC
ieu-b-5118	MR Egger	549.13	0.00	0.16230452484867799	SCLC
ukb-a-397	MR Egger	218.90	0.04	0.16400901827768999	SCLC
ukb-a-397	MR Egger	233.89	0.03	0.166287197143867	LUAD
ukb-b-9405	IVW	532.40	0.00	0.16791471532567601	LUSC
ukb-b-9405	MR Egger	531.66	0.00	0.16864603954766599	LUSC
ukb-b-2303	IVW	748.50	0.00	0.16900919217833199	LUSC
ukb-a-272	IVW	438.05	0.00	0.16905294000708301	Lung cancer
ukb-b-2303	MR Egger	747.82	0.00	0.16958816230388199	LUSC
ukb-b-5192	IVW	128.90	0.07	0.16987827542457401	Lung cancer
ukb-a-272	MR Egger	438.05	0.00	0.17133111056210301	Lung cancer
ukb-b-11842	MR Egger	934.91	0.00	0.172113596196494	LUSC
ukb-a-283	IVW	386.55	0.01	0.17215823129798299	LUSC
ukb-a-279	IVW	401.06	0.01	0.17218829769000699	LUSC
ukb-b-18377	IVW	566.57	0.00	0.172211155626771	SCLC
eqtl-a-ENSG00000162736	IVW	20.54	0.25	0.17248081163679899	LUSC
ukb-a-283	MR Egger	385.71	0.01	0.17295028540447499	LUSC
ukb-b-11268	MR Egger	37.50	0.20	0.17323197539226701	Lung cancer
ukb-b-18377	MR Egger	566.07	0.00	0.17324263229295001	SCLC
ukb-b-11842	IVW	937.47	0.00	0.173309693179686	LUSC
ebi-a-GCST90000050	IVW	66.57	0.14	0.173837789896681	Lung cancer
ukb-a-279	MR Egger	400.98	0.01	0.174521485903185	LUSC
ukb-b-19393	IVW	707.61	0.00	0.17468617915422399	LUSC
ukb-b-19393	MR Egger	706.82	0.00	0.17518452634569301	LUSC
ukb-a-382	IVW	304.32	0.01	0.17521623083864701	SCLC
ebi-a-GCST90013870	MR Egger	652.33	0.00	0.175261456083742	LUSC
ukb-b-6704	IVW	701.58	0.00	0.17614089610486899	LUSC
ukb-a-180	IVW	2.43	0.30	0.17662594323561301	Lung cancer
ukb-a-397	IVW	238.11	0.02	0.17685439692441299	LUAD
ukb-b-6704	MR Egger	701.08	0.00	0.176979080772943	LUSC
ukb-b-5192	MR Egger	128.83	0.07	0.177199127813398	Lung cancer

ukb-a-382	MR Egger	303.91	0.01	0.17737478876311699	SCLC
ebi-a-GCST90013870	IVW	655.22	0.00	0.177375798389415	LUSC
ebi-a-GCST90018947	IVW	634.56	0.00	0.177388854838646	LUSC
ebi-a-GCST90018947	MR Egger	633.50	0.00	0.17758568792947099	LUSC
ukb-b-1489	IVW	63.30	0.14	0.17857316142638299	LUSC
ebi-a-GCST90000047	IVW	245.97	0.02	0.17876595064265799	SCLC
ukb-b-20531	IVW	578.45	0.00	0.17883344897056799	SCLC
ieu-b-5118	MR Egger	553.24	0.00	0.17938299924895099	LUSC
ukb-b-20531	MR Egger	577.65	0.00	0.17943718299283201	SCLC
ieu-b-5118	IVW	554.59	0.00	0.17957340523462101	LUSC
ukb-a-248	IVW	439.11	0.00	0.18016747842151201	LUSC
prot-a-3203	MR Egger	3.66	0.30	0.18031220023667199	LUSC
ukb-a-248	MR Egger	438.54	0.00	0.18137937166961399	LUSC
ukb-a-287	MR Egger	407.09	0.00	0.18200531012954399	SCLC
ebi-a-GCST90013974	MR Egger	655.79	0.00	0.18266261126984201	LUSC
ebi-a-GCST90000047	MR Egger	245.95	0.02	0.18276465069745099	SCLC
ebi-a-GCST90012877	IVW	60.00	0.13	0.18338822739900501	LUSC
ukb-b-6591	MR Egger	502.32	0.00	0.18378574813121401	LUAD
ukb-a-287	IVW	409.24	0.00	0.183854716161824	SCLC
ebi-a-GCST90013974	IVW	658.19	0.00	0.184129934510528	LUSC
finn-b-RHEU_ARTHRITIS_OTH	IVW	3.68	0.30	0.18478806768233899	Lung cancer
ebi-a-GCST90012794	MR Egger	25.76	0.22	0.18492878480379299	Lung cancer
ukb-b-20044	IVW	704.90	0.00	0.18570491535650399	SCLC
ukb-b-20044	MR Egger	703.75	0.00	0.185796056533341	SCLC
ukb-a-283	MR Egger	389.36	0.00	0.18583493628589001	SCLC
ukb-b-6591	IVW	505.18	0.00	0.186436148063507	LUAD
ukb-b-20044	MR Egger	710.50	0.00	0.18648440450179701	LUSC
ukb-b-19953	MR Egger	774.84	0.00	0.18692697423325599	LUSC
ukb-a-273	IVW	471.12	0.00	0.18703648188049399	Lung cancer
ukb-a-275	IVW	395.05	0.00	0.18745107708518999	SCLC
ukb-b-19953	IVW	776.60	0.00	0.187482141254117	LUSC
ukb-a-283	IVW	391.48	0.00	0.187707655512836	SCLC
ukb-a-275	MR Egger	394.09	0.00	0.187993545803703	SCLC
ebi-a-GCST90000050	MR Egger	66.54	0.12	0.18847210463043099	Lung cancer
eqtl-a-ENSG00000168411	MR Egger	4.93	0.29	0.18864330263285101	SCLC
ukb-b-20044	IVW	713.66	0.00	0.18868499067983699	LUSC
ukb-d-20116_0	MR Egger	83.83	0.09	0.18883963691862099	LUSC
ukb-a-273	MR Egger	470.94	0.00	0.18885197165512899	Lung cancer
ukb-b-20188	IVW	613.07	0.00	0.18931933252404501	LUSC
ukb-a-398	IVW	75.25	0.10	0.189333666386649	Lung cancer
ukb-b-20188	MR Egger	612.50	0.00	0.190205767752581	LUSC
ukb-d-20116_0	IVW	85.23	0.09	0.19042031951938301	LUSC
ukb-a-398	MR Egger	74.11	0.10	0.19042833231933701	Lung cancer
ukb-a-249	MR Egger	1119.54	0.00	0.19073733466142201	SCLC
ieu-a-1239	IVW	482.07	0.00	0.19098618525059399	Lung cancer
ebi-a-GCST004604	IVW	139.69	0.04	0.19104920759664501	Lung cancer

ukb-b-7212	IVW	698.47	0.00	0.191085505799374	LUSC
ukb-b-7212	MR Egger	697.33	0.00	0.19120099794932	LUSC
ukb-b-19921	IVW	951.17	0.00	0.19152046244793899	LUSC
ukb-b-19921	MR Egger	950.06	0.00	0.191632697846954	LUSC
ieu-a-1239	MR Egger	482.07	0.00	0.193057042066857	Lung cancer
ukb-b-11615	IVW	94.28	0.08	0.193874717998229	SCLC
ebi-a-GCST90025994	IVW	711.32	0.00	0.19445655820367799	LUSC
ebi-a-GCST90025994	MR Egger	710.46	0.00	0.19488393080233801	LUSC
ukb-a-278	MR Egger	330.64	0.00	0.19550114465014301	SCLC
ukb-b-16407	IVW	603.13	0.00	0.19586742621901099	LUSC
ukb-a-278	IVW	332.06	0.00	0.1959286998709	SCLC
ukb-b-16407	MR Egger	601.95	0.00	0.19594520279658401	LUSC
ukb-a-249	IVW	1128.10	0.00	0.195992288117497	SCLC
ebi-a-GCST006368	IVW	211.50	0.02	0.19621725106660001	SCLC
ebi-a-GCST004604	MR Egger	139.40	0.04	0.196584599002533	Lung cancer
ukb-a-275	IVW	412.85	0.00	0.198252532236845	LUSC
ebi-a-GCST90025972	IVW	754.01	0.00	0.19894741858502901	Lung cancer
ebi-a-GCST90025972	MR Egger	752.88	0.00	0.19908032922154301	Lung cancer
ebi-a-GCST90012877	MR Egger	59.98	0.12	0.19969386511387999	LUSC
ebi-a-GCST90018949	IVW	800.14	0.00	0.20014429460594901	LUSC
ukb-a-275	MR Egger	412.66	0.00	0.20030245415793199	LUSC
ebi-a-GCST006368	MR Egger	211.35	0.01	0.20038152320723801	SCLC
ebi-a-GCST90018949	MR Egger	799.34	0.00	0.20059444216302499	LUSC
ieu-a-1239	MR Egger	497.66	0.00	0.20226318580768901	LUAD
finn-b-D3_ANAEMIA_B12_DEF	IVW	2.51	0.29	0.20237384421335899	Lung cancer
ieu-a-1239	IVW	499.17	0.00	0.202675628652975	LUAD
ukb-a-201	IVW	20.10	0.22	0.20405541624661899	LUSC
ukb-b-11615	MR Egger	94.24	0.07	0.20414940076285101	SCLC
ukb-a-201	IVW	17.60	0.23	0.20451333262179899	Lung cancer
eqtl-a-ENSG00000184056	MR Egger	7.55	0.27	0.205615152068895	Lung cancer
ukb-a-328	MR Egger	5.04	0.28	0.20615408195101101	LUAD
ukb-b-8909	MR Egger	636.46	0.00	0.206544399869344	SCLC
ukb-a-201	MR Egger	18.91	0.22	0.20670141387028301	LUSC
ukb-b-17670	IVW	3.79	0.29	0.207734373810569	Lung cancer
ukb-b-6134	IVW	44.25	0.14	0.20895117622883599	LUSC
ubm-b-1571	IVW	2.53	0.28	0.209618319267209	Lung cancer
ukb-b-8909	IVW	649.56	0.00	0.21023812203467099	LUSC
ukb-b-8909	IVW	641.28	0.00	0.210954698605235	SCLC
ebi-a-GCST90013975	IVW	613.79	0.00	0.21146040403331401	LUSC
ukb-b-8909	MR Egger	649.35	0.00	0.21152208331990299	LUSC
ebi-a-GCST90000046	IVW	79.92	0.07	0.211669623436154	Lung cancer
ebi-a-GCST90013975	MR Egger	613.78	0.00	0.213076668298185	LUSC
ukb-a-286	MR Egger	352.10	0.00	0.213295980583566	SCLC
eqtl-a-ENSG00000186470	IVW	12.71	0.24	0.213312484766432	SCLC
ukb-b-18096	MR Egger	693.90	0.00	0.214581437987651	LUSC
ukb-b-12854	MR Egger	636.64	0.00	0.21462738816947299	LUSC

ukb-b-12854	IVW	638.44	0.00	0.21527676149849201	LUSC
ukb-a-278	IVW	351.88	0.00	0.215631884950826	LUSC
ukb-b-18096	IVW	696.57	0.00	0.216154766976872	LUSC
ukb-a-286	IVW	354.79	0.00	0.21643697363412501	SCLC
ebi-a-GCST90000046	MR Egger	79.18	0.07	0.21698517284139299	Lung cancer
ukb-a-278	MR Egger	351.78	0.00	0.21825655935472199	LUSC
ukb-a-5	MR Egger	62.87	0.09	0.22055613900166701	Lung cancer
eqtl-a-ENSG00000111725	IVW	16.70	0.21	0.22156559201842399	LUSC
eqtl-a-ENSG00000149573	MR Egger	34.78	0.14	0.223677938828237	Lung cancer
ebi-a-GCST90000048	MR Egger	71.26	0.07	0.228166424779187	SCLC
ukb-a-5	IVW	64.83	0.08	0.228777958709455	Lung cancer
eqtl-a-ENSG00000174749	MR Egger	9.08	0.25	0.22891044018368401	Lung cancer
ukb-b-6134	MR Egger	44.14	0.11	0.229776094950914	LUSC
ukb-a-265	IVW	437.68	0.00	0.23002546214571601	LUSC
ukb-b-18377	MR Egger	620.16	0.00	0.23084571590434499	LUSC
ukb-a-265	MR Egger	437.30	0.00	0.23164420151225701	LUSC
ukb-b-18377	IVW	623.45	0.00	0.23330165433796701	LUSC
ukb-b-11615	IVW	100.49	0.04	0.233753804030853	Lung cancer
ukb-a-291	MR Egger	444.44	0.00	0.234997507300549	LUSC
ukb-a-291	IVW	445.96	0.00	0.23535430007038499	LUSC
ubm-b-847	MR Egger	2.62	0.27	0.23537312765592899	Lung cancer
eqtl-a-ENSG00000149573	IVW	35.31	0.13	0.23538191789576099	LUAD
ebi-a-GCST90000048	IVW	73.25	0.06	0.23551010460400901	SCLC
ukb-b-8755	MR Egger	10.47	0.23	0.23575694250382201	LUSC
ukb-a-290	MR Egger	347.24	0.00	0.23684015825335999	SCLC
ukb-b-19379	IVW	857.76	0.00	0.237548768814699	Lung cancer
ukb-b-19379	MR Egger	857.74	0.00	0.23869543329815601	Lung cancer
ukb-a-274	IVW	378.97	0.00	0.2400392430046	LUSC
ebi-a-GCST90018990	IVW	151.43	0.01	0.24057234315345499	LUSC
ukb-a-274	MR Egger	378.90	0.00	0.242552439290255	LUSC
ukb-b-11615	MR Egger	100.43	0.03	0.243280588110314	Lung cancer
ebi-a-GCST006250	IVW	228.95	0.00	0.24437794825015099	Lung cancer
ebi-a-GCST90029012	MR Egger	319.05	0.00	0.244632961706261	LUAD
ukb-b-16489	MR Egger	413.55	0.00	0.245559594890324	LUAD
ebi-a-GCST90018990	MR Egger	151.40	0.01	0.247020292180054	LUSC
ebi-a-GCST006250	MR Egger	228.52	0.00	0.24732471651998	Lung cancer
ukb-a-290	IVW	353.47	0.00	0.24745992709905701	SCLC
ukb-a-291	IVW	447.98	0.00	0.24774025410460401	SCLC
ukb-a-291	MR Egger	447.19	0.00	0.24864039840369401	SCLC
ebi-a-GCST006250	IVW	229.17	0.00	0.249468180238552	LUSC
eqtl-a-ENSG00000026036	IVW	1.33	0.25	0.25048914696966701	LUAD
ukb-a-282	MR Egger	366.13	0.00	0.25162607177074497	SCLC
ukb-b-20531	MR Egger	652.49	0.00	0.252100127134256	LUSC
ebi-a-GCST90016675	IVW	5.35	0.25	0.25260653112632903	Lung cancer
ebi-a-GCST006250	MR Egger	229.14	0.00	0.25371805604304298	LUSC
ukb-a-265	IVW	453.17	0.00	0.25414814481859099	SCLC

ukb-a-282	IVW	368.85	0.00	0.25443807482509201	SCLC
ukb-a-265	MR Egger	452.10	0.00	0.254588848153247	SCLC
eqtl-a-ENSG00000107890	IVW	5.37	0.25	0.25546823410726599	LUSC
ukb-b-16489	IVW	420.66	0.00	0.25592533160523401	LUAD
ukb-b-20531	IVW	657.55	0.00	0.25633021675750201	LUSC
ebi-a-GCST90014021	IVW	633.20	0.00	0.25773791985787597	SCLC
ukb-a-328	IVW	6.74	0.24	0.25842289504246801	LUAD
ebi-a-GCST90014021	MR Egger	632.86	0.00	0.25891631589978797	SCLC
ukb-a-201	MR Egger	17.57	0.17	0.26005657045758301	Lung cancer
ebi-a-GCST90029012	IVW	329.11	0.00	0.264687506925677	LUAD
ebi-a-GCST90012794	IVW	29.96	0.12	0.26575782497795197	Lung cancer
ebi-a-GCST90000047	MR Egger	557.38	0.00	0.27159852914419502	LUAD
ukb-a-264	IVW	407.07	0.00	0.27286059770016502	LUSC
ebi-a-GCST90029025	IVW	1414.13	0.00	0.27376031252630101	Lung cancer
ebi-a-GCST90029025	MR Egger	1412.84	0.00	0.27380218526697597	Lung cancer
eqtl-a-ENSG00000169045	IVW	12.40	0.19	0.27397915302850501	Lung cancer
ukb-b-19921	IVW	1074.59	0.00	0.27413979048465298	Lung cancer
ukb-b-12405	MR Egger	42.73	0.08	0.27453959773877701	Lung cancer
ebi-a-GCST90000047	IVW	561.09	0.00	0.27462488592648499	LUAD
ukb-b-12405	IVW	44.13	0.08	0.27483563597260802	Lung cancer
ukb-b-19921	MR Egger	1074.49	0.00	0.27500484174443102	Lung cancer
ukb-a-264	MR Egger	407.07	0.00	0.27530873219822699	LUSC
ukb-b-16446	MR Egger	1256.90	0.00	0.27599422021890602	Lung cancer
ieu-a-1126	IVW	207.23	0.00	0.27616392544629598	LUAD
ukb-b-16446	IVW	1259.63	0.00	0.27677158838064703	Lung cancer
ieu-a-1126	MR Egger	206.03	0.00	0.27680218406045298	LUAD
ebi-a-GCST011365	MR Egger	90.04	0.02	0.27806513796247301	LUSC
eqtl-a-ENSG00000041357	IVW	1.39	0.24	0.28007739804637699	Lung cancer
ukb-b-16489	MR Egger	418.74	0.00	0.28118228654857202	Lung cancer
ukb-b-6306	IVW	151.74	0.00	0.28168677858839503	Lung cancer
ukb-a-282	IVW	393.46	0.00	0.28583068464595301	LUSC
ukb-b-16698	MR Egger	1137.79	0.00	0.28721677690179098	Lung cancer
ukb-a-282	MR Egger	393.21	0.00	0.28791363895807498	LUSC
ukb-b-6306	MR Egger	151.72	0.00	0.28818141251879198	Lung cancer
ukb-b-16698	IVW	1141.17	0.00	0.28844921881429397	Lung cancer
ukb-b-19925	MR Egger	1183.14	0.00	0.290868848124126	Lung cancer
ukb-b-19925	IVW	1184.80	0.00	0.29102175567699001	Lung cancer
eqtl-a-ENSG00000186470	MR Egger	12.70	0.18	0.29150166391378801	SCLC
ukb-a-274	IVW	384.04	0.00	0.29174006643686601	SCLC
ukb-b-16489	IVW	426.52	0.00	0.29193662112060698	Lung cancer
eqtl-a-ENSG00000227598	MR Egger	5.65	0.23	0.29257253819432399	Lung cancer
ieu-b-40	IVW	1113.12	0.00	0.29297748137972002	Lung cancer
ukb-a-274	MR Egger	383.50	0.00	0.29334397033021498	SCLC
ieu-b-40	MR Egger	1112.71	0.00	0.29361384422449099	Lung cancer
ieu-b-4879	MR Egger	672.46	0.00	0.29363605158321399	LUAD
ukb-b-6591	IVW	297.54	0.00	0.29757648487396299	SCLC

ebi-a-GCST90000045	IVW	99.78	0.01	0.29847199850678102	Lung cancer
ebi-a-GCST90000514	MR Egger	91.49	0.01	0.30045940460492099	SCLC
ukb-a-382	IVW	371.71	0.00	0.30052939035156501	Lung cancer
ukb-b-19520	MR Egger	1139.77	0.00	0.30073358728086702	Lung cancer
ukb-b-19520	IVW	1141.29	0.00	0.30078833044783998	Lung cancer
eqtl-a-ENSG00000149573	IVW	40.05	0.07	0.30079947445816002	Lung cancer
ukb-b-6591	MR Egger	297.53	0.00	0.30090883252964301	SCLC
ukb-a-382	MR Egger	370.64	0.00	0.30120224173170301	Lung cancer
ebi-a-GCST90000045	MR Egger	98.87	0.01	0.30214239160175799	Lung cancer
ebi-a-GCST90000047	IVW	290.14	0.00	0.30378809679404001	Lung cancer
finn-b-I9_CORATHER	MR Egger	31.66	0.08	0.30514438504296898	LUSC
ukb-b-9093	MR Egger	1178.69	0.00	0.30516108758754101	Lung cancer
ukb-b-9093	IVW	1180.52	0.00	0.30538842630673801	Lung cancer
ebi-a-GCST90000047	MR Egger	289.96	0.00	0.30680146373258099	Lung cancer
ukb-b-17729	IVW	124.49	0.00	0.30915835597542302	Lung cancer
ukb-b-12039	IVW	1077.57	0.00	0.31048440131347999	Lung cancer
ukb-b-12039	MR Egger	1076.71	0.00	0.31086312083902801	Lung cancer
ukb-a-281	MR Egger	700.88	0.00	0.31229740652355298	Lung cancer
ukb-b-17729	MR Egger	123.87	0.00	0.31381598250249798	Lung cancer
ukb-a-281	IVW	704.14	0.00	0.31405850964169502	Lung cancer
ukb-a-397	MR Egger	274.17	0.00	0.31430221732289298	Lung cancer
ukb-b-469	MR Egger	1.46	0.23	0.31487828739060197	LUAD
ebi-a-GCST009971	IVW	17.53	0.13	0.31534965897430101	LUSC
finn-b-I9_CORATHER	IVW	33.60	0.07	0.31553840864843302	LUSC
ebi-a-GCST90018793	MR Egger	67.24	0.02	0.31591897895360699	LUSC
ukb-a-397	IVW	276.93	0.00	0.317519313926932	Lung cancer
ukb-a-249	MR Egger	675.53	0.00	0.31757407738711002	Lung cancer
ukb-a-264	IVW	432.73	0.00	0.31828281444530498	SCLC
ukb-d-I9_IHD	IVW	32.28	0.07	0.318501118110256	LUSC
ebi-a-GCST007432	IVW	412.35	0.00	0.31853993499600503	LUSC
ebi-a-GCST90000514	IVW	95.40	0.01	0.31865972642401003	SCLC
ukb-a-249	IVW	678.30	0.00	0.318885124459383	Lung cancer
ebi-a-GCST007432	MR Egger	411.52	0.00	0.31959813087660099	LUSC
ukb-a-264	MR Egger	432.10	0.00	0.31960149288795697	SCLC
eqtl-a-ENSG00000138593	IVW	1.47	0.23	0.32050432759825498	LUAD
ebi-a-GCST90018949	IVW	949.45	0.00	0.32065694593129002	Lung cancer
ebi-a-GCST90029007	IVW	1043.97	0.00	0.32086239452460202	Lung cancer
ebi-a-GCST90018949	MR Egger	948.70	0.00	0.32117293108075101	Lung cancer
ebi-a-GCST90029007	MR Egger	1043.44	0.00	0.32147580001223303	Lung cancer
ebi-a-GCST90018982	IVW	32.46	0.07	0.32219414118203699	Lung cancer
ukb-b-8338	MR Egger	841.35	0.00	0.32370637297827498	Lung cancer
ukb-b-19393	MR Egger	869.73	0.00	0.32392554015913799	Lung cancer
eqtl-a-ENSG00000159873	IVW	4.44	0.22	0.32418986291566598	LUSC
ebi-a-GCST90018793	IVW	69.64	0.02	0.32508565074298501	LUSC
ebi-a-GCST011365	IVW	97.80	0.01	0.32518625276608398	LUSC
ukb-b-8338	IVW	847.03	0.00	0.32705712565543199	Lung cancer

ukb-b-19393	IVW	875.79	0.00	0.32746112943208699	Lung cancer
ukb-b-2732	IVW	2.98	0.23	0.32830020689193601	SCLC
ebi-a-GCST90013975	MR Egger	715.99	0.00	0.32959975137301301	Lung cancer
ukb-b-15590	MR Egger	828.72	0.00	0.33150187972259598	Lung cancer
ebi-a-GCST90029012	MR Egger	350.08	0.00	0.331578210656852	Lung cancer
ebi-a-GCST90016675	MR Egger	4.49	0.21	0.33202183485682502	Lung cancer
ukb-b-18408	MR Egger	35.93	0.06	0.33206301468038901	LUSC
ukb-b-15590	IVW	830.98	0.00	0.33211590709890598	Lung cancer
ukb-b-18408	IVW	37.46	0.05	0.33269087846148199	LUSC
finn-b-I9_REVASC	IVW	43.50	0.04	0.333403305217852	LUSC
ebi-a-GCST004441	MR Egger	3.00	0.22	0.33378964990934001	LUAD
ebi-a-GCST90018947	IVW	791.15	0.00	0.333879484599027	Lung cancer
ukb-b-7212	IVW	851.47	0.00	0.33409223760879397	Lung cancer
ebi-a-GCST90013975	IVW	722.60	0.00	0.33434915997036602	Lung cancer
ebi-a-GCST90018947	MR Egger	790.37	0.00	0.33448850037522099	Lung cancer
ukb-b-7212	MR Egger	850.68	0.00	0.33465277021710799	Lung cancer
ukb-a-132	IVW	7.55	0.18	0.33753966965681298	LUSC
ukb-b-17670	MR Egger	3.02	0.22	0.33861049582650898	Lung cancer
ukb-a-278	MR Egger	409.77	0.00	0.33865911260174902	Lung cancer
ebi-a-GCST90029012	IVW	356.10	0.00	0.34007118736344599	Lung cancer
ieu-a-1001	MR Egger	92.54	0.01	0.340850933916375	Lung cancer
ebi-a-GCST009971	MR Egger	16.76	0.12	0.34366013341967	LUAD
ukb-b-18096	IVW	840.42	0.00	0.344371672294223	Lung cancer
ukb-b-18096	MR Egger	840.35	0.00	0.34550900604488999	Lung cancer
ukb-b-19953	IVW	978.96	0.00	0.34726631203837599	Lung cancer
ukb-b-2303	IVW	967.71	0.00	0.34794569043991702	Lung cancer
ukb-b-19953	MR Egger	978.80	0.00	0.34818330689264199	Lung cancer
ieu-a-1001	IVW	95.13	0.00	0.34828566387237803	Lung cancer
ukb-b-2303	MR Egger	967.50	0.00	0.34883639854488102	Lung cancer
ukb-d-I9_IHD	MR Egger	32.27	0.05	0.34919257293866701	LUSC
ieu-b-4879	IVW	732.17	0.00	0.34987417587854203	LUAD
ebi-a-GCST90014021	IVW	738.44	0.00	0.35268566957408198	Lung cancer
ukb-b-6704	MR Egger	907.00	0.00	0.35280806198881898	Lung cancer
ukb-a-278	IVW	420.36	0.00	0.35293939128684099	Lung cancer
ebi-a-GCST90018982	MR Egger	32.46	0.05	0.35299763647029297	Lung cancer
ukb-b-20044	MR Egger	907.82	0.00	0.35339761974482198	Lung cancer
ebi-a-GCST90014021	MR Egger	737.76	0.00	0.35344725622318002	Lung cancer
ebi-a-GCST90018982	IVW	34.03	0.05	0.35353533381205099	LUSC
ukb-a-277	MR Egger	763.02	0.00	0.353881332683043	Lung cancer
finn-b-I9_REVASC	MR Egger	43.34	0.03	0.35392138589356298	LUSC
ukb-b-6704	IVW	912.36	0.00	0.35551830382951	Lung cancer
ukb-b-20261	IVW	107.11	0.00	0.355817853647515	Lung cancer
ukb-b-20044	IVW	912.84	0.00	0.35585924096497501	Lung cancer
ukb-b-8755	IVW	13.98	0.12	0.356039297035669	LUSC
ukb-a-277	IVW	767.38	0.00	0.356248854977954	Lung cancer
ukb-b-20188	MR Egger	778.08	0.00	0.357395104046136	Lung cancer

ukb-a-291	MR Egger	541.95	0.00	0.357879041936841	Lung cancer
ukb-b-20261	MR Egger	106.52	0.00	0.36159473651124102	Lung cancer
ukb-b-20188	IVW	785.62	0.00	0.36228379193247001	Lung cancer
ebi-a-GCST009971	MR Egger	17.27	0.10	0.36302373840040503	LUSC
ukb-b-6019	MR Egger	9.43	0.15	0.36376868638429	LUSC
ukb-a-291	IVW	550.20	0.00	0.36568561849460202	Lung cancer
ebi-a-GCST90014020	IVW	601.52	0.00	0.36660876309177998	Lung cancer
ukb-b-7408	IVW	67.89	0.01	0.366639087903469	Lung cancer
ukb-b-1668	MR Egger	34.76	0.04	0.367062851698921	LUSC
ebi-a-GCST90018982	MR Egger	33.18	0.04	0.36717672514522898	LUSC
ebi-a-GCST90014020	MR Egger	601.00	0.00	0.36771964110503702	Lung cancer
ukb-b-16407	IVW	769.75	0.00	0.36992243371371902	Lung cancer
ukb-b-18377	IVW	756.46	0.00	0.37074954297124901	Lung cancer
ukb-b-16407	MR Egger	769.44	0.00	0.37096848273979599	Lung cancer
ukb-b-18377	MR Egger	756.35	0.00	0.37198633194259501	Lung cancer
ebi-a-GCST90013870	IVW	855.29	0.00	0.37214246299279002	Lung cancer
ebi-a-GCST90013870	MR Egger	855.29	0.00	0.37330882139482002	Lung cancer
ukb-a-248	IVW	580.90	0.00	0.37338201716524799	Lung cancer
ukb-b-12854	MR Egger	787.07	0.00	0.37362728854352401	Lung cancer
ukb-a-287	MR Egger	538.07	0.00	0.37368332309365898	Lung cancer
ebi-a-GCST90025994	IVW	922.32	0.00	0.37440521163703699	Lung cancer
ukb-b-20531	IVW	769.01	0.00	0.37452418117715403	Lung cancer
ukb-b-7408	MR Egger	67.15	0.01	0.37453414473826802	Lung cancer
ukb-a-283	MR Egger	513.58	0.00	0.374976167208111	Lung cancer
ukb-a-248	MR Egger	580.88	0.00	0.37509124069678101	Lung cancer
ebi-a-GCST90025994	MR Egger	922.20	0.00	0.37540751403567102	Lung cancer
ukb-b-2134	IVW	140.95	0.00	0.37568177580534401	Lung cancer
ieu-b-142	IVW	35.24	0.04	0.37573864439108001	LUSC
ieu-b-25	IVW	35.24	0.04	0.37573864439108001	LUSC
ukb-b-20531	MR Egger	768.92	0.00	0.37574425066586498	Lung cancer
ukb-a-287	IVW	542.11	0.00	0.37650545331142499	Lung cancer
eqtl-a-ENSG00000158406	MR Egger	36.91	0.03	0.37682149993268599	Lung cancer
ebi-a-GCST009971	IVW	19.27	0.08	0.37712532962662099	LUAD
ukb-a-283	IVW	517.39	0.00	0.37765023124493102	Lung cancer
ukb-b-2134	MR Egger	139.87	0.00	0.37799253377574998	Lung cancer
ukb-b-12854	IVW	794.43	0.00	0.37817089833303502	Lung cancer
ukb-a-274	MR Egger	450.01	0.00	0.38224258000151301	Lung cancer
eqtl-a-ENSG00000107890	MR Egger	4.86	0.18	0.38287968597115801	LUSC
ebi-a-GCST90013974	IVW	870.31	0.00	0.38297901402655699	Lung cancer
ebi-a-GCST90013974	MR Egger	870.29	0.00	0.38410986884263998	Lung cancer
finn-b-I9_ANGINA	IVW	27.61	0.05	0.38433896252375499	LUSC
ukb-b-8909	MR Egger	829.93	0.00	0.38789689926814303	Lung cancer
ukb-a-274	IVW	456.05	0.00	0.38823042291557902	Lung cancer
ukb-b-9405	IVW	725.20	0.00	0.38912987535263999	Lung cancer
ukb-b-9405	MR Egger	724.26	0.00	0.389723516406077	Lung cancer
ukb-b-8909	IVW	837.92	0.00	0.39254387974249699	Lung cancer

ukb-b-969	MR Egger	54.37	0.01	0.39307243158400101	Lung cancer
ieu-b-5117	MR Egger	257.10	0.00	0.393234392718109	Lung cancer
ukb-a-282	MR Egger	461.91	0.00	0.39382752344602001	Lung cancer
ieu-b-5117	IVW	259.76	0.00	0.395601252916091	Lung cancer
ieu-b-5118	IVW	760.34	0.00	0.39632279802465697	Lung cancer
ukb-b-969	IVW	56.38	0.01	0.39690906426330902	Lung cancer
ukb-a-286	MR Egger	471.46	0.00	0.397614743252376	Lung cancer
ieu-b-5118	MR Egger	760.34	0.00	0.39763758993163201	Lung cancer
ukb-a-286	IVW	474.14	0.00	0.39891160219550398	Lung cancer
ukb-a-282	IVW	469.48	0.00	0.40147082434585402	Lung cancer
finn-b-RHEU_ARTHRITIS_OTH	MR Egger	3.35	0.19	0.40214997388654899	Lung cancer
ukb-a-279	MR Egger	558.33	0.00	0.40358317496902901	Lung cancer
ukb-a-279	IVW	560.80	0.00	0.404426247347835	Lung cancer
ukb-a-275	MR Egger	567.58	0.00	0.41506422121643299	Lung cancer
ukb-a-265	MR Egger	585.62	0.00	0.41600019244648301	Lung cancer
finn-b-I9_CORATHER_EXNONE	IVW	35.99	0.02	0.41642994160980301	LUSC
ukb-a-275	IVW	571.58	0.00	0.41740794709358497	Lung cancer
ukb-a-265	IVW	589.54	0.00	0.41819054022733498	Lung cancer
finn-b-I9_ANGINA	MR Egger	27.55	0.04	0.419265943957635	LUSC
ukb-d-I9_CORATHER	IVW	41.36	0.02	0.41973724990642802	LUSC
ukb-b-6591	IVW	355.40	0.00	0.42037302244908498	Lung cancer
ukb-a-264	IVW	516.42	0.00	0.42294513278412599	Lung cancer
ukb-b-6591	MR Egger	355.40	0.00	0.423185986819967	Lung cancer
ukb-a-264	MR Egger	515.69	0.00	0.42407606042849999	Lung cancer
finn-b-I9_CABG_EXNONE	IVW	22.75	0.04	0.42846316881013502	LUSC
ukb-b-1668	IVW	40.29	0.01	0.429157849096654	LUSC
bbj-a-73	IVW	76.34	0.00	0.43672006387182599	Lung cancer
ieu-b-4879	MR Egger	284.11	0.00	0.43684618913675699	Lung cancer
finn-b-I9_CORATHER_EXNONE	MR Egger	35.76	0.02	0.440764320941745	LUSC
ukb-d-I9_CORATHER	MR Egger	41.36	0.01	0.44385554408458	LUSC
eqtl-a-ENSG00000158406	IVW	43.18	0.01	0.444198308585877	Lung cancer
eqtl-a-ENSG00000159873	MR Egger	3.63	0.16	0.44925462631235502	LUSC
bbj-a-73	MR Egger	76.34	0.00	0.449819588541986	Lung cancer
ieu-b-142	IVW	36.67	0.01	0.45459265477540201	SCLC
ieu-b-25	IVW	36.67	0.01	0.45459265477540201	SCLC
ieu-b-142	MR Egger	34.98	0.01	0.45690294732224002	SCLC
ieu-b-25	MR Egger	34.98	0.01	0.45690294732224002	SCLC
ukb-a-434	IVW	16.61	0.06	0.45799846725820498	LUSC
finn-b-I9_CABG_EXNONE	MR Egger	22.48	0.03	0.46630248673867802	LUSC
ukb-a-132	MR Egger	7.53	0.11	0.46885185244117	LUSC
ukb-b-7460	MR Egger	64.07	0.00	0.469292704758214	LUAD
prot-a-1347	IVW	1.92	0.17	0.47980756033099198	LUSC
ukb-a-434	MR Egger	15.46	0.05	0.482635774636059	LUSC
ieu-b-4879	IVW	313.35	0.00	0.48619897332798401	Lung cancer
ukb-a-237	MR Egger	13.64	0.06	0.48690759449552201	SCLC
finn-b-I9_CABG	IVW	33.66	0.01	0.49497695144293102	LUSC

ukb-b-17685	IVW	23.91	0.02	0.49806097337738298	LUSC
ebi-a-GCST90000514	IVW	143.49	0.00	0.49821225392579899	Lung cancer
ebi-a-GCST90013972	MR Egger	10.01	0.07	0.50067831833838705	SCLC
ebi-a-GCST90096909	IVW	80.30	0.00	0.50188949176181097	Lung cancer
ukb-a-328	MR Egger	8.03	0.09	0.501963608773906	LUSC
ebi-a-GCST90000514	MR Egger	143.20	0.00	0.50420364578994004	Lung cancer
ebi-a-GCST90013922	MR Egger	10.14	0.07	0.50699805000514298	SCLC
eqtl-a-ENSG00000134193	IVW	2.04	0.15	0.510304686667577	LUSC
ebi-a-GCST90096909	MR Egger	79.66	0.00	0.51044219059186702	Lung cancer
ubm-b-1571	MR Egger	2.05	0.15	0.51328663637889804	Lung cancer
eqtl-a-ENSG00000196812	IVW	4.15	0.13	0.51817795474659101	LUSC
finn-b-I9_CABG	MR Egger	33.63	0.01	0.52420619687778003	LUSC
ukb-a-205	MR Egger	2.14	0.14	0.53289755068135503	LUSC
ukb-a-328	IVW	10.75	0.06	0.53478254991453	LUSC
ukb-b-7460	MR Egger	23.65	0.01	0.53479854894134504	LUSC
ukb-a-238	MR Egger	19.40	0.02	0.53612224864456703	SCLC
eqtl-a-ENSG00000160588	IVW	4.32	0.12	0.53688351956592095	LUAD
ieu-b-142	MR Egger	45.35	0.00	0.53694480509944098	LUAD
ieu-b-25	MR Egger	45.35	0.00	0.53694480509944098	LUAD
ukb-b-7460	IVW	25.47	0.01	0.56816756032285898	SCLC
ukb-a-328	MR Egger	9.48	0.05	0.57786534188374605	SCLC
ukb-b-469	IVW	4.96	0.08	0.59669375614845299	Lung cancer
ukb-a-238	IVW	26.00	0.00	0.61538622250735997	LUSC
ukb-b-2732	MR Egger	2.64	0.10	0.62116274447675002	LUSC
ukb-a-343	IVW	5.42	0.07	0.63091293641992097	SCLC
ieu-b-142	IVW	60.88	0.00	0.63862705279095799	LUAD
ieu-b-25	IVW	60.88	0.00	0.63862705279095799	LUAD
ukb-b-10831	MR Egger	22.37	0.00	0.64234358055312102	LUSC
ebi-a-GCST009971	IVW	36.45	0.00	0.64332281269564096	Lung cancer
ukb-a-238	MR Egger	25.43	0.00	0.64611500521229104	LUSC
ukb-b-6019	IVW	19.80	0.01	0.64647638036559396	LUSC
ukb-b-7460	IVW	34.10	0.00	0.64807826298725302	LUSC
ebi-a-GCST009971	MR Egger	34.98	0.00	0.65695491568826903	Lung cancer
ukb-b-469	IVW	5.84	0.05	0.65769944698156602	LUSC
ukb-b-6019	IVW	17.83	0.01	0.66342974160715096	Lung cancer
ukb-b-7460	IVW	104.64	0.00	0.66550991897738598	LUAD
ukb-b-10831	IVW	23.95	0.00	0.66601231138510697	SCLC
ukb-b-7460	IVW	27.13	0.00	0.66831638513713199	Lung cancer
ukb-b-6019	MR Egger	15.11	0.01	0.66902997819855203	Lung cancer
eqtl-a-ENSG00000223534	IVW	18.35	0.01	0.67307149931030397	LUSC
ukb-b-6019	MR Egger	18.43	0.01	0.67447191459158096	SCLC
ebi-a-GCST90013972	IVW	18.58	0.00	0.67701386116425799	SCLC
ukb-b-7460	MR Egger	24.91	0.00	0.67882210964963297	Lung cancer
ebi-a-GCST90013922	IVW	18.77	0.00	0.68035281624628996	SCLC
ukb-a-328	IVW	15.68	0.01	0.68102504727408897	SCLC
ukb-a-328	MR Egger	12.62	0.01	0.68310319390005703	Lung cancer

ukb-b-6019	IVW	22.88	0.00	0.69410399046092497	SCLC
ukb-a-238	IVW	35.70	0.00	0.71986444504832703	SCLC
ukb-b-10831	IVW	39.55	0.00	0.72183886110317497	LUAD
eqtl-a-ENSG00000223534	MR Egger	18.24	0.00	0.72593511938543598	LUSC
ukb-a-328	IVW	18.51	0.00	0.72981436763194896	Lung cancer
ukb-b-10831	IVW	33.72	0.00	0.73310058311748805	LUSC
ukb-a-343	MR Egger	3.82	0.05	0.73788982971813	LUSC
ukb-a-237	MR Egger	30.90	0.00	0.741058431940114	LUSC
ukb-b-469	MR Egger	4.01	0.05	0.75069973905131004	Lung cancer
ukb-a-237	IVW	33.37	0.00	0.76024137107881096	SCLC
ukb-b-3599	MR Egger	4.18	0.04	0.76062809878420801	Lung cancer
ukb-b-3599	IVW	8.46	0.01	0.76362183705268105	Lung cancer
eqtl-a-ENSG00000160588	MR Egger	4.24	0.04	0.76392508264884895	LUAD
ukb-b-469	MR Egger	4.32	0.04	0.76847315915615999	LUSC
ukb-a-237	IVW	51.74	0.00	0.82606132379171204	LUSC
ieu-b-142	MR Egger	122.90	0.00	0.829129415429806	Lung cancer
ieu-b-25	MR Egger	122.90	0.00	0.829129415429806	Lung cancer
finn-b-J10_COPDNAS_INCLAVO	IVW	5.91	0.02	0.83088424144509399	SCLC
ieu-b-142	IVW	150.13	0.00	0.853463183749259	Lung cancer
ieu-b-25	IVW	150.13	0.00	0.853463183749259	Lung cancer
ukb-a-342	MR Egger	14.54	0.00	0.86243141755808805	LUSC
ukb-a-342	IVW	22.83	0.00	0.86859202847205397	LUAD
ebi-a-GCST90013922	IVW	54.69	0.00	0.87200066969297596	LUSC
ebi-a-GCST90013972	IVW	54.88	0.00	0.87244208102530496	LUSC
ukb-a-342	IVW	23.86	0.00	0.87426541315455297	LUSC
finn-b-J10_COPDNAS_INCLAVO	IVW	8.27	0.00	0.87910920621577904	LUAD
ebi-a-GCST90013922	MR Egger	50.98	0.00	0.88231455507473899	LUSC
ebi-a-GCST90013972	MR Egger	51.12	0.00	0.88262241703316002	LUSC
ukb-b-1572	IVW	18.42	0.00	0.89142658134877195	LUSC
ukb-a-342	MR Egger	18.62	0.00	0.89257785226658803	LUAD
ukb-b-14521	MR Egger	18.63	0.00	0.89262892985581999	LUSC
ukb-b-10831	MR Egger	48.90	0.00	0.89775996362420996	Lung cancer
ukb-b-2732	IVW	20.12	0.00	0.90058537851085696	LUSC
finn-b-COPD_HOSPITAL	IVW	10.35	0.00	0.90342824362699703	LUAD
ukb-b-10831	IVW	65.62	0.00	0.90856240214127904	Lung cancer
ukb-b-14521	MR Egger	22.11	0.00	0.90952861476549696	LUAD
ukb-b-14521	IVW	33.25	0.00	0.90976927711512301	LUSC
ukb-b-8133	IVW	11.94	0.00	0.91626043944457702	LUSC
ukb-b-14521	MR Egger	24.47	0.00	0.918277863410352	Lung cancer
ukb-a-343	IVW	24.57	0.00	0.91861556300705205	LUSC
ukb-b-1572	MR Egger	12.54	0.00	0.92024035035006901	LUSC
finn-b-J10_COPDNAS_INCLAVO	IVW	13.22	0.00	0.92434893425444697	LUSC
ukb-a-205	MR Egger	13.75	0.00	0.92726025382357002	LUAD
ebi-a-GCST90013972	MR Egger	85.00	0.00	0.929412426680435	Lung cancer
ebi-a-GCST90013922	MR Egger	87.76	0.00	0.93162952140701605	Lung cancer
ukb-b-14521	IVW	43.92	0.00	0.93169059684773203	LUAD

ebi-a-GCST90013972	IVW	110.48	0.00	0.93664072511884799	Lung cancer
ebi-a-GCST90013922	IVW	112.54	0.00	0.93779741292872698	Lung cancer
ukb-a-205	IVW	32.26	0.00	0.93799420973173098	LUAD
ukb-a-342	MR Egger	35.66	0.00	0.94391815899751796	Lung cancer
ukb-a-342	IVW	56.65	0.00	0.94704136859066801	Lung cancer
ukb-a-205	IVW	41.08	0.00	0.951309878230302	Lung cancer
ukb-b-14521	IVW	78.47	0.00	0.961768958682792	Lung cancer
ukb-a-205	MR Egger	30.23	0.00	0.96692078946172999	Lung cancer

supplementary Table 7 The pleiotropy test of between exposures and all subtypes of lung cancer

id.exposure	egger_intercept	se	pval	type
ieu-b-4879	-0.01	0.00	0.00	LUAD
ukb-b-7460	0.07	0.02	0.00	LUAD
ieu-b-4879	-0.01	0.00	0.00	Lung cancer
ukb-b-10831	0.11	0.02	0.00	LUAD
ieu-b-142	-0.03	0.01	0.01	LUSC
ieu-b-25	-0.03	0.01	0.01	LUSC
ukb-b-7460	-0.08	0.02	0.01	SCLC
ebi-a-GCST90029012	-0.02	0.01	0.01	LUAD
ukb-b-10831	-0.10	0.03	0.01	SCLC
ukb-a-278	0.01	0.00	0.01	Lung cancer
ukb-a-249	0.01	0.00	0.01	SCLC
ukb-b-17685	-0.11	0.04	0.01	LUSC
ukb-a-398	0.04	0.02	0.01	LUSC
ebi-a-GCST90018934	0.02	0.01	0.01	LUSC
ieu-b-142	-0.03	0.01	0.01	LUAD
ieu-b-25	-0.03	0.01	0.01	LUAD
ukb-a-237	-0.12	0.04	0.02	SCLC
ukb-b-16489	-0.01	0.00	0.02	Lung cancer
ebi-a-GCST011365	0.02	0.01	0.02	LUSC
ukb-b-16489	-0.01	0.00	0.02	LUAD
ukb-a-291	0.01	0.00	0.02	Lung cancer
ukb-a-238	-0.10	0.04	0.02	SCLC
ukb-b-8909	0.01	0.00	0.03	Lung cancer
ukb-b-20188	0.01	0.00	0.03	Lung cancer
ukb-a-290	0.02	0.01	0.03	SCLC
ukb-b-12854	0.01	0.00	0.03	Lung cancer
ukb-a-282	0.01	0.00	0.03	Lung cancer
ebi-a-GCST90013975	0.01	0.00	0.04	Lung cancer
eqtl-a-ENSG00000149573	0.02	0.01	0.04	LUAD
ukb-b-6019	-0.07	0.03	0.04	LUSC
ieu-b-142	-0.03	0.01	0.04	Lung cancer
ieu-b-25	-0.03	0.01	0.04	Lung cancer
ukb-b-19393	0.00	0.00	0.04	Lung cancer

ebi-a-GCST90029012	-0.01	0.00	0.05	Lung cancer
ukb-a-237	-0.08	0.03	0.05	LUSC
ebi-a-GCST90029013	-0.01	0.01	0.05	LUAD
ukb-b-7460	-0.04	0.02	0.05	LUSC
ukb-b-8338	0.00	0.00	0.05	Lung cancer
ukb-b-8909	0.01	0.01	0.05	SCLC
ukb-b-20531	-0.01	0.00	0.05	LUSC
eqtl-a-ENSG00000149573	0.01	0.01	0.05	Lung cancer
ukb-a-274	0.01	0.00	0.05	Lung cancer
eqtl-a-ENSG00000158406	-0.02	0.01	0.06	Lung cancer
ukb-a-397	-0.01	0.01	0.06	LUAD
ukb-b-6704	0.00	0.00	0.06	Lung cancer
ukb-b-17729	-0.03	0.02	0.07	SCLC
ukb-b-20044	0.00	0.00	0.07	Lung cancer
finn-b-CD2_BENIGN_LEIOMYOMA_UTERI_EXALLC	0.02	0.01	0.07	LUSC
eqtl-a-ENSG00000169045	0.02	0.01	0.07	Lung cancer
ukb-b-1668	0.03	0.01	0.07	LUSC
ebi-a-GCST90012794	0.03	0.02	0.08	Lung cancer
ukb-b-10831	-0.05	0.03	0.08	LUSC
ukb-d-20116_0	-0.01	0.01	0.08	Lung cancer
ukb-a-279	0.01	0.01	0.08	SCLC
ukb-b-13799	0.06	0.03	0.09	LUSC
ebi-a-GCST90029013	-0.01	0.00	0.09	Lung cancer
ebi-a-GCST90013972	-0.16	0.08	0.09	SCLC
ukb-a-277	0.00	0.00	0.09	Lung cancer
ebi-a-GCST90013922	-0.16	0.08	0.09	SCLC
eqtl-a-ENSG00000145476	-0.02	0.01	0.10	LUSC
ebi-a-GCST90000046	-0.02	0.01	0.10	LUSC
ebi-a-GCST90000047	-0.01	0.00	0.10	LUAD
ebi-a-GCST90000514	-0.04	0.03	0.10	SCLC
ukb-b-20044	0.01	0.00	0.11	LUSC
ukb-b-12854	0.01	0.01	0.11	SCLC
ukb-b-18377	-0.01	0.00	0.11	LUSC
ukb-a-287	0.00	0.00	0.11	Lung cancer

eqtl-a-ENSG00000197077	-0.02	0.01	0.12	Lung cancer
ukb-b-16698	0.00	0.00	0.12	Lung cancer
ukb-a-399	-0.03	0.02	0.12	LUSC
ebi-a-GCST90013870	0.00	0.00	0.12	LUSC
ukb-a-283	0.00	0.00	0.12	Lung cancer
eqtl-a-ENSG00000162736	-0.03	0.02	0.13	LUSC
ukb-b-6591	-0.01	0.00	0.13	LUAD
ukb-a-275	0.00	0.00	0.13	Lung cancer
ukb-a-265	0.00	0.00	0.13	Lung cancer
ukb-b-6019	-0.03	0.02	0.13	LUAD
ukb-a-281	0.00	0.00	0.14	Lung cancer
eqtl-a-ENSG00000141298	0.02	0.01	0.14	Lung cancer
ukb-b-8755	0.04	0.02	0.14	LUSC
eqtl-a-ENSG00000111725	-0.02	0.02	0.14	LUSC
ukb-b-1489	-0.02	0.01	0.14	LUSC
ukb-b-11842	0.00	0.00	0.15	LUSC
ukb-a-286	0.01	0.01	0.15	SCLC
ukb-b-18096	0.00	0.00	0.15	LUSC
ebi-a-GCST90019017	0.01	0.01	0.15	LUSC
ukb-b-15590	0.00	0.00	0.15	LUSC
ukb-a-282	0.01	0.01	0.15	SCLC
eqtl-a-ENSG00000205726	-0.02	0.01	0.16	LUSC
ukb-a-398	-0.04	0.03	0.16	SCLC
ukb-b-16446	0.00	0.00	0.16	Lung cancer
ebi-a-GCST90013974	0.00	0.00	0.16	LUSC
ukb-d-30710_raw	0.01	0.01	0.16	SCLC
ukb-a-249	0.00	0.00	0.17	Lung cancer
ieu-a-1239	0.01	0.01	0.17	SCLC
ukb-b-14521	-0.20	0.09	0.17	Lung cancer
ukb-a-397	-0.01	0.01	0.17	Lung cancer
ukb-b-12039	0.00	0.00	0.17	LUSC
ukb-b-19393	0.01	0.00	0.18	SCLC
eqtl-a-ENSG00000135698	-0.02	0.01	0.18	LUAD
ukb-a-328	-0.10	0.06	0.18	SCLC
ukb-a-238	-0.02	0.02	0.18	Lung cancer
eqtl-a-ENSG00000177706	-0.02	0.01	0.18	Lung cancer

ukb-a-287	0.01	0.01	0.19	SCLC
ukb-a-283	0.01	0.01	0.19	SCLC
finn-b-DM_SEVERAL_COMPLICATIONS	-0.09	0.06	0.19	SCLC
ieu-a-1001	-0.01	0.01	0.20	Lung cancer
eqtl-a-ENSG00000168411	-0.02	0.01	0.20	Lung cancer
ukb-a-249	0.00	0.00	0.20	LUSC
finn-b-CD2_BENIGN_LEIOMYOMA_UTERI	0.01	0.01	0.20	LUSC
ukb-a-286	0.00	0.00	0.20	Lung cancer
ieu-b-5117	0.01	0.00	0.21	Lung cancer
ukb-b-5174	-0.02	0.01	0.21	LUAD
ebi-a-GCST90018793	0.01	0.01	0.21	LUSC
ebi-a-GCST90012024	-0.02	0.01	0.21	Lung cancer
ebi-a-GCST90000048	-0.01	0.01	0.21	Lung cancer
eqtl-a-ENSG00000213694	0.03	0.03	0.22	SCLC
ukb-b-15590	0.00	0.00	0.22	Lung cancer
eqtl-a-ENSG00000177508	0.01	0.01	0.22	Lung cancer
ebi-a-GCST90000048	-0.03	0.03	0.22	SCLC
ukb-a-5	-0.01	0.01	0.22	Lung cancer
ukb-a-237	-0.02	0.02	0.22	Lung cancer
ukb-b-20188	0.01	0.01	0.22	SCLC
ukb-a-279	0.00	0.00	0.23	Lung cancer
ebi-a-GCST009971	-0.03	0.02	0.23	LUAD
ebi-a-GCST90013972	-0.11	0.08	0.23	Lung cancer
ukb-b-19953	0.00	0.00	0.23	LUSC
ukb-b-12854	0.00	0.00	0.23	LUSC
ukb-b-2732	0.33	0.13	0.24	LUSC
ebi-a-GCST90013922	-0.11	0.08	0.24	Lung cancer
ukb-a-328	-0.04	0.03	0.24	Lung cancer
ukb-b-7859	0.00	0.00	0.24	LUSC
ukb-b-16878	0.03	0.03	0.25	LUSC
ukb-b-10831	-0.05	0.04	0.25	Lung cancer
ukb-a-343	0.68	0.29	0.26	LUSC
finn-b-I9_CORATHER	-0.02	0.02	0.26	LUSC
ukb-a-287	0.00	0.00	0.26	LUSC

prot-a-1051	-0.10	0.06	0.26	LUAD
ukb-b-9093	0.00	0.00	0.26	Lung cancer
ukb-a-343	0.53	0.23	0.26	SCLC
ebi-a-GCST90014020	0.00	0.00	0.26	LUSC
ukb-b-13799	0.02	0.02	0.27	Lung cancer
ieu-a-1239	0.00	0.00	0.27	LUAD
ukb-b-6019	-0.06	0.05	0.27	SCLC
finn-b-CD2_BENIGN_EXALLC	0.02	0.02	0.27	LUSC
ukb-b-19925	0.00	0.00	0.28	Lung cancer
ukb-b-969	-0.02	0.02	0.28	Lung cancer
ebi-a-GCST90029012	-0.01	0.01	0.28	LUSC
ukb-b-11842	0.00	0.00	0.28	SCLC
ukb-a-291	0.00	0.00	0.28	LUSC
ukb-a-278	0.01	0.01	0.29	SCLC
ukb-b-11075	0.10	0.07	0.29	LUSC
eqtl-a-ENSG00000072163	-0.03	0.02	0.29	LUSC
ukb-d-20116_0	-0.01	0.01	0.29	LUSC
ieu-b-5118	0.00	0.00	0.29	LUSC
ukb-b-14521	-0.17	0.12	0.30	LUAD
eqtl-a-ENSG00000196812	-0.08	0.04	0.30	LUSC
ukb-b-19520	0.00	0.00	0.30	Lung cancer
ukb-a-328	-0.04	0.04	0.31	LUSC
ukb-a-328	-0.03	0.02	0.31	LUAD
ukb-b-5174	-0.01	0.01	0.31	Lung cancer
ukb-b-18408	0.01	0.01	0.32	LUSC
ukb-b-12405	-0.02	0.02	0.32	Lung cancer
ieu-a-835	0.01	0.01	0.32	SCLC
ukb-b-16407	0.00	0.00	0.33	LUSC
ebi-a-GCST90029025	0.00	0.00	0.33	Lung cancer
ukb-b-20044	0.00	0.00	0.33	SCLC
ebi-a-GCST90018890	-0.05	0.04	0.33	LUSC
ukb-b-14521	-0.16	0.13	0.34	LUSC
ukb-b-7212	0.00	0.00	0.34	LUSC
ukb-b-2732	0.21	0.12	0.34	SCLC
eqtl-a-ENSG00000137218	-0.07	0.06	0.34	SCLC
ukb-a-398	0.01	0.01	0.34	Lung cancer
ebi-a-GCST90025972	0.00	0.00	0.34	Lung cancer
ukb-b-19921	0.00	0.00	0.34	LUSC

ukb-b-8338	0.00	0.00	0.35	SCLC
ukb-a-201	0.02	0.02	0.35	LUSC
ebi-a-GCST90018947	0.00	0.00	0.35	LUSC
ieu-b-142	-0.02	0.02	0.35	SCLC
ieu-b-25	-0.02	0.02	0.35	SCLC
ieu-a-1126	0.00	0.00	0.35	LUAD
finn-b-KRA_PSY_DEMENTIA_EXMORE	-0.02	0.02	0.35	SCLC
ukb-a-176	0.01	0.01	0.36	LUSC
eqtl-a-ENSG00000188199	-0.02	0.02	0.37	LUAD
eqtl-a-ENSG00000145416	-0.03	0.04	0.37	SCLC
ukb-a-180	-0.04	0.03	0.37	Lung cancer
ukb-a-265	0.01	0.01	0.37	SCLC
finn-b-KRA_PSY_DEMENTIA	-0.02	0.02	0.37	SCLC
ukb-a-342	-0.16	0.14	0.37	LUSC
ukb-a-275	0.01	0.01	0.38	SCLC
ukb-b-6306	0.01	0.01	0.38	LUSC
finn-b-AD_LO	-0.01	0.01	0.38	Lung cancer
ukb-b-6019	-0.02	0.02	0.39	Lung cancer
ukb-a-382	0.00	0.00	0.39	Lung cancer
ebi-a-GCST90029014	0.01	0.01	0.39	LUAD
ukb-a-342	-0.15	0.14	0.39	Lung cancer
eqtl-a-ENSG00000174007	-0.01	0.01	0.39	Lung cancer
ukb-b-6704	0.00	0.00	0.39	SCLC
ukb-b-12039	0.00	0.00	0.39	SCLC
eqtl-a-ENSG00000106009	-0.02	0.02	0.40	Lung cancer
ebi-a-GCST90029014	0.01	0.01	0.40	SCLC
ukb-b-12018	-0.06	0.04	0.40	Lung cancer
ebi-a-GCST90025994	0.00	0.00	0.40	LUSC
finn-b-AD_LO	-0.01	0.02	0.41	SCLC
ukb-a-283	0.00	0.00	0.41	LUSC
ukb-b-7212	0.00	0.00	0.41	SCLC
ukb-b-2134	0.01	0.01	0.41	Lung cancer
ukb-a-28	-0.01	0.01	0.42	LUAD
eqtl-a-ENSG00000108384	-0.01	0.02	0.42	LUSC
ebi-a-GCST90029007	0.00	0.00	0.42	LUSC
eqtl-a-ENSG00000156414	-0.01	0.01	0.42	Lung cancer
ukb-a-397	-0.01	0.01	0.42	LUSC
ukb-b-20531	0.00	0.01	0.42	SCLC

ukb-b-19393	0.00	0.00	0.42	LUSC
ieu-b-5118	0.00	0.01	0.42	SCLC
ukb-b-7460	0.02	0.03	0.42	Lung cancer
ebi-a-GCST90018949	0.00	0.00	0.42	LUSC
ieu-a-1001	-0.02	0.02	0.43	SCLC
finn-b-D3_ANAEMIA_B12_DEF	-0.03	0.02	0.43	Lung cancer
ebi-a-GCST90000045	0.01	0.01	0.43	Lung cancer
ukb-b-9405	0.00	0.00	0.43	LUSC
ukb-a-397	-0.01	0.01	0.44	SCLC
eqtl-a-ENSG00000101574	0.04	0.04	0.44	Lung cancer
eqtl-a-ENSG00000063438	0.09	0.09	0.44	LUSC
ukb-b-17729	0.01	0.01	0.44	LUSC
ukb-a-291	0.01	0.01	0.44	SCLC
ukb-b-12039	0.00	0.00	0.44	Lung cancer
ieu-b-4877	0.01	0.01	0.45	LUAD
eqtl-a-ENSG00000177406	0.01	0.02	0.45	LUAD
finn-b-F5_DEMENTIA_INCLAVO	-0.01	0.01	0.45	LUSC
ukb-b-19379	0.00	0.00	0.45	LUSC
ukb-b-12405	-0.02	0.02	0.45	LUSC
ukb-b-9405	0.00	0.00	0.45	Lung cancer
ebi-a-GCST90000046	-0.01	0.01	0.45	Lung cancer
ukb-b-2303	0.00	0.00	0.45	LUSC
ukb-a-205	0.13	0.11	0.45	LUAD
ebi-a-GCST007432	0.00	0.01	0.45	LUSC
ukb-b-3855	-0.08	0.10	0.46	SCLC
ukb-a-399	-0.01	0.01	0.46	Lung cancer
ukb-a-399	0.02	0.03	0.46	SCLC
ukb-b-11615	0.01	0.01	0.46	LUAD
ukb-a-434	0.02	0.03	0.46	LUSC
finn-b-F5_DEMENTIA	-0.01	0.01	0.47	LUSC
eqtl-a-ENSG00000041357	0.03	0.03	0.47	LUAD
ukb-b-7212	0.00	0.00	0.47	Lung cancer
ebi-a-GCST009971	0.03	0.04	0.47	SCLC
ebi-a-GCST90018947	0.00	0.00	0.47	Lung cancer
ebi-a-GCST90018982	0.01	0.02	0.47	LUSC
ebi-a-GCST90018949	0.00	0.00	0.48	Lung cancer
eqtl-a-ENSG00000166037	0.02	0.03	0.48	Lung cancer

ebi-a-GCST90018992	0.05	0.05	0.48	LUSC
ukb-a-248	0.00	0.01	0.48	SCLC
eqtl-a-ENSG00000196812	-0.03	0.03	0.49	Lung cancer
eqtl-a-ENSG00000101695	-0.02	0.02	0.49	Lung cancer
ebi-a-GCST009971	-0.01	0.02	0.49	Lung cancer
eqtl-a-ENSG00000259015	-0.03	0.03	0.49	Lung cancer
ukb-a-248	0.00	0.00	0.49	LUSC
ukb-b-3599	-0.03	0.03	0.50	Lung cancer
ukb-b-12018	-0.11	0.11	0.50	SCLC
ukb-b-7408	0.01	0.01	0.50	Lung cancer
ukb-b-20188	0.00	0.00	0.50	LUSC
ebi-a-GCST90016675	0.03	0.03	0.50	Lung cancer
ebi-a-GCST90000514	0.01	0.01	0.51	LUSC
ebi-a-GCST90014021	0.00	0.00	0.51	Lung cancer
ukb-b-7408	0.02	0.02	0.51	SCLC
ukb-a-264	0.01	0.01	0.51	SCLC
eqtl-a-ENSG00000124508	0.03	0.04	0.52	SCLC
ieu-a-1001	-0.01	0.01	0.52	LUSC
ukb-b-17729	0.01	0.01	0.52	Lung cancer
ukb-a-264	0.00	0.00	0.52	Lung cancer
ukb-b-18377	0.00	0.01	0.52	SCLC
ukb-b-6704	0.00	0.00	0.52	LUSC
ebi-a-GCST90000048	-0.01	0.01	0.53	LUSC
ebi-a-GCST90013972	-0.12	0.19	0.53	LUSC
ebi-a-GCST90013922	-0.12	0.18	0.53	LUSC
ukb-a-274	0.01	0.01	0.54	SCLC
ebi-a-GCST90029014	0.00	0.01	0.54	Lung cancer
eqtl-a-ENSG00000184056	0.01	0.02	0.54	Lung cancer
ukb-b-20261	-0.01	0.01	0.54	Lung cancer
ukb-a-202	0.03	0.04	0.54	Lung cancer
ebi-a-GCST90013975	0.00	0.01	0.55	SCLC
ebi-a-GCST90029007	0.00	0.00	0.55	Lung cancer
ukb-b-17670	-0.02	0.03	0.55	Lung cancer

ubm-b-3277	-0.09	0.10	0.56	LUSC
eqtl-a-ENSG00000229515	-0.02	0.04	0.56	LUSC
ukb-b-9405	0.00	0.01	0.56	SCLC
ebi-a-GCST90060127	0.02	0.02	0.56	Lung cancer
ukb-a-382	0.00	0.01	0.56	SCLC
eqtl-a-ENSG00000171055	-0.01	0.01	0.56	Lung cancer
ebi-a-GCST90014020	0.00	0.00	0.56	Lung cancer
ebi-a-GCST006250	0.00	0.01	0.57	Lung cancer
eqtl-a-ENSG00000260228	0.02	0.03	0.57	LUSC
ukb-a-342	-0.09	0.13	0.57	LUAD
eqtl-a-ENSG00000168411	-0.02	0.03	0.57	SCLC
eqtl-a-ENSG00000159873	-0.03	0.04	0.57	LUSC
eqtl-a-ENSG00000174749	0.01	0.02	0.58	Lung cancer
ebi-a-GCST006250	0.01	0.01	0.58	SCLC
ebi-a-GCST90096909	-0.01	0.01	0.58	Lung cancer
eqtl-a-ENSG00000175164	0.01	0.02	0.59	LUSC
ukb-b-11268	0.00	0.01	0.59	Lung cancer
ieu-b-40	0.00	0.00	0.59	Lung cancer
ukb-a-265	0.00	0.00	0.59	LUSC
eqtl-a-ENSG00000103160	-0.01	0.01	0.59	Lung cancer
ukb-a-382	0.00	0.01	0.59	LUSC
finn-b-DM_PERIPHATHERO	-0.04	0.06	0.59	LUSC
prot-a-2470	0.01	0.02	0.60	Lung cancer
finn-b-I9_REVASC	0.00	0.01	0.60	Lung cancer
eqtl-a-ENSG00000095261	0.05	0.08	0.60	Lung cancer
ieu-a-1239	0.00	0.00	0.61	LUSC
ukb-a-202	0.03	0.05	0.61	LUSC
eqtl-a-ENSG00000107890	-0.02	0.03	0.61	LUSC
ebi-a-GCST90014021	0.00	0.01	0.61	SCLC
ukb-b-1572	-0.10	0.14	0.62	LUSC
ukb-b-5192	0.00	0.01	0.62	LUSC
eqtl-a-ENSG00000139531	-0.01	0.03	0.63	Lung cancer
ebi-a-GCST90000045	-0.01	0.02	0.63	SCLC
ieu-b-40	0.00	0.00	0.63	SCLC
ubm-b-289	0.01	0.02	0.63	Lung cancer

ebi-a-GCST004604	0.00	0.00	0.63	Lung cancer
ieu-b-5117	0.00	0.01	0.64	LUSC
ebi-a-GCST90104006	0.00	0.00	0.65	LUSC
ukb-a-205	0.07	0.12	0.66	Lung cancer
ieu-b-40	0.00	0.00	0.66	LUSC
ukb-b-469	-0.06	0.10	0.66	LUSC
ukb-b-16407	0.00	0.00	0.66	Lung cancer
eqtl-a-ENSG00000145416	-0.01	0.01	0.66	Lung cancer
ukb-a-238	-0.02	0.04	0.66	LUSC
ebi-a-GCST90029012	0.00	0.01	0.67	SCLC
ebi-a-GCST006696	-0.03	0.06	0.67	LUSC
ukb-a-282	0.00	0.01	0.67	LUSC
ebi-a-GCST90000045	0.01	0.01	0.68	LUSC
ukb-d-D22	0.01	0.03	0.68	LUAD
ukb-b-8909	0.00	0.00	0.68	LUSC
ieu-b-24	0.02	0.04	0.69	LUSC
ukb-a-132	-0.01	0.03	0.69	Lung cancer
ebi-a-GCST90016675	0.02	0.05	0.69	LUSC
ebi-a-GCST009971	0.01	0.03	0.69	LUSC
eqtl-a-ENSG00000167483	0.01	0.02	0.69	Lung cancer
ukb-a-275	0.00	0.00	0.70	LUSC
finn-b-RHEU_ARTHRITIS_OTH	-0.02	0.04	0.70	Lung cancer
finn-b-G6_AD_WIDE	-0.01	0.02	0.70	SCLC
ukb-a-273	0.00	0.00	0.70	Lung cancer
ebi-a-GCST90000514	0.00	0.01	0.71	Lung cancer
ebi-a-GCST90029007	0.00	0.00	0.71	SCLC
ukb-b-2303	0.00	0.00	0.71	Lung cancer
eqtl-a-ENSG00000106305	-0.01	0.03	0.71	LUSC
ukb-b-469	-0.03	0.06	0.71	Lung cancer
ubm-b-1571	-0.03	0.07	0.71	Lung cancer
finn-b-I9_CABG_EXNONE	0.01	0.02	0.72	LUSC
eqtl-a-ENSG00000260276	0.01	0.02	0.72	LUSC
prot-a-1051	-0.02	0.04	0.72	Lung cancer
eqtl-a-ENSG00000119403	-0.01	0.02	0.72	Lung cancer

ebi-a-GCST90000047	0.00	0.00	0.72	Lung cancer
finn-b-I9_CORATHER_EXNONE	-0.01	0.02	0.73	LUSC
ebi-a-GCST006368	0.00	0.01	0.73	SCLC
eqtl-a-ENSG00000111906	0.00	0.01	0.73	Lung cancer
ukb-b-8338	0.00	0.00	0.73	LUSC
eqtl-a-ENSG00000134758	-0.01	0.02	0.73	LUSC
eqtl-a-ENSG00000156414	0.00	0.01	0.74	LUAD
ukb-b-19953	0.00	0.00	0.74	SCLC
eqtl-a-ENSG00000066084	0.01	0.02	0.74	LUAD
finn-b-I9_REVASC	0.00	0.01	0.75	LUSC
eqtl-a-ENSG00000259015	-0.02	0.05	0.75	LUAD
ukb-b-19953	0.00	0.00	0.75	Lung cancer
eqtl-a-ENSG00000142233	-0.01	0.02	0.75	LUAD
prot-a-3203	0.01	0.02	0.76	LUSC
ukb-a-237	0.01	0.03	0.76	LUAD
ukb-b-15169	0.01	0.02	0.77	Lung cancer
ukb-b-6134	0.00	0.02	0.78	LUSC
ukb-a-278	0.00	0.01	0.78	LUSC
ebi-a-GCST90025994	0.00	0.00	0.78	Lung cancer
ebi-a-GCST90018890	-0.01	0.03	0.79	Lung cancer
ukb-b-19921	0.00	0.00	0.79	Lung cancer
prot-a-710	0.01	0.03	0.79	LUSC
ebi-a-GCST90029013	0.00	0.01	0.80	LUSC
ukb-b-18377	0.00	0.00	0.80	Lung cancer
ukb-a-279	0.00	0.00	0.80	LUSC
ukb-b-6134	0.00	0.01	0.80	Lung cancer
eqtl-a-ENSG00000227598	0.00	0.02	0.80	Lung cancer
ukb-a-205	0.02	0.05	0.80	LUSC
ukb-b-20531	0.00	0.00	0.80	Lung cancer
eqtl-a-ENSG00000065911	0.01	0.05	0.80	SCLC
ukb-b-16878	0.00	0.02	0.81	Lung cancer
ukb-b-469	0.01	0.05	0.81	LUAD
ukb-b-5192	0.00	0.01	0.81	Lung cancer
ukb-a-434	0.00	0.02	0.81	Lung cancer
eqtl-a-ENSG00000204789	-0.01	0.02	0.82	LUAD

eqtl-a-ENSG00000041357	0.01	0.04	0.82	LUSC
ubm-b-847	-0.02	0.09	0.83	Lung cancer
ukb-a-274	0.00	0.01	0.83	LUSC
eqtl-a-ENSG00000026297	0.00	0.01	0.83	Lung cancer
eqtl-a-ENSG00000175164	0.00	0.01	0.83	Lung cancer
ebi-a-GCST90029013	0.00	0.01	0.83	SCLC
ukb-b-18096	0.00	0.00	0.83	Lung cancer
ukb-b-11615	0.00	0.01	0.84	Lung cancer
ebi-a-GCST90000050	0.00	0.01	0.84	LUSC
eqtl-a-ENSG00000140650	0.00	0.02	0.84	LUSC
finn-b-I9_ANGINA	0.00	0.02	0.85	LUSC
ukb-b-2303	0.00	0.00	0.85	SCLC
eqtl-a-ENSG00000074657	0.01	0.05	0.85	LUSC
ukb-b-11615	0.00	0.02	0.86	SCLC
eqtl-a-ENSG00000010818	0.01	0.04	0.86	Lung cancer
eqtl-a-ENSG00000107890	0.00	0.01	0.86	Lung cancer
ukb-b-4667	0.00	0.03	0.87	Lung cancer
eqtl-a-ENSG00000223534	-0.01	0.03	0.87	LUSC
ukb-b-16489	0.00	0.01	0.87	SCLC
ebi-a-GCST006250	0.00	0.01	0.87	LUSC
ebi-a-GCST90000050	0.00	0.01	0.87	Lung cancer
eqtl-a-ENSG00000239415	0.00	0.01	0.88	Lung cancer
ebi-a-GCST90018990	0.00	0.01	0.88	LUSC
eqtl-a-ENSG00000165905	0.01	0.05	0.88	Lung cancer
ukb-a-201	0.00	0.01	0.88	Lung cancer
ebi-a-GCST90012877	0.00	0.01	0.88	LUSC
eqtl-a-ENSG00000100462	0.00	0.02	0.89	LUAD
eqtl-a-ENSG00000111906	0.00	0.01	0.89	LUSC
ieu-b-4877	0.00	0.01	0.89	LUSC
ukb-b-18096	0.00	0.00	0.90	SCLC
ebi-a-GCST90018949	0.00	0.00	0.90	SCLC
ieu-b-4877	0.00	0.01	0.90	Lung cancer
ukb-b-19379	0.00	0.00	0.90	Lung cancer
ebi-a-GCST90000047	0.00	0.01	0.90	SCLC
eqtl-a-ENSG00000205978	0.00	0.02	0.90	LUSC

ebi-a-GCST90013974	0.00	0.00	0.90	Lung cancer
finn-b-I9_CABG	0.00	0.02	0.90	LUSC
ukb-b-6306	0.00	0.01	0.90	Lung cancer
eqtl-a-ENSG00000100450	0.00	0.03	0.91	LUSC
eqtl-a-ENSG00000082641	0.04	0.25	0.91	SCLC
ebi-a-GCST90029014	0.00	0.01	0.91	LUSC
ukb-b-7408	0.00	0.01	0.91	LUSC
eqtl-a-ENSG00000160588	0.02	0.16	0.91	LUAD
finn-b-DM_PERIPHATHERO	0.00	0.04	0.91	Lung cancer
ukb-b-11615	0.00	0.01	0.91	LUSC
ebi-a-GCST90013974	0.00	0.00	0.92	SCLC
eqtl-a-ENSG00000100462	0.00	0.02	0.92	Lung cancer
ukb-d-I9_IHD	0.00	0.02	0.92	LUSC
ukb-b-6591	0.00	0.01	0.93	SCLC
ebi-a-GCST90013870	0.00	0.00	0.93	SCLC
ebi-a-GCST90013975	0.00	0.00	0.93	LUSC
ukb-a-132	0.00	0.05	0.93	LUSC
ukb-a-248	0.00	0.00	0.93	Lung cancer
ukb-b-12841	0.01	0.07	0.94	LUSC
ukb-a-238	0.00	0.02	0.94	LUAD
eqtl-a-ENSG00000186470	0.00	0.03	0.94	SCLC
ebi-a-GCST90025994	0.00	0.00	0.94	SCLC
ukb-a-142	0.00	0.01	0.95	Lung cancer
ebi-a-GCST90018947	0.00	0.00	0.95	SCLC
ukb-a-264	0.00	0.01	0.95	LUSC
ebi-a-GCST90014020	0.00	0.01	0.95	SCLC
ukb-a-505	0.00	0.01	0.95	Lung cancer
ebi-a-GCST004441	0.00	0.07	0.96	LUAD
eqtl-a-ENSG00000211677	0.00	0.02	0.96	LUAD
ebi-a-GCST90013870	0.00	0.00	0.96	Lung cancer
ukb-d-I9_CORATHER	0.00	0.01	0.96	LUSC
eqtl-a-ENSG00000060709	0.00	0.02	0.96	LUSC
ukb-a-272	0.00	0.00	0.96	Lung cancer
ebi-a-GCST90000047	0.00	0.01	0.97	LUSC
ukb-b-15169	0.00	0.03	0.97	LUSC
ukb-b-16489	0.00	0.01	0.97	LUSC
ieu-a-1239	0.00	0.00	0.97	Lung cancer
ukb-b-6591	0.00	0.01	0.98	LUSC

ieu-b-5118	0.00	0.00	0.99	Lung cancer
ebi-a-GCST90019476	0.00	0.02	0.99	LUSC
ukb-b-6591	0.00	0.01	0.99	Lung cancer
ebi-a-GCST90018982	0.00	0.01	0.99	Lung cancer
bbj-a-73	0.00	0.00	1.00	Lung cancer

supplementary Table 8 The MR results of between exposures and LUAD using MRAID method

name	beta	SE	p	sigmabeta	sigmaeta	sigma_error1	sigma_error2	type
ieu-a-1239	-1.86	0.00	0.00E+00	1.24E-03	6.57E-03	0.96	0.79	LUAD
ukb-b-10831	2.55	0.00	0.00E+00	5.03E-02	1.17E-01	1.00	0.98	LUAD
finn-b-J10_COPDNAS	0.26	0.03	2.22E-16	6.60E-01	6.79E-01	0.76	0.98	LUAD
ieu-b-4879	0.90	0.11	2.22E-16	2.70E-03	5.02E-03	0.96	0.95	LUAD
ieu-b-142	1.28	0.17	2.09E-13	1.56E-02	3.63E-02	0.99	0.97	LUAD
ukb-a-328	1.64	0.23	2.63E-12	6.69E-02	1.63E-01	0.99	0.97	LUAD
ebi-a-GCST90000047	-0.58	0.08	5.09E-12	2.25E-03	4.41E-03	0.97	0.98	LUAD
ukb-b-6591	-0.35	0.05	6.25E-11	2.21E-03	4.72E-03	0.96	0.99	LUAD
ieu-b-25	1.49	0.23	2.44E-10	1.56E-02	3.57E-02	0.99	0.97	LUAD
ukb-b-469	0.86	0.14	1.66E-09	1.40E-01	3.03E-01	0.99	1.00	LUAD
ukb-a-397	-0.35	0.06	6.97E-09	2.32E-03	5.06E-03	0.96	0.99	LUAD
ebi-a-GCST90029013	-0.31	0.06	1.80E-08	2.05E-03	4.24E-03	0.96	0.99	LUAD
ieu-b-4877	0.61	0.11	4.77E-08	4.46E-03	1.24E-02	0.99	1.00	LUAD
eqtl-a-ENSG00000160588	-0.30	0.06	1.17E-07	1.35E-01	3.00E-01	0.96	1.00	LUAD
ebi-a-GCST90029014	0.46	0.09	1.44E-07	3.09E-03	7.96E-03	0.99	1.00	LUAD
ebi-a-GCST009970	0.89	0.17	3.75E-07	3.87E-01	5.98E-01	0.98	0.98	LUAD
eqtl-a-ENSG00000138593	-0.75	0.16	2.41E-06	2.15E-01	5.09E-01	1.00	1.00	LUAD
ukb-d-D22	-1.60	0.34	2.68E-06	5.98E-02	1.51E-01	1.00	1.00	LUAD
ukb-a-237	0.78	0.17	3.27E-06	4.61E-02	8.49E-02	0.99	0.98	LUAD
ebi-a-GCST90029012	-0.32	0.07	3.51E-06	1.97E-03	4.01E-03	0.96	0.98	LUAD
ukb-b-8133	-3.99	0.87	5.06E-06	3.32E-01	7.98E-01	1.00	0.98	LUAD
eqtl-a-ENSG00000026036	-1.00	0.23	1.39E-05	2.11E-01	3.81E-01	1.00	1.00	LUAD
ukb-d-COPD_EARLYANDLATER	12.43	2.96	2.67E-05	4.53E-01	6.50E-01	1.00	0.99	LUAD
ukb-b-6019	1.45	0.35	2.71E-05	4.54E-02	9.01E-02	0.99	0.98	LUAD
ukb-b-13532	-8.76	2.13	3.80E-05	3.63E-01	6.23E-01	1.00	1.00	LUAD
prot-a-1051	-0.10	0.02	4.21E-05	2.18E-01	1.99E-01	0.67	1.00	LUAD
eqtl-a-ENSG00000259015	0.10	0.03	6.02E-05	1.30E-01	2.36E-01	0.87	1.00	LUAD
eqtl-a-ENSG00000188266	-3.62	0.92	8.46E-05	3.75E-01	6.79E-01	1.00	0.98	LUAD
ukb-a-238	0.54	0.14	1.40E-04	3.13E-02	9.24E-02	0.99	0.98	LUAD
ukb-b-11615	-0.42	0.12	3.23E-04	4.63E-03	1.30E-02	0.99	1.00	LUAD
ukb-d-K11_OTHDIG	1.40	0.39	3.52E-04	1.98E-01	4.11E-01	1.00	1.00	LUAD
eqtl-a-ENSG00000066084	-0.17	0.05	3.70E-04	9.45E-02	1.87E-01	0.95	1.00	LUAD
eqtl-a-ENSG00000156414	-0.14	0.04	4.89E-04	2.53E-02	6.01E-02	0.94	1.00	LUAD
eqtl-a-ENSG00000041357	1.22	0.35	5.19E-04	1.40E-01	2.97E-01	0.98	0.98	LUAD
ukb-b-5174	0.70	0.21	6.18E-04	2.13E-02	5.42E-02	1.00	1.00	LUAD
prot-c-5102_55_3	-0.05	0.02	6.79E-04	3.60E-01	5.24E-01	0.59	1.00	LUAD
eqtl-a-ENSG00000142233	0.24	0.07	8.95E-04	1.06E-01	2.46E-01	0.97	1.00	LUAD
eqtl-a-ENSG00000211677	0.21	0.06	1.01E-03	1.12E-01	2.22E-01	0.97	1.00	LUAD
ieu-a-1126	0.19	0.06	1.95E-03	3.13E-03	6.35E-03	0.93	0.99	LUAD
ukb-a-28	0.80	0.26	1.97E-03	3.13E-02	8.80E-02	1.00	1.00	LUAD
ukb-a-40	-15.33	5.08	2.54E-03	3.48E-01	5.01E-01	1.00	0.98	LUAD
ukb-b-7460	1.95	0.65	2.62E-03	3.14E-02	8.30E-02	0.99	0.98	LUAD
ukb-a-342	1.02	0.34	2.68E-03	1.04E-01	2.71E-01	0.99	0.98	LUAD

ukb-b-16489	-0.40	0.14	2.83E-03	1.70E-03	2.12E-03	0.94	0.93	LUAD
eqtl-a-ENSG00000177406	0.12	0.04	3.35E-03	6.65E-02	1.31E-01	0.94	1.00	LUAD
ukb-b-16751	5.14	1.89	6.54E-03	4.02E-01	8.25E-01	1.00	1.00	LUAD
ebi-a-GCST90006898	-0.86	0.32	6.71E-03	4.36E-01	1.21E+00	1.00	1.00	LUAD
finn-b-COPD_HOSPITAL	0.19	0.07	1.17E-02	3.16E-01	3.95E-01	0.71	0.98	LUAD
eqtl-a-ENSG00000100462	-0.17	0.07	1.25E-02	6.28E-02	1.18E-01	0.98	1.00	LUAD
ukb-b-16019	8.84	3.57	1.32E-02	3.65E-01	8.47E-01	1.00	0.99	LUAD
ebi-a-GCST90006885	0.69	0.29	1.66E-02	3.89E-01	6.72E-01	1.00	1.00	LUAD
ukb-b-4801	7.90	3.32	1.75E-02	3.42E-01	1.03E+00	1.00	0.98	LUAD
eqtl-a-ENSG00000204789	0.20	0.08	1.98E-02	7.10E-02	1.67E-01	0.97	1.00	LUAD
finn-b-J10_COPDNAS_INCLAVO	0.18	0.08	2.05E-02	3.54E-01	3.64E-01	0.70	0.98	LUAD
ebi-a-GCST90018902	18.35	8.70	3.49E-02	3.50E-01	4.84E-01	1.00	0.98	LUAD
ukb-b-13952	10.47	5.43	5.38E-02	3.87E-01	8.28E-01	1.00	0.98	LUAD
eqtl-a-ENSG00000149573	-0.06	0.04	7.56E-02	1.66E-02	3.27E-02	0.91	1.00	LUAD
ebi-a-GCST90013883	-4.41	2.85	1.22E-01	3.60E-01	9.12E-01	1.00	1.00	LUAD
ebi-a-GCST90101889	0.38	0.25	1.35E-01	3.62E-01	5.21E-01	0.97	0.99	LUAD
eqtl-a-ENSG00000104047	-1.42	0.95	1.38E-01	3.81E-01	6.23E-01	1.00	1.00	LUAD
prot-a-1256	-0.44	0.31	1.59E-01	3.84E-01	7.35E-01	0.99	1.00	LUAD
ebi-a-GCST009971	0.08	0.05	1.61E-01	4.02E-02	6.67E-02	0.80	1.00	LUAD
ukb-b-14521	3.59	2.60	1.67E-01	9.95E-02	2.20E-01	1.00	0.98	LUAD
ukb-a-205	6.60	4.85	1.74E-01	1.37E-01	2.17E-01	1.00	0.98	LUAD
eqtl-a-ENSG00000135698	0.06	0.05	1.93E-01	3.03E-02	6.35E-02	0.95	1.00	LUAD
prot-a-1255	-0.77	0.62	2.16E-01	3.77E-01	1.22E+00	0.99	1.00	LUAD
eqtl-a-ENSG00000130755	0.87	0.70	2.18E-01	3.42E-01	1.11E+00	0.98	1.00	LUAD
eqtl-a-ENSG00000118680	-0.42	0.35	2.28E-01	4.03E-01	8.65E-01	0.99	1.00	LUAD
ebi-a-GCST90016676	-0.99	0.85	2.43E-01	3.09E-01	8.35E-01	1.00	1.00	LUAD
eqtl-a-ENSG00000071054	2.80	2.40	2.44E-01	3.67E-01	6.04E-01	1.00	1.00	LUAD
ukb-b-7647	6.66	6.16	2.80E-01	3.40E-01	1.47E+00	1.00	0.99	LUAD
ebi-a-GCST009968	1.02	0.95	2.84E-01	4.34E-01	7.84E-01	0.99	0.98	LUAD
eqtl-a-ENSG00000145414	-0.62	0.62	3.18E-01	3.32E-01	8.14E-01	1.00	1.00	LUAD
ukb-b-1997	-8.68	8.72	3.19E-01	3.69E-01	4.46E-01	1.00	1.00	LUAD
ebi-a-GCST009965	0.65	0.67	3.32E-01	3.90E-01	4.30E-01	0.98	0.98	LUAD
eqtl-a-ENSG00000099821	-0.79	0.93	3.97E-01	3.77E-01	6.93E-01	0.99	1.00	LUAD
eqtl-a-ENSG00000188199	-0.03	0.04	4.44E-01	1.03E-01	1.71E-01	0.84	1.00	LUAD
ukb-b-11231	6.16	8.85	4.87E-01	4.02E-01	4.92E-01	1.00	1.00	LUAD
eqtl-a-ENSG00000074527	0.67	1.11	5.42E-01	4.19E-01	7.42E-01	1.00	1.00	LUAD
ebi-a-GCST90013933	-2.95	4.91	5.47E-01	4.39E-01	6.50E-01	1.00	1.00	LUAD
ukb-a-298	3.78	6.66	5.70E-01	3.94E-01	8.12E-01	1.00	0.99	LUAD
prot-a-1091	-0.35	0.64	5.80E-01	3.73E-01	6.25E-01	0.98	1.00	LUAD
eqtl-a-ENSG00000156958	-0.15	0.30	6.23E-01	2.24E-01	4.27E-01	0.98	1.00	LUAD
ebi-a-GCST009966	-0.23	0.51	6.57E-01	3.56E-01	7.04E-01	0.99	1.00	LUAD
prot-a-1621	-0.02	0.07	7.72E-01	5.12E-01	3.86E-01	0.91	1.00	LUAD
eqtl-a-ENSG00000137265	0.19	0.79	8.16E-01	4.16E-01	6.24E-01	1.00	1.00	LUAD
ebi-a-GCST004441	0.00	0.02	8.50E-01	1.18E-01	1.99E-01	0.93	1.00	LUAD
finn-b-C3_RESPIRATORY_INTRATHORACIC	0.01	0.05	8.89E-01	4.11E-01	2.84E-01	0.91	0.98	LUAD

finn-b-C3_RESPIRATORY_INTRATHORACIC_EXALLC	-0.01	0.05	9.19E-01	5.51E-01	2.89E-01	0.91	0.98	LUAD
finn-b-COPD_LATER	0.00	0.02	9.87E-01	4.21E-01	2.62E-01	0.94	0.98	LUAD

supplementary Table 9 The MR results of between exposures and SCLC using MRAID method

name	beta	SE	p	sigmabeta	sigmaeta	sigma_error1	sigma_error2	type
ebi-a-GCST006250	-0.54	0.04	1.00E-50	1.29E-03	9.53E-03	0.96	0.42	SCLC
ebi-a-GCST006368	0.84	0.06	1.00E-50	1.65E-02	4.70E-02	0.99	0.95	SCLC
ebi-a-GCST009968	1.84	0.22	2.22E-16	2.00E-03	4.35E-03	0.96	0.97	SCLC
ebi-a-GCST009970	1.22	0.15	1.11E-15	1.67E-02	4.67E-02	0.99	0.95	SCLC
ebi-a-GCST009971	0.19	0.02	2.66E-15	1.95E-03	2.74E-03	0.91	0.76	SCLC
ebi-a-GCST90000045	-0.80	0.11	7.66E-14	1.84E-03	1.01E-02	0.90	0.43	SCLC
ebi-a-GCST90000047	-0.73	0.10	9.15E-14	2.65E-03	5.79E-03	0.96	0.97	SCLC
ebi-a-GCST90000048	-1.31	0.18	6.84E-13	2.12E-03	3.19E-03	0.94	0.82	SCLC
ebi-a-GCST90000514	1.57	0.23	1.45E-11	2.15E-03	4.54E-03	0.97	0.95	SCLC
ebi-a-GCST90013870	0.21	0.03	3.28E-11	2.06E-03	4.24E-03	0.96	0.97	SCLC
ebi-a-GCST90013922	6.70	1.06	2.32E-10	1.94E-03	4.18E-03	0.92	0.64	SCLC
ebi-a-GCST90013972	6.88	1.19	7.14E-09	2.39E-03	5.33E-03	0.97	0.98	SCLC
ebi-a-GCST90013974	0.12	0.02	1.29E-08	5.75E-02	1.36E-01	1.00	0.95	SCLC
ebi-a-GCST90013975	0.06	0.01	1.61E-08	1.99E-03	2.93E-03	0.93	0.79	SCLC
ebi-a-GCST90014020	1.42	0.25	2.37E-08	2.70E-03	5.72E-03	0.96	0.99	SCLC
ebi-a-GCST90014021	0.08	0.02	5.72E-08	1.75E-03	3.18E-03	0.95	0.92	SCLC
ebi-a-GCST90018902	23.19	4.27	5.82E-08	1.89E-03	4.52E-03	0.91	0.62	SCLC
ebi-a-GCST90018947	0.07	0.01	1.05E-07	3.24E-01	6.34E-01	1.00	0.97	SCLC
ebi-a-GCST90018949	0.03	0.01	1.30E-07	2.17E-03	4.82E-03	0.97	0.97	SCLC
ebi-a-GCST90025994	0.04	0.01	2.31E-07	1.33E-01	3.36E-01	1.00	0.97	SCLC
ebi-a-GCST90029007	0.01	0.00	2.82E-07	5.79E-03	1.54E-02	0.99	0.99	SCLC
ebi-a-GCST90029012	-0.79	0.16	9.61E-07	2.03E-03	3.22E-03	0.93	0.77	SCLC
ebi-a-GCST90029013	-0.90	0.19	2.09E-06	5.65E-02	1.31E-01	1.00	0.95	SCLC
ebi-a-GCST90029014	0.82	0.18	4.86E-06	4.33E-03	1.14E-02	0.99	0.99	SCLC

ebi-a-GCST90093341	-1.37	0.30	5.43E-06	7.56E-01	7.41E-01	0.99	0.97	SCLC
eqtl-a-ENSG00000041357	0.09	0.02	6.66E-06	6.25E-03	1.74E-02	0.99	0.99	SCLC
eqtl-a-ENSG00000065911	0.53	0.12	1.03E-05	3.14E-03	7.67E-03	0.99	0.99	SCLC
eqtl-a-ENSG00000082641	0.42	0.10	1.05E-05	5.38E-03	1.45E-02	1.00	0.99	SCLC
eqtl-a-ENSG00000096654	2.45	0.57	1.66E-05	2.08E-03	3.13E-03	0.93	0.82	SCLC
eqtl-a-ENSG00000100281	1.84	0.43	1.78E-05	7.55E-03	1.99E-02	0.99	0.99	SCLC
eqtl-a-ENSG00000124508	0.38	0.09	1.93E-05	4.09E-01	9.68E-01	0.90	0.97	SCLC
eqtl-a-ENSG00000124549	1.52	0.36	2.09E-05	8.03E-03	2.28E-02	1.00	0.99	SCLC
eqtl-a-ENSG00000137218	0.59	0.14	2.21E-05	1.39E-01	3.14E-01	0.99	0.97	SCLC
eqtl-a-ENSG00000145416	0.14	0.03	2.42E-05	1.93E-03	3.80E-03	0.93	0.68	SCLC
eqtl-a-ENSG00000162623	1.97	0.47	2.88E-05	2.07E-03	2.97E-03	0.93	0.81	SCLC
eqtl-a-ENSG00000168411	-0.29	0.07	3.98E-05	1.62E-03	1.04E-02	0.92	0.44	SCLC
eqtl-a-ENSG00000186354	-1.78	0.44	5.12E-05	1.50E-01	1.28E-01	0.18	1.00	SCLC
eqtl-a-ENSG00000186470	-0.13	0.03	5.41E-05	5.26E-03	1.33E-02	0.99	0.99	SCLC
eqtl-a-ENSG00000188266	-4.08	1.01	5.44E-05	1.91E-03	3.33E-03	0.91	0.70	SCLC
eqtl-a-ENSG00000198563	-0.34	0.09	1.30E-04	7.52E-02	1.61E-01	0.95	1.00	SCLC
eqtl-a-ENSG00000204592	0.29	0.08	1.54E-04	3.66E-01	1.66E-02	15.24	0.99	SCLC
eqtl-a-ENSG00000211785	0.57	0.15	2.53E-04	6.63E-02	1.72E-01	1.00	1.00	SCLC
eqtl-a-ENSG00000213694	-0.33	0.09	2.88E-04	4.81E-03	1.31E-02	0.99	1.00	SCLC
finn-b-AD_LO	-0.04	0.01	3.96E-04	1.53E-01	3.36E-01	0.97	1.00	SCLC
finn-b-C3_RESPIRATORY_INTRATHORACIC	0.59	0.17	5.03E-04	2.50E-01	7.49E-02	2.11	1.00	SCLC
finn-b-C3_RESPIRATORY_INTRATHORACIC_EXALLC	1.02	0.30	6.32E-04	3.01E-01	6.52E-01	1.00	0.98	SCLC
finn-b-COPD_LATER	0.62	0.18	6.40E-04	1.32E-01	3.38E-01	1.00	1.00	SCLC
finn-b-DM_SEVERAL_COMPLICATIONS	-0.09	0.03	7.51E-04	3.10E-01	9.31E-02	2.42	1.00	SCLC
finn-b-G6_AD_WIDE	-0.03	0.01	7.86E-04	5.19E-03	1.42E-02	0.99	0.99	SCLC

finn-b-J10_COPDNAS	0.00	0.00	8.04E-04	2.91E-01	8.87E-02	2.50	1.00	SCLC
finn-b-J10_COPDNAS_INCLAVO	0.00	0.00	9.68E-04	6.49E-03	1.75E-02	0.99	0.99	SCLC
finn-b-KRA_PSY_DEMENTIA	-0.04	0.01	1.68E-03	2.84E-02	7.73E-02	0.99	0.96	SCLC
finn-b-KRA_PSY_DEMENTIA_EXMORE	-0.04	0.01	2.30E-03	5.65E-02	1.25E-01	0.95	1.00	SCLC
ieu-a-1001	-1.12	0.38	2.92E-03	3.17E-01	5.15E-02	3.98	1.00	SCLC
ieu-a-1239	-3.48	1.19	3.41E-03	7.87E-02	1.25E-01	0.81	0.99	SCLC
ieu-a-835	0.60	0.21	4.56E-03	1.06E-01	2.46E-01	0.98	1.00	SCLC
ieu-b-142	2.20	0.80	5.67E-03	3.46E-01	6.52E-01	1.00	0.97	SCLC
ieu-b-25	2.54	0.93	6.07E-03	2.16E-01	4.32E-01	1.00	0.99	SCLC
ieu-b-40	0.01	0.00	6.50E-03	6.86E-02	1.51E-01	0.99	0.96	SCLC
ieu-b-5118	0.15	0.06	1.08E-02	1.01E-01	2.22E-01	0.98	1.00	SCLC
prot-c-5102_55_3	-0.05	0.02	1.65E-02	4.54E-02	9.35E-02	1.00	0.97	SCLC
ukb-a-237	0.38	0.16	1.88E-02	3.85E-01	6.50E-01	1.00	1.00	SCLC
ukb-a-238	0.89	0.38	1.91E-02	1.90E-03	7.20E-03	0.91	0.54	SCLC
ukb-a-243	5.20	2.27	2.20E-02	1.85E-03	2.99E-03	0.94	0.78	SCLC
ukb-a-248	1.58	0.72	2.83E-02	2.12E-01	4.50E-01	0.95	1.00	SCLC
ukb-a-249	0.09	0.04	4.79E-02	4.04E-01	6.89E-01	1.00	1.00	SCLC
ukb-a-264	0.83	0.42	4.89E-02	3.15E-02	8.56E-02	0.99	0.96	SCLC
ukb-a-265	1.04	0.53	5.17E-02	8.13E-02	1.51E-01	0.98	1.00	SCLC
ukb-a-274	0.33	0.18	7.12E-02	4.10E-01	6.33E-01	0.91	0.97	SCLC
ukb-a-275	0.83	0.47	7.50E-02	1.91E-01	4.28E-01	1.00	0.99	SCLC
ukb-a-278	0.62	0.37	9.79E-02	7.34E-01	7.54E-01	1.00	0.97	SCLC
ukb-a-279	1.07	0.68	1.14E-01	5.21E-02	1.07E-01	0.99	0.97	SCLC
ukb-a-282	0.92	0.63	1.42E-01	7.61E-01	8.35E-01	1.00	0.97	SCLC
ukb-a-283	1.40	1.04	1.80E-01	3.71E-01	3.19E+00	1.00	1.00	SCLC

ukb-a-286	0.92	0.82	2.66E-01	3.61E-01	5.24E-01	0.98	0.97	SCLC
ukb-a-287	0.27	0.24	2.73E-01	1.87E-03	4.98E-03	0.91	0.62	SCLC
ukb-a-290	0.58	0.56	3.03E-01	4.28E-02	8.85E-02	0.96	1.00	SCLC
ukb-a-291	0.61	0.64	3.35E-01	5.15E-02	9.49E-02	0.98	1.00	SCLC
ukb-a-328	1.42	1.67	3.97E-01	3.90E-01	5.83E-01	1.00	0.97	SCLC
ukb-a-343	-2.26	2.91	4.37E-01	3.27E-01	8.11E-01	1.00	0.99	SCLC
ukb-a-382	1.02	1.36	4.52E-01	3.47E-01	9.88E-01	0.99	0.99	SCLC
ukb-a-397	-0.77	1.03	4.57E-01	1.65E-03	7.09E-03	0.89	0.39	SCLC
ukb-a-398	1.16	1.60	4.67E-01	3.68E-01	3.35E-01	0.59	1.00	SCLC
ukb-a-399	-1.16	1.63	4.76E-01	1.39E-03	6.41E-03	0.89	0.46	SCLC
ukb-a-40	-25.65	36.11	4.78E-01	1.36E-03	4.42E-03	0.89	0.46	SCLC
ukb-b-10831	1.84	2.62	4.82E-01	1.37E-03	5.56E-03	0.91	0.43	SCLC
ukb-b-11615	-0.88	1.31	5.03E-01	1.53E-03	5.46E-03	0.90	0.42	SCLC
ukb-b-11842	0.01	0.02	5.07E-01	1.37E-03	4.69E-03	0.91	0.42	SCLC
ukb-b-12018	4.90	7.56	5.17E-01	1.48E-03	7.70E-03	0.90	0.39	SCLC
ukb-b-12039	0.01	0.01	5.20E-01	1.40E-03	5.94E-03	0.92	0.42	SCLC
ukb-b-12854	0.13	0.21	5.26E-01	4.35E-02	1.17E-01	0.99	0.97	SCLC
ukb-b-13952	15.70	24.84	5.27E-01	1.35E-03	5.68E-03	0.91	0.47	SCLC
ukb-b-16489	-0.88	1.41	5.32E-01	1.47E-03	7.39E-03	0.92	0.38	SCLC
ukb-b-17729	1.16	1.88	5.39E-01	1.32E-03	8.74E-03	0.90	0.52	SCLC
ukb-b-18096	0.12	0.19	5.49E-01	4.13E-01	5.37E-01	0.94	0.97	SCLC
ukb-b-18377	0.06	0.11	5.51E-01	1.30E-03	1.70E-02	0.90	0.74	SCLC
ukb-b-19393	0.03	0.05	5.78E-01	1.50E-03	1.63E-02	0.87	0.69	SCLC
ukb-b-19953	0.03	0.05	6.11E-01	1.39E-03	5.91E-03	0.91	0.42	SCLC
ukb-b-20044	0.04	0.07	6.13E-01	1.39E-03	8.34E-03	0.91	0.38	SCLC

ukb-b-20188	0.15	0.30	6.25E-01	1.28E-03	2.49E-02	0.89	0.96	SCLC
ukb-b-20531	0.06	0.14	6.57E-01	1.30E-03	3.68E-02	0.89	1.17	SCLC
ukb-b-2303	0.04	0.08	6.59E-01	3.15E-01	4.90E-01	0.99	1.00	SCLC
ukb-b-2732	-2.70	8.30	7.45E-01	1.29E-03	4.89E-02	0.89	1.57	SCLC
ukb-b-3855	-3.60	12.87	7.79E-01	1.27E-03	5.53E-02	0.89	1.73	SCLC
ukb-b-4801	10.06	36.86	7.85E-01	1.21E-03	8.24E-02	0.89	2.60	SCLC
ukb-b-6019	1.05	3.85	7.85E-01	1.35E-03	1.08E-01	0.85	3.22	SCLC
ukb-b-6591	-1.03	3.78	7.85E-01	1.30E-03	6.34E-02	0.89	2.01	SCLC
ukb-b-6704	0.10	0.52	8.40E-01	1.20E-03	1.23E-01	0.88	3.85	SCLC
ukb-b-7212	0.15	0.87	8.62E-01	3.82E-01	6.21E-01	0.99	0.99	SCLC
ukb-b-7408	-1.24	7.37	8.66E-01	3.16E-01	2.07E-01	0.70	0.97	SCLC
ukb-b-7460	1.95	12.14	8.72E-01	1.10E-03	1.78E-01	0.87	6.31	SCLC
ukb-b-8338	0.09	0.56	8.74E-01	1.07E-03	1.46E-01	0.89	4.84	SCLC
ukb-b-8909	0.11	0.71	8.74E-01	1.13E-03	1.98E-01	0.87	6.46	SCLC
ukb-b-9405	0.08	0.96	9.31E-01	9.06E-04	2.49E-01	0.90	8.24	SCLC
ukb-d-30710_raw	0.02	0.40	9.54E-01	3.64E-01	9.71E-01	0.99	0.97	SCLC
ukb-d-COPD_EARLYANDLATER	16.67	471.86	9.72E-01	6.34E-01	2.88E-01	0.77	0.97	SCLC

supplementary Table 10 The MR results of between exposures and LC using MRAID method

name	beta	SE	p	sigmabeta	sigmaeta	sigma_error1	sigma_error2	type
bbj-a-73	-0.03	0.00	1.00E-50	0.00	0.00	0.96	0.99	Lung cancer
ebi-a-GCST004604	-0.10	0.01	1.00E-50	0.04	0.08	1.00	0.98	Lung cancer
ebi-a-GCST004622	-0.09	0.01	1.00E-50	0.00	0.01	0.87	0.83	Lung cancer
ebi-a-GCST006250	-0.22	0.01	1.00E-50	0.00	0.01	0.84	0.80	Lung cancer
ebi-a-GCST009965	0.00	0.00	1.00E-50	0.00	0.01	0.85	0.83	Lung cancer
ebi-a-GCST009966	-0.48	0.03	1.00E-50	0.00	0.00	0.96	0.99	Lung cancer
ebi-a-GCST009968	1.29	0.09	1.00E-50	0.00	0.01	0.99	0.99	Lung cancer
ebi-a-GCST009970	0.00	0.00	1.00E-50	0.00	0.00	0.83	0.72	Lung cancer
ebi-a-GCST009971	0.09	0.01	1.00E-50	0.18	0.51	0.98	0.98	Lung cancer
ebi-a-GCST90000045	-0.40	0.03	1.00E-50	0.00	0.00	0.96	0.92	Lung cancer
ebi-a-GCST90000046	-0.26	0.02	1.00E-50	0.02	0.04	0.99	0.97	Lung cancer
ebi-a-GCST90000047	-0.43	0.03	1.00E-50	0.02	0.03	0.99	0.97	Lung cancer
ebi-a-GCST90000048	-0.53	0.04	1.00E-50	0.00	0.01	0.96	0.99	Lung cancer
ebi-a-GCST90000050	-0.43	0.03	1.00E-50	0.00	0.01	0.87	0.87	Lung cancer
ebi-a-GCST90000514	0.33	0.02	1.00E-50	0.00	0.01	0.86	0.85	Lung cancer
ebi-a-GCST90006898	-0.24	0.02	1.00E-50	0.00	0.01	0.86	0.86	Lung cancer
ebi-a-GCST90006921	0.20	0.01	1.00E-50	0.00	0.01	0.90	0.82	Lung cancer
ebi-a-GCST90006923	0.38	0.03	1.00E-50	0.00	0.00	0.80	0.73	Lung cancer
ebi-a-GCST90006924	-0.43	0.03	1.00E-50	0.00	0.01	0.90	0.85	Lung cancer
ebi-a-GCST90012024	-0.09	0.01	1.00E-50	0.00	0.01	0.88	0.81	Lung cancer
ebi-a-GCST90012794	0.68	0.05	1.00E-50	0.00	0.01	0.83	0.75	Lung cancer
ebi-a-GCST90013870	2.45	0.16	1.00E-50	0.00	0.01	0.88	0.80	Lung cancer
ebi-a-GCST90013879	3.13	0.21	1.00E-50	0.00	0.01	0.90	0.85	Lung cancer
ebi-a-GCST90013922	5.66	0.38	1.00E-50	0.00	0.01	0.91	0.85	Lung cancer
ebi-a-GCST90013929	2.71	0.18	1.00E-50	0.00	0.01	0.88	0.79	Lung cancer
ebi-a-GCST90013972	5.95	0.40	1.00E-50	0.05	0.08	0.99	0.98	Lung cancer
ebi-a-GCST90013974	0.61	0.04	1.00E-50	0.00	0.00	0.96	0.98	Lung cancer
ebi-a-GCST90013975	-0.57	0.04	1.00E-50	0.00	0.01	0.89	0.81	Lung cancer
ebi-a-GCST90014020	0.28	0.02	1.00E-50	0.00	0.00	0.91	0.88	Lung cancer
ebi-a-GCST90014021	-0.07	0.01	4.44E-16	0.07	0.13	0.99	0.98	Lung cancer
ebi-a-GCST90014239	-1.75	0.22	4.44E-16	0.11	0.23	0.99	0.98	Lung cancer
ebi-a-GCST90016675	-0.24	0.03	6.66E-16	0.00	0.01	0.88	0.81	Lung cancer
ebi-a-GCST90018848	-6.42	0.79	6.66E-16	0.00	0.00	0.95	0.96	Lung cancer
ebi-a-GCST90018890	-1.07	0.14	2.66E-15	0.00	0.01	0.99	1.00	Lung cancer
ebi-a-GCST90018902	9.91	1.26	3.77E-15	0.00	0.01	0.95	1.00	Lung cancer
ebi-a-GCST90018947	2.31	0.30	1.11E-14	0.03	0.07	0.99	0.98	Lung cancer
ebi-a-GCST90018949	1.88	0.24	1.22E-14	0.13	0.29	0.99	1.00	Lung cancer
ebi-a-GCST90018982	-0.32	0.04	2.21E-13	0.00	0.01	0.99	1.00	Lung cancer
ebi-a-GCST90025972	-1.69	0.23	5.14E-13	0.01	0.01	0.99	1.00	Lung cancer
ebi-a-GCST90025994	0.10	0.01	1.25E-12	0.00	0.01	0.99	1.00	Lung cancer
ebi-a-GCST90027596	-0.62	0.09	1.47E-12	0.35	0.53	0.99	0.98	Lung cancer
ebi-a-GCST90029007	1.69	0.25	5.84E-12	0.00	0.01	0.99	1.00	Lung cancer

ebi-a-GCST90029012	-0.39	0.06	9.03E-12	0.01	0.02	0.99	1.00	Lung cancer
ebi-a-GCST90029013	-0.41	0.06	1.06E-11	0.04	0.10	0.99	0.98	Lung cancer
ebi-a-GCST90029014	0.61	0.09	1.44E-11	0.05	0.11	1.00	0.98	Lung cancer
ebi-a-GCST90029025	2.44	0.36	1.66E-11	0.00	0.00	0.93	1.00	Lung cancer
ebi-a-GCST90060127	0.17	0.03	2.35E-11	0.00	0.00	0.93	0.98	Lung cancer
ebi-a-GCST90060294	0.37	0.06	2.82E-11	0.00	0.01	0.89	0.75	Lung cancer
ebi-a-GCST90060337	0.22	0.03	2.83E-11	0.01	0.02	0.99	1.00	Lung cancer
ebi-a-GCST90060342	0.21	0.03	3.46E-11	0.03	0.07	0.99	0.98	Lung cancer
ebi-a-GCST90060514	0.00	0.00	4.77E-11	0.01	0.02	0.99	1.00	Lung cancer
ebi-a-GCST90093332	0.00	0.00	5.04E-11	0.00	0.01	0.96	1.00	Lung cancer
ebi-a-GCST90093341	-0.10	0.02	7.46E-11	0.00	0.00	0.92	0.97	Lung cancer
ebi-a-GCST90096909	-0.38	0.06	1.91E-10	0.01	0.02	0.99	1.00	Lung cancer
ebi-a-GCST90101889	0.01	0.00	5.22E-10	0.00	0.01	0.89	0.81	Lung cancer
eqtl-a-ENSG00000002016	-0.12	0.02	7.00E-10	0.00	0.01	0.99	1.00	Lung cancer
eqtl-a-ENSG00000010818	0.27	0.04	1.47E-09	0.00	0.00	0.93	0.99	Lung cancer
eqtl-a-ENSG00000026297	0.06	0.01	9.79E-09	0.00	0.00	0.90	1.00	Lung cancer
eqtl-a-ENSG00000041357	1.01	0.18	1.04E-08	0.00	0.00	0.91	0.95	Lung cancer
eqtl-a-ENSG00000087053	-0.27	0.05	1.16E-08	0.01	0.02	0.99	1.00	Lung cancer
eqtl-a-ENSG00000092330	0.79	0.14	1.77E-08	0.41	0.92	0.90	0.99	Lung cancer
eqtl-a-ENSG00000095261	-0.14	0.03	1.81E-08	0.04	0.08	1.00	0.98	Lung cancer
eqtl-a-ENSG00000100462	-0.09	0.02	2.39E-08	0.00	0.01	0.97	1.00	Lung cancer
eqtl-a-ENSG00000101574	-0.17	0.03	3.66E-08	0.00	0.00	0.93	1.00	Lung cancer
eqtl-a-ENSG00000101695	0.00	0.00	7.36E-08	0.39	0.55	1.00	0.99	Lung cancer
eqtl-a-ENSG00000103160	0.06	0.01	7.75E-08	0.01	0.02	0.99	1.00	Lung cancer
eqtl-a-ENSG00000106009	0.11	0.02	8.50E-08	0.00	0.01	0.99	1.00	Lung cancer
eqtl-a-ENSG00000107890	0.11	0.02	9.07E-08	0.39	0.57	0.98	1.00	Lung cancer
eqtl-a-ENSG00000111906	-0.05	0.01	9.13E-08	0.00	0.01	0.89	0.81	Lung cancer
eqtl-a-ENSG00000112812	0.05	0.01	1.59E-07	0.01	0.03	1.00	1.00	Lung cancer
eqtl-a-ENSG00000119402	0.29	0.06	4.31E-07	0.14	0.25	1.00	0.98	Lung cancer
eqtl-a-ENSG00000119403	0.11	0.02	4.66E-07	0.01	0.02	0.98	1.00	Lung cancer
eqtl-a-ENSG00000119487	0.37	0.08	8.54E-07	0.02	0.04	1.00	1.00	Lung cancer
eqtl-a-ENSG00000130755	0.35	0.07	1.54E-06	0.02	0.06	1.00	1.00	Lung cancer
eqtl-a-ENSG00000134851	-0.09	0.02	2.93E-06	0.00	0.01	0.89	0.86	Lung cancer
eqtl-a-ENSG00000138496	-0.29	0.06	2.97E-06	0.01	0.02	1.00	1.00	Lung cancer
eqtl-a-ENSG00000139531	0.06	0.01	5.54E-06	0.20	0.50	0.99	1.00	Lung cancer
eqtl-a-ENSG00000141298	0.07	0.02	5.81E-06	0.00	0.01	0.91	0.85	Lung cancer
eqtl-a-ENSG00000141499	-1.08	0.24	6.29E-06	0.00	0.01	0.88	1.00	Lung cancer
eqtl-a-ENSG00000145414	-0.47	0.10	7.00E-06	0.07	0.15	1.00	1.00	Lung cancer
eqtl-a-ENSG00000145416	0.05	0.01	1.95E-05	0.01	0.02	1.00	1.00	Lung cancer
eqtl-a-ENSG00000149573	-0.04	0.01	2.10E-05	0.07	0.19	0.95	1.00	Lung cancer
eqtl-a-ENSG00000156414	-0.07	0.02	2.24E-05	0.04	0.06	0.76	1.00	Lung cancer
eqtl-a-ENSG00000158406	0.05	0.01	3.55E-05	0.10	0.22	1.00	0.98	Lung cancer
eqtl-a-ENSG00000165905	0.22	0.05	3.62E-05	0.15	0.04	2.56	1.00	Lung cancer
eqtl-a-ENSG00000166037	-0.09	0.02	4.48E-05	0.10	0.22	1.00	1.00	Lung cancer
eqtl-a-ENSG00000166763	-0.05	0.01	5.66E-05	0.18	0.25	1.00	1.00	Lung cancer
eqtl-a-ENSG00000167004	0.28	0.07	6.29E-05	0.01	0.02	0.99	1.00	Lung cancer

eqtl-a-ENSG00000167483	0.18	0.04	7.42E-05	0.02	0.06	1.00	1.00	Lung cancer
eqtl-a-ENSG00000168411	-0.11	0.03	8.45E-05	0.01	0.03	0.99	1.00	Lung cancer
eqtl-a-ENSG00000169045	-0.05	0.01	8.54E-05	0.00	0.01	0.94	1.00	Lung cancer
eqtl-a-ENSG00000169554	0.81	0.21	9.20E-05	0.08	0.19	0.99	1.00	Lung cancer
eqtl-a-ENSG00000171055	0.02	0.01	1.03E-04	0.03	0.08	0.91	1.00	Lung cancer
eqtl-a-ENSG00000174007	-0.06	0.02	1.06E-04	0.36	0.67	1.00	1.00	Lung cancer
eqtl-a-ENSG00000174749	-0.04	0.01	1.07E-04	0.19	0.23	0.67	1.00	Lung cancer
eqtl-a-ENSG00000175164	0.03	0.01	1.17E-04	0.20	0.28	0.63	1.00	Lung cancer
eqtl-a-ENSG00000177508	0.09	0.02	1.17E-04	0.00	0.00	0.91	0.93	Lung cancer
eqtl-a-ENSG00000177706	-0.08	0.02	1.26E-04	0.02	0.07	1.00	1.00	Lung cancer
eqtl-a-ENSG00000179361	-0.23	0.06	1.89E-04	0.04	0.11	0.96	1.00	Lung cancer
eqtl-a-ENSG00000184056	0.15	0.04	2.30E-04	0.02	0.06	1.00	1.00	Lung cancer
eqtl-a-ENSG00000185361	0.23	0.07	3.13E-04	0.00	0.01	0.99	1.00	Lung cancer
eqtl-a-ENSG00000188266	-3.47	0.97	3.49E-04	0.09	0.25	1.00	1.00	Lung cancer
eqtl-a-ENSG00000189298	0.00	0.00	3.68E-04	0.13	0.29	1.00	1.00	Lung cancer
eqtl-a-ENSG00000196812	0.23	0.07	4.11E-04	0.07	0.18	0.99	1.00	Lung cancer
eqtl-a-ENSG00000197077	0.08	0.02	4.38E-04	0.36	0.73	0.99	1.00	Lung cancer
eqtl-a-ENSG00000198518	0.32	0.09	5.27E-04	0.25	0.08	2.29	1.00	Lung cancer
eqtl-a-ENSG00000198643	-0.32	0.09	6.20E-04	0.19	0.22	0.67	1.00	Lung cancer
eqtl-a-ENSG00000204592	0.55	0.16	6.35E-04	0.14	0.32	1.00	1.00	Lung cancer
eqtl-a-ENSG00000211785	0.42	0.12	7.01E-04	0.39	0.43	0.59	1.00	Lung cancer
eqtl-a-ENSG00000223534	-0.05	0.01	7.07E-04	0.10	0.23	1.00	1.00	Lung cancer
eqtl-a-ENSG00000227598	0.03	0.01	7.52E-04	0.22	0.44	1.00	1.00	Lung cancer
eqtl-a-ENSG00000239415	0.05	0.01	7.54E-04	0.02	0.07	1.00	1.00	Lung cancer
eqtl-a-ENSG00000243753	0.29	0.09	7.98E-04	0.19	0.34	0.98	1.00	Lung cancer
eqtl-a-ENSG00000259015	0.06	0.02	8.08E-04	0.08	0.16	0.93	1.00	Lung cancer
eqtl-a-ENSG00000260103	-0.01	0.00	8.35E-04	0.04	0.10	0.94	1.00	Lung cancer
finn-b-AB1_INFECTIONS	-0.10	0.03	8.62E-04	0.01	0.03	0.99	1.00	Lung cancer
finn-b-AB1_VIRAL_SKIN_MUCOUS_MEMBRANE	0.04	0.01	9.12E-04	0.21	0.42	1.00	1.00	Lung cancer
finn-b-AD_LO	-0.01	0.00	9.26E-04	0.20	0.45	1.00	1.00	Lung cancer
finn-b-C3_RESPIRATORY_INTRATHORACIC	0.23	0.07	9.37E-04	0.04	0.11	1.00	1.00	Lung cancer
finn-b-C3_RESPIRATORY_INTRATHORACIC_EXALLC	0.41	0.12	9.77E-04	0.08	0.20	1.00	1.00	Lung cancer
finn-b-COPD_HOSPITAL	0.05	0.01	9.88E-04	0.13	0.21	0.87	1.00	Lung cancer
finn-b-COPD_LATER	0.37	0.12	1.20E-03	0.19	0.38	0.99	1.00	Lung cancer
finn-b-D3_ANAEMIA_B12_DEF	0.03	0.01	1.23E-03	0.20	0.37	0.99	1.00	Lung cancer
finn-b-DM_NEPHROPATHY_EXMORE	0.08	0.02	1.24E-03	0.13	0.30	0.99	1.00	Lung cancer
finn-b-DM_PERIPHATHERO	-0.04	0.01	1.31E-03	0.00	0.00	0.92	0.92	Lung cancer

finn-b-DM_VITREOUS_BLEEDING	0.04	0.01	1.33E-03	0.11	0.26	0.99	1.00	Lung cancer
finn-b-E4_GLUCOPANCREAS	0.06	0.02	1.37E-03	0.10	0.25	0.99	1.00	Lung cancer
finn-b-G6_ALZHEIMER	-0.01	0.00	1.50E-03	0.20	0.52	1.00	1.00	Lung cancer
finn-b-H7_VITRHAEMORR	0.04	0.01	1.59E-03	0.19	0.46	1.00	1.00	Lung cancer
finn-b-I9_CARDARR	-0.04	0.01	1.60E-03	0.14	0.29	1.00	1.00	Lung cancer
finn-b-I9_REVASC	-0.02	0.00	1.65E-03	0.36	0.55	1.00	1.00	Lung cancer
finn-b-J10_COPDNAS_INCLAVO	0.05	0.02	1.68E-03	0.13	0.36	0.99	1.00	Lung cancer
finn-b-R18_ABNORMAL_FINDI_EXAMI_OTHER_BODY_FLUIDS_SUBST_TISSU_WO_DIAGNOSIS	0.08	0.03	1.74E-03	0.09	0.18	0.96	1.00	Lung cancer
finn-b-R18_ABNORMAL_FINDI_SECRE_SMEARS_CERVIX_UTERI_VAGINA_VULVA	0.08	0.03	2.08E-03	0.19	0.40	0.99	1.00	Lung cancer
finn-b-RHEU_ARTHRITIS_OTH	0.04	0.01	2.09E-03	0.03	0.06	0.94	1.00	Lung cancer
ieu-a-1001	-0.48	0.16	2.28E-03	0.35	0.81	0.99	1.00	Lung cancer
ieu-a-1239	-0.90	0.30	2.52E-03	0.02	0.07	1.00	1.00	Lung cancer
ieu-b-142	1.28	0.43	3.01E-03	0.13	0.32	0.99	1.00	Lung cancer
ieu-b-25	1.48	0.50	3.21E-03	0.18	0.44	1.00	1.00	Lung cancer
ieu-b-40	2.17	0.74	3.32E-03	0.20	0.42	0.99	1.00	Lung cancer
ieu-b-4877	0.61	0.21	3.45E-03	0.01	0.03	1.00	1.00	Lung cancer
ieu-b-4879	0.36	0.12	3.49E-03	0.00	0.00	0.97	0.99	Lung cancer
ieu-b-5117	0.22	0.07	3.97E-03	0.06	0.13	0.97	1.00	Lung cancer
ieu-b-5118	-0.37	0.13	4.50E-03	0.06	0.12	0.98	1.00	Lung cancer
met-a-510	0.40	0.14	4.65E-03	0.04	0.10	0.95	1.00	Lung cancer
prot-a-1051	-0.04	0.02	4.90E-03	0.19	0.40	0.99	1.00	Lung cancer
prot-a-1124	0.01	0.00	5.05E-03	1.01	0.44	0.68	1.00	Lung cancer
prot-a-1238	-0.15	0.05	5.60E-03	0.20	0.43	1.00	1.00	Lung cancer
prot-a-1347	-0.06	0.02	5.89E-03	0.01	0.02	1.00	1.00	Lung cancer
prot-a-2470	-0.03	0.01	6.14E-03	0.34	0.66	1.00	1.00	Lung cancer
prot-a-2481	0.08	0.03	6.41E-03	0.11	0.25	0.95	1.00	Lung cancer
prot-a-725	0.00	0.00	6.67E-03	0.10	0.22	1.00	1.00	Lung cancer
prot-a-885	0.27	0.10	6.73E-03	0.30	0.75	0.99	1.00	Lung cancer
prot-c-4964_67_1	0.02	0.01	6.76E-03	0.17	0.24	0.76	1.00	Lung cancer
prot-c-5102_55_3	-0.07	0.03	7.00E-03	0.04	0.06	0.86	1.00	Lung cancer
ubm-a-3048	0.58	0.22	7.99E-03	0.37	0.91	0.99	1.00	Lung cancer
ubm-b-1447	-0.68	0.26	8.58E-03	0.14	0.33	0.98	1.00	Lung cancer
ubm-b-1564	-0.23	0.09	8.99E-03	0.31	0.61	1.00	0.98	Lung cancer
ubm-b-1571	0.41	0.16	9.20E-03	0.02	0.04	0.99	1.00	Lung cancer
ubm-b-289	0.25	0.10	9.38E-03	0.84	0.66	1.00	1.00	Lung cancer
ubm-b-789	0.11	0.04	9.57E-03	0.01	0.01	0.99	1.00	Lung cancer
ubm-b-847	0.33	0.13	1.17E-02	0.03	0.06	0.94	1.00	Lung cancer

ukb-a-132	-0.73	0.30	1.45E-02	0.38	0.99	1.00	1.00	Lung cancer
ukb-a-142	-0.28	0.12	1.54E-02	0.00	0.00	0.91	0.96	Lung cancer
ukb-a-180	-1.17	0.49	1.67E-02	0.43	0.64	1.00	1.00	Lung cancer
ukb-a-201	-0.41	0.17	1.83E-02	0.00	0.00	0.93	1.00	Lung cancer
ukb-a-202	0.97	0.42	2.07E-02	0.03	0.07	0.96	1.00	Lung cancer
ukb-a-205	6.49	2.81	2.10E-02	0.37	0.54	1.00	1.00	Lung cancer
ukb-a-217	-0.37	0.16	2.20E-02	0.36	0.87	1.00	1.00	Lung cancer
ukb-a-237	0.75	0.33	2.39E-02	0.02	0.03	0.92	1.00	Lung cancer
ukb-a-238	0.68	0.30	2.44E-02	0.49	1.39	1.00	0.98	Lung cancer
ukb-a-248	0.33	0.15	2.44E-02	0.03	0.07	0.97	1.00	Lung cancer
ukb-a-249	2.48	1.13	2.89E-02	0.00	0.01	0.84	0.72	Lung cancer
ukb-a-264	0.24	0.11	3.16E-02	0.06	0.12	0.98	1.00	Lung cancer
ukb-a-265	0.28	0.13	3.37E-02	0.00	0.00	0.90	0.93	Lung cancer
ukb-a-272	-0.14	0.07	3.37E-02	0.12	0.10	0.76	1.00	Lung cancer
ukb-a-273	0.00	0.00	3.59E-02	0.02	0.03	0.91	1.00	Lung cancer
ukb-a-274	0.48	0.23	3.77E-02	0.36	3.23	1.00	1.00	Lung cancer
ukb-a-275	0.32	0.15	3.88E-02	0.00	0.01	0.89	0.78	Lung cancer
ukb-a-277	-0.13	0.07	4.52E-02	0.30	0.67	0.80	1.00	Lung cancer
ukb-a-278	0.20	0.10	4.93E-02	0.14	0.17	1.00	0.99	Lung cancer
ukb-a-279	0.13	0.07	5.56E-02	0.47	0.64	1.00	1.00	Lung cancer
ukb-a-281	1.27	0.67	5.99E-02	0.00	0.01	0.89	0.80	Lung cancer
ukb-a-282	0.24	0.13	6.99E-02	0.96	0.71	0.91	1.00	Lung cancer
ukb-a-283	0.12	0.07	7.62E-02	0.48	0.61	0.94	0.99	Lung cancer
ukb-a-284	1.75	1.00	8.16E-02	0.26	0.07	2.79	1.00	Lung cancer
ukb-a-286	0.15	0.09	8.33E-02	0.00	0.01	0.89	0.74	Lung cancer
ukb-a-287	0.08	0.05	1.09E-01	0.04	0.09	0.97	1.00	Lung cancer
ukb-a-291	0.24	0.15	1.17E-01	0.02	0.02	0.61	1.00	Lung cancer
ukb-a-298	6.17	3.95	1.18E-01	0.32	0.55	1.00	1.00	Lung cancer
ukb-a-328	1.30	0.85	1.28E-01	0.39	0.63	0.89	1.00	Lung cancer
ukb-a-342	0.76	0.53	1.52E-01	0.00	0.00	0.90	0.98	Lung cancer
ukb-a-344	0.45	0.32	1.67E-01	0.00	0.01	0.84	0.72	Lung cancer
ukb-a-382	0.12	0.09	1.69E-01	0.00	0.00	0.92	0.95	Lung cancer
ukb-a-397	-0.27	0.20	1.79E-01	0.37	0.67	1.00	1.00	Lung cancer
ukb-a-398	0.39	0.29	1.85E-01	0.04	0.10	0.98	1.00	Lung cancer
ukb-a-399	-0.50	0.39	1.98E-01	0.60	0.93	0.76	1.00	Lung cancer
ukb-a-40	-11.52	9.06	2.03E-01	0.21	0.48	0.96	1.00	Lung cancer
ukb-a-434	-0.50	0.41	2.17E-01	0.34	0.60	1.00	1.00	Lung cancer
ukb-a-5	0.36	0.30	2.24E-01	0.35	0.85	1.00	1.00	Lung cancer
ukb-a-505	-0.46	0.38	2.30E-01	0.38	0.54	1.00	1.00	Lung cancer
ukb-b-10831	2.20	1.87	2.40E-01	0.33	0.82	1.00	0.99	Lung cancer
ukb-b-10911	-2.52	2.22	2.57E-01	0.55	0.77	0.85	1.00	Lung cancer
ukb-b-11188	1.29	1.14	2.57E-01	0.10	0.12	0.93	1.00	Lung cancer
ukb-b-11268	-0.27	0.24	2.60E-01	0.47	0.43	0.99	1.00	Lung cancer
ukb-b-11615	-0.60	0.54	2.65E-01	0.07	0.10	0.89	1.00	Lung cancer
ukb-b-12018	1.61	1.48	2.77E-01	0.00	0.01	0.88	0.77	Lung cancer

ukb-b-12039	-0.15	0.14	2.84E-01	0.00	0.01	0.86	0.83	Lung cancer
ukb-b-12405	-0.36	0.35	3.01E-01	0.12	0.19	0.91	1.00	Lung cancer
ukb-b-12854	3.18	3.17	3.16E-01	0.00	0.00	0.92	0.97	Lung cancer
ukb-b-13799	-0.67	0.66	3.17E-01	0.00	0.00	0.91	0.95	Lung cancer
ukb-b-13952	7.99	8.21	3.30E-01	0.00	0.00	0.82	0.70	Lung cancer
ukb-b-14206	1.57	1.62	3.32E-01	0.02	0.05	0.94	1.00	Lung cancer
ukb-b-14521	2.38	2.46	3.34E-01	0.00	0.01	0.91	0.86	Lung cancer
ukb-b-14540	2.08	2.16	3.36E-01	0.08	0.17	0.99	1.00	Lung cancer
ukb-b-15169	0.99	1.04	3.40E-01	0.60	0.68	0.90	0.98	Lung cancer
ukb-b-1553	0.75	0.79	3.47E-01	0.51	0.94	1.00	1.00	Lung cancer
ukb-b-15590	-0.87	0.93	3.49E-01	0.04	0.08	0.97	1.00	Lung cancer
ukb-b-15797	1.30	1.45	3.69E-01	0.20	0.41	1.00	1.00	Lung cancer
ukb-b-16019	6.99	7.82	3.72E-01	0.28	0.59	0.85	1.00	Lung cancer
ukb-b-16407	2.21	2.55	3.85E-01	0.79	0.59	0.99	1.00	Lung cancer
ukb-b-16446	-0.19	0.22	3.89E-01	0.38	0.51	0.99	1.00	Lung cancer
ukb-b-16489	-0.54	0.63	3.94E-01	0.00	0.01	0.86	0.74	Lung cancer
ukb-b-16698	-0.35	0.42	4.13E-01	0.35	0.53	0.99	1.00	Lung cancer
ukb-b-16878	-0.52	0.63	4.14E-01	0.48	0.75	1.00	1.00	Lung cancer
ukb-b-17670	0.93	1.16	4.23E-01	0.00	0.01	0.86	0.74	Lung cancer
ukb-b-17729	0.62	0.78	4.26E-01	0.48	0.58	0.99	1.00	Lung cancer
ukb-b-18096	2.46	3.31	4.58E-01	0.05	0.10	0.96	1.00	Lung cancer
ukb-b-18377	2.70	3.70	4.66E-01	0.18	0.44	1.00	1.00	Lung cancer
ukb-b-18802	-0.20	0.29	4.81E-01	0.21	0.38	0.96	1.00	Lung cancer
ukb-b-19379	0.00	0.00	4.83E-01	0.35	0.51	1.00	1.00	Lung cancer
ukb-b-19393	2.83	4.19	4.99E-01	0.53	0.62	0.87	1.00	Lung cancer
ukb-b-19520	2.03	3.06	5.06E-01	0.00	0.01	0.90	0.82	Lung cancer
ukb-b-19698	3.05	4.73	5.19E-01	0.62	0.68	0.85	1.00	Lung cancer
ukb-b-19842	-1.66	2.62	5.25E-01	0.43	0.41	0.94	1.00	Lung cancer
ukb-b-19921	0.16	0.26	5.30E-01	0.38	0.68	1.00	1.00	Lung cancer
ukb-b-19925	0.09	0.15	5.46E-01	0.00	0.01	0.89	0.80	Lung cancer
ukb-b-19953	0.30	0.53	5.72E-01	0.50	0.64	1.00	1.00	Lung cancer
ukb-b-20044	2.63	4.80	5.84E-01	0.31	0.64	1.00	1.00	Lung cancer
ukb-b-20188	2.33	4.62	6.13E-01	0.00	0.01	0.89	0.85	Lung cancer
ukb-b-20261	0.32	0.63	6.16E-01	0.38	0.34	0.94	1.00	Lung cancer
ukb-b-20531	2.70	5.78	6.41E-01	0.39	0.75	0.94	1.00	Lung cancer
ukb-b-2134	-0.52	1.22	6.67E-01	0.00	0.01	0.89	0.80	Lung cancer
ukb-b-2205	-0.99	2.44	6.86E-01	0.00	0.00	0.84	0.70	Lung cancer
ukb-b-2303	2.44	6.08	6.88E-01	0.44	0.62	0.92	1.00	Lung cancer
ukb-b-2399	-1.79	4.57	6.95E-01	0.23	0.44	0.95	1.00	Lung cancer
ukb-b-2732	-0.51	1.45	7.25E-01	0.35	0.38	1.00	1.00	Lung cancer
ukb-b-3599	0.90	2.75	7.44E-01	0.41	0.55	0.94	1.00	Lung cancer
ukb-b-4667	-0.58	1.98	7.70E-01	0.37	0.49	1.00	1.00	Lung cancer
ukb-b-469	0.76	2.68	7.77E-01	0.34	0.79	1.00	1.00	Lung cancer
ukb-b-5174	0.66	2.44	7.87E-01	0.18	0.43	1.00	1.00	Lung cancer
ukb-b-5192	0.35	1.35	7.97E-01	0.39	0.79	1.00	1.00	Lung cancer
ukb-b-6019	1.15	4.53	8.00E-01	2.28	0.52	0.84	1.00	Lung cancer

ukb-b-6134	-0.55	2.37	8.16E-01	0.21	0.34	0.72	1.00	Lung cancer
ukb-b-6306	0.46	2.24	8.39E-01	0.59	0.60	0.99	1.00	Lung cancer
ukb-b-6591	-0.48	2.93	8.69E-01	0.14	0.27	0.97	1.00	Lung cancer
ukb-b-6704	0.65	4.94	8.96E-01	0.44	0.25	0.97	1.00	Lung cancer
ukb-b-7212	2.20	17.05	8.97E-01	0.38	0.35	0.97	1.00	Lung cancer
ukb-b-7408	-0.54	4.86	9.11E-01	0.32	0.45	1.00	1.00	Lung cancer
ukb-b-7460	0.96	9.44	9.19E-01	0.45	0.75	1.00	1.00	Lung cancer
ukb-b-7647	8.13	83.09	9.22E-01	0.26	0.45	0.91	1.00	Lung cancer
ukb-b-8184	-1.41	16.69	9.33E-01	0.37	0.73	1.00	1.00	Lung cancer
ukb-b-8338	2.58	34.61	9.41E-01	0.37	0.61	1.00	1.00	Lung cancer
ukb-b-8909	0.73	11.87	9.51E-01	0.46	0.56	1.00	1.00	Lung cancer
ukb-b-9093	-0.31	5.02	9.51E-01	0.38	0.28	0.97	1.00	Lung cancer
ukb-b-9405	1.42	27.03	9.58E-01	0.43	0.29	0.98	0.98	Lung cancer
ukb-b-969	0.43	12.05	9.72E-01	0.09	0.17	0.98	1.00	Lung cancer
ukb-d-1448_4	1.25	35.43	9.72E-01	0.22	0.28	0.97	1.00	Lung cancer
ukb-d-20116_0	-0.52	18.01	9.77E-01	0.86	0.28	0.98	0.99	Lung cancer
ukb-d-30020_irnt	-0.08	3.50	9.81E-01	0.00	0.00	0.90	0.92	Lung cancer
ukb-d-I9_PAD	-1.54	647.47	9.98E-01	0.00	0.01	0.87	0.77	Lung cancer

supplementary Table 11 The MR results of between exposures and LUSC using MRAID method

name	beta	SE	p	sigmabeta	sigmaeta	sigma_error1	sigma_error2	type
ebi-a-GCST004604	-0.24	0.02	1.00E-50	2.19E-03	4.71E-03	0.96	0.98	LUSC
ebi-a-GCST006250	-0.37	0.03	1.00E-50	2.04E-03	4.42E-03	0.97	0.99	LUSC
ebi-a-GCST006696	-1.28	0.09	1.00E-50	2.04E-03	4.28E-03	0.96	0.99	LUSC
ebi-a-GCST007432	-0.59	0.04	1.00E-50	2.90E-03	7.70E-03	0.98	0.99	LUSC
ebi-a-GCST009966	-0.79	0.05	1.00E-50	1.34E-01	2.93E-01	0.98	0.97	LUSC
ebi-a-GCST009967	1.60	0.11	1.00E-50	1.70E-02	3.66E-02	0.99	0.95	LUSC
ebi-a-GCST009968	1.17	0.08	1.00E-50	1.66E-02	3.48E-02	0.99	0.95	LUSC
ebi-a-GCST009970	0.15	0.01	1.00E-50	1.32E-01	3.53E-01	1.00	0.96	LUSC
ebi-a-GCST009971	0.14	0.01	1.00E-50	2.22E-03	4.61E-03	0.96	0.98	LUSC
ebi-a-GCST011365	-0.44	0.05	2.22E-16	6.67E-02	1.55E-01	0.99	0.97	LUSC
ebi-a-GCST90000045	-0.82	0.10	4.22E-15	4.94E-03	1.27E-02	0.98	0.99	LUSC
ebi-a-GCST90000046	-0.47	0.06	6.33E-14	4.24E-03	1.13E-02	0.99	0.99	LUSC
ebi-a-GCST90000047	-0.57	0.08	8.99E-14	2.39E-03	5.18E-03	0.96	0.99	LUSC
ebi-a-GCST90000048	-1.03	0.14	1.95E-13	5.89E-02	1.28E-01	0.99	0.97	LUSC
ebi-a-GCST90000050	-0.70	0.10	2.12E-13	4.76E-03	1.34E-02	0.99	0.99	LUSC
ebi-a-GCST90000514	1.09	0.15	3.89E-13	1.27E-03	6.18E-03	0.96	0.71	LUSC
ebi-a-GCST90006921	0.63	0.09	2.51E-11	4.39E-03	1.14E-02	0.99	0.99	LUSC
ebi-a-GCST90006923	0.16	0.02	2.83E-11	2.07E-03	2.30E-03	0.93	0.91	LUSC
ebi-a-GCST90006924	0.47	0.07	4.98E-11	5.71E-03	1.58E-02	0.99	0.99	LUSC
ebi-a-GCST90006929	0.52	0.08	2.80E-10	4.99E-03	1.36E-02	0.99	0.99	LUSC
ebi-a-GCST90012877	-0.52	0.08	3.46E-10	7.78E-03	2.23E-02	1.00	0.99	LUSC
ebi-a-GCST90013870	0.24	0.04	7.57E-10	3.56E-03	8.84E-03	0.99	0.99	LUSC
ebi-a-GCST90013922	4.82	0.80	2.05E-09	6.87E-03	1.95E-02	0.99	0.99	LUSC
ebi-a-GCST90013972	5.56	0.95	5.06E-09	1.35E-01	2.67E-01	0.99	0.99	LUSC
ebi-a-GCST90013974	0.48	0.08	1.74E-08	4.92E-03	1.46E-02	0.99	1.00	LUSC
ebi-a-GCST90013975	0.27	0.05	3.74E-08	4.83E-03	2.65E-03	0.57	0.88	LUSC
ebi-a-GCST90014020	0.00	0.00	1.89E-07	6.20E-03	1.69E-02	0.99	1.00	LUSC
ebi-a-GCST90016675	-0.41	0.08	2.31E-07	4.31E-01	4.04E-01	0.59	0.99	LUSC
ebi-a-GCST90018793	-0.58	0.11	2.99E-07	1.85E-03	2.42E-03	0.91	0.85	LUSC
ebi-a-GCST90018848	-8.37	1.65	3.69E-07	1.91E-01	3.92E-01	0.99	1.00	LUSC
ebi-a-GCST90018890	-2.43	0.49	5.76E-07	1.86E-03	2.74E-03	0.91	0.86	LUSC
ebi-a-GCST90018902	20.36	4.18	1.11E-06	1.71E-03	2.33E-03	0.95	0.88	LUSC
ebi-a-GCST90018934	-0.38	0.08	1.31E-06	1.12E-02	3.25E-02	0.99	1.00	LUSC
ebi-a-GCST90018947	0.59	0.13	2.71E-06	2.23E-01	3.72E-01	0.99	1.00	LUSC
ebi-a-GCST90018949	0.13	0.03	3.30E-06	7.94E-02	1.86E-01	1.00	1.00	LUSC
ebi-a-GCST90018982	-0.72	0.16	3.37E-06	1.52E-02	4.26E-02	0.99	1.00	LUSC
ebi-a-GCST90018990	-0.26	0.06	3.72E-06	4.67E-01	9.24E-01	1.00	0.98	LUSC
ebi-a-GCST90018992	-1.71	0.37	3.82E-06	2.94E-02	8.14E-02	0.99	0.97	LUSC
ebi-a-GCST90019017	-0.10	0.02	4.13E-06	1.84E-01	2.18E-01	0.63	1.00	LUSC
ebi-a-GCST90019476	0.20	0.04	4.32E-06	2.10E-03	3.07E-03	0.92	0.84	LUSC
ebi-a-GCST90025994	0.24	0.05	6.00E-06	5.98E-03	1.55E-02	0.99	1.00	LUSC
ebi-a-GCST90029007	0.37	0.08	7.11E-06	6.05E-03	1.58E-02	0.99	0.99	LUSC
ebi-a-GCST90029012	-0.62	0.14	7.58E-06	3.58E-03	9.32E-03	0.99	0.99	LUSC
ebi-a-GCST90029013	-0.59	0.14	1.12E-05	7.26E-03	1.97E-02	0.99	1.00	LUSC

ebi-a-GCST90029014	0.90	0.21	1.96E-05	3.35E-02	6.83E-02	0.90	1.00	LUSC
ebi-a-GCST90060467	-0.47	0.11	2.37E-05	4.78E-03	1.23E-02	0.97	1.00	LUSC
ebi-a-GCST90060470	-0.46	0.11	2.48E-05	1.23E-01	3.19E-01	1.00	1.00	LUSC
ebi-a-GCST90093332	-0.41	0.10	3.67E-05	4.21E-02	6.18E-02	0.78	0.99	LUSC
ebi-a-GCST90093341	-0.78	0.19	3.84E-05	5.48E-02	1.36E-01	1.00	1.00	LUSC
ebi-a-GCST90104006	-0.34	0.08	4.30E-05	4.09E-03	8.97E-03	0.94	0.99	LUSC
eqtl-a-ENSG00000041357	1.20	0.29	4.62E-05	1.03E-02	2.90E-02	0.99	1.00	LUSC
eqtl-a-ENSG00000060709	0.26	0.06	5.09E-05	1.97E-01	4.65E-01	1.00	1.00	LUSC
eqtl-a-ENSG00000063438	-0.37	0.09	5.74E-05	1.79E-01	5.34E-02	1.66	1.00	LUSC
eqtl-a-ENSG00000072163	0.20	0.05	5.77E-05	3.47E-01	5.75E-01	1.00	0.98	LUSC
eqtl-a-ENSG00000074657	-0.21	0.05	5.94E-05	2.16E-02	5.55E-02	1.00	1.00	LUSC
eqtl-a-ENSG00000100450	0.34	0.09	6.26E-05	1.35E-01	4.55E-02	1.82	1.00	LUSC
eqtl-a-ENSG00000106305	-0.23	0.06	6.44E-05	3.51E-01	6.38E-01	1.00	0.98	LUSC
eqtl-a-ENSG00000107593	0.57	0.14	6.84E-05	6.56E-02	1.54E-01	1.00	1.00	LUSC
eqtl-a-ENSG00000107890	0.28	0.07	7.28E-05	1.44E-01	3.20E-02	3.04	0.99	LUSC
eqtl-a-ENSG00000108384	0.10	0.03	9.01E-05	2.71E-02	7.09E-02	0.99	0.97	LUSC
eqtl-a-ENSG00000111725	-0.18	0.04	9.28E-05	2.09E-03	3.75E-03	0.94	0.98	LUSC
eqtl-a-ENSG00000111906	-0.05	0.01	1.05E-04	3.38E-01	7.14E-01	1.00	1.00	LUSC
eqtl-a-ENSG00000134193	-1.02	0.26	1.13E-04	2.20E-02	6.15E-02	1.00	1.00	LUSC
eqtl-a-ENSG00000134758	-0.20	0.05	1.16E-04	8.69E-02	1.89E-01	0.96	1.00	LUSC
eqtl-a-ENSG00000137338	0.76	0.20	1.28E-04	2.25E-01	3.94E-01	0.96	1.00	LUSC
eqtl-a-ENSG00000140650	0.14	0.04	1.34E-04	8.17E-02	2.17E-01	0.98	1.00	LUSC
eqtl-a-ENSG00000145476	0.09	0.02	1.40E-04	1.33E-01	2.92E-01	0.99	0.98	LUSC
eqtl-a-ENSG00000155026	0.41	0.11	1.49E-04	5.81E-03	1.65E-02	0.99	1.00	LUSC
eqtl-a-ENSG00000159873	-0.40	0.11	2.08E-04	1.00E-01	2.76E-01	1.00	1.00	LUSC
eqtl-a-ENSG00000162736	0.12	0.03	2.12E-04	9.88E-03	2.79E-02	1.00	1.00	LUSC
eqtl-a-ENSG00000163788	-0.50	0.14	2.27E-04	2.65E-03	5.74E-03	0.96	0.99	LUSC
eqtl-a-ENSG00000164961	3.06	0.84	2.64E-04	6.70E-03	1.92E-02	0.99	1.00	LUSC
eqtl-a-ENSG00000166763	-0.10	0.03	2.65E-04	9.69E-02	2.11E-01	1.00	1.00	LUSC
eqtl-a-ENSG00000167004	0.77	0.21	2.66E-04	3.37E-01	6.65E-02	3.49	1.00	LUSC
eqtl-a-ENSG00000173744	-0.38	0.10	2.78E-04	1.98E-03	2.73E-03	0.94	0.88	LUSC
eqtl-a-ENSG00000175164	0.03	0.01	2.87E-04	8.76E-02	2.05E-01	0.95	1.00	LUSC
eqtl-a-ENSG00000188266	-4.88	1.35	2.97E-04	9.10E-02	2.05E-01	0.96	1.00	LUSC
eqtl-a-ENSG00000189298	0.24	0.07	3.14E-04	1.53E-01	5.63E-02	1.47	1.00	LUSC
eqtl-a-ENSG00000196812	0.37	0.11	5.34E-04	6.26E-02	1.45E-01	0.97	1.00	LUSC
eqtl-a-ENSG00000198518	0.59	0.17	5.99E-04	3.32E-02	9.79E-02	1.00	1.00	LUSC
eqtl-a-ENSG00000204435	0.67	0.20	6.12E-04	2.28E-02	5.74E-02	1.00	1.00	LUSC
eqtl-a-ENSG00000204592	0.77	0.23	6.98E-04	1.16E-01	7.06E-02	0.51	1.00	LUSC
eqtl-a-ENSG00000205726	-0.16	0.05	7.50E-04	9.79E-02	2.02E-01	0.92	1.00	LUSC
eqtl-a-ENSG00000205978	-0.26	0.08	7.66E-04	1.45E-02	3.97E-02	0.99	0.99	LUSC
eqtl-a-ENSG00000211785	1.24	0.37	9.14E-04	1.47E-01	7.02E-02	0.78	1.00	LUSC
eqtl-a-ENSG00000223534	-0.02	0.01	1.01E-03	4.09E-01	5.53E-01	0.99	0.99	LUSC
eqtl-a-ENSG00000229515	-0.09	0.03	1.02E-03	1.91E-01	4.52E-01	0.99	1.00	LUSC
eqtl-a-ENSG00000243753	0.69	0.21	1.04E-03	1.52E-02	4.28E-02	0.99	0.99	LUSC
eqtl-a-ENSG00000250366	0.26	0.08	1.08E-03	3.32E-02	8.72E-02	0.99	0.97	LUSC
eqtl-a-ENSG00000260228	-0.03	0.01	1.13E-03	1.55E-01	5.28E-02	1.26	1.00	LUSC

eqtl-a-ENSG00000260276	0.13	0.04	1.18E-03	3.57E-01	7.18E-02	3.47	1.00	LUSC
finn-b-AB1_ARTHROPOD	0.01	0.00	1.19E-03	7.40E-02	1.72E-01	0.96	1.00	LUSC
finn-b-AB1_INFECTIONS	0.01	0.00	1.22E-03	2.70E-03	6.00E-03	0.96	0.99	LUSC
finn-b-AB1_VIRAL_HEMOR_FEVER_NOS	0.00	0.00	1.45E-03	9.60E-02	2.37E-01	1.00	1.00	LUSC
finn-b-C3_RESPIRATORY_INTRATHORACIC	0.00	0.00	1.46E-03	1.34E-02	3.83E-02	1.00	1.00	LUSC
finn-b-C3_RESPIRATORY_INTRATHORACIC_EXALLC	0.00	0.00	1.65E-03	3.25E-01	7.14E-01	0.99	1.00	LUSC
finn-b-CD2_BENIGN_EXALLC	-0.04	0.01	1.73E-03	8.12E-02	2.07E-01	0.99	1.00	LUSC
finn-b-CD2_BENIGN_LEIOMYOMA_UTERI	-0.03	0.01	1.85E-03	2.24E-01	3.60E-01	0.98	1.00	LUSC
finn-b-CD2_BENIGN_LEIOMYOMA_UTERI_EXALLC	-0.03	0.01	1.86E-03	1.13E-01	2.54E-01	0.96	1.00	LUSC
finn-b-COPD_LATER	0.00	0.00	1.94E-03	1.04E-01	2.24E-01	0.99	1.00	LUSC
finn-b-DM_NEPHROPATHY_EXMORE	0.06	0.02	2.09E-03	1.20E-01	4.05E-02	1.58	1.00	LUSC
finn-b-DM_PERIPHATHERO	-0.09	0.03	2.10E-03	2.33E-01	4.47E-01	0.95	1.00	LUSC
finn-b-DM_VITREOUS_BLEEDING	0.01	0.00	2.14E-03	2.11E-01	3.94E-01	0.97	1.00	LUSC
finn-b-E4_GLUCOPANCREAS	0.01	0.00	2.19E-03	7.47E-03	2.09E-02	0.99	1.00	LUSC
finn-b-F5_DEMENTIA	-0.02	0.01	2.35E-03	1.37E-01	2.18E-01	0.68	1.00	LUSC
finn-b-F5_DEMENTIA_INCLAVO	-0.02	0.01	2.44E-03	1.98E-03	2.39E-03	0.93	0.92	LUSC
finn-b-H7_VITRHAEMORR	0.02	0.01	2.44E-03	2.09E-01	3.79E-01	1.00	1.00	LUSC
finn-b-I9_ANGINA	-0.04	0.01	2.61E-03	1.94E-01	4.76E-01	0.99	1.00	LUSC
finn-b-I9_CABG	-0.04	0.01	2.84E-03	1.96E-01	9.71E-01	1.00	1.00	LUSC
finn-b-I9_CABG_EXNONE	-0.04	0.01	3.37E-03	1.04E-01	2.49E-01	1.00	1.00	LUSC
finn-b-I9_CARDARR	0.00	0.00	3.66E-03	4.23E-01	8.63E-01	0.99	1.00	LUSC
finn-b-I9_CORATHER	-0.03	0.01	3.80E-03	1.37E-01	2.75E-01	1.00	1.00	LUSC
finn-b-I9_CORATHER_EXNONE	-0.03	0.01	4.48E-03	1.01E-01	1.55E-01	0.99	0.97	LUSC
finn-b-I9_POSTAMI	0.00	0.00	4.83E-03	4.49E-02	8.42E-02	1.00	0.96	LUSC
finn-b-I9_REVASC	-0.04	0.01	5.64E-03	1.33E-01	2.68E-01	1.00	1.00	LUSC
finn-b-J10_COPDNAS	0.41	0.15	5.83E-03	2.36E-01	5.07E-01	0.99	1.00	LUSC
finn-b-J10_COPDNAS_INCLAVO	0.21	0.08	5.97E-03	3.34E-02	9.92E-02	1.00	1.00	LUSC
finn-b-OTHER_SYSTCON_FG	0.00	0.00	6.42E-03	8.64E-02	1.95E-01	0.96	1.00	LUSC
finn-b-R18_ABNORMAL_FINDI_EXAMI_OTHER_BODY_FLUIDS_SUBST_TISSU_WO_DIAGNOSIS	0.00	0.00	6.52E-03	1.89E-03	2.41E-03	0.91	0.89	LUSC
finn-b-R18_ABNORMAL_FINDI_SECRE_SMEARS_CERVIX_UTERI_VAGINA_VULVA	0.00	0.00	7.72E-03	3.23E-02	6.37E-02	0.91	1.00	LUSC
finn-b-Z21_SPECIAL_SCREEN_EXAM_OTH_DISEA_DISORD	0.00	0.00	8.20E-03	1.16E-01	4.25E-02	1.54	1.00	LUSC
ieu-a-1001	-0.68	0.26	8.82E-03	5.54E-03	1.41E-02	0.99	0.99	LUSC
ieu-a-1239	-2.91	1.14	1.06E-02	3.34E-01	8.83E-01	0.99	1.00	LUSC
ieu-b-104	-0.22	0.09	1.31E-02	3.71E-02	8.63E-02	0.96	1.00	LUSC
ieu-b-142	2.10	0.86	1.42E-02	7.15E-01	5.98E-01	1.00	1.00	LUSC
ieu-b-24	-3.18	1.31	1.47E-02	3.92E-01	8.97E-01	0.98	1.00	LUSC
ieu-b-25	2.47	1.02	1.55E-02	3.47E-01	5.90E-01	1.00	0.98	LUSC
ieu-b-40	0.21	0.09	1.67E-02	1.04E-01	2.26E-01	0.98	1.00	LUSC
ieu-b-4877	1.04	0.43	1.67E-02	4.45E-02	7.46E-02	1.00	0.96	LUSC
ieu-b-5113	-0.40	0.17	1.81E-02	4.19E-01	7.94E-01	1.00	1.00	LUSC
ieu-b-5117	0.33	0.14	2.11E-02	5.71E-02	1.05E-01	0.95	1.00	LUSC
ieu-b-5118	0.09	0.04	2.12E-02	5.84E-01	5.57E-01	0.77	0.97	LUSC
prot-a-1347	-0.25	0.11	2.15E-02	8.01E-02	1.52E-01	0.96	1.00	LUSC
prot-a-21	0.67	0.29	2.24E-02	1.83E-03	2.64E-03	0.92	0.86	LUSC
prot-a-2481	0.25	0.11	2.31E-02	3.55E-01	6.38E-01	1.00	1.00	LUSC

prot-a-3203	0.09	0.04	2.45E-02	3.27E-01	6.97E-01	1.00	0.99	LUSC
prot-a-710	-0.07	0.03	2.62E-02	1.47E-02	4.09E-02	1.00	1.00	LUSC
prot-a-746	-0.42	0.20	3.20E-02	3.68E-03	8.58E-03	0.97	0.99	LUSC
prot-a-885	0.65	0.30	3.23E-02	2.39E-02	5.58E-02	0.94	1.00	LUSC
prot-c-5102_55_3	-0.13	0.06	3.34E-02	3.49E-01	7.30E-01	1.00	1.00	LUSC
prot-c-5312_49_3	0.00	0.00	3.36E-02	1.47E-03	1.26E-02	0.87	0.52	LUSC
prot-c-5494_52_3	0.01	0.00	4.30E-02	3.46E-02	7.46E-02	0.99	0.97	LUSC
ubm-b-2149	1.35	0.67	4.31E-02	4.49E-01	6.20E-01	1.00	1.00	LUSC
ubm-b-2246	-1.19	0.59	4.54E-02	7.67E-03	2.07E-02	0.99	0.99	LUSC
ubm-b-3277	-0.66	0.33	4.56E-02	3.69E-01	6.31E-01	1.00	1.00	LUSC
ubm-b-571	-1.51	0.76	4.83E-02	1.92E-03	2.32E-03	0.93	0.89	LUSC
ubm-b-623	1.16	0.60	5.38E-02	3.50E-01	8.03E-01	1.00	0.99	LUSC
ubm-b-687	0.88	0.46	5.46E-02	1.08E-01	1.47E-01	0.82	1.00	LUSC
ubm-b-789	-3.60	1.90	5.77E-02	3.53E-01	6.01E-01	1.00	1.00	LUSC
ukb-a-132	-1.88	0.99	5.88E-02	1.14E-01	1.77E-01	0.93	1.00	LUSC
ukb-a-176	-0.85	0.46	6.47E-02	3.90E-01	6.44E-01	0.99	1.00	LUSC
ukb-a-201	-0.78	0.45	8.49E-02	8.81E-03	1.66E-02	0.86	1.00	LUSC
ukb-a-202	2.05	1.21	9.01E-02	2.73E-01	4.27E-01	0.80	1.00	LUSC
ukb-a-205	9.72	5.87	9.80E-02	1.31E-01	1.97E-01	0.99	0.98	LUSC
ukb-a-237	1.10	0.68	1.04E-01	3.61E-01	3.02E-01	0.70	0.98	LUSC
ukb-a-238	0.97	0.60	1.05E-01	1.35E-01	2.88E-01	1.00	0.98	LUSC
ukb-a-248	0.00	0.00	1.09E-01	1.48E-01	3.18E-01	0.99	1.00	LUSC
ukb-a-249	0.92	0.59	1.15E-01	1.98E-03	2.77E-03	0.93	0.86	LUSC
ukb-a-264	0.28	0.18	1.20E-01	1.94E-03	2.27E-03	0.93	0.92	LUSC
ukb-a-265	0.00	0.00	1.25E-01	7.63E-01	5.45E-01	0.99	1.00	LUSC
ukb-a-274	0.17	0.11	1.31E-01	1.01E-01	1.49E-01	1.00	0.97	LUSC
ukb-a-275	0.65	0.44	1.35E-01	1.78E-03	3.10E-03	0.92	0.85	LUSC
ukb-a-278	0.74	0.50	1.39E-01	1.28E-03	9.45E-03	0.90	0.53	LUSC
ukb-a-279	0.33	0.22	1.39E-01	1.47E-03	1.30E-02	0.89	0.57	LUSC
ukb-a-282	0.31	0.21	1.45E-01	1.31E-03	9.65E-03	0.89	0.52	LUSC
ukb-a-283	0.30	0.21	1.48E-01	1.35E-03	1.14E-02	0.90	0.52	LUSC
ukb-a-286	0.38	0.28	1.64E-01	1.22E-03	7.42E-03	0.88	0.53	LUSC
ukb-a-287	0.17	0.12	1.64E-01	4.17E-01	3.88E+00	0.99	1.00	LUSC
ukb-a-291	0.63	0.45	1.68E-01	1.35E-03	1.14E-02	0.91	0.53	LUSC
ukb-a-298	7.77	5.68	1.71E-01	1.27E-03	8.53E-03	0.89	0.53	LUSC
ukb-a-328	1.90	1.44	1.87E-01	1.38E-03	1.04E-02	0.89	0.52	LUSC
ukb-a-342	0.91	0.69	1.87E-01	3.10E-01	5.50E-01	1.00	1.00	LUSC
ukb-a-343	-1.03	0.78	1.88E-01	3.29E-01	1.38E+00	0.99	0.99	LUSC
ukb-a-345	4.55	3.53	1.98E-01	1.18E-03	6.46E-03	0.89	0.52	LUSC
ukb-a-382	0.29	0.23	2.06E-01	1.28E-03	9.19E-03	0.89	0.54	LUSC
ukb-a-397	-0.45	0.36	2.07E-01	1.41E-03	1.07E-02	0.91	0.53	LUSC
ukb-a-398	0.80	0.65	2.20E-01	3.09E-02	7.23E-02	0.97	1.00	LUSC
ukb-a-399	-0.91	0.75	2.21E-01	1.20E-03	7.12E-03	0.88	0.53	LUSC
ukb-a-40	-9.56	7.83	2.22E-01	1.46E-03	1.33E-02	0.91	0.60	LUSC
ukb-a-434	-1.15	0.95	2.23E-01	1.37E-03	1.16E-02	0.91	0.53	LUSC
ukb-b-10756	-4.50	3.74	2.30E-01	1.30E-03	8.63E-03	0.89	0.52	LUSC

ukb-b-10831	1.41	1.19	2.35E-01	3.24E-01	6.16E-01	0.99	1.00	LUSC
ukb-b-10911	-9.42	7.97	2.37E-01	1.06E-03	4.90E-03	0.89	0.52	LUSC
ukb-b-11075	-2.37	2.00	2.38E-01	3.39E-01	6.87E-01	1.00	1.00	LUSC
ukb-b-11615	-1.02	0.87	2.42E-01	1.28E-03	9.36E-03	0.89	0.53	LUSC
ukb-b-11842	0.05	0.05	2.46E-01	3.29E-01	6.63E-01	0.99	1.00	LUSC
ukb-b-12039	0.22	0.19	2.50E-01	1.39E-03	1.23E-02	0.91	0.60	LUSC
ukb-b-12405	-0.56	0.49	2.51E-01	1.10E-03	4.78E-03	0.87	0.52	LUSC
ukb-b-12687	4.60	4.01	2.52E-01	3.78E-02	5.84E-02	0.86	1.00	LUSC
ukb-b-12841	-1.14	0.99	2.52E-01	1.39E-03	1.05E-02	0.88	0.53	LUSC
ukb-b-12854	0.31	0.27	2.62E-01	3.71E-01	6.10E-01	0.99	0.98	LUSC
ukb-b-13799	-1.30	1.18	2.71E-01	1.30E-03	1.02E-02	0.89	0.54	LUSC
ukb-b-13952	13.51	12.46	2.78E-01	3.69E-01	7.35E-01	1.00	0.99	LUSC
ukb-b-14206	-4.96	4.60	2.81E-01	4.31E-01	3.26E+00	0.99	0.98	LUSC
ukb-b-14521	5.08	4.76	2.85E-01	3.25E-01	7.24E-01	1.00	1.00	LUSC
ukb-b-1489	-0.67	0.63	2.86E-01	4.27E-01	6.40E-01	1.00	1.00	LUSC
ukb-b-15169	1.76	1.65	2.87E-01	3.29E-01	6.24E-01	1.00	0.98	LUSC
ukb-b-15590	0.26	0.24	2.90E-01	1.19E-03	6.85E-03	0.88	0.54	LUSC
ukb-b-1572	0.82	0.78	2.98E-01	1.89E-01	2.96E-01	1.00	0.98	LUSC
ukb-b-15797	-5.23	5.16	3.11E-01	1.30E-03	9.40E-03	0.89	0.53	LUSC
ukb-b-16019	9.77	10.08	3.32E-01	1.61E-03	1.27E-02	0.87	0.61	LUSC
ukb-b-16407	0.18	0.19	3.34E-01	8.99E-04	3.45E-03	0.90	0.52	LUSC
ukb-b-16489	-0.84	0.88	3.42E-01	3.80E-01	1.11E+00	1.00	1.00	LUSC
ukb-b-1668	-0.87	0.93	3.45E-01	4.16E-01	5.48E-01	0.99	1.00	LUSC
ukb-b-16878	-0.87	1.01	3.86E-01	3.42E-01	5.78E-01	0.99	1.00	LUSC
ukb-b-17685	0.98	1.16	3.96E-01	1.45E-03	1.26E-02	0.91	0.57	LUSC
ukb-b-17729	1.01	1.22	4.05E-01	8.24E-02	1.26E-01	0.90	1.00	LUSC
ukb-b-18096	0.36	0.44	4.16E-01	3.73E-01	9.49E-01	1.00	1.00	LUSC
ukb-b-18377	0.37	0.46	4.18E-01	1.09E-03	3.39E-03	0.87	0.52	LUSC
ukb-b-18408	-0.72	1.05	4.94E-01	2.38E-02	5.49E-02	1.00	0.97	LUSC
ukb-b-1867	3.96	6.15	5.20E-01	1.16E-01	8.67E-02	0.76	0.99	LUSC
ukb-b-18802	-4.59	7.27	5.28E-01	1.36E-01	2.20E-01	0.87	1.00	LUSC
ukb-b-19379	-0.21	0.36	5.66E-01	1.34E-03	6.07E-03	0.85	0.52	LUSC
ukb-b-19393	0.36	0.66	5.81E-01	5.44E-01	6.44E-01	0.76	1.00	LUSC
ukb-b-19698	-1.99	4.06	6.23E-01	3.83E-01	8.29E-01	0.99	1.00	LUSC
ukb-b-19842	-2.16	4.85	6.57E-01	3.89E-01	5.51E-01	0.99	1.00	LUSC
ukb-b-19921	-0.16	0.41	6.99E-01	1.39E-03	1.24E-02	0.91	0.56	LUSC
ukb-b-19953	0.34	0.95	7.21E-01	4.20E-01	5.22E-01	1.00	1.00	LUSC
ukb-b-20044	0.17	0.54	7.49E-01	3.91E-01	4.18E-01	1.00	1.00	LUSC
ukb-b-20188	0.18	0.58	7.59E-01	1.08E-03	3.82E-03	0.87	0.52	LUSC
ukb-b-20531	0.04	0.14	7.59E-01	3.19E-01	5.38E-01	1.00	1.00	LUSC
ukb-b-2303	0.36	1.44	8.04E-01	4.92E-01	5.20E-01	0.89	1.00	LUSC
ukb-b-2732	-1.02	4.11	8.05E-01	3.03E-01	2.99E-01	0.99	1.00	LUSC
ukb-b-469	1.03	4.52	8.20E-01	3.75E-01	5.49E-01	1.00	0.99	LUSC
ukb-b-4801	9.13	41.88	8.27E-01	5.26E-01	3.15E-01	0.85	1.00	LUSC
ukb-b-5192	0.73	3.39	8.30E-01	4.63E-01	3.85E-01	0.89	1.00	LUSC
ukb-b-6019	2.10	11.39	8.54E-01	3.79E-01	5.15E-01	0.98	0.98	LUSC

ukb-b-6134	-0.70	4.83	8.85E-01	4.55E-01	3.81E-01	0.84	1.00	LUSC
ukb-b-6306	0.57	4.40	8.97E-01	4.76E-01	3.96E-01	0.86	1.00	LUSC
ukb-b-6591	-0.71	6.24	9.10E-01	3.78E-01	4.93E-01	1.00	1.00	LUSC
ukb-b-6704	0.37	3.38	9.13E-01	4.90E-01	3.19E-01	0.87	1.00	LUSC
ukb-b-7212	0.40	3.83	9.18E-01	1.64E-03	9.88E-03	0.92	0.68	LUSC
ukb-b-7408	-1.07	11.05	9.23E-01	3.75E-01	4.02E-01	0.89	1.00	LUSC
ukb-b-7460	1.53	16.53	9.26E-01	5.61E-01	3.60E-01	0.91	1.00	LUSC
ukb-b-7647	2.17	34.28	9.50E-01	4.38E-01	3.04E-01	0.89	1.00	LUSC
ukb-b-7859	-0.27	4.91	9.56E-01	3.81E-01	8.56E-01	1.00	1.00	LUSC
ukb-b-8133	-2.50	47.63	9.58E-01	5.12E-01	2.60E-01	0.91	0.98	LUSC
ukb-b-8338	0.28	7.95	9.72E-01	1.85E-03	2.75E-03	0.90	0.84	LUSC
ukb-b-8755	-1.41	43.49	9.74E-01	4.62E-01	2.89E-01	0.88	1.00	LUSC
ukb-b-8909	0.32	14.07	9.82E-01	3.76E-01	2.65E-01	0.94	1.00	LUSC
ukb-b-9405	0.21	10.44	9.84E-01	4.20E-01	3.17E-01	0.94	0.98	LUSC
ukb-d-20116_0	-0.70	60.77	9.91E-01	4.50E-01	2.92E-01	0.91	0.98	LUSC
ukb-d-COPD_EARLYANDLATER	12.21	######	9.96E-01	4.40E-01	2.78E-01	0.92	1.00	LUSC
ukb-d-I9_CORATHER	-0.88	208.74	9.97E-01	1.86E-03	4.37E-03	0.91	0.80	LUSC
ukb-d-I9_IHD	-0.97	307.54	9.97E-01	4.03E-01	2.92E-01	0.92	1.00	LUSC
ukb-d-III_BLOOD_IMMUN	4.33	######	9.99E-01	4.08E-01	2.51E-01	0.94	1.00	LUSC

Supplementary table 12 The detailed results of of two-step Mendelian randomization

exposure	content	outcome	method	nsnp	b	se	pval
age of first birth		LUAD	MR Egger	4428	-0.08	0.02	1.26E-04
age of first birth		LUAD	Weighted median	4428	-0.08	0.01	2.18E-13
age of first birth	GCST90000048, GCST90000050, UKB-b-12405	LUAD	ivw	4428	-0.09	0.01	1.65E-29
age of first birth		LUAD	Simple mode	4428	-0.03	0.08	6.73E-01
age of first birth		LUAD	Weighted mode	4428	-0.05	0.08	5.17E-01
bmi		LUAD	MR Egger	15526	0.30	0.06	2.53E-06
bmi	UKB-b-19953, GCST90029007, ieu-b-40, ieu-a-835, UKB-b-2303, UKB-a-248	LUAD	Weighted median	15526	0.28	0.05	6.40E-10
bmi		LUAD	ivw	15526	0.30	0.03	2.55E-27
bmi		LUAD	Simple mode	15526	0.00	0.36	9.91E-01
bmi		LUAD	Weighted mode	15526	0.12	0.24	6.28E-01
education	ebi-a-GCST90029012, ebi-a-GCST90029013, ieu-a-1001, ieu-a-1239, ukb-a-397, ukb-a-398, ukb-a-399, ukb-b-11615, ukb-b-13799, ukb-b-16489, ukb-b-17729	LUAD	MR Egger	12913	-0.54	0.15	3.81E-04
education		LUAD	Weighted median	12913	-0.75	0.09	7.42E-16
education		LUAD	ivw	12913	-0.75	0.06	3.02E-34
education		LUAD	Simple mode	12913	-2.40	0.85	4.72E-03
education		LUAD	Weighted mode	12913	-0.76	0.69	2.74E-01
fat_mass	ukb-a-265, ukb-a-275, ukb-a-279, ukb-a-283, ukb-a-287, ukb-a-291, ukb-a-264, ukb-a-278, ukb-a-282, ukb-a-286, ukb-a-290	LUAD	MR Egger	47577	-0.14	0.04	3.45E-04
fat_mass		LUAD	Weighted median	47577	-0.19	0.03	2.43E-10
fat_mass		LUAD	ivw	47577	-0.19	0.02	2.73E-25
fat_mass		LUAD	Simple mode	47577	-0.13	0.32	6.89E-01
fat_mass		LUAD	Weighted mode	47577	-0.06	0.23	8.05E-01
fat_percentage		LUAD	MR Egger	1863	0.29	0.18	1.09E-01
fat_percentage		LUAD	Weighted median	1863	0.19	0.19	3.22E-01

fat_percentage	ukb-b-8909，ukb-b-12854，ukb-b-16407，ukb-b-18377，ukb-b-20188，ukb-b-20531	LUAD	ivw	1863	0.37	0.10	1.42E-04
fat_percentage		LUAD	Simple mode	1863	0.18	0.63	7.75E-01
fat_percentage		LUAD	Weighted mode	1863	0.18	0.32	5.77E-01
age of first sex	GCST006368，GCST90013870，GCST90013974，GCST90025994，UKB-a-505，GCST90000045，GCST90000046，GCST90000047，UKB-b-6591，UKB-b-6134	LUAD	MR Egger	1825	-0.55	0.14	1.64E-04
age of first sex		LUAD	Weighted median	1825	-0.59	0.07	4.75E-19
age of first sex		LUAD	ivw	1825	-0.58	0.05	2.33E-36
age of first sex		LUAD	Simple mode	1825	-0.51	0.36	1.55E-01
age of first sex		LUAD	Weighted mode	1825	-0.54	0.31	8.63E-02
smoke	UKB-b-469，ieu-b-142，GCST009965，GCST009966，GCST009968，GCST009970，UKB-b-6019，ieu-b-25，ieu-b-4877 ， UKB-a-342，UKB-a-328	LUAD	MR Egger	35	3.61	0.33	1.63E-12
smoke		LUAD	Weighted median	35	3.25	0.28	1.23E-31
smoke		LUAD	ivw	35	2.90	0.22	1.02E-38
smoke		LUAD	Simple mode	35	2.09	0.99	4.08E-02
smoke		LUAD	Weighted mode	35	3.16	0.28	5.23E-13
age of first birth		SCLC	MR Egger	4212	-0.04	0.03	1.72E-01
age of first birth		SCLC	Weighted median	4212	-0.09	0.02	4.23E-07
age of first birth		SCLC	ivw	4212	-0.08	0.01	3.53E-11
age of first birth		SCLC	Simple mode	4212	-0.21	0.13	1.07E-01
age of first birth		SCLC	Weighted mode	4212	-0.21	0.12	7.38E-02
bmi		SCLC	MR Egger	14790	0.42	0.10	5.92E-05
bmi		SCLC	Weighted median	14790	0.50	0.08	8.56E-11

bmi	SCLC	ivw	14790	0.45	0.04	1.80E-25
bmi	SCLC	Simple mode	14790	0.09	0.58	8.75E-01
bmi	SCLC	Weighted mode	14790	0.36	0.32	2.69E-01
education	SCLC	MR Egger	12288	-1.49	0.28	1.57E-07
education	SCLC	Weighted median	12288	-1.88	0.18	8.11E-26
education	SCLC	ivw	12288	-1.76	0.11	2.48E-54
education	SCLC	Simple mode	12288	-2.02	1.47	1.69E-01
education	SCLC	Weighted mode	12288	-2.74	1.19	2.18E-02
fat_mass	SCLC	MR Egger	43691	-0.23	0.06	2.28E-04
fat_mass	SCLC	Weighted median	43691	-0.33	0.05	3.72E-11
fat_mass	SCLC	ivw	43691	-0.29	0.03	2.10E-22
fat_mass	SCLC	Simple mode	43691	-0.44	0.52	4.00E-01
fat_mass	SCLC	Weighted mode	43691	-0.44	0.31	1.58E-01
fat_percentage	SCLC	MR Egger	1487	-0.05	0.31	8.78E-01
fat_percentage	SCLC	Weighted median	1487	0.15	0.29	6.09E-01
fat_percentage	SCLC	ivw	1487	0.48	0.17	3.47E-03
fat_percentage	SCLC	Simple mode	1487	-0.42	1.10	6.99E-01
fat_percentage	SCLC	Weighted mode	1487	-0.03	0.55	9.63E-01
age of first sex	SCLC	MR Egger	1796	-0.43	0.24	7.20E-02
age of first sex	SCLC	Weighted median	1796	-0.74	0.11	5.43E-12
age of first sex	SCLC	ivw	1796	-0.71	0.07	9.67E-23
age of first sex	SCLC	Simple mode	1796	-1.28	0.63	4.23E-02
age of first sex	SCLC	Weighted mode	1796	-1.07	0.51	3.77E-02
smoke	SCLC	MR Egger	38	3.94	0.51	3.43E-09
smoke	SCLC	Weighted median	38	3.45	0.44	3.74E-15
smoke	SCLC	ivw	38	3.06	0.32	2.60E-22

smoke	SCLC	Simple mode	38	1.96	1.16	9.81E-02
smoke	SCLC	Weighted mode	38	3.35	0.45	6.93E-09
age of first birth	Lung cancer	MR Egger	4407	-0.03	0.01	2.33E-02
age of first birth	Lung cancer	Weighted median	4407	-0.06	0.01	5.98E-18
age of first birth	Lung cancer	ivw	4407	-0.06	0.00	1.13E-34
age of first birth	Lung cancer	Simple mode	4407	-0.02	0.05	7.20E-01
age of first birth	Lung cancer	Weighted mode	4407	-0.09	0.05	8.20E-02
bmi	Lung cancer	MR Egger	15481	0.15	0.04	2.60E-04
bmi	Lung cancer	Weighted median	15481	0.19	0.03	7.93E-12
bmi	Lung cancer	ivw	15481	0.18	0.02	4.58E-27
bmi	Lung cancer	Simple mode	15481	0.05	0.24	8.20E-01
bmi	Lung cancer	Weighted mode	15481	0.16	0.16	3.21E-01
education	Lung cancer	MR Egger	12950	-0.77	0.11	2.15E-12
education	Lung cancer	Weighted median	12950	-0.98	0.07	1.38E-45
education	Lung cancer	ivw	12950	-1.00	0.04	2.34E-112
education	Lung cancer	Simple mode	12950	-0.66	0.62	2.91E-01
education	Lung cancer	Weighted mode	12950	-0.93	0.51	6.91E-02
fat_mass	Lung cancer	MR Egger	47947	-0.09	0.02	1.87E-04
fat_mass	Lung cancer	Weighted median	47947	-0.16	0.02	9.18E-17
fat_mass	Lung cancer	ivw	47947	-0.15	0.01	1.14E-36
fat_mass	Lung cancer	Simple mode	47947	-0.17	0.19	3.62E-01
fat_mass	Lung cancer	Weighted mode	47947	-0.17	0.15	2.59E-01
fat_percentage	Lung cancer	MR Egger	1872	0.30	0.11	9.74E-03
fat_percentage	Lung cancer	Weighted median	1872	0.21	0.11	6.02E-02
fat_percentage	Lung cancer	ivw	1872	0.27	0.06	8.40E-06
fat_percentage	Lung cancer	Simple mode	1872	-0.27	0.37	4.63E-01

fat_percentage	Lung cancer	Weighted mode	1872	0.17	0.20	3.89E-01
age of first sex	Lung cancer	MR Egger	1775	-0.50	0.09	7.46E-08
age of first sex	Lung cancer	Weighted median	1775	-0.44	0.04	6.48E-25
age of first sex	Lung cancer	ivw	1775	-0.45	0.03	6.62E-54
age of first sex	Lung cancer	Simple mode	1775	-0.37	0.25	1.38E-01
age of first sex	Lung cancer	Weighted mode	1775	-0.46	0.21	2.69E-02
smoke	Lung cancer	MR Egger	30	1.77	0.44	3.60E-04
smoke	Lung cancer	Weighted median	30	1.78	0.28	2.31E-10
smoke	Lung cancer	ivw	30	2.06	0.20	4.03E-25
smoke	Lung cancer	Simple mode	30	1.38	0.46	5.77E-03
smoke	Lung cancer	Weighted mode	30	1.55	0.38	3.30E-04
age of first birth	LUSC	MR Egger	4428	-0.08	0.02	1.26E-04
age of first birth	LUSC	Weighted median	4428	-0.08	0.01	1.12E-12
age of first birth	LUSC	ivw	4428	-0.09	0.01	1.65E-29
age of first birth	LUSC	Simple mode	4428	-0.03	0.09	6.89E-01
age of first birth	LUSC	Weighted mode	4428	-0.05	0.08	5.34E-01
bmi	LUSC	MR Egger	15526	0.30	0.06	2.53E-06
bmi	LUSC	Weighted median	15526	0.28	0.05	4.45E-09
bmi	LUSC	ivw	15526	0.30	0.03	2.55E-27
bmi	LUSC	Simple mode	15526	0.00	0.38	9.92E-01
bmi	LUSC	Weighted mode	15526	0.12	0.22	5.95E-01

education	LUSC	MR Egger	12887	-1.25	0.18	1.45E-12
education	LUSC	Weighted median	12887	-1.42	0.11	3.38E-37
education	LUSC	ivw	12887	-1.33	0.07	3.01E-77
education	LUSC	Simple mode	12887	-1.65	0.94	8.02E-02
education	LUSC	Weighted mode	12887	-1.92	0.70	6.23E-03
fat_mass	LUSC	MR Egger	47577	-0.14	0.04	3.45E-04
fat_mass	LUSC	Weighted median	47577	-0.19	0.03	1.49E-10
fat_mass	LUSC	ivw	47577	-0.19	0.02	2.73E-25
fat_mass	LUSC	Simple mode	47577	-0.13	0.32	6.90E-01
fat_mass	LUSC	Weighted mode	47577	-0.06	0.22	7.99E-01
fat_percentage	LUSC	MR Egger	1863	0.29	0.18	1.09E-01
fat_percentage	LUSC	Weighted median	1863	0.19	0.18	3.11E-01
fat_percentage	LUSC	ivw	1863	0.37	0.10	1.42E-04
fat_percentage	LUSC	Simple mode	1863	0.18	0.65	7.81E-01
fat_percentage	LUSC	Weighted mode	1863	0.18	0.34	5.96E-01
age of first sex	LUSC	MR Egger	1825	-0.55	0.14	1.64E-04
age of first sex	LUSC	Weighted median	1825	-0.59	0.06	4.01E-20
age of first sex	LUSC	ivw	1825	-0.58	0.05	2.33E-36
age of first sex	LUSC	Simple mode	1825	-0.51	0.38	1.84E-01
age of first sex	LUSC	Weighted mode	1825	-0.54	0.31	8.17E-02
smoke	LUSC	MR Egger	35	3.61	0.33	1.63E-12
smoke	LUSC	Weighted median	35	3.25	0.29	1.22E-28
smoke	LUSC	ivw	35	2.90	0.22	1.02E-38
smoke	LUSC	Simple mode	35	2.09	0.96	3.53E-02
smoke	LUSC	Weighted mode	35	3.16	0.29	1.36E-12

education	age of first birth	MR Egger	10652	4.11	0.06	0.00E+00
education	age of first birth	Weighted median	10652	4.08	0.04	0.00E+00
education	age of first birth	ivw	10652	4.26	0.03	0.00E+00
education	age of first birth	Simple mode	10652	4.29	0.43	3.25E-23
education	age of first birth	Weighted mode	10652	4.19	0.34	2.14E-35
education	bmi	MR Egger	8245	-0.46	0.01	0.00E+00
education	bmi	Weighted median	8245	-0.44	0.01	0.00E+00
education	bmi	ivw	8245	-0.47	0.00	0.00E+00
education	bmi	Simple mode	8245	-0.50	0.07	5.78E-14
education	bmi	Weighted mode	8245	-0.39	0.06	2.45E-10
education	fat_mass	MR Egger	5874	0.63	0.01	0.00E+00
education	fat_mass	Weighted median	5874	0.62	0.01	0.00E+00
education	fat_mass	ivw	5874	0.65	0.00	0.00E+00
education	fat_mass	Simple mode	5874	0.80	0.06	1.61E-45
education	fat_mass	Weighted mode	5874	0.49	0.05	4.15E-24
education	fat_percentage	MR Egger	12797	-0.12	0.00	2.15E-176
education	fat_percentage	Weighted median	12797	-0.13	0.00	0.00E+00
education	fat_percentage	ivw	12797	-0.13	0.00	0.00E+00
education	fat_percentage	Simple mode	12797	-0.11	0.03	1.29E-04
education	fat_percentage	Weighted mode	12797	-0.11	0.02	3.36E-06
education	age of first sex	MR Egger	11522	0.99	0.01	0.00E+00
education	age of first sex	Weighted median	11522	1.00	0.01	0.00E+00
education	age of first sex	ivw	11522	1.05	0.01	0.00E+00

education	age of first sex	Simple mode	11522	0.97	0.10	1.32E-22
education	age of first sex	Weighted mode	11522	0.95	0.09	1.58E-26
education	smoke	MR Egger	14428	-0.25	0.01	6.33E-69
education	smoke	Weighted median	14428	-0.28	0.01	3.93E-213
education	smoke	ivw	14428	-0.29	0.01	0.00E+00
education	smoke	Simple mode	14428	-0.26	0.09	4.61E-03
education	smoke	Weighted mode	14428	-0.26	0.09	3.42E-03

supplementary table 13 Results of the mediation Mendelian randomization analysis
outcome

			Indirect action					direct action					
exposrue	outcome	medium	beta	se	p	95% IC		beta	se	p	95% IC		Proportion Mediated
eduction	LUAD	age of first birth	-0.38	2.31E-03	<0.0001	-0.38	-0.37	-0.36	0.06	3.03E-09	-0.48	-0.24	0.54
		bmi	-0.14	1.23E-03	<0.0001	-0.14	-0.14	-0.60	0.00	<0.0001	-0.60	-0.60	0.20
		fat_percentage	-0.28	6.69E-04	<0.0001	-0.28	-0.28	-0.47	0.00	<0.0001	-0.47	-0.47	0.40
		fat_mass	-0.12	7.59E-04	<0.0001	-0.13	-0.12	-0.62	0.00	<0.0001	-0.62	-0.61	0.18
		age of first sex	-0.61	3.56E-03	<0.0001	-0.61	-0.60	-0.13	0.00	<0.0001	-0.14	-0.13	0.87
		smoke	-0.85	1.77E-02	<0.0001	-0.89	-0.82	0.11	0.02	1.79E-10	0.08	0.15	1.22
	SCLC	age of first birth	-0.35	2.18E-03	<0.0001	-0.36	-0.35	-1.41	0.11	<0.0001	-1.63	-1.19	0.20
		bmi	-0.21	1.89E-03	<0.0001	-0.22	-0.21	-1.55	0.00	<0.0001	-1.56	-1.55	0.12
		fat_percentage	0.51	8.67E-04	<0.0001	0.51	0.51	-2.27	0.00	<0.0001	-2.28	-2.27	-0.29
		fat_mass	-0.19	1.14E-03	<0.0001	-0.19	-0.19	-1.58	0.00	<0.0001	-1.58	-1.58	0.11
		age of first sex	-0.75	4.40E-03	<0.0001	-0.76	-0.74	-1.01	0.00	<0.0001	-1.02	-1.01	0.43
		smoke	-0.90	1.87E-02	<0.0001	-0.94	-0.87	-0.86	0.02	<0.0001	-0.90	-0.83	0.51
	Lung cancer	age of first birth	-0.26	1.57E-03	<0.0001	-0.26	-0.25	-1.00	0.04	<0.0001	-1.09	-0.92	0.26
		bmi	-0.09	7.69E-04	<0.0001	-0.09	-0.09	0.00	0.00	3.17E-01	0.00	0.00	0.09
		fat_percentage	-0.27	4.92E-04	<0.0001	-0.28	-0.27	-0.73	0.00	<0.0001	-0.73	-0.73	0.27
		fat_mass	-0.09	5.77E-04	<0.0001	-0.10	-0.09	0.00	0.00	3.17E-01	0.00	0.00	0.09
		age of first sex	-0.47	2.78E-03	<0.0001	-0.48	-0.47	0.00	0.00	3.17E-01	-0.01	0.00	0.47
		smoke	-0.61	1.26E-02	<0.0001	-0.63	-0.58	-0.01	0.01	3.17E-01	-0.04	0.01	0.61
	LUSC	age of first birth	-0.38	2.31E-03	<0.0001	-0.38	-0.37	-0.95	0.07	<0.0001	-1.09	-0.81	0.28
		bmi	-0.14	1.23E-03	<0.0001	-0.14	-0.14	-1.19	0.00	<0.0001	-1.19	-1.19	0.10
		fat_percentage	-0.49	6.69E-04	<0.0001	-0.50	-0.49	-0.83	0.00	<0.0001	-0.83	-0.83	0.37
		fat_mass	-0.12	7.59E-04	<0.0001	-0.13	-0.12	-1.21	0.00	<0.0001	-1.21	-1.20	0.09
		age of first sex	-0.61	3.56E-03	<0.0001	-0.61	-0.60	-0.72	0.00	<0.0001	-0.73	-0.72	0.46
		smoke	-0.85	1.77E-02	<0.0001	-0.89	-0.82	-0.48	0.02	<0.0001	-0.51	-0.44	0.64

supplementary Table 14 The colocalization result between exposures and LUAD

id.exposure	SNP	pos.exposure	chr	id	snp	PPH3	PPH4	PPH5
ebi-a-GCST004441	rs71478720	112009605	11	LUAD	4217	0.11	0.65	0.86
ebi-a-GCST009971	rs11671669	41324392	19	LUAD	4410	0.00	1.00	1.00
ebi-a-GCST009971	rs11881522	41403509	19	LUAD	4335	0.00	1.00	1.00
ebi-a-GCST009971	rs148103807	40976693	19	LUAD	4531	0.00	1.00	1.00
ebi-a-GCST009971	rs56113850	41353107	19	LUAD	4352	0.00	1.00	1.00
ebi-a-GCST90000047	rs12907546	47684280	15	LUAD	5012	0.10	0.73	0.88
ebi-a-GCST90000047	rs1962545	7522336	1	LUAD	5303	0.13	0.59	0.82
ebi-a-GCST90029012	rs1461200	166918027	2	LUAD	4933	0.21	0.58	0.73
ebi-a-GCST90029012	rs4235642	103818412	5	LUAD	4847	0.08	0.65	0.89
ebi-a-GCST90029012	rs7043386	134866354	9	LUAD	4939	0.11	0.74	0.87
ebi-a-GCST90029013	rs2706762	70488470	2	LUAD	4522	0.04	0.86	0.96
ebi-a-GCST90029013	rs4246167	134933730	9	LUAD	4993	0.10	0.77	0.89
ebi-a-GCST90029014	rs10210512	199489760	2	LUAD	2927	0.10	0.80	0.89
ebi-a-GCST90029014	rs12910916	47675655	15	LUAD	4892	0.18	0.51	0.74
eqtl-a-ENSG00000041357	rs57708073	79066653	15	LUAD	5949	0.12	0.88	0.88
eqtl-a-ENSG00000041357	rs6495304	78800416	15	LUAD	5949	0.12	0.88	0.88
eqtl-a-ENSG00000041357	rs931794	78826180	15	LUAD	5949	0.12	0.88	0.88
eqtl-a-ENSG00000142233	rs60718055	49304402	19	LUAD	6194	0.02	0.98	0.98
eqtl-a-ENSG00000142233	rs681343	49206462	19	LUAD	6194	0.02	0.98	0.98
eqtl-a-ENSG00000142233	rs76576741	49270230	19	LUAD	6194	0.02	0.98	0.98
eqtl-a-ENSG00000142233	rs77623030	49298313	19	LUAD	6194	0.02	0.98	0.98
eqtl-a-ENSG00000149573	rs111778408	35572163	14	LUAD	5110	0.05	0.95	0.95
eqtl-a-ENSG00000149573	rs12419365	118112332	11	LUAD	5110	0.05	0.95	0.95
eqtl-a-ENSG00000149573	rs2156850	118030186	11	LUAD	5110	0.05	0.95	0.95
eqtl-a-ENSG00000149573	rs512849	118405343	11	LUAD	5110	0.05	0.95	0.95
eqtl-a-ENSG00000149573	rs566848	118711830	11	LUAD	5110	0.05	0.95	0.95
eqtl-a-ENSG00000160588	rs143620404	117941082	11	LUAD	5139	0.03	0.97	0.97
eqtl-a-ENSG00000160588	rs1715456	118123517	11	LUAD	5139	0.03	0.97	0.97
eqtl-a-ENSG00000160588	rs77144326	118315731	11	LUAD	5139	0.03	0.97	0.97
eqtl-a-ENSG00000188199	rs1059225	81374986	10	LUAD	6653	0.19	0.65	0.78
eqtl-a-ENSG00000188199	rs12767431	81210298	10	LUAD	6653	0.19	0.65	0.78
eqtl-a-ENSG00000188199	rs77745801	82355312	10	LUAD	6653	0.19	0.65	0.78
eqtl-a-ENSG00000188199	rs7903937	81187974	10	LUAD	6653	0.19	0.65	0.78
eqtl-a-ENSG00000188199	rs79434154	81769515	10	LUAD	6653	0.19	0.65	0.78
eqtl-a-ENSG00000259015	rs1007934	73463479	14	LUAD	5548	0.08	0.78	0.91
eqtl-a-ENSG00000259015	rs17182237	73717452	14	LUAD	5548	0.08	0.78	0.91
eqtl-a-ENSG00000259015	rs72730304	73199290	14	LUAD	5548	0.08	0.78	0.91
eqtl-a-ENSG00000259015	rs9652296	74185596	14	LUAD	5548	0.08	0.78	0.91
ieu-a-1126	rs17145151	9090705	10	LUAD	6752	0.15	0.69	0.82
ieu-a-1126	rs3769821	202123430	2	LUAD	4521	0.02	0.94	0.98
ieu-a-1239	rs2706762	70488470	2	LUAD	4485	0.07	0.78	0.91

ieu-a-1239	rs4382592	134870755	9	LUAD	4906	0.11	0.74	0.87
ieu-a-1239	rs73344830	103816828	10	LUAD	3614	0.14	0.50	0.78
ieu-a-1239	rs74998289	43913558	17	LUAD	1686	0.17	0.60	0.78
ieu-b-142	rs11671669	41324392	19	LUAD	4518	0.00	1.00	1.00
ieu-b-142	rs1579233	52074530	16	LUAD	4767	0.13	0.64	0.84
ieu-b-142	rs56113850	41353107	19	LUAD	4461	0.00	1.00	1.00
ieu-b-142	rs57708073	79066653	15	LUAD	5430	0.03	0.97	0.97
ieu-b-142	rs58379124	42579203	8	LUAD	3723	0.17	0.52	0.76
ieu-b-142	rs73229090	27442127	8	LUAD	6219	0.23	0.64	0.73
ieu-b-142	rs8034191	78806023	15	LUAD	5278	0.03	0.97	0.97
ieu-b-25	rs11671669	41324392	19	LUAD	4517	0.00	1.00	1.00
ieu-b-25	rs1579233	52074530	16	LUAD	4767	0.13	0.64	0.84
ieu-b-25	rs56113850	41353107	19	LUAD	4460	0.00	1.00	1.00
ieu-b-25	rs57708073	79066653	15	LUAD	5430	0.03	0.97	0.97
ieu-b-25	rs58379124	42579203	8	LUAD	3723	0.17	0.52	0.76
ieu-b-25	rs73229090	27442127	8	LUAD	6219	0.23	0.64	0.73
ieu-b-25	rs8034191	78806023	15	LUAD	5278	0.03	0.97	0.97
ieu-b-4879	rs111716290	105989765	10	LUAD	4234	0.02	0.98	0.98
ieu-b-4879	rs131794	50971752	22	LUAD	3652	0.03	0.89	0.97
ieu-b-4879	rs148297846	1298017	5	LUAD	6482	0.01	0.99	0.99
ieu-b-4879	rs2967355	82200103	16	LUAD	8884	0.06	0.93	0.94
ieu-b-4879	rs6536702	164028105	4	LUAD	5516	0.18	0.81	0.82
ieu-b-4879	rs7705526	1285974	5	LUAD	6450	0.01	0.99	0.99
ieu-b-4879	rs79744444	1363825	5	LUAD	6700	0.00	1.00	1.00
ieu-b-4879	rs9419958	105675946	10	LUAD	3875	0.02	0.98	0.98
prot-a-1051	rs550057	136146597	9	LUAD	6536	0.18	0.52	0.75
prot-a-1051	rs601338	49206674	19	LUAD	6192	0.02	0.98	0.98
prot-a-1051	rs78534112	136327818	9	LUAD	6572	0.18	0.52	0.74
ukb-a-205	rs2036527	78851615	15	LUAD	5645	0.04	0.96	0.96
ukb-a-237	rs10958726	42535909	8	LUAD	4064	0.18	0.51	0.74
ukb-a-237	rs2002403	78681002	15	LUAD	5422	0.04	0.96	0.96
ukb-a-237	rs8034191	78806023	15	LUAD	5639	0.04	0.96	0.96
ukb-a-238	rs10958726	42535909	8	LUAD	4064	0.18	0.51	0.74
ukb-a-238	rs2002403	78681002	15	LUAD	5422	0.02	0.98	0.98
ukb-a-238	rs4887067	78886947	15	LUAD	5666	0.02	0.98	0.98
ukb-a-28	rs1342780	91198676	1	LUAD	4877	0.16	0.60	0.79
ukb-a-328	rs184589612	41412192	19	LUAD	4620	0.00	1.00	1.00
ukb-a-328	rs56113850	41353107	19	LUAD	4667	0.00	1.00	1.00
ukb-a-328	rs58365910	78849034	15	LUAD	5643	0.04	0.96	0.96
ukb-a-328	rs62012629	79070351	15	LUAD	5809	0.04	0.96	0.96
ukb-a-342	rs11671669	41324392	19	LUAD	4724	0.00	1.00	1.00
ukb-a-342	rs56113850	41353107	19	LUAD	4667	0.00	1.00	1.00
ukb-a-342	rs6987704	42547623	8	LUAD	4051	0.18	0.51	0.74

ukb-a-342	rs8034191	78806023	15	LUAD	5639	0.05	0.95	0.95
ukb-b-14521	rs62012629	79070351	15	LUAD	5767	0.04	0.96	0.96
ukb-b-14521	rs72740955	78849779	15	LUAD	5605	0.04	0.96	0.96
ukb-b-16489	rs11685076	166925084	2	LUAD	4937	0.20	0.60	0.75
ukb-b-16489	rs4235642	103818412	5	LUAD	4820	0.09	0.60	0.87
ukb-b-469	rs11671669	41324392	19	LUAD	4729	0.00	1.00	1.00
ukb-b-469	rs56113850	41353107	19	LUAD	4670	0.00	1.00	1.00
ukb-b-469	rs6987704	42547623	8	LUAD	3832	0.17	0.52	0.75
ukb-b-6019	rs11671669	41324392	19	LUAD	4855	0.00	1.00	1.00
ukb-b-6019	rs184589612	41412192	19	LUAD	4745	0.00	1.00	1.00
ukb-b-6019	rs56113850	41353107	19	LUAD	4797	0.00	1.00	1.00
ukb-b-6019	rs6495304	78800416	15	LUAD	5676	0.04	0.96	0.96
ukb-b-6019	rs72740955	78849779	15	LUAD	5684	0.04	0.96	0.96
ukb-b-6591	rs12907546	47684280	15	LUAD	4860	0.13	0.68	0.84
ukb-b-6591	rs6692613	7524974	1	LUAD	5056	0.13	0.65	0.83
ukb-b-6591	rs7025089	134881443	9	LUAD	4955	0.09	0.77	0.89
ukb-b-7460	rs62012628	79070000	15	LUAD	5853	0.14	0.86	0.86
ukb-b-7460	rs6987704	42547623	8	LUAD	4080	0.18	0.50	0.73
ukb-b-7460	rs8042849	78817929	15	LUAD	5678	0.10	0.90	0.90

supplementary Table 15 The colocalization result between exposures and SCLC

id.exposure	SNP	pos.exposure	chr	id	snp	PPH3	PPH4	PPH5
ebi-a-GCST006368	rs10134820	30501885	14	SCLC	4518	0.09	0.72	0.89
ebi-a-GCST006368	rs7161194	101529005	14	SCLC	5078	0.13	0.57	0.81
ebi-a-GCST006368	rs12885454	29736838	14	SCLC	4805	0.11	0.71	0.86
ebi-a-GCST009971	rs11881522	41403509	19	SCLC	3848	0.09	0.71	0.89
ebi-a-GCST009971	rs11671669	41324392	19	SCLC	3949	0.09	0.70	0.88
ebi-a-GCST009971	rs149293272	41776752	19	SCLC	3578	0.08	0.71	0.90
ebi-a-GCST009971	rs56113850	41353107	19	SCLC	3854	0.09	0.71	0.89
ebi-a-GCST009971	rs7246742	41345395	19	SCLC	3894	0.09	0.71	0.89
ebi-a-GCST90000047	rs6504551	65903326	17	SCLC	4879	0.27	0.64	0.70
ebi-a-GCST90000514	rs2164300	67813017	4	SCLC	1531	0.08	0.75	0.90
ebi-a-GCST90013870	rs7161194	101529005	14	SCLC	5123	0.13	0.57	0.81
ebi-a-GCST90013922	rs6913550	26540683	6	SCLC	4759	0.26	0.71	0.73
ebi-a-GCST90013922	rs34684276	78813155	15	SCLC	5670	0.05	0.95	0.95
ebi-a-GCST90013922	rs4245739	204518842	1	SCLC	4910	0.19	0.57	0.75
ebi-a-GCST90013922	rs147560086	1054956	12	SCLC	5709	0.02	0.97	0.98
ebi-a-GCST90013972	rs6913550	26540683	6	SCLC	4759	0.26	0.71	0.73
ebi-a-GCST90013972	rs421629	1320136	5	SCLC	6656	0.19	0.54	0.74
ebi-a-GCST90013972	rs147560086	1054956	12	SCLC	5709	0.02	0.97	0.98
ebi-a-GCST90013972	rs34684276	78813155	15	SCLC	5670	0.05	0.95	0.95
ebi-a-GCST90013974	rs7161194	101529005	14	SCLC	5123	0.13	0.57	0.81
ebi-a-GCST90014021	rs3218036	30305684	19	SCLC	5046	0.15	0.63	0.81
ebi-a-GCST90018902	rs931794	78826180	15	SCLC	5384	0.04	0.95	0.96
ebi-a-GCST90018947	rs12357890	99762693	10	SCLC	4627	0.06	0.81	0.93
ebi-a-GCST90018947	rs73286281	76083337	10	SCLC	2781	0.16	0.60	0.78
ebi-a-GCST90018947	rs62086046	65982943	17	SCLC	4645	0.13	0.82	0.86
ebi-a-GCST90018947	rs62104483	30300017	19	SCLC	5953	0.17	0.61	0.78
ebi-a-GCST90018949	rs62104483	30300017	19	SCLC	5953	0.17	0.62	0.79
ebi-a-GCST90018949	rs7216064	65898809	17	SCLC	4756	0.13	0.83	0.86
ebi-a-GCST90018949	rs2439823	99778226	10	SCLC	4626	0.06	0.81	0.93
ebi-a-GCST90025994	rs8102137	30296853	19	SCLC	523	0.01	0.76	0.98
ebi-a-GCST90025994	rs1307813	29755868	14	SCLC	337	0.01	0.71	0.98
ebi-a-GCST90025994	rs8079754	65887896	17	SCLC	474	0.01	0.95	0.99
ebi-a-GCST90025994	rs11636917	63793873	15	SCLC	485	0.02	0.52	0.96
ebi-a-GCST90025994	rs11000945	76030739	10	SCLC	230	0.01	0.64	0.98
ebi-a-GCST90029007	rs113701136	30277729	19	SCLC	5911	0.16	0.62	0.79
ebi-a-GCST90029007	rs2439823	99778226	10	SCLC	4525	0.13	0.57	0.81
ebi-a-GCST90029007	rs7161194	101529005	14	SCLC	4867	0.14	0.57	0.81
ebi-a-GCST90029012	rs2860049	47515395	15	SCLC	4989	0.08	0.86	0.91
ebi-a-GCST90029012	rs1423920	63523020	16	SCLC	4731	0.15	0.51	0.78
eqtl-a-ENSG00000041357	rs931794	78826180	15	SCLC	5417	0.08	0.92	0.92
eqtl-a-ENSG00000065911	rs12621729	74482548	2	SCLC	3723	0.07	0.76	0.91

eqtl-a-ENSG00000065911	rs4853012	74361290	2	SCLC	3723	0.07	0.76	0.91
eqtl-a-ENSG00000065911	rs13411185	74065119	2	SCLC	3723	0.07	0.76	0.91
eqtl-a-ENSG00000065911	rs1667600	74402277	2	SCLC	3723	0.07	0.76	0.91
eqtl-a-ENSG00000124549	rs6916321	26464789	6	SCLC	5103	0.21	0.77	0.79
eqtl-a-ENSG00000124549	rs9295675	25918473	6	SCLC	3894	0.20	0.79	0.80
eqtl-a-ENSG00000137218	rs12529962	41623300	6	SCLC	4604	0.02	0.97	0.98
eqtl-a-ENSG00000137218	rs12526550	41970404	6	SCLC	4604	0.02	0.97	0.98
eqtl-a-ENSG00000137218	rs1057227	41738158	6	SCLC	4604	0.02	0.97	0.98
eqtl-a-ENSG00000137218	rs114071735	41627043	6	SCLC	4604	0.02	0.97	0.98
eqtl-a-ENSG00000145416	rs76109254	164137902	4	SCLC	6011	0.07	0.92	0.93
eqtl-a-ENSG00000145416	rs36091410	164328085	4	SCLC	6011	0.07	0.92	0.93
eqtl-a-ENSG00000145416	rs75403003	164571263	4	SCLC	6011	0.07	0.92	0.93
eqtl-a-ENSG00000145416	rs78984977	164478104	4	SCLC	6011	0.07	0.92	0.93
eqtl-a-ENSG00000145416	rs7689631	164412382	4	SCLC	6011	0.07	0.92	0.93
eqtl-a-ENSG00000213694	rs71510292	91898790	9	SCLC	4906	0.04	0.91	0.96
eqtl-a-ENSG00000213694	rs12478601	43721508	9	SCLC	4906	0.04	0.91	0.96
eqtl-a-ENSG00000213694	rs10780209	91472127	9	SCLC	4906	0.04	0.91	0.96
eqtl-a-ENSG00000213694	rs35979828	54685880	9	SCLC	4906	0.04	0.91	0.96
eqtl-a-ENSG00000213694	rs7858626	91612639	9	SCLC	4906	0.04	0.91	0.96
ieu-a-1001	rs1378214	47579004	15	SCLC	4699	0.18	0.70	0.80
ieu-a-1239	rs13141210	67891641	4	SCLC	5199	0.12	0.80	0.87
ieu-a-1239	rs6493265	47513253	15	SCLC	4954	0.09	0.85	0.91
ieu-a-835	rs11847697	30515112	14	SCLC	1476	0.03	0.77	0.96
ieu-a-835	rs12885454	29736838	14	SCLC	1497	0.04	0.76	0.95
ieu-b-142	rs8034191	78806023	15	SCLC	4847	0.03	0.97	0.97
ieu-b-142	rs56113850	41353107	19	SCLC	3910	0.09	0.71	0.89
ieu-b-142	rs13141210	67891641	4	SCLC	4896	0.21	0.61	0.74
ieu-b-25	rs13141210	67891641	4	SCLC	4896	0.21	0.61	0.74
ieu-b-25	rs8034191	78806023	15	SCLC	4847	0.03	0.97	0.97
ieu-b-25	rs56113850	41353107	19	SCLC	3910	0.09	0.71	0.89
ieu-b-40	rs17513613	30286822	19	SCLC	1756	0.05	0.69	0.93
ieu-b-40	rs12602912	65870073	17	SCLC	1147	0.03	0.93	0.97
ieu-b-40	rs577525	99769388	10	SCLC	1499	0.05	0.65	0.93
ieu-b-40	rs10483389	30495719	14	SCLC	1295	0.03	0.77	0.97
ieu-b-40	rs12885454	29736838	14	SCLC	1345	0.04	0.76	0.95
ieu-b-40	rs4880341	133992689	10	SCLC	1471	0.08	0.52	0.86
ieu-b-5118	rs62107111	30285548	19	SCLC	5641	0.16	0.61	0.79
ieu-b-5118	rs11000993	76084111	10	SCLC	2644	0.14	0.64	0.82
ieu-b-5118	rs11000993	76084111	10	SCLC	2644	0.15	0.64	0.81
ieu-b-5118	rs7161194	101529005	14	SCLC	4640	0.13	0.57	0.81
ieu-b-5118	rs12357890	99762693	10	SCLC	4404	0.12	0.62	0.84
ieu-b-5118	rs55931203	65854602	17	SCLC	4593	0.13	0.82	0.86
ukb-a-237	rs2316205	41346768	19	SCLC	4126	0.14	0.56	0.80

ukb-a-237	rs739447	136477511	9	SCLC	5778	0.20	0.51	0.71
ukb-a-237	rs2002403	78681002	15	SCLC	5059	0.05	0.95	0.95
ukb-a-237	rs8034191	78806023	15	SCLC	5208	0.05	0.95	0.95
ukb-a-238	rs2316205	41346768	19	SCLC	4126	0.14	0.57	0.81
ukb-a-238	rs739447	136477511	9	SCLC	5778	0.17	0.59	0.78
ukb-a-238	rs4887067	78886947	15	SCLC	5292	0.04	0.96	0.96
ukb-a-248	rs2439823	99778226	10	SCLC	4508	0.12	0.62	0.84
ukb-a-248	rs7161194	101529005	14	SCLC	4818	0.15	0.52	0.77
ukb-a-249	rs58551145	65837235	17	SCLC	4771	0.13	0.83	0.87
ukb-a-249	rs2439823	99778226	10	SCLC	4508	0.12	0.63	0.85
ukb-a-264	rs62104477	30294991	19	SCLC	5825	0.17	0.62	0.79
ukb-a-264	rs2439823	99778226	10	SCLC	4508	0.12	0.61	0.84
ukb-a-264	rs80135947	65836001	17	SCLC	4769	0.13	0.83	0.87
ukb-a-265	rs2439823	99778226	10	SCLC	4508	0.12	0.62	0.84
ukb-a-265	rs80135947	65836001	17	SCLC	4769	0.13	0.83	0.87
ukb-a-274	rs2439823	99778226	10	SCLC	4508	0.13	0.57	0.81
ukb-a-274	rs8073510	65839210	17	SCLC	4769	0.13	0.83	0.86
ukb-a-274	rs62104477	30294991	19	SCLC	5825	0.18	0.59	0.77
ukb-a-275	rs80135947	65836001	17	SCLC	4769	0.13	0.83	0.87
ukb-a-275	rs62104477	30294991	19	SCLC	5825	0.17	0.61	0.79
ukb-a-275	rs2439823	99778226	10	SCLC	4508	0.12	0.62	0.84
ukb-a-278	rs2439823	99778226	10	SCLC	4508	0.13	0.60	0.83
ukb-a-278	rs17513752	30290811	19	SCLC	5836	0.17	0.60	0.78
ukb-a-279	rs2439823	99778226	10	SCLC	4508	0.12	0.63	0.84
ukb-a-282	rs80135947	65836001	17	SCLC	4769	0.13	0.83	0.87
ukb-a-282	rs2439823	99778226	10	SCLC	4508	0.12	0.61	0.83
ukb-a-282	rs3218036	30305684	19	SCLC	5810	0.17	0.62	0.79
ukb-a-283	rs2439823	99778226	10	SCLC	4508	0.12	0.62	0.84
ukb-a-283	rs62104483	30300017	19	SCLC	5833	0.17	0.61	0.79
ukb-a-283	rs16895130	41924931	6	SCLC	4986	0.19	0.69	0.79
ukb-a-286	rs2439823	99778226	10	SCLC	4508	0.12	0.61	0.84
ukb-a-286	rs62104483	30300017	19	SCLC	5833	0.16	0.62	0.79
ukb-a-286	rs80135947	65836001	17	SCLC	4769	0.13	0.83	0.87
ukb-a-287	rs62104483	30300017	19	SCLC	5833	0.17	0.62	0.79
ukb-a-287	rs16895130	41924931	6	SCLC	4986	0.18	0.70	0.79
ukb-a-287	rs2439823	99778226	10	SCLC	4508	0.12	0.62	0.84
ukb-a-290	rs577525	99769388	10	SCLC	4508	0.12	0.61	0.83
ukb-a-290	rs3218036	30305684	19	SCLC	5810	0.16	0.62	0.79
ukb-a-290	rs80135947	65836001	17	SCLC	4769	0.13	0.83	0.87
ukb-a-291	rs2439823	99778226	10	SCLC	4508	0.12	0.62	0.84
ukb-a-291	rs62104483	30300017	19	SCLC	5833	0.17	0.62	0.79
ukb-a-328	rs56113850	41353107	19	SCLC	4096	0.09	0.71	0.88
ukb-a-328	rs62012629	79070351	15	SCLC	5470	0.02	0.98	0.98

ukb-a-328	rs2002403	78681002	15	SCLC	5059	0.02	0.98	0.98
ukb-a-328	rs58365910	78849034	15	SCLC	5203	0.02	0.98	0.98
ukb-a-343	rs8034191	78806023	15	SCLC	5208	0.06	0.94	0.94
ukb-a-343	rs7867984	136485598	9	SCLC	5787	0.02	0.95	0.98
ukb-a-382	rs2439823	99778226	10	SCLC	4508	0.12	0.62	0.84
ukb-a-382	rs80135947	65836001	17	SCLC	4769	0.13	0.83	0.87
ukb-a-397	rs2860049	47515395	15	SCLC	4940	0.08	0.87	0.92
ukb-a-399	rs1424532	186178814	2	SCLC	4490	0.15	0.57	0.79
ukb-b-10831	rs2316205	41346768	19	SCLC	4248	0.13	0.59	0.82
ukb-b-10831	rs8042849	78817929	15	SCLC	5230	0.17	0.83	0.83
ukb-b-11842	rs2439823	99778226	10	SCLC	4518	0.12	0.63	0.85
ukb-b-11842	rs12462975	30272202	19	SCLC	5910	0.17	0.61	0.78
ukb-b-12039	rs2439823	99778226	10	SCLC	4518	0.11	0.63	0.85
ukb-b-12854	rs12462975	30272202	19	SCLC	5910	0.17	0.62	0.79
ukb-b-12854	rs7161194	101529005	14	SCLC	4861	0.14	0.57	0.81
ukb-b-12854	rs2439823	99778226	10	SCLC	4518	0.12	0.62	0.84
ukb-b-16489	rs6493265	47513253	15	SCLC	4983	0.08	0.86	0.91
ukb-b-16489	rs1423920	63523020	16	SCLC	4720	0.15	0.50	0.77
ukb-b-17729	rs13141210	67891641	4	SCLC	5229	0.13	0.78	0.86
ukb-b-18096	rs17513752	30290811	19	SCLC	5863	0.17	0.61	0.78
ukb-b-18096	rs577525	99769388	10	SCLC	4518	0.12	0.61	0.83
ukb-b-18096	rs7161194	101529005	14	SCLC	4861	0.14	0.56	0.80
ukb-b-18377	rs577525	99769388	10	SCLC	4518	0.13	0.59	0.82
ukb-b-19393	rs10141106	59348019	14	SCLC	5178	0.16	0.56	0.77
ukb-b-19393	rs12462975	30272202	19	SCLC	5910	0.17	0.62	0.79
ukb-b-19393	rs577525	99769388	10	SCLC	4518	0.12	0.61	0.84
ukb-b-19953	rs10824211	76363107	10	SCLC	2870	0.17	0.58	0.77
ukb-b-19953	rs7161194	101529005	14	SCLC	4861	0.14	0.57	0.81
ukb-b-19953	rs12462975	30272202	19	SCLC	5910	0.17	0.62	0.78
ukb-b-19953	rs2439823	99778226	10	SCLC	4518	0.12	0.61	0.83
ukb-b-20044	rs12462975	30272202	19	SCLC	5910	0.17	0.62	0.79
ukb-b-20188	rs522110	99772885	10	SCLC	4519	0.12	0.62	0.84
ukb-b-20188	rs7161194	101529005	14	SCLC	4861	0.14	0.56	0.80
ukb-b-20188	rs12462975	30272202	19	SCLC	5910	0.17	0.62	0.79
ukb-b-20531	rs577525	99769388	10	SCLC	4518	0.14	0.56	0.81
ukb-b-20531	rs7161194	101529005	14	SCLC	4861	0.14	0.57	0.81
ukb-b-2303	rs12462975	30272202	19	SCLC	5910	0.17	0.62	0.79
ukb-b-2303	rs11000993	76084111	10	SCLC	2721	0.18	0.55	0.75
ukb-b-2303	rs7161194	101529005	14	SCLC	4861	0.14	0.57	0.81
ukb-b-2303	rs577525	99769388	10	SCLC	4518	0.12	0.61	0.83
ukb-b-2732	rs7867984	136485598	9	SCLC	5682	0.03	0.94	0.97
ukb-b-2732	rs8034191	78806023	15	SCLC	5117	0.08	0.92	0.92
ukb-b-6019	rs2002403	78681002	15	SCLC	5087	0.02	0.98	0.98

ukb-b-6019	rs56113850	41353107	19	SCLC	4216	0.10	0.70	0.88
ukb-b-6019	rs11671669	41324392	19	SCLC	4311	0.10	0.70	0.87
ukb-b-6019	rs72740955	78849779	15	SCLC	5223	0.02	0.98	0.98
ukb-b-6591	rs993700	67825894	4	SCLC	5187	0.23	0.60	0.73
ukb-b-6704	rs12462975	30272202	19	SCLC	5910	0.17	0.61	0.78
ukb-b-6704	rs2439823	99778226	10	SCLC	4518	0.12	0.62	0.84
ukb-b-6704	rs7161194	101529005	14	SCLC	4861	0.14	0.56	0.80
ukb-b-7212	rs7161194	101529005	14	SCLC	4861	0.15	0.54	0.79
ukb-b-7212	rs62104473	30289779	19	SCLC	5866	0.17	0.61	0.78
ukb-b-7212	rs577525	99769388	10	SCLC	4518	0.12	0.62	0.84
ukb-b-7460	rs3025360	136481205	9	SCLC	5829	0.20	0.53	0.73
ukb-b-7460	rs2316205	41346768	19	SCLC	4248	0.12	0.62	0.83
ukb-b-7460	rs8042849	78817929	15	SCLC	5230	0.04	0.96	0.96
ukb-b-8338	rs12462975	30272202	19	SCLC	5910	0.17	0.62	0.79
ukb-b-8338	rs577525	99769388	10	SCLC	4518	0.12	0.62	0.84
ukb-b-8338	rs7161194	101529005	14	SCLC	4861	0.15	0.51	0.77
ukb-b-8338	rs13340461	41924278	6	SCLC	5041	0.21	0.66	0.76
ukb-b-8909	rs12462975	30272202	19	SCLC	5910	0.17	0.62	0.79
ukb-b-8909	rs577525	99769388	10	SCLC	4518	0.12	0.61	0.83
ukb-b-9405	rs12462975	30272202	19	SCLC	5910	0.17	0.62	0.79
ukb-b-9405	rs2439823	99778226	10	SCLC	4518	0.12	0.62	0.84
ukb-d-30710_raw	rs11230768	61430471	11	SCLC	4263	0.12	0.60	0.83
ukb-d-30710_raw	rs1037170	72702914	17	SCLC	5546	0.09	0.74	0.89

supplementary Table 16 The colocalization result between exposures and LC

id.exposure	SNP	pos.exposure	chr	id	snp	PPH3	PPH4	PPH5
ebi-a-GCST004622	rs2263316	31421297	6	Lung cancer	3165	0.03	0.97	0.97
ebi-a-GCST004622	rs2523611	31321464	6	Lung cancer	3138	0.03	0.97	0.97
ebi-a-GCST006250	rs11634187	40722781	15	Lung cancer	4040	0.08	0.84	0.91
ebi-a-GCST006250	rs1455344	199516059	2	Lung cancer	2773	0.13	0.73	0.85
ebi-a-GCST006250	rs7248006	31929180	19	Lung cancer	4198	0.16	0.73	0.82
ebi-a-GCST006250	rs1007934	73463479	14	Lung cancer	5408	0.12	0.52	0.81
ebi-a-GCST009971	rs11881522	41403509	19	Lung cancer	4178	0.00	1.00	1.00
ebi-a-GCST009971	rs11671669	41324392	19	Lung cancer	4251	0.00	1.00	1.00
ebi-a-GCST009971	rs148103807	40976693	19	Lung cancer	4337	0.00	1.00	1.00
ebi-a-GCST009971	rs56113850	41353107	19	Lung cancer	4193	0.00	1.00	1.00
ebi-a-GCST009971	rs7246742	41345395	19	Lung cancer	4234	0.00	1.00	1.00
ebi-a-GCST90000047	rs1962545	7522336	1	Lung cancer	5374	0.11	0.52	0.83
ebi-a-GCST90000047	rs34517439	78450517	1	Lung cancer	5277	0.07	0.93	0.93
ebi-a-GCST90000048	rs329122	133864599	5	Lung cancer	4246	0.01	0.98	0.99
ebi-a-GCST90000048	rs1702877	56427808	12	Lung cancer	3868	0.12	0.60	0.83
ebi-a-GCST90000050	rs17391694	78623626	1	Lung cancer	5384	0.16	0.84	0.84
ebi-a-GCST90000050	rs1702877	56427808	12	Lung cancer	3854	0.13	0.57	0.81
ebi-a-GCST90000514	rs4382592	134870755	9	Lung cancer	1572	0.05	0.61	0.92
ebi-a-GCST90000514	rs2782641	44013355	1	Lung cancer	1298	0.06	0.65	0.92
ebi-a-GCST90000514	rs7032155	122672771	9	Lung cancer	1645	0.11	0.51	0.82
ebi-a-GCST90000514	rs215614	32347335	7	Lung cancer	2000	0.07	0.67	0.91
ebi-a-GCST90000514	rs773109	56374695	12	Lung cancer	982	0.03	0.77	0.97
ebi-a-GCST90000514	rs329122	133864599	5	Lung cancer	1355	0.00	0.97	1.00
ebi-a-GCST90000514	rs6711584	104421692	2	Lung cancer	1417	0.03	0.87	0.96
ebi-a-GCST90012794	rs6871635	133830395	5	Lung cancer	4260	0.05	0.85	0.95
ebi-a-GCST90012794	rs6723226	32849207	2	Lung cancer	5940	0.10	0.82	0.89
ebi-a-GCST90013870	rs215634	32369148	7	Lung cancer	6494	0.16	0.56	0.78
ebi-a-GCST90013870	rs329118	133861663	5	Lung cancer	6481	0.16	0.56	0.78
ebi-a-GCST90013870	rs748761	108453086	12	Lung cancer	5560	0.16	0.64	0.79
ebi-a-GCST90013870	rs34517439	78450517	1	Lung cancer	5210	0.01	0.99	0.99
ebi-a-GCST90013870	rs6707445	104420858	2	Lung cancer	3394	0.09	0.53	0.85
ebi-a-GCST90013922	rs57708073	79066653	15	Lung cancer	5922	0.07	0.93	0.93
ebi-a-GCST90013922	rs2853677	1287194	5	Lung cancer	7449	0.02	0.98	0.98
ebi-a-GCST90013922	rs421629	1320136	5	Lung cancer	7498	0.02	0.98	0.98
ebi-a-GCST90013972	rs57708073	79066653	15	Lung cancer	5922	0.07	0.93	0.93
ebi-a-GCST90013972	rs34684276	78813155	15	Lung cancer	6343	0.07	0.93	0.93
ebi-a-GCST90013974	rs329118	133861663	5	Lung cancer	4307	0.01	0.98	0.99
ebi-a-GCST90013974	rs35107973	143125948	4	Lung cancer	5002	0.11	0.60	0.84
ebi-a-GCST90013974	rs34517439	78450517	1	Lung cancer	5210	0.01	0.99	0.99
ebi-a-GCST90013974	rs215634	32369148	7	Lung cancer	6494	0.16	0.56	0.78
ebi-a-GCST90013974	rs748761	108453086	12	Lung cancer	5560	0.16	0.64	0.79

ebi-a-GCST90013975	rs215669	32378979	7	Lung cancer	6490	0.18	0.51	0.74
ebi-a-GCST90013975	rs10946808	26233387	6	Lung cancer	5320	0.17	0.83	0.83
ebi-a-GCST90014020	rs3212038	104178186	14	Lung cancer	6098	0.14	0.61	0.82
ebi-a-GCST90014020	rs6711584	104421692	2	Lung cancer	4481	0.06	0.85	0.93
ebi-a-GCST90014020	rs34517439	78450517	1	Lung cancer	5210	0.00	1.00	1.00
ebi-a-GCST90014021	rs28667353	32619128	6	Lung cancer	5046	0.15	0.63	0.81
ebi-a-GCST90018947	rs215669	32378979	7	Lung cancer	6469	0.14	0.60	0.81
ebi-a-GCST90018947	rs3935101	134878437	9	Lung cancer	5046	0.12	0.57	0.83
ebi-a-GCST90018947	rs329124	133865452	5	Lung cancer	4126	0.01	0.98	0.99
ebi-a-GCST90018947	rs36172	134651283	3	Lung cancer	4777	0.21	0.56	0.72
ebi-a-GCST90018947	rs72820274	104412924	2	Lung cancer	3991	0.12	0.72	0.86
ebi-a-GCST90018947	rs73193736	108294381	12	Lung cancer	5529	0.18	0.60	0.77
ebi-a-GCST90018947	rs34517439	78450517	1	Lung cancer	5110	0.01	0.99	0.99
ebi-a-GCST90018949	rs34517439	78450517	1	Lung cancer	5110	0.01	0.99	0.99
ebi-a-GCST90018949	rs329118	133861663	5	Lung cancer	4136	0.01	0.98	0.99
ebi-a-GCST90025972	rs61318425	109748506	6	Lung cancer	520	0.02	0.94	0.98
ebi-a-GCST90025972	rs10760112	123467570	9	Lung cancer	457	0.06	0.54	0.89
ebi-a-GCST90025972	rs35756394	72419796	8	Lung cancer	443	0.03	0.71	0.95
ebi-a-GCST90025994	rs1229984	100239319	4	Lung cancer	490	0.19	0.50	0.72
ebi-a-GCST90025994	rs1007934	73463479	14	Lung cancer	599	0.02	0.50	0.97
ebi-a-GCST90025994	rs749953	108422857	12	Lung cancer	461	0.01	0.78	0.99
ebi-a-GCST90025994	rs6443750	181329682	3	Lung cancer	334	0.01	0.53	0.99
ebi-a-GCST90025994	rs7043386	134866354	9	Lung cancer	673	0.01	0.66	0.99
ebi-a-GCST90025994	rs2838818	46641017	21	Lung cancer	1023	0.02	0.63	0.97
ebi-a-GCST90025994	rs12616219	104352495	2	Lung cancer	330	0.02	0.52	0.96
ebi-a-GCST90025994	rs17391694	78623626	1	Lung cancer	361	0.00	1.00	1.00
ebi-a-GCST90025994	rs215614	32347335	7	Lung cancer	574	0.01	0.79	0.99
ebi-a-GCST90025994	rs329118	133861663	5	Lung cancer	418	0.00	0.98	1.00
ebi-a-GCST90025994	rs35390852	143067054	4	Lung cancer	293	0.01	0.73	0.99
ebi-a-GCST90029007	rs2466103	32412304	8	Lung cancer	5038	0.24	0.63	0.72
ebi-a-GCST90029007	rs215634	32369148	7	Lung cancer	6140	0.17	0.51	0.75
ebi-a-GCST90029007	rs10861861	108436396	12	Lung cancer	5450	0.15	0.64	0.81
ebi-a-GCST90029007	rs329124	133865452	5	Lung cancer	4103	0.01	0.98	0.99
ebi-a-GCST90029007	rs4601460	134849254	9	Lung cancer	4917	0.12	0.59	0.84
ebi-a-GCST90029007	rs34517439	78450517	1	Lung cancer	5055	0.01	0.99	0.99
ebi-a-GCST90029012	rs7043386	134866354	9	Lung cancer	4928	0.12	0.55	0.82
ebi-a-GCST90029012	rs2860049	47515395	15	Lung cancer	5143	0.07	0.93	0.93
ebi-a-GCST90029012	rs12448821	24703630	16	Lung cancer	5714	0.12	0.54	0.82
ebi-a-GCST90029012	rs1254989	95508121	11	Lung cancer	4820	0.20	0.66	0.77
ebi-a-GCST90029012	rs806789	26229161	6	Lung cancer	5288	0.06	0.94	0.94
ebi-a-GCST90029012	rs772921	56403577	12	Lung cancer	3563	0.12	0.59	0.83
ebi-a-GCST90029013	rs4246167	134933730	9	Lung cancer	4959	0.11	0.60	0.84
ebi-a-GCST90029013	rs6935954	26255451	6	Lung cancer	5248	0.21	0.79	0.79

ebi-a-GCST90029013	rs10765775	95656362	11	Lung cancer	4963	0.13	0.77	0.85
ebi-a-GCST90029014	rs10210512	199489760	2	Lung cancer	2979	0.18	0.66	0.79
ebi-a-GCST90029014	rs329120	133861756	5	Lung cancer	4113	0.01	0.98	0.99
ebi-a-GCST90029025	rs7766641	26184102	6	Lung cancer	5266	0.12	0.88	0.88
ebi-a-GCST90029025	rs34517439	78450517	1	Lung cancer	5055	0.01	0.99	0.99
ebi-a-GCST90060294	rs174570	61597212	11	Lung cancer	410	0.01	0.90	0.99
ebi-a-GCST90096909	rs114328297	91190854	1	Lung cancer	3426	0.16	0.80	0.84
eqtl-a-ENSG00000026297	rs118180337	167310401	6	Lung cancer	5724	0.17	0.83	0.83
eqtl-a-ENSG00000026297	rs2072640	167188463	6	Lung cancer	5724	0.17	0.83	0.83
eqtl-a-ENSG00000026297	rs57237533	167364197	6	Lung cancer	5724	0.17	0.83	0.83
eqtl-a-ENSG00000026297	rs62436418	167345772	6	Lung cancer	5724	0.17	0.83	0.83
eqtl-a-ENSG00000026297	rs6931756	167622389	6	Lung cancer	5724	0.17	0.83	0.83
eqtl-a-ENSG00000095261	rs116930913	123713832	9	Lung cancer	3864	0.14	0.78	0.85
eqtl-a-ENSG00000095261	rs1060817	123583192	9	Lung cancer	3864	0.14	0.78	0.85
eqtl-a-ENSG00000103160	rs149935628	84065833	16	Lung cancer	10111	0.18	0.53	0.74
eqtl-a-ENSG00000103160	rs34915457	84098815	16	Lung cancer	10111	0.18	0.53	0.74
eqtl-a-ENSG00000103160	rs4150166	84213728	16	Lung cancer	10111	0.18	0.53	0.74
eqtl-a-ENSG00000103160	rs80139892	84141147	16	Lung cancer	10111	0.18	0.53	0.74
eqtl-a-ENSG00000103160	rs8051893	84340877	16	Lung cancer	10111	0.18	0.53	0.74
eqtl-a-ENSG00000106009	rs13243437	2596579	7	Lung cancer	7747	0.05	0.87	0.94
eqtl-a-ENSG00000106009	rs60038222	2645425	7	Lung cancer	7747	0.05	0.87	0.94
eqtl-a-ENSG00000106009	rs149007767	50370254	7	Lung cancer	7747	0.05	0.87	0.94
eqtl-a-ENSG00000106009	rs148044391	2586106	7	Lung cancer	7747	0.05	0.87	0.94
eqtl-a-ENSG00000107890	rs73598041	27475275	10	Lung cancer	6470	0.15	0.77	0.84
eqtl-a-ENSG00000107890	rs10829226	27573952	10	Lung cancer	6470	0.15	0.77	0.84
eqtl-a-ENSG00000107890	rs144610740	26975803	10	Lung cancer	6470	0.15	0.77	0.84
eqtl-a-ENSG00000107890	rs77728719	28235976	10	Lung cancer	6470	0.15	0.77	0.84
eqtl-a-ENSG00000107890	rs118183234	26965158	10	Lung cancer	6470	0.15	0.77	0.84
eqtl-a-ENSG00000149573	rs12419365	118112332	11	Lung cancer	5187	0.05	0.95	0.95
eqtl-a-ENSG00000149573	rs2156850	118030186	11	Lung cancer	5187	0.05	0.95	0.95
eqtl-a-ENSG00000149573	rs45578540	118478681	11	Lung cancer	5187	0.05	0.95	0.95
eqtl-a-ENSG00000149573	rs512849	118405343	11	Lung cancer	5187	0.05	0.95	0.95
eqtl-a-ENSG00000149573	rs73020226	118322924	11	Lung cancer	5187	0.05	0.95	0.95
eqtl-a-ENSG00000156414	rs11160739	103719055	14	Lung cancer	6134	0.08	0.77	0.91
eqtl-a-ENSG00000156414	rs138747322	104673950	14	Lung cancer	6134	0.08	0.77	0.91
eqtl-a-ENSG00000156414	rs1886459	103683407	14	Lung cancer	6134	0.08	0.77	0.91
eqtl-a-ENSG00000156414	rs34557614	104339548	14	Lung cancer	6134	0.08	0.77	0.91
eqtl-a-ENSG00000158406	rs113282904	25986650	6	Lung cancer	4762	0.08	0.92	0.92
eqtl-a-ENSG00000158406	rs1543681	26187783	6	Lung cancer	4762	0.08	0.92	0.92
eqtl-a-ENSG00000158406	rs34661691	26173478	6	Lung cancer	4762	0.08	0.92	0.92
eqtl-a-ENSG00000158406	rs6911765	26464642	6	Lung cancer	4762	0.08	0.92	0.92
eqtl-a-ENSG00000166037	rs12271427	95528891	11	Lung cancer	4936	0.13	0.78	0.86
eqtl-a-ENSG00000166037	rs139579105	95474631	11	Lung cancer	4936	0.13	0.78	0.86

eqtl-a-ENSG00000166037	rs543530	95545170	11	Lung cancer	4936	0.13	0.78	0.86
eqtl-a-ENSG00000166037	rs680664	95717152	11	Lung cancer	4936	0.13	0.78	0.86
eqtl-a-ENSG00000169045	rs10078712	178978805	5	Lung cancer	7339	0.02	0.97	0.98
eqtl-a-ENSG00000169045	rs183418222	179523214	5	Lung cancer	7339	0.02	0.97	0.98
eqtl-a-ENSG00000169045	rs28713706	178949669	5	Lung cancer	7339	0.02	0.97	0.98
eqtl-a-ENSG00000169045	rs73809239	178927757	5	Lung cancer	7339	0.02	0.97	0.98
eqtl-a-ENSG00000169045	rs77281718	179459933	5	Lung cancer	7339	0.02	0.97	0.98
eqtl-a-ENSG00000175164	rs10793956	136079542	9	Lung cancer	6165	0.15	0.61	0.80
eqtl-a-ENSG00000175164	rs118077853	136387799	9	Lung cancer	6165	0.15	0.61	0.80
eqtl-a-ENSG00000175164	rs75179845	136132954	9	Lung cancer	6165	0.15	0.61	0.80
eqtl-a-ENSG00000175164	rs78534112	136327818	9	Lung cancer	6165	0.15	0.61	0.80
eqtl-a-ENSG00000175164	rs9411378	136145425	9	Lung cancer	6165	0.15	0.61	0.80
eqtl-a-ENSG00000177706	rs11984258	41421	7	Lung cancer	3587	0.06	0.60	0.90
eqtl-a-ENSG00000177706	rs12540534	197913	7	Lung cancer	3587	0.06	0.60	0.90
eqtl-a-ENSG00000177706	rs13308834	82204	7	Lung cancer	3587	0.06	0.60	0.90
eqtl-a-ENSG00000177706	rs76029673	161970	7	Lung cancer	3587	0.06	0.60	0.90
eqtl-a-ENSG00000227598	rs1044059	167369897	6	Lung cancer	5547	0.12	0.88	0.88
eqtl-a-ENSG00000227598	rs12153952	167713949	6	Lung cancer	5547	0.12	0.88	0.88
eqtl-a-ENSG00000227598	rs61592390	167559256	6	Lung cancer	5547	0.12	0.88	0.88
eqtl-a-ENSG00000227598	rs62438721	167186776	6	Lung cancer	5547	0.12	0.88	0.88
eqtl-a-ENSG00000227598	rs73787928	167520375	6	Lung cancer	5547	0.12	0.88	0.88
eqtl-a-ENSG00000239415	rs2839154	47637760	21	Lung cancer	4407	0.12	0.61	0.83
eqtl-a-ENSG00000239415	rs6518272	47528910	21	Lung cancer	4407	0.12	0.61	0.83
eqtl-a-ENSG00000239415	rs7277463	47900194	21	Lung cancer	4407	0.12	0.61	0.83
eqtl-a-ENSG00000239415	rs8127941	47987547	21	Lung cancer	4407	0.12	0.61	0.83
eqtl-a-ENSG00000259015	rs72730304	73199290	14	Lung cancer	5662	0.10	0.59	0.85
eqtl-a-ENSG00000259015	rs17182237	73717452	14	Lung cancer	5662	0.10	0.59	0.85
eqtl-a-ENSG00000259015	rs9652296	74185596	14	Lung cancer	5662	0.10	0.59	0.85
eqtl-a-ENSG00000259015	rs1007934	73463479	14	Lung cancer	5662	0.10	0.59	0.85
finn-b-D3_ANAEMIA_B12_DEF	rs1990760	163124051	2	Lung cancer	3868	0.06	0.62	0.91
ieu-a-1001	rs12410444	44188719	1	Lung cancer	4287	0.17	0.59	0.78
ieu-a-1001	rs523934	95641775	11	Lung cancer	4892	0.13	0.77	0.85
ieu-a-1001	rs56236451	237056840	2	Lung cancer	5630	0.11	0.64	0.86
ieu-a-1001	rs1378214	47579004	15	Lung cancer	4806	0.03	0.97	0.97
ieu-a-1001	rs2456973	56416928	12	Lung cancer	3773	0.13	0.56	0.81
ieu-a-1239	rs4382592	134870755	9	Lung cancer	4891	0.13	0.55	0.81
ieu-a-1239	rs2819336	44015809	1	Lung cancer	4231	0.17	0.53	0.76
ieu-a-1239	rs10765775	95656362	11	Lung cancer	4924	0.12	0.80	0.87
ieu-a-1239	rs13141210	67891641	4	Lung cancer	5225	0.15	0.61	0.80
ieu-a-1239	rs6871635	133830395	5	Lung cancer	4070	0.12	0.63	0.84
ieu-a-1239	rs6493265	47513253	15	Lung cancer	5094	0.07	0.93	0.93
ieu-b-142	rs57708073	79066653	15	Lung cancer	5511	0.02	0.98	0.98

ieu-b-142	rs56116178	136460224	9	Lung cancer	5640	0.02	0.95	0.98
ieu-b-142	rs8034191	78806023	15	Lung cancer	5100	0.02	0.98	0.98
ieu-b-142	rs79409323	79164636	15	Lung cancer	5482	0.02	0.98	0.98
ieu-b-142	rs806798	26214473	6	Lung cancer	4915	0.14	0.86	0.86
ieu-b-142	rs11671669	41324392	19	Lung cancer	4384	0.00	1.00	1.00
ieu-b-142	rs56113850	41353107	19	Lung cancer	4330	0.00	1.00	1.00
ieu-b-142	rs1579233	52074530	16	Lung cancer	4787	0.10	0.72	0.88
ieu-b-142	rs3025383	136502369	9	Lung cancer	5664	0.02	0.95	0.98
ieu-b-142	rs2273500	61986949	20	Lung cancer	5600	0.08	0.92	0.92
ieu-b-142	rs73229090	27442127	8	Lung cancer	6182	0.25	0.75	0.75
ieu-b-25	rs57708073	79066653	15	Lung cancer	5511	0.02	0.98	0.98
ieu-b-25	rs8034191	78806023	15	Lung cancer	5100	0.02	0.98	0.98
ieu-b-25	rs1579233	52074530	16	Lung cancer	4787	0.10	0.72	0.88
ieu-b-25	rs806798	26214473	6	Lung cancer	4910	0.14	0.86	0.86
ieu-b-25	rs56113850	41353107	19	Lung cancer	4329	0.00	1.00	1.00
ieu-b-25	rs3025383	136502369	9	Lung cancer	5660	0.02	0.95	0.98
ieu-b-25	rs79409323	79164636	15	Lung cancer	5482	0.02	0.98	0.98
ieu-b-25	rs56116178	136460224	9	Lung cancer	5636	0.02	0.95	0.98
ieu-b-25	rs2273500	61986949	20	Lung cancer	5600	0.08	0.92	0.92
ieu-b-25	rs73229090	27442127	8	Lung cancer	6182	0.25	0.75	0.75
ieu-b-25	rs11671669	41324392	19	Lung cancer	4383	0.00	1.00	1.00
ieu-b-40	rs17391694	78623626	1	Lung cancer	1514	0.00	1.00	1.00
ieu-b-40	rs329122	133864599	5	Lung cancer	1287	0.00	0.98	1.00
ieu-b-4879	rs6536702	164028105	4	Lung cancer	5574	0.22	0.66	0.75
ieu-b-4879	rs9419958	105675946	10	Lung cancer	3991	0.09	0.87	0.90
ieu-b-4879	rs2967355	82200103	16	Lung cancer	9057	0.09	0.83	0.90
ieu-b-5117	rs35390852	143067054	4	Lung cancer	4671	0.08	0.72	0.90
ieu-b-5117	rs11609659	108296260	12	Lung cancer	5255	0.17	0.60	0.78
ieu-b-5117	rs34517439	78450517	1	Lung cancer	4866	0.01	0.99	0.99
ieu-b-5118	rs1250597	81010250	10	Lung cancer	6328	0.15	0.50	0.77
ieu-b-5118	rs215634	32369148	7	Lung cancer	6102	0.15	0.57	0.79
ieu-b-5118	rs34517439	78450517	1	Lung cancer	4866	0.01	0.99	0.99
ieu-b-5118	rs329118	133861663	5	Lung cancer	3925	0.01	0.98	0.99
prot-a-1051	rs550057	136146597	9	Lung cancer	6327	0.16	0.61	0.79
prot-a-1051	rs78534112	136327818	9	Lung cancer	6385	0.17	0.60	0.79
prot-a-1238	rs550057	136146597	9	Lung cancer	6327	0.20	0.52	0.72
ukb-a-205	rs2036527	78851615	15	Lung cancer	5531	0.02	0.98	0.98
ukb-a-205	rs414965	1324121	5	Lung cancer	6667	0.02	0.98	0.98
ukb-a-205	rs11571818	32968810	13	Lung cancer	5024	0.12	0.71	0.85
ukb-a-237	rs2002403	78681002	15	Lung cancer	5227	0.12	0.88	0.88
ukb-a-237	rs8034191	78806023	15	Lung cancer	5449	0.12	0.88	0.88
ukb-a-237	rs739447	136477511	9	Lung cancer	6116	0.16	0.60	0.79
ukb-a-238	rs2002403	78681002	15	Lung cancer	5227	0.15	0.85	0.85

ukb-a-238	rs739447	136477511	9	Lung cancer	6116	0.13	0.67	0.83
ukb-a-238	rs4887067	78886947	15	Lung cancer	5648	0.15	0.85	0.85
ukb-a-248	rs215634	32369148	7	Lung cancer	6095	0.13	0.63	0.83
ukb-a-248	rs74710200	108412850	12	Lung cancer	5387	0.16	0.62	0.79
ukb-a-248	rs34517439	78450517	1	Lung cancer	4998	0.00	1.00	1.00
ukb-a-248	rs72820274	104412924	2	Lung cancer	3969	0.11	0.75	0.88
ukb-a-248	rs329118	133861663	5	Lung cancer	4062	0.01	0.98	0.99
ukb-a-249	rs329118	133861663	5	Lung cancer	4062	0.01	0.98	0.99
ukb-a-249	rs34517439	78450517	1	Lung cancer	4998	0.01	0.99	0.99
ukb-a-249	rs541577	32382895	7	Lung cancer	6108	0.11	0.68	0.86
ukb-a-264	rs1324088	25841122	6	Lung cancer	5852	0.19	0.81	0.81
ukb-a-264	rs1653892	32399469	7	Lung cancer	6128	0.10	0.72	0.88
ukb-a-264	rs9358912	26211146	6	Lung cancer	5220	0.19	0.81	0.81
ukb-a-265	rs329118	133861663	5	Lung cancer	4062	0.01	0.98	0.99
ukb-a-265	rs1653892	32399469	7	Lung cancer	6128	0.11	0.68	0.86
ukb-a-265	rs34517439	78450517	1	Lung cancer	4998	0.00	1.00	1.00
ukb-a-272	rs2693698	99719219	14	Lung cancer	6292	0.15	0.54	0.78
ukb-a-273	rs34517439	78450517	1	Lung cancer	4998	0.10	0.90	0.90
ukb-a-274	rs4601460	134849254	9	Lung cancer	4856	0.12	0.58	0.83
ukb-a-274	rs1324087	25841408	6	Lung cancer	5850	0.19	0.81	0.81
ukb-a-274	rs12049202	77967523	1	Lung cancer	4900	0.05	0.95	0.95
ukb-a-274	rs1653892	32399469	7	Lung cancer	6128	0.09	0.74	0.89
ukb-a-274	rs6711584	104421692	2	Lung cancer	3967	0.08	0.81	0.91
ukb-a-275	rs541577	32382895	7	Lung cancer	6108	0.11	0.69	0.86
ukb-a-275	rs34517439	78450517	1	Lung cancer	4998	0.00	1.00	1.00
ukb-a-275	rs329118	133861663	5	Lung cancer	4062	0.01	0.98	0.99
ukb-a-275	rs72820274	104412924	2	Lung cancer	3969	0.11	0.73	0.87
ukb-a-275	rs1624064	26378681	6	Lung cancer	5113	0.22	0.78	0.78
ukb-a-277	rs34517439	78450517	1	Lung cancer	4998	0.01	0.99	0.99
ukb-a-277	rs1967315	129224182	8	Lung cancer	5711	0.27	0.64	0.70
ukb-a-277	rs8126213	62611478	20	Lung cancer	4317	0.29	0.70	0.70
ukb-a-278	rs4082793	99700080	14	Lung cancer	6330	0.12	0.64	0.85
ukb-a-278	rs6711584	104421692	2	Lung cancer	3967	0.08	0.81	0.91
ukb-a-278	rs1653892	32399469	7	Lung cancer	6128	0.10	0.72	0.88
ukb-a-278	rs75499503	26145217	6	Lung cancer	5210	0.28	0.72	0.72
ukb-a-279	rs34517439	78450517	1	Lung cancer	4998	0.00	1.00	1.00
ukb-a-279	rs541577	32382895	7	Lung cancer	6108	0.11	0.69	0.86
ukb-a-279	rs72820274	104412924	2	Lung cancer	3969	0.11	0.74	0.87
ukb-a-279	rs329118	133861663	5	Lung cancer	4062	0.01	0.98	0.99
ukb-a-279	rs1624064	26378681	6	Lung cancer	5113	0.21	0.79	0.79
ukb-a-281	rs116165844	62610556	20	Lung cancer	4320	0.29	0.69	0.70
ukb-a-281	rs34517439	78450517	1	Lung cancer	4998	0.01	0.99	0.99
ukb-a-281	rs7220	21863290	8	Lung cancer	5942	0.11	0.76	0.87

ukb-a-282	rs11609659	108296260	12	Lung cancer	5466	0.19	0.56	0.74
ukb-a-282	rs1653892	32399469	7	Lung cancer	6128	0.12	0.67	0.85
ukb-a-282	rs329118	133861663	5	Lung cancer	4062	0.01	0.97	0.99
ukb-a-283	rs34517439	78450517	1	Lung cancer	4998	0.00	1.00	1.00
ukb-a-283	rs541577	32382895	7	Lung cancer	6108	0.11	0.67	0.85
ukb-a-283	rs329118	133861663	5	Lung cancer	4062	0.01	0.98	0.99
ukb-a-284	rs34517439	78450517	1	Lung cancer	4998	0.01	0.99	0.99
ukb-a-284	rs116165844	62610556	20	Lung cancer	4320	0.29	0.70	0.71
ukb-a-286	rs329118	133861663	5	Lung cancer	4062	0.01	0.98	0.99
ukb-a-286	rs1653892	32399469	7	Lung cancer	6128	0.10	0.70	0.87
ukb-a-287	rs329118	133861663	5	Lung cancer	4062	0.01	0.98	0.99
ukb-a-287	rs541577	32382895	7	Lung cancer	6108	0.11	0.69	0.86
ukb-a-287	rs34517439	78450517	1	Lung cancer	4998	0.01	0.99	0.99
ukb-a-291	rs34517439	78450517	1	Lung cancer	4998	0.01	0.99	0.99
ukb-a-291	rs1653892	32399469	7	Lung cancer	6128	0.11	0.69	0.86
ukb-a-328	rs62012629	79070351	15	Lung cancer	5904	0.06	0.94	0.94
ukb-a-328	rs2002403	78681002	15	Lung cancer	5227	0.06	0.94	0.94
ukb-a-328	rs58365910	78849034	15	Lung cancer	5528	0.06	0.94	0.94
ukb-a-328	rs184589612	41412192	19	Lung cancer	4470	0.00	1.00	1.00
ukb-a-328	rs56113850	41353107	19	Lung cancer	4516	0.00	1.00	1.00
ukb-a-342	rs11671669	41324392	19	Lung cancer	4571	0.00	1.00	1.00
ukb-a-342	rs8034191	78806023	15	Lung cancer	5449	0.03	0.97	0.97
ukb-a-342	rs56113850	41353107	19	Lung cancer	4516	0.00	1.00	1.00
ukb-a-382	rs74710200	108412850	12	Lung cancer	5387	0.17	0.61	0.79
ukb-a-382	rs541577	32382895	7	Lung cancer	6108	0.14	0.61	0.82
ukb-a-397	rs2860049	47515395	15	Lung cancer	5050	0.07	0.93	0.93
ukb-a-397	rs7902	95565288	11	Lung cancer	4834	0.17	0.71	0.81
ukb-a-397	rs9357004	26303319	6	Lung cancer	5184	0.05	0.95	0.95
ukb-a-398	rs10765777	95656385	11	Lung cancer	4918	0.11	0.80	0.88
ukb-a-5	rs10831418	95468828	11	Lung cancer	4730	0.20	0.66	0.77
ukb-a-505	rs7110786	95656648	11	Lung cancer	4920	0.14	0.75	0.84
ukb-b-10831	rs4900590	104146421	14	Lung cancer	5651	0.12	0.64	0.84
ukb-b-10831	rs3025360	136481205	9	Lung cancer	6206	0.17	0.58	0.78
ukb-b-11615	rs3808977	95657761	11	Lung cancer	4955	0.11	0.81	0.88
ukb-b-11615	rs2640564	56455294	12	Lung cancer	3493	0.12	0.58	0.82
ukb-b-12018	rs111596452	136485294	9	Lung cancer	5864	0.17	0.56	0.77
ukb-b-12039	rs329118	133861663	5	Lung cancer	4085	0.01	0.98	0.99
ukb-b-12039	rs1624064	26378681	6	Lung cancer	5175	0.22	0.78	0.78
ukb-b-12039	rs34517439	78450517	1	Lung cancer	5029	0.01	0.99	0.99
ukb-b-12405	rs2456973	56416928	12	Lung cancer	3520	0.12	0.59	0.84
ukb-b-12854	rs329118	133861663	5	Lung cancer	4085	0.01	0.98	0.99
ukb-b-12854	rs215669	32378979	7	Lung cancer	6139	0.13	0.62	0.82
ukb-b-13952	rs2036527	78851615	15	Lung cancer	4488	0.01	0.99	0.99

ukb-b-14521	rs62012629	79070351	15	Lung cancer	5862	0.03	0.97	0.97
ukb-b-14521	rs421284	1325590	5	Lung cancer	6726	0.02	0.98	0.98
ukb-b-14521	rs72740955	78849779	15	Lung cancer	5488	0.03	0.97	0.97
ukb-b-14540	rs34517439	78450517	1	Lung cancer	5029	0.01	0.99	0.99
ukb-b-15590	rs34517439	78450517	1	Lung cancer	5029	0.01	0.99	0.99
ukb-b-15590	rs1624064	26378681	6	Lung cancer	5175	0.23	0.77	0.77
ukb-b-15590	rs1328262	25458217	6	Lung cancer	6538	0.13	0.87	0.87
ukb-b-15590	rs329118	133861663	5	Lung cancer	4085	0.01	0.98	0.99
ukb-b-16407	rs9379833	26207175	6	Lung cancer	5277	0.21	0.79	0.79
ukb-b-16446	rs34517439	78450517	1	Lung cancer	5029	0.01	0.99	0.99
ukb-b-16446	rs1624064	26378681	6	Lung cancer	5175	0.23	0.77	0.77
ukb-b-16489	rs7902	95565288	11	Lung cancer	4865	0.20	0.66	0.77
ukb-b-16489	rs329122	133864599	5	Lung cancer	4073	0.02	0.94	0.98
ukb-b-16489	rs6497751	24702978	16	Lung cancer	5685	0.13	0.51	0.80
ukb-b-16489	rs6493265	47513253	15	Lung cancer	5123	0.07	0.93	0.93
ukb-b-16698	rs34517439	78450517	1	Lung cancer	5029	0.01	0.99	0.99
ukb-b-16878	rs72720396	91191582	1	Lung cancer	4715	0.14	0.82	0.85
ukb-b-16878	rs806789	26229161	6	Lung cancer	5270	0.08	0.92	0.92
ukb-b-17729	rs13141210	67891641	4	Lung cancer	5246	0.17	0.57	0.77
ukb-b-17729	rs10765777	95656385	11	Lung cancer	4941	0.12	0.80	0.87
ukb-b-17729	rs12046747	204593696	1	Lung cancer	5384	0.06	0.89	0.93
ukb-b-17729	rs4962223	134930213	9	Lung cancer	4936	0.12	0.57	0.83
ukb-b-18096	rs35390852	143067054	4	Lung cancer	4803	0.16	0.51	0.77
ukb-b-18096	rs329118	133861663	5	Lung cancer	4085	0.01	0.98	0.99
ukb-b-18096	rs4601460	134849254	9	Lung cancer	4901	0.12	0.59	0.83
ukb-b-18096	rs215634	32369148	7	Lung cancer	6122	0.15	0.56	0.78
ukb-b-18096	rs1624064	26378681	6	Lung cancer	5175	0.24	0.76	0.76
ukb-b-18096	rs34517439	78450517	1	Lung cancer	5029	0.01	0.99	0.99
ukb-b-18377	rs1749850	80999977	10	Lung cancer	6273	0.14	0.54	0.79
ukb-b-18377	rs329118	133861663	5	Lung cancer	4085	0.01	0.96	0.99
ukb-b-18377	rs34517439	78450517	1	Lung cancer	5029	0.02	0.98	0.98
ukb-b-18377	rs215669	32378979	7	Lung cancer	6139	0.14	0.60	0.81
ukb-b-19379	rs34517439	78450517	1	Lung cancer	5029	0.01	0.99	0.99
ukb-b-19379	rs7936928	130279168	11	Lung cancer	5535	0.06	0.82	0.93
ukb-b-19393	rs329118	133861663	5	Lung cancer	4085	0.01	0.98	0.99
ukb-b-19393	rs215634	32369148	7	Lung cancer	6122	0.16	0.55	0.78
ukb-b-19393	rs1624064	26378681	6	Lung cancer	5175	0.24	0.76	0.76
ukb-b-19393	rs34517439	78450517	1	Lung cancer	5029	0.01	0.99	0.99
ukb-b-19520	rs34517439	78450517	1	Lung cancer	5029	0.01	0.99	0.99
ukb-b-19921	rs34517439	78450517	1	Lung cancer	5029	0.01	0.99	0.99
ukb-b-19921	rs329118	133861663	5	Lung cancer	4085	0.02	0.93	0.98
ukb-b-19921	rs7823498	32403573	8	Lung cancer	5031	0.01	0.98	0.99
ukb-b-19921	rs2065221	127020828	9	Lung cancer	4249	0.10	0.51	0.84

ukb-b-19925	rs1624064	26378681	6	Lung cancer	5175	0.23	0.77	0.77
ukb-b-19925	rs34517439	78450517	1	Lung cancer	5029	0.01	0.99	0.99
ukb-b-19953	rs17645692	32369901	8	Lung cancer	5046	0.14	0.78	0.85
ukb-b-19953	rs35390852	143067054	4	Lung cancer	4803	0.14	0.57	0.81
ukb-b-19953	rs34517439	78450517	1	Lung cancer	5029	0.01	0.99	0.99
ukb-b-19953	rs329118	133861663	5	Lung cancer	4085	0.01	0.98	0.99
ukb-b-19953	rs2466103	32412304	8	Lung cancer	5017	0.15	0.77	0.84
ukb-b-19953	rs73193736	108294381	12	Lung cancer	5510	0.17	0.60	0.78
ukb-b-19953	rs215634	32369148	7	Lung cancer	6122	0.16	0.55	0.78
ukb-b-20044	rs31251	130833946	5	Lung cancer	3389	0.23	0.53	0.70
ukb-b-20044	rs215634	32369148	7	Lung cancer	6122	0.15	0.56	0.78
ukb-b-20044	rs34517439	78450517	1	Lung cancer	5029	0.01	0.99	0.99
ukb-b-20188	rs215669	32378979	7	Lung cancer	6139	0.14	0.60	0.81
ukb-b-20188	rs329124	133865452	5	Lung cancer	4075	0.01	0.98	0.99
ukb-b-20531	rs4601460	134849254	9	Lung cancer	4901	0.12	0.58	0.83
ukb-b-20531	rs34517439	78450517	1	Lung cancer	5029	0.03	0.97	0.97
ukb-b-20531	rs75499503	26145217	6	Lung cancer	5273	0.30	0.70	0.70
ukb-b-20531	rs35390852	143067054	4	Lung cancer	4803	0.14	0.56	0.80
ukb-b-20531	rs215669	32378979	7	Lung cancer	6139	0.13	0.63	0.83
ukb-b-2303	rs73193736	108294381	12	Lung cancer	5510	0.18	0.57	0.76
ukb-b-2303	rs34517439	78450517	1	Lung cancer	5029	0.01	0.99	0.99
ukb-b-2303	rs215634	32369148	7	Lung cancer	6122	0.16	0.55	0.78
ukb-b-2303	rs329118	133861663	5	Lung cancer	4085	0.01	0.98	0.99
ukb-b-2303	rs35390852	143067054	4	Lung cancer	4803	0.12	0.62	0.84
ukb-b-2303	rs17645692	32369901	8	Lung cancer	5046	0.11	0.83	0.89
ukb-b-2303	rs2466103	32412304	8	Lung cancer	5017	0.11	0.82	0.88
ukb-b-2732	rs6011779	61984317	20	Lung cancer	6171	0.03	0.96	0.97
ukb-b-2732	rs7867984	136485598	9	Lung cancer	6029	0.03	0.92	0.97
ukb-b-2732	rs8034191	78806023	15	Lung cancer	5379	0.12	0.88	0.88
ukb-b-3599	rs66972160	26359306	6	Lung cancer	4193	0.04	0.96	0.96
ukb-b-469	rs56113850	41353107	19	Lung cancer	4527	0.00	1.00	1.00
ukb-b-469	rs11671669	41324392	19	Lung cancer	4585	0.00	1.00	1.00
ukb-b-5174	rs12046000	91192396	1	Lung cancer	4720	0.10	0.88	0.90
ukb-b-5192	rs10765776	95656364	11	Lung cancer	4951	0.11	0.81	0.88
ukb-b-6019	rs806795	26205293	6	Lung cancer	5270	0.08	0.92	0.92
ukb-b-6019	rs56113850	41353107	19	Lung cancer	4653	0.00	1.00	1.00
ukb-b-6019	rs2002403	78681002	15	Lung cancer	5280	0.05	0.95	0.95
ukb-b-6019	rs11671669	41324392	19	Lung cancer	4710	0.00	1.00	1.00
ukb-b-6019	rs184589612	41412192	19	Lung cancer	4602	0.00	1.00	1.00
ukb-b-6019	rs72740955	78849779	15	Lung cancer	5575	0.05	0.95	0.95
ukb-b-6134	rs7110786	95656648	11	Lung cancer	4953	0.14	0.76	0.84
ukb-b-6591	rs6692613	7524974	1	Lung cancer	5116	0.09	0.60	0.87
ukb-b-6591	rs7025089	134881443	9	Lung cancer	4946	0.11	0.59	0.84

ukb-b-6591	rs34517439	78450517	1	Lung cancer	5029	0.17	0.83	0.83
ukb-b-6704	rs11609659	108296260	12	Lung cancer	5504	0.18	0.58	0.76
ukb-b-6704	rs329118	133861663	5	Lung cancer	4085	0.01	0.98	0.99
ukb-b-6704	rs215634	32369148	7	Lung cancer	6122	0.15	0.58	0.80
ukb-b-6704	rs34517439	78450517	1	Lung cancer	5029	0.01	0.99	0.99
ukb-b-6704	rs1624064	26378681	6	Lung cancer	5175	0.23	0.77	0.77
ukb-b-6704	rs35390852	143067054	4	Lung cancer	4803	0.12	0.62	0.84
ukb-b-7212	rs35390852	143067054	4	Lung cancer	4803	0.10	0.68	0.87
ukb-b-7212	rs34517439	78450517	1	Lung cancer	5029	0.01	0.99	0.99
ukb-b-7212	rs329118	133861663	5	Lung cancer	4085	0.01	0.98	0.99
ukb-b-7212	rs1624064	26378681	6	Lung cancer	5175	0.24	0.76	0.76
ukb-b-7212	rs215669	32378979	7	Lung cancer	6139	0.16	0.55	0.77
ukb-b-7408	rs387780	32502495	2	Lung cancer	5353	0.09	0.82	0.90
ukb-b-7408	rs11588857	204587047	1	Lung cancer	5392	0.23	0.58	0.71
ukb-b-7460	rs62012628	79070000	15	Lung cancer	5955	0.16	0.84	0.84
ukb-b-7460	rs8042849	78817929	15	Lung cancer	5523	0.16	0.84	0.84
ukb-b-7460	rs3025360	136481205	9	Lung cancer	6206	0.16	0.61	0.79
ukb-b-7460	rs4900590	104146421	14	Lung cancer	5651	0.12	0.64	0.84
ukb-b-8338	rs329118	133861663	5	Lung cancer	4085	0.01	0.98	0.99
ukb-b-8338	rs34517439	78450517	1	Lung cancer	5029	0.01	0.99	0.99
ukb-b-8338	rs11609659	108296260	12	Lung cancer	5504	0.19	0.56	0.75
ukb-b-8338	rs215634	32369148	7	Lung cancer	6122	0.15	0.58	0.80
ukb-b-8909	rs9379833	26207175	6	Lung cancer	5277	0.20	0.80	0.80
ukb-b-8909	rs1324088	25841122	6	Lung cancer	5915	0.20	0.80	0.80
ukb-b-8909	rs215669	32378979	7	Lung cancer	6139	0.15	0.56	0.78
ukb-b-8909	rs329118	133861663	5	Lung cancer	4085	0.01	0.98	0.99
ukb-b-9093	rs34517439	78450517	1	Lung cancer	5029	0.01	0.99	0.99
ukb-b-9093	rs1624064	26378681	6	Lung cancer	5175	0.23	0.77	0.77
ukb-b-9405	rs215669	32378979	7	Lung cancer	6139	0.16	0.55	0.78
ukb-b-9405	rs34517439	78450517	1	Lung cancer	5029	0.01	0.99	0.99
ukb-b-9405	rs329118	133861663	5	Lung cancer	4085	0.01	0.98	0.99
ukb-b-9405	rs6711584	104421692	2	Lung cancer	3982	0.12	0.71	0.85
ukb-b-9405	rs7826312	32400115	8	Lung cancer	5036	0.17	0.74	0.81
ukb-b-969	rs7587930	199504423	2	Lung cancer	2964	0.11	0.79	0.88
ukb-b-969	rs34517439	78450517	1	Lung cancer	5029	0.00	1.00	1.00
ukb-b-969	rs644799	95564259	11	Lung cancer	4867	0.13	0.78	0.86
ukb-d-20116_0	rs772921	56403577	12	Lung cancer	3872	0.13	0.58	0.82

supplementary Table 17 The colocalization result between exposures and LUSC

id.exposure	SNP	pos.exposure	chr	id	snp	PPH3	PPH4	PPH5
ebi-a-GCST004604	rs2732480	48736303	12	LUSC	5548	0.17	0.59	0.77
ebi-a-GCST006250	rs3896224	106467853	10	LUSC	4647	0.13	0.56	0.81
ebi-a-GCST006250	rs566237	11543342	6	LUSC	5370	0.15	0.58	0.80
ebi-a-GCST007432	rs931794	78826180	15	LUSC	5610	0.17	0.83	0.83
ebi-a-GCST009971	rs11671669	41324392	19	LUSC	4244	0.00	1.00	1.00
ebi-a-GCST009971	rs11881522	41403509	19	LUSC	4162	0.00	1.00	1.00
ebi-a-GCST009971	rs149293272	41776752	19	LUSC	3736	0.00	1.00	1.00
ebi-a-GCST009971	rs56113850	41353107	19	LUSC	4173	0.00	1.00	1.00
ebi-a-GCST009971	rs62117431	42420808	19	LUSC	3209	0.10	0.71	0.87
ebi-a-GCST011365	rs2891168	22098619	9	LUSC	5707	0.02	0.98	0.98
ebi-a-GCST011365	rs41290120	45382675	19	LUSC	5477	0.07	0.79	0.92
ebi-a-GCST011365	rs429358	45411941	19	LUSC	5534	0.07	0.79	0.92
ebi-a-GCST90000045	rs12203592	396321	6	LUSC	1019	0.03	0.60	0.95
ebi-a-GCST90000046	rs3896224	106467853	10	LUSC	4959	0.15	0.52	0.78
ebi-a-GCST90000047	rs12203592	396321	6	LUSC	4176	0.10	0.53	0.84
ebi-a-GCST90000047	rs12907546	47684280	15	LUSC	4923	0.10	0.75	0.88
ebi-a-GCST90000047	rs34517439	78450517	1	LUSC	5311	0.22	0.56	0.72
ebi-a-GCST90000047	rs3896224	106467853	10	LUSC	4959	0.11	0.64	0.85
ebi-a-GCST90000047	rs838042	140320186	2	LUSC	6532	0.17	0.68	0.80
ebi-a-GCST90000048	rs329122	133864599	5	LUSC	4447	0.01	0.98	0.99
ebi-a-GCST90000050	rs11242222	133875033	5	LUSC	4400	0.02	0.97	0.98
ebi-a-GCST90000050	rs17391694	78623626	1	LUSC	5368	0.15	0.62	0.80
ebi-a-GCST90000514	rs215614	32347335	7	LUSC	2046	0.10	0.60	0.86
ebi-a-GCST90000514	rs2782641	44013355	1	LUSC	1302	0.08	0.89	0.92
ebi-a-GCST90000514	rs329122	133864599	5	LUSC	1433	0.01	0.98	0.99
ebi-a-GCST90013870	rs13176429	43152216	5	LUSC	4666	0.12	0.81	0.87
ebi-a-GCST90013870	rs2066295	26168903	6	LUSC	4491	0.01	0.98	0.99
ebi-a-GCST90013870	rs329118	133861663	5	LUSC	4493	0.01	0.98	0.99
ebi-a-GCST90013870	rs34517439	78450517	1	LUSC	5227	0.03	0.92	0.97
ebi-a-GCST90013870	rs7132908	50263148	12	LUSC	4116	0.01	0.97	0.99
ebi-a-GCST90013972	rs34684276	78813155	15	LUSC	6125	0.19	0.81	0.81
ebi-a-GCST90013972	rs421629	1320136	5	LUSC	7628	0.04	0.96	0.96
ebi-a-GCST90013972	rs57708073	79066653	15	LUSC	5605	0.19	0.81	0.81
ebi-a-GCST90013974	rs13176429	43152216	5	LUSC	4666	0.12	0.80	0.87
ebi-a-GCST90013974	rs329118	133861663	5	LUSC	4493	0.01	0.98	0.99
ebi-a-GCST90013974	rs34517439	78450517	1	LUSC	5227	0.03	0.92	0.97
ebi-a-GCST90013974	rs7132908	50263148	12	LUSC	4116	0.01	0.97	0.99
ebi-a-GCST90013975	rs12367809	50256063	12	LUSC	4103	0.01	0.98	0.99
ebi-a-GCST90013975	rs4884331	59878922	13	LUSC	5446	0.19	0.73	0.79
ebi-a-GCST90014020	rs10153031	67987293	15	LUSC	4423	0.20	0.64	0.76
ebi-a-GCST90014020	rs12367809	50256063	12	LUSC	4103	0.01	0.98	0.99

ebi-a-GCST90014020	rs34517439	78450517	1	LUSC	5227	0.03	0.93	0.97
ebi-a-GCST90018793	rs1333042	22103813	9	LUSC	5725	0.07	0.92	0.93
ebi-a-GCST90018793	rs2106115	21959900	9	LUSC	5570	0.07	0.92	0.93
ebi-a-GCST90018890	rs10757271	22076795	9	LUSC	5670	0.09	0.91	0.91
ebi-a-GCST90018947	rs10941611	43162608	5	LUSC	4684	0.10	0.83	0.89
ebi-a-GCST90018947	rs2297146	27382236	10	LUSC	6165	0.27	0.72	0.72
ebi-a-GCST90018947	rs329124	133865452	5	LUSC	4314	0.01	0.98	0.99
ebi-a-GCST90018947	rs34517439	78450517	1	LUSC	5145	0.05	0.91	0.95
ebi-a-GCST90018947	rs7132908	50263148	12	LUSC	3645	0.02	0.96	0.98
ebi-a-GCST90018947	rs76095247	66593468	15	LUSC	5393	0.14	0.73	0.84
ebi-a-GCST90018949	rs329118	133861663	5	LUSC	4325	0.01	0.98	0.99
ebi-a-GCST90018949	rs34517439	78450517	1	LUSC	5145	0.05	0.91	0.95
ebi-a-GCST90018949	rs7132908	50263148	12	LUSC	3645	0.02	0.96	0.98
ebi-a-GCST90018982	rs10757274	22096055	9	LUSC	5326	0.02	0.98	0.98
ebi-a-GCST90018982	rs7412	45412079	19	LUSC	5196	0.11	0.63	0.85
ebi-a-GCST90018982	rs769449	45410002	19	LUSC	5193	0.11	0.63	0.85
ebi-a-GCST90018992	rs146683910	31335997	6	LUSC	517	0.01	0.98	0.99
ebi-a-GCST90019476	rs192351044	140086704	5	LUSC	3247	0.09	0.69	0.89
ebi-a-GCST90019476	rs5744441	140016847	5	LUSC	3096	0.08	0.69	0.89
ebi-a-GCST90025994	rs112095287	66792087	15	LUSC	535	0.02	0.73	0.98
ebi-a-GCST90025994	rs17391694	78623626	1	LUSC	358	0.01	0.71	0.98
ebi-a-GCST90025994	rs215614	32347335	7	LUSC	583	0.02	0.65	0.97
ebi-a-GCST90025994	rs329118	133861663	5	LUSC	432	0.00	0.99	1.00
ebi-a-GCST90025994	rs7132908	50263148	12	LUSC	443	0.00	0.97	1.00
ebi-a-GCST90025994	rs73601548	18549889	10	LUSC	424	0.02	0.52	0.96
ebi-a-GCST90029007	rs10935143	134665159	3	LUSC	4579	0.17	0.54	0.76
ebi-a-GCST90029007	rs13176429	43152216	5	LUSC	4500	0.12	0.80	0.87
ebi-a-GCST90029007	rs329124	133865452	5	LUSC	4302	0.01	0.98	0.99
ebi-a-GCST90029007	rs34517439	78450517	1	LUSC	5080	0.05	0.91	0.95
ebi-a-GCST90029007	rs7132908	50263148	12	LUSC	3613	0.01	0.97	0.99
ebi-a-GCST90029012	rs11690224	4952314	2	LUSC	6143	0.20	0.51	0.72
ebi-a-GCST90029013	rs215632	32368524	7	LUSC	6196	0.21	0.54	0.72
ebi-a-GCST90029014	rs12910916	47675655	15	LUSC	4804	0.18	0.54	0.75
ebi-a-GCST90029014	rs329120	133861756	5	LUSC	4313	0.01	0.98	0.99
ebi-a-GCST90029014	rs3896224	106467853	10	LUSC	4788	0.12	0.63	0.84
eqtl-a-ENSG00000041357	rs57708073	79066653	15	LUSC	5872	0.18	0.82	0.82
eqtl-a-ENSG00000041357	rs6495304	78800416	15	LUSC	5872	0.18	0.82	0.82
eqtl-a-ENSG00000041357	rs931794	78826180	15	LUSC	5872	0.18	0.82	0.82
eqtl-a-ENSG00000074657	rs140045584	56307643	18	LUSC	6035	0.22	0.58	0.72
eqtl-a-ENSG00000074657	rs145735679	56554764	18	LUSC	6035	0.22	0.58	0.72
eqtl-a-ENSG00000074657	rs146252031	56646739	18	LUSC	6035	0.22	0.58	0.72
eqtl-a-ENSG00000074657	rs34538306	56656824	18	LUSC	6035	0.22	0.58	0.72
eqtl-a-ENSG00000074657	rs9961719	56489137	18	LUSC	6035	0.22	0.58	0.72

eqtl-a-ENSG00000106305	rs113242762	6531360	7	LUSC	7482	0.17	0.61	0.78
eqtl-a-ENSG00000106305	rs117419850	5917073	7	LUSC	7482	0.17	0.61	0.78
eqtl-a-ENSG00000106305	rs74189692	6037723	7	LUSC	7482	0.17	0.61	0.78
eqtl-a-ENSG00000106305	rs852479	5639126	7	LUSC	7482	0.17	0.61	0.78
eqtl-a-ENSG00000107890	rs10829226	27573952	10	LUSC	6398	0.14	0.86	0.86
eqtl-a-ENSG00000107890	rs118183234	26965158	10	LUSC	6398	0.14	0.86	0.86
eqtl-a-ENSG00000107890	rs144610740	26975803	10	LUSC	6398	0.14	0.86	0.86
eqtl-a-ENSG00000107890	rs73598041	27475275	10	LUSC	6398	0.14	0.86	0.86
eqtl-a-ENSG00000107890	rs77728719	28235976	10	LUSC	6398	0.14	0.86	0.86
eqtl-a-ENSG00000162736	rs11579550	160348664	1	LUSC	5362	0.08	0.91	0.92
eqtl-a-ENSG00000162736	rs116011917	160275992	1	LUSC	5362	0.08	0.91	0.92
eqtl-a-ENSG00000162736	rs16831677	160209273	1	LUSC	5362	0.08	0.91	0.92
eqtl-a-ENSG00000162736	rs617698	160162872	1	LUSC	5362	0.08	0.91	0.92
eqtl-a-ENSG00000162736	rs72706674	160282703	1	LUSC	5362	0.08	0.91	0.92
eqtl-a-ENSG00000163788	rs2135511	43368539	3	LUSC	3764	0.02	0.94	0.98
finn-b-CD2_BENIGN_EXALLC	rs61913672	107983096	11	LUSC	4960	0.17	0.58	0.77
finn-b-CD2_BENIGN_EXALLC	rs78378222	7571752	17	LUSC	5118	0.22	0.51	0.70
finn-b-CD2_BENIGN_LEIOMYOMA_UTERI	rs78378222	7571752	17	LUSC	5118	0.22	0.51	0.70
finn-b-DM_PERIPHATHERO	rs10757271	22076795	9	LUSC	5555	0.12	0.87	0.88
finn-b-I9_ANGINA	rs10757272	22088260	9	LUSC	5557	0.04	0.96	0.96
finn-b-I9_ANGINA	rs2106116	21959966	9	LUSC	5467	0.04	0.96	0.96
finn-b-I9_ANGINA	rs7412	45412079	19	LUSC	5659	0.15	0.54	0.78
finn-b-I9_CABG	rs1537371	22099568	9	LUSC	5596	0.09	0.91	0.91
finn-b-I9_CABG	rs2106115	21959900	9	LUSC	5467	0.09	0.91	0.91
finn-b-I9_CABG	rs7412	45412079	19	LUSC	5659	0.07	0.77	0.91
finn-b-I9_CABG_EXNONE	rs1537371	22099568	9	LUSC	5596	0.09	0.91	0.91
finn-b-I9_CABG_EXNONE	rs2106115	21959900	9	LUSC	5467	0.09	0.91	0.91
finn-b-I9_CORATHER	rs7412	45412079	19	LUSC	5659	0.16	0.51	0.76
finn-b-I9_CORATHER_EXNONE	rs1537371	22099568	9	LUSC	5596	0.09	0.91	0.91
finn-b-I9_CORATHER_EXNONE	rs7412	45412079	19	LUSC	5659	0.16	0.52	0.76
finn-b-I9_REVASC	rs1537371	22099568	9	LUSC	5596	0.09	0.91	0.91
finn-b-I9_REVASC	rs7412	45412079	19	LUSC	5659	0.15	0.54	0.78
ieu-a-1001	rs11588857	204587047	1	LUSC	5228	0.24	0.57	0.70
ieu-a-1001	rs12410444	44188719	1	LUSC	4286	0.12	0.86	0.87
ieu-a-1001	rs1378214	47579004	15	LUSC	4606	0.16	0.60	0.79
ieu-a-1001	rs56236451	237056840	2	LUSC	5692	0.15	0.59	0.80
ieu-a-1239	rs2819336	44015809	1	LUSC	4224	0.21	0.77	0.79
ieu-a-1239	rs6871635	133830395	5	LUSC	4258	0.17	0.71	0.81
ieu-a-1239	rs7603132	4951548	2	LUSC	6072	0.20	0.50	0.72
ieu-b-104	rs17843614	32620661	6	LUSC	573	0.03	0.82	0.97
ieu-b-142	rs11671669	41324392	19	LUSC	4387	0.00	1.00	1.00
ieu-b-142	rs2273500	61986949	20	LUSC	5619	0.05	0.91	0.95

ieu-b-142	rs3025383	136502369	9	LUSC	5706	0.01	0.99	0.99
ieu-b-142	rs56113850	41353107	19	LUSC	4320	0.00	1.00	1.00
ieu-b-142	rs56116178	136460224	9	LUSC	5707	0.01	0.99	0.99
ieu-b-142	rs57708073	79066653	15	LUSC	5351	0.05	0.95	0.95
ieu-b-142	rs8034191	78806023	15	LUSC	5191	0.05	0.95	0.95
ieu-b-25	rs11671669	41324392	19	LUSC	4386	0.00	1.00	1.00
ieu-b-25	rs2273500	61986949	20	LUSC	5619	0.05	0.91	0.95
ieu-b-25	rs3025383	136502369	9	LUSC	5701	0.01	0.99	0.99
ieu-b-25	rs56113850	41353107	19	LUSC	4319	0.00	1.00	1.00
ieu-b-25	rs56116178	136460224	9	LUSC	5702	0.01	0.99	0.99
ieu-b-25	rs57708073	79066653	15	LUSC	5351	0.05	0.95	0.95
ieu-b-25	rs8034191	78806023	15	LUSC	5191	0.05	0.95	0.95
ieu-b-40	rs12595158	62316035	15	LUSC	1973	0.08	0.67	0.89
ieu-b-40	rs17391694	78623626	1	LUSC	1480	0.05	0.68	0.93
ieu-b-40	rs329122	133864599	5	LUSC	1358	0.01	0.99	0.99
ieu-b-40	rs7138803	50247468	12	LUSC	811	0.00	0.97	1.00
ieu-b-40	rs7730004	43191033	5	LUSC	993	0.03	0.89	0.97
ieu-b-4877	rs12474587	162802993	2	LUSC	3483	0.11	0.55	0.83
ieu-b-4877	rs1899896	93201036	8	LUSC	3482	0.15	0.50	0.77
ieu-b-5113	rs12036042	204469314	1	LUSC	4777	0.11	0.80	0.88
ieu-b-5113	rs6904596	27491299	6	LUSC	4397	0.25	0.75	0.75
ieu-b-5113	rs72738786	78828086	15	LUSC	5532	0.07	0.93	0.93
ieu-b-5117	rs34517439	78450517	1	LUSC	4890	0.06	0.86	0.94
ieu-b-5117	rs7132908	50263148	12	LUSC	3458	0.01	0.98	0.99
ieu-b-5118	rs10935143	134665159	3	LUSC	4445	0.16	0.56	0.78
ieu-b-5118	rs329118	133861663	5	LUSC	4103	0.01	0.98	0.99
ieu-b-5118	rs34517439	78450517	1	LUSC	4890	0.04	0.92	0.96
ieu-b-5118	rs7132908	50263148	12	LUSC	3458	0.01	0.97	0.99
ubm-b-3277	rs2284871	162888055	2	LUSC				
ubm-b-3277	rs6716700	48192190	2	LUSC				
ubm-b-3277	rs67316928	89482234	3	LUSC				
ukb-a-205	rs11571818	32968810	13	LUSC	4869	0.12	0.70	0.85
ukb-a-205	rs2036527	78851615	15	LUSC	5545	0.06	0.94	0.94
ukb-a-205	rs414965	1324121	5	LUSC	6806	0.04	0.96	0.96
ukb-a-237	rs2002403	78681002	15	LUSC	5413	0.12	0.88	0.88
ukb-a-237	rs2316205	41346768	19	LUSC	4537	0.04	0.96	0.96
ukb-a-237	rs8034191	78806023	15	LUSC	5558	0.12	0.88	0.88
ukb-a-238	rs2002403	78681002	15	LUSC	5413	0.12	0.88	0.88
ukb-a-238	rs2316205	41346768	19	LUSC	4537	0.01	0.99	0.99
ukb-a-238	rs4887067	78886947	15	LUSC	5569	0.12	0.88	0.88
ukb-a-248	rs329118	133861663	5	LUSC	4260	0.01	0.98	0.99
ukb-a-248	rs34517439	78450517	1	LUSC	5027	0.04	0.92	0.96
ukb-a-248	rs7132908	50263148	12	LUSC	3538	0.01	0.97	0.99

ukb-a-248	rs7723426	43186097	5	LUSC	4488	0.13	0.79	0.86
ukb-a-249	rs329118	133861663	5	LUSC	4260	0.01	0.98	0.99
ukb-a-249	rs34517439	78450517	1	LUSC	5027	0.05	0.91	0.95
ukb-a-249	rs7132908	50263148	12	LUSC	3538	0.02	0.96	0.98
ukb-a-264	rs12367809	50256063	12	LUSC	3534	0.01	0.99	0.99
ukb-a-264	rs1653892	32399469	7	LUSC	6186	0.21	0.55	0.73
ukb-a-265	rs12367809	50256063	12	LUSC	3534	0.01	0.98	0.99
ukb-a-265	rs329118	133861663	5	LUSC	4260	0.01	0.98	0.99
ukb-a-265	rs34517439	78450517	1	LUSC	5027	0.04	0.91	0.95
ukb-a-265	rs3784699	68007591	15	LUSC	4819	0.19	0.67	0.78
ukb-a-274	rs10999460	72428283	10	LUSC	6915	0.21	0.60	0.74
ukb-a-274	rs12367809	50256063	12	LUSC	3534	0.01	0.98	0.99
ukb-a-274	rs1653892	32399469	7	LUSC	6186	0.19	0.58	0.75
ukb-a-274	rs3784699	68007591	15	LUSC	4819	0.20	0.64	0.76
ukb-a-275	rs329118	133861663	5	LUSC	4260	0.01	0.98	0.99
ukb-a-275	rs34517439	78450517	1	LUSC	5027	0.04	0.92	0.96
ukb-a-275	rs3784699	68007591	15	LUSC	4819	0.20	0.65	0.76
ukb-a-275	rs7132908	50263148	12	LUSC	3538	0.01	0.97	0.99
ukb-a-278	rs10999460	72428283	10	LUSC	6915	0.15	0.71	0.82
ukb-a-278	rs12367809	50256063	12	LUSC	3534	0.01	0.98	0.99
ukb-a-278	rs1653892	32399469	7	LUSC	6186	0.20	0.57	0.74
ukb-a-278	rs3784699	68007591	15	LUSC	4819	0.20	0.64	0.76
ukb-a-279	rs329118	133861663	5	LUSC	4260	0.01	0.98	0.99
ukb-a-279	rs34517439	78450517	1	LUSC	5027	0.04	0.92	0.96
ukb-a-279	rs3784699	68007591	15	LUSC	4819	0.20	0.64	0.76
ukb-a-279	rs7132908	50263148	12	LUSC	3538	0.01	0.97	0.99
ukb-a-282	rs12367809	50256063	12	LUSC	3534	0.01	0.99	0.99
ukb-a-282	rs329118	133861663	5	LUSC	4260	0.02	0.97	0.98
ukb-a-282	rs3784699	68007591	15	LUSC	4819	0.21	0.64	0.76
ukb-a-283	rs12367809	50256063	12	LUSC	3534	0.01	0.98	0.99
ukb-a-283	rs329118	133861663	5	LUSC	4260	0.01	0.98	0.99
ukb-a-283	rs34517439	78450517	1	LUSC	5027	0.04	0.91	0.95
ukb-a-286	rs12367809	50256063	12	LUSC	3534	0.01	0.99	0.99
ukb-a-286	rs1653892	32399469	7	LUSC	6186	0.22	0.52	0.70
ukb-a-286	rs329118	133861663	5	LUSC	4260	0.01	0.98	0.99
ukb-a-287	rs12367809	50256063	12	LUSC	3534	0.01	0.98	0.99
ukb-a-287	rs329118	133861663	5	LUSC	4260	0.01	0.98	0.99
ukb-a-287	rs34517439	78450517	1	LUSC	5027	0.04	0.91	0.95
ukb-a-287	rs7723426	43186097	5	LUSC	4488	0.12	0.80	0.87
ukb-a-291	rs12367809	50256063	12	LUSC	3534	0.01	0.98	0.99
ukb-a-291	rs34517439	78450517	1	LUSC	5027	0.04	0.91	0.95
ukb-a-328	rs184589612	41412192	19	LUSC	4465	0.00	1.00	1.00
ukb-a-328	rs56113850	41353107	19	LUSC	4505	0.00	1.00	1.00

ukb-a-328	rs58365910	78849034	15	LUSC	5547	0.09	0.91	0.91
ukb-a-328	rs62012629	79070351	15	LUSC	5736	0.09	0.91	0.91
ukb-a-342	rs11671669	41324392	19	LUSC	4573	0.00	1.00	1.00
ukb-a-342	rs56113850	41353107	19	LUSC	4505	0.00	1.00	1.00
ukb-a-342	rs8034191	78806023	15	LUSC	5558	0.02	0.98	0.98
ukb-a-343	rs11697662	61992005	20	LUSC	6258	0.00	0.99	1.00
ukb-a-343	rs8034191	78806023	15	LUSC	5558	0.06	0.94	0.94
ukb-a-382	rs12367809	50256063	12	LUSC	3534	0.01	0.99	0.99
ukb-a-382	rs3784692	67988133	15	LUSC	4820	0.21	0.64	0.76
ukb-a-434	rs1333042	22103813	9	LUSC	5491	0.09	0.90	0.91
ukb-a-434	rs6857	45392254	19	LUSC	5254	0.09	0.69	0.88
ukb-a-434	rs7412	45412079	19	LUSC	5271	0.09	0.69	0.88
ukb-b-11842	rs329118	133861663	5	LUSC	4282	0.01	0.98	0.99
ukb-b-11842	rs34517439	78450517	1	LUSC	5054	0.05	0.91	0.95
ukb-b-11842	rs7132908	50263148	12	LUSC	3585	0.02	0.96	0.98
ukb-b-12039	rs329118	133861663	5	LUSC	4282	0.01	0.98	0.99
ukb-b-12039	rs34517439	78450517	1	LUSC	5054	0.05	0.91	0.95
ukb-b-12039	rs7132908	50263148	12	LUSC	3585	0.02	0.96	0.98
ukb-b-12854	rs10074873	43191823	5	LUSC	4520	0.12	0.80	0.87
ukb-b-12854	rs10935143	134665159	3	LUSC	4557	0.16	0.56	0.77
ukb-b-12854	rs12367809	50256063	12	LUSC	3581	0.01	0.98	0.99
ukb-b-12854	rs329118	133861663	5	LUSC	4282	0.01	0.98	0.99
ukb-b-14521	rs421284	1325590	5	LUSC	6845	0.03	0.97	0.97
ukb-b-14521	rs62012629	79070351	15	LUSC	5690	0.07	0.93	0.93
ukb-b-14521	rs72740955	78849779	15	LUSC	5503	0.07	0.93	0.93
ukb-b-15590	rs329118	133861663	5	LUSC	4282	0.01	0.98	0.99
ukb-b-15590	rs34517439	78450517	1	LUSC	5054	0.05	0.91	0.95
ukb-b-15590	rs7132908	50263148	12	LUSC	3585	0.01	0.96	0.98
ukb-b-16489	rs11690224	4952314	2	LUSC	6119	0.19	0.52	0.73
ukb-b-16489	rs329122	133864599	5	LUSC	4269	0.04	0.93	0.96
ukb-b-16878	rs12203592	396321	6	LUSC	3921	0.10	0.59	0.86
ukb-b-16878	rs72720396	91191582	1	LUSC	4976	0.13	0.83	0.87
ukb-b-17685	rs12405972	44097438	1	LUSC	4264	0.14	0.85	0.86
ukb-b-17685	rs6011779	61984317	20	LUSC	6330	0.07	0.87	0.92
ukb-b-18096	rs10935143	134665159	3	LUSC	4557	0.16	0.57	0.78
ukb-b-18096	rs329118	133861663	5	LUSC	4282	0.01	0.98	0.99
ukb-b-18096	rs329118	133861663	5	LUSC	4282	0.01	0.98	0.99
ukb-b-18096	rs34517439	78450517	1	LUSC	5054	0.05	0.91	0.95
ukb-b-18096	rs7132908	50263148	12	LUSC	3585	0.02	0.96	0.98
ukb-b-18377	rs13177679	42915470	5	LUSC	4262	0.21	0.64	0.75
ukb-b-18377	rs329118	133861663	5	LUSC	4282	0.02	0.97	0.98
ukb-b-18377	rs34517439	78450517	1	LUSC	5054	0.18	0.65	0.79
ukb-b-18377	rs3784699	68007591	15	LUSC	4845	0.20	0.66	0.77

ukb-b-18377	rs7132908	50263148	12	LUSC	3585	0.01	0.97	0.99
ukb-b-19379	rs34517439	78450517	1	LUSC	5054	0.05	0.91	0.95
ukb-b-19379	rs67177346	27439904	10	LUSC	5970	0.13	0.86	0.87
ukb-b-19379	rs7531110	65937065	1	LUSC	4741	0.05	0.91	0.95
ukb-b-19393	rs10935143	134665159	3	LUSC	4557	0.16	0.56	0.78
ukb-b-19393	rs329118	133861663	5	LUSC	4282	0.01	0.98	0.99
ukb-b-19393	rs34517439	78450517	1	LUSC	5054	0.05	0.91	0.95
ukb-b-19393	rs7132908	50263148	12	LUSC	3585	0.01	0.96	0.99
ukb-b-19921	rs329118	133861663	5	LUSC	4282	0.04	0.92	0.95
ukb-b-19921	rs34517439	78450517	1	LUSC	5054	0.05	0.91	0.95
ukb-b-19921	rs67425403	27439900	10	LUSC	5970	0.19	0.80	0.81
ukb-b-19921	rs7132908	50263148	12	LUSC	3585	0.05	0.89	0.95
ukb-b-19921	rs7531110	65937065	1	LUSC	4741	0.05	0.92	0.95
ukb-b-19953	rs13176429	43152216	5	LUSC	4477	0.12	0.80	0.87
ukb-b-19953	rs329118	133861663	5	LUSC	4282	0.01	0.98	0.99
ukb-b-19953	rs34517439	78450517	1	LUSC	5054	0.05	0.91	0.95
ukb-b-19953	rs34517439	78450517	1	LUSC	5054	0.04	0.91	0.95
ukb-b-19953	rs7132908	50263148	12	LUSC	3585	0.02	0.96	0.98
ukb-b-19953	rs76095247	66593468	15	LUSC	5342	0.18	0.66	0.79
ukb-b-20044	rs31251	130833946	5	LUSC	3405	0.13	0.52	0.80
ukb-b-20044	rs34517439	78450517	1	LUSC	5054	0.04	0.91	0.95
ukb-b-20044	rs7132908	50263148	12	LUSC	3585	0.01	0.97	0.99
ukb-b-20188	rs12367809	50256063	12	LUSC	3581	0.01	0.98	0.99
ukb-b-20188	rs13177679	42915470	5	LUSC	4262	0.13	0.79	0.86
ukb-b-20188	rs329124	133865452	5	LUSC	4271	0.01	0.98	0.99
ukb-b-20531	rs12050481	68023809	15	LUSC	4883	0.19	0.67	0.78
ukb-b-20531	rs34517439	78450517	1	LUSC	5054	0.21	0.57	0.73
ukb-b-20531	rs7132908	50263148	12	LUSC	3585	0.01	0.97	0.99
ukb-b-2303	rs10935143	134665159	3	LUSC	4557	0.19	0.50	0.73
ukb-b-2303	rs13176429	43152216	5	LUSC	4477	0.12	0.80	0.87
ukb-b-2303	rs329118	133861663	5	LUSC	4282	0.01	0.98	0.99
ukb-b-2303	rs34517439	78450517	1	LUSC	5054	0.05	0.91	0.95
ukb-b-2303	rs7132908	50263148	12	LUSC	3585	0.01	0.96	0.99
ukb-b-2303	rs76095247	66593468	15	LUSC	5342	0.19	0.64	0.77
ukb-b-2732	rs6011779	61984317	20	LUSC	6192	0.01	0.99	0.99
ukb-b-2732	rs8034191	78806023	15	LUSC	5490	0.15	0.85	0.85
ukb-b-469	rs11671669	41324392	19	LUSC	4567	0.00	1.00	1.00
ukb-b-469	rs56113850	41353107	19	LUSC	4496	0.00	1.00	1.00
ukb-b-6019	rs11671669	41324392	19	LUSC	4704	0.00	1.00	1.00
ukb-b-6019	rs2002403	78681002	15	LUSC	5459	0.08	0.92	0.92
ukb-b-6019	rs56113850	41353107	19	LUSC	4634	0.00	1.00	1.00
ukb-b-6019	rs6495304	78800416	15	LUSC	5593	0.08	0.92	0.92
ukb-b-6019	rs72740955	78849779	15	LUSC	5585	0.08	0.92	0.92

ukb-b-6019	rs72740955	78849779	15	LUSC	5585	0.08	0.92	0.92
ukb-b-6591	rs12907546	47684280	15	LUSC	4776	0.10	0.75	0.88
ukb-b-6591	rs3896224	106467853	10	LUSC	4774	0.11	0.65	0.86
ukb-b-6704	rs10935143	134665159	3	LUSC	4557	0.16	0.56	0.77
ukb-b-6704	rs329118	133861663	5	LUSC	4282	0.01	0.98	0.99
ukb-b-6704	rs34517439	78450517	1	LUSC	5054	0.04	0.91	0.95
ukb-b-6704	rs7132908	50263148	12	LUSC	3585	0.01	0.97	0.99
ukb-b-7212	rs10935143	134665159	3	LUSC	4557	0.16	0.57	0.78
ukb-b-7212	rs329118	133861663	5	LUSC	4282	0.01	0.98	0.99
ukb-b-7212	rs34517439	78450517	1	LUSC	5054	0.05	0.91	0.95
ukb-b-7212	rs7132908	50263148	12	LUSC	3585	0.02	0.96	0.98
ukb-b-7212	rs76095247	66593468	15	LUSC	5342	0.19	0.65	0.78
ukb-b-7460	rs2316205	41346768	19	LUSC	4667	0.00	1.00	1.00
ukb-b-7460	rs62012628	79070000	15	LUSC	5779	0.29	0.71	0.71
ukb-b-7460	rs8042849	78817929	15	LUSC	5596	0.23	0.77	0.77
ukb-b-7859	rs34517439	78450517	1	LUSC	5054	0.05	0.90	0.95
ukb-b-7859	rs67425403	27439900	10	LUSC	5970	0.12	0.88	0.88
ukb-b-7859	rs7531110	65937065	1	LUSC	4741	0.05	0.92	0.95
ukb-b-8338	rs10935143	134665159	3	LUSC	4557	0.16	0.56	0.77
ukb-b-8338	rs13176429	43152216	5	LUSC	4477	0.11	0.81	0.88
ukb-b-8338	rs329118	133861663	5	LUSC	4282	0.01	0.98	0.99
ukb-b-8338	rs34517439	78450517	1	LUSC	5054	0.05	0.91	0.95
ukb-b-8338	rs7132908	50263148	12	LUSC	3585	0.01	0.97	0.99
ukb-b-8755	rs1831733	22076071	9	LUSC	5474	0.14	0.85	0.86
ukb-b-8755	rs7412	45412079	19	LUSC	5277	0.15	0.52	0.78
ukb-b-8909	rs13177679	42915470	5	LUSC	4262	0.17	0.70	0.80
ukb-b-8909	rs329118	133861663	5	LUSC	4282	0.02	0.97	0.98
ukb-b-8909	rs7132908	50263148	12	LUSC	3585	0.01	0.98	0.99
ukb-b-9405	rs329118	133861663	5	LUSC	4282	0.01	0.98	0.99
ukb-b-9405	rs34517439	78450517	1	LUSC	5054	0.04	0.91	0.95
ukb-b-9405	rs7132908	50263148	12	LUSC	3585	0.01	0.98	0.99
ukb-d-I9_CORATHER	rs1333042	22103813	9	LUSC	5811	0.07	0.93	0.93
ukb-d-I9_CORATHER	rs769449	45410002	19	LUSC	5672	0.14	0.57	0.80
ukb-d-I9_IHD	rs1333042	22103813	9	LUSC	5811	0.07	0.92	0.92
ukb-d-I9_IHD	rs429358	45411941	19	LUSC	5674	0.09	0.73	0.89
